# Nanographenic bowls based on contorted hexabenzocoronene: Synthesis, structure, and supramolecular assembly with fullerene C_60_

**DOI:** 10.1126/sciadv.aed5921

**Published:** 2026-05-01

**Authors:** Yixun Sun, Bo Yang, Min Jia, Jing Guo, Yiting Wang, Jiale Hu, Xin Xu, Qingxiang Liang, Jingshuang Dang, Juan Fan, Jing Li, Huaming Sun, Junfa Wei

**Affiliations:** School of Chemistry and Chemical Engineering, Shaanxi Normal University, Xi’an 710119, China.

## Abstract

Buckybowls, the curved fragments of fullerenes, are attractive synthetic targets for exploring carbon nanomaterials, but constructing atomically well-defined, large multilayered architectures remains a formidable challenge. Here we report the synthesis of a class of coronene-bottomed nanographenic bowls, namely, tricarbon-annulated trifluorenocoronenes (TCTFCs), based on a contorted hexa-*cata*-hexabenzocoronene (*c*-HBC) skeleton. Two TCTFC derivatives bearing *gem*-diaryl or *gem*-dialkyl appendages were prepared via a “branching arms cyclization” (BAC) strategy. X-ray crystallography unambiguously confirms their C_3_-symmetric bowl-shaped geometry with bowl depths up to 2.3 angstrom and diameters exceeding 1 nanometer. These bowls exhibit red-shifted absorption/emission profiles, enhanced electron affinity, and reduced aromaticity relative to the *c*-HBC derivative. Notably, both compounds form 1:1 complexes with C_60_ in solution, while their cocrystals adopt columnar or honeycomb packing motifs. This work establishes a synthetic paradigm in constructing fully edge-closed *c*-HBC–based buckybowls, further delivering fundamental insights into their substituent-dependent host-guest binding and supramolecular assembly.

## Teaser

Fully edge-closed nanographenic bowls with C_3_ symmetry provide insights into host-guest chemistry with fullerene C_60_.

## INTRODUCTION

Buckybowls or π-bowls, whose nano-sized congeners are also referred to as nanographenic bowls, have attracted considerable interest due to their gorgeous architecture, intriguing properties (*1*–*7*), and promising applications in organic synthesis (*8*–*13*), coordination complexes (*14*–*18*), fullerene supramolecular chemistry (*19*–*25*), and optoelectronic materials (*26*–*32*). Nevertheless, the synthesis of buckybowls, particularly those of larger dimensions, remains a formidable challenge because the insistence of aromatic systems on planarity leads to severe strain in curved π-conjugated frameworks. Despite the great efforts dedicated to the design and synthesis of buckybowls and much progress has been achieved over the past few decades, the library of buckybowls reported in the literature is much smaller in comparison to their planar counterparts. To date, most of the reported carbon-based (*33*–*38*) or heteroatom-embedded buckybowls (*3*, *19*, *39*–*46*) derived from the corannulene (*47*) and sumanene (*48*) frameworks, the subunits of fullerene C_60_, have relatively small dimensions of π-surface. Examples of large nanographenic bowls with successive three-layered circumferential rings and complete topological rims are extremely rare (*24*, *31*, *32*, *49*–*51*). Extension of π-surfaces can substantially modulate the (opto-)electronic properties of buckybowls and enhance their host-guest interactions with fullerenes. From a broader perspective, achieving such large, curved π-systems may enable their use as structural templates for the controlled growth of carbon nanotubes (*52*, *53*). Accordingly, the design and synthesis of large buckylbowls is highly desirable, despite massive obstacles from the snowballing strain inherent in their multicircumscribed structures.

**Fig. 1.**
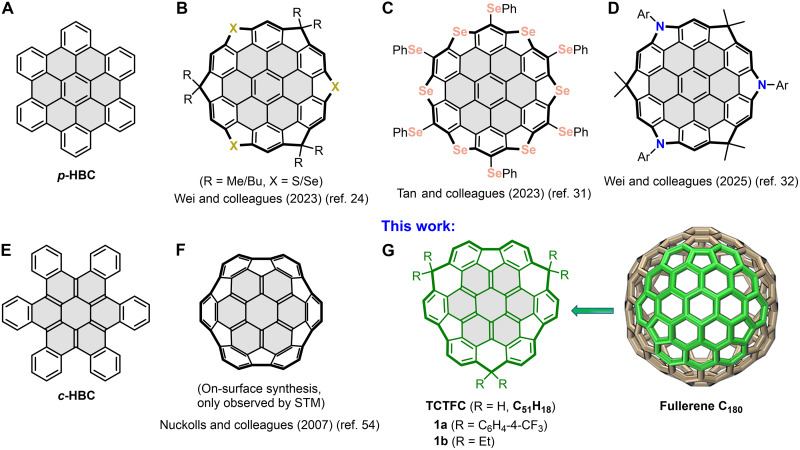
Chemical structures of representative HBC-based buckybowls and the target TCTFC. (**A**) Planar *p*-HBC skeleton. (**B**) Trichalcogenasupersumanene derivatives (*24*). (**C**) Hexaselannulated *p*-HBC derivative (*31*). (**D**) Triazasupersumanenes derivatives (*32*). (**E**) Doubly concave *c*-HBC skeleton. (**F**) Hemispherical *c*-HBC–based buckybowl generated via on-surface synthesis (*54*). (**G**) Solution-synthesized C_3_-symmetric *c*-HBC–based buckybowls reported in this work, along with the DFT-calculated tubular structure of fullerene C_180_. Coronene nuclei are highlighted in gray. STM, scanning tunneling microscopy.

Very recently, our group successfully synthesized two types of coronene-bottomed buckybowls based on the planar hexa-*peri*-hexabenzocoronene (*p*-HBC; Fig. 1A) skeleton, namely, trichalcogena- and triazasupersumanene derivatives (Fig. 1, B and D), and verified their distinctive properties, e.g., high fullerene-binding affinity, reversible mechanofluorochromic behavior, open-shell diradical dication characteristics, and unexpected p-type charge transport in organic field-effect transistors (*24*, *32*). Concurrently, Tan and coworkers (*31*) reported a hexaselenannulated *p*-HBC analog (Fig. 1C) synthesized via nucleophilic selenolation, which forms asymmetric one-dimensional (1D) columnar stacks in the solid state and exhibits a strong polarization-dependent second harmonic generation response. In stark contrast, doubly concave hexa-*cata*-hexabenzocoronene (*c*-HBC; Fig. 1E) skeleton, often regarded as the twin sister of *p*-HBC, has remained markedly underdeveloped as a platform for large-sized buckybowls with an intact rim. This disparity stems primarily from heightened synthetic challenges and steric constraints inherent to *c*-HBC–based architectures, particularly around the cove regions. A review of prior work on *c*-HBC–based buckybowls reveals two key efforts by Nuckolls and coworkers (*54**,* *55*). Initially, they reported an on-surface synthesis in which the parent *c*-HBC adsorbed on a Ru(0001) surface was converted into a hemispherical bowl-shaped product (Fig. 1F) through thermal activation (*54*). Unfortunately, the resulting species could only be observed via scanning tunneling microscopy, and its confinement to the metal surface prevented isolation and further structural analysis. Subsequently, the group pursued the synthesis of *c*-HBC–based buckybowls in solution via a mirror-symmetric design strategy (*55*). However, their synthesis yielded only partially notched structures, without progressing to a fully intact bowl geometry.

To address this long-standing challenge and expand the structural and synthetic diversity of *c*-HBC–based buckybowls, we developed a triple symmetry strategy termed “branching arms cyclization (BAC).” This approach enabled the successful construction of a C_3_-symmetric, fully edge-closed nanographenic bowl core, namely, tricarbon-annulated trifluorenocoronene (TCTFC; Fig. 1G), which comprises 51 carbon atoms and a 19-ring fused system. Moreover, such a polycyclic scaffold, consisting of a coronene (circumbenzene) nucleus circumscribed by alternating three sets of benzene-hexagon-benzene-pentagon motifs, represents a subunit of the giant fullerene C_180_ (*56*). Here, we report the bottom-up synthesis of two TCTFC derivatives (**1a** and **1b**). Their structures were unambiguously confirmed, revealing profound bowl depths and diameters exceeding 1 nm. We further present a comprehensive investigation into their structural, aromatic, conformational, photophysical, and electrochemical properties. Notably, these nanographenic bowls exhibit strong and substituent-dependent binding affinity for C_60_, forming stable 1:1 host-guest complexes in solution and yielding different supramolecular assembly motifs in the solid state. This work not only establishes a versatile synthetic paradigm for *c*-HBC–based buckybowls but also provides fundamental insights into the property evolution from doubly concave nanographenes to curved π-bowls and their potential in supramolecular chemistry.

## RESULTS

### Design, synthesis, and characterization

**Fig. 2.**
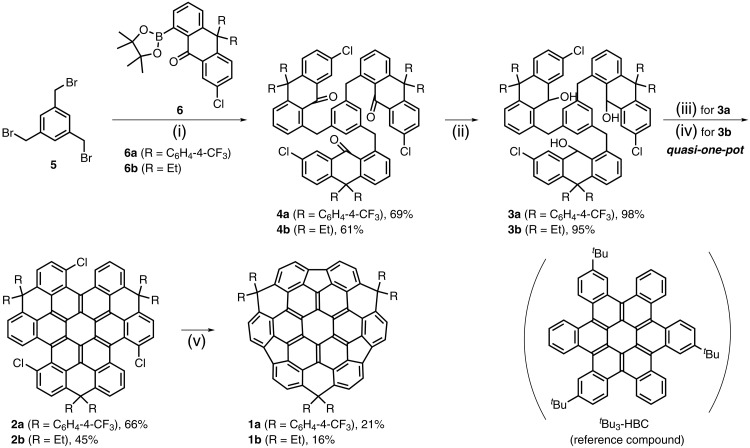
Synthetic route to nanographenic bowls 1a and 1b. Reagents and conditions: (i) Pd(PPh_3_)_4_, Cs_2_CO_3_, toluene/H_2_O, 80°C, overnight; (ii) NaBH_4_, THF/MeOH, r.t., 2 hours; (iii) For **3a**: 1) BF_3_·Et_2_O, dichloromethane (DCM), room temperature (r.t.), 2 hours; 2) DDQ, 1,2-dichloroethane (DCE), 80°C, 30 min; 3) TfOH, 0°C, 15 min; (iv) For **3b**: 1) BBr_3_, DCM, r.t., overnight; 2) DDQ, DCE, 80°C, 30 min; 3) TfOH, 0°C, 15 min; (v) PdCl_2_(PCy_3_)_2_, DBU, DMAc, 145°C, overnight.

The successful implementation of our BAC strategy is detailed in Fig. 2. A pivotal element of this synthetic design is the strategic introduction of three sp^3^-hybridized bridge carbons, serving as “molecular anchors” to lock the local positive curvature from three pentagons into a stable bowl conformation. These anchors are indispensable, as density functional theory (DFT) calculations revealed that the unbridged analog (trifluorenocoronene) would adopt a nearly planar conformation (fig. S61). Given that introducing such bridges directly into the sterically hindered cove regions of a preformed *c*-HBC skeleton is impractical, we rationally designed anthracenone-based building blocks **6** to preinstall these essential sp^3^ bridge carbon units at an early synthetic stage. In addition to providing the molecular backbone, these blocks were engineered with precisely positioned functional groups: boronic esters for coupling to the central core, carbonyls for annulation into the coronene nucleus, and chlorine atoms for pentagon formation at the future cove sites. Furthermore, to ensure solubility and prevent steric interference in the critical cyclization steps, *gem*-diaryl (**6a**) or dialkyl (**6b**) substituents were incorporated onto these prefunctionalized sp^3^ bridge carbons.

On the basis of this design, building blocks **6a** and **6b** were first prepared on a gram scale through optimized procedures (see section S1.2). A triple intermolecular Suzuki-Miyaura coupling of 1,3,5-tris(bromomethyl)benzene **5** with **6a** or **6b** afforded dendritic triketones **4a** (69% yield) and **4b** (61% yield). Subsequent NaBH_4_ reduction furnished the corresponding triols **3a** and **3b** in nearly quantitative yield. These triols were then subjected to a sequential *quasi*-one-pot transformation involving Lewis acid–mediated intramolecular Friedel-Crafts cyclization, 2,3-dichloro-5,6-dicyano-1,4-benzoquinone (DDQ) dehydrogenative aromatization, and DDQ/trifluoromethanesulfonic acid (TfOH)-promoted Scholl cyclization, yielding key precursors **2a** and **2b**. It is worth mentioning that **3a** cyclized smoothly using BF_3_.Et_2_O to give **2a** in 66% yield over three steps. In contrast, the analogous reaction for **3b** afforded only trace amounts of **2b** alongside substantial complex by-products. After careful optimization of the reaction conditions, we found BBr_3_ as a superior Lewis acid for **3b**, delivering **2b** in 45% yield. The structure of **2b** was unambiguously confirmed by x-ray crystallography (see section S2.1) in addition to high-resolution mass spectroscopy (HRMS) (fig. S120). In the final step, precursors **2a** and **2b** underwent intramolecular arylation using PdCl_2_(PCy_3_)_2_ catalyst in a 1,8-diazabicyclo[5.4.0]undecane-7-ene (DBU)/*N*,*N*-dimethylacetamide (DMAc) mixture at 145°C, furnishing the fully ring-fused targets. The cove-connected **1a** was isolated in 21% yield by preparative thin-layer chromatography, while **1b**, which exhibited limited solubility, was purified by repeated recrystallization to afford 16% yield.

**Fig. 3.**
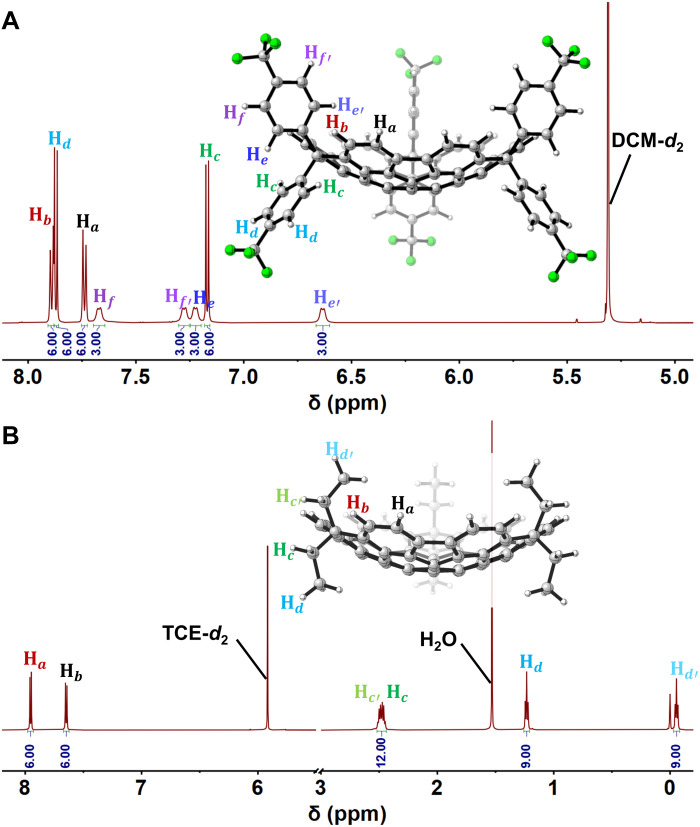
NMR spectroscopic characterization confirming the **C_3_**-symmetric bowl-shaped structures of 1a and 1b in solution. (**A**) ^1^H NMR spectrum (600 MHz, CD_2_Cl_2_, 298 K) of **1a**. (**B**) ^1^H NMR spectrum (600 MHz, C_2_D_2_Cl_4_, 298 K) of **1b**. Insets: DFT-optimized structures [M062X/6-31G(d,p)] with proton assignments. Full spectra are provided in the Supplementary Materials.

The structures of **1a** and **1b** were confirmed by comprehensive spectroscopic characterization, with the spectroscopic data being in agreement with the structures optimized by DFT calculations and further corroborated by x-ray crystallographic analysis (vide infra). Matrix-assisted laser desorption/ionization–time-of-flight–mass spectra (MALDI-TOF-MS) showed intense molecular ion peaks at mass/charge ratios (*m*/*z*) 1495.25484 for **1a** and 798.32776 for **1b**, consistent with the calculated molecular mass of 1495.25578 (C_93_H_36_F_18_) and 798.32810 (C_63_H_42_), respectively (figs. S127 and S133). The ^1^H nuclear magnetic resonance (NMR) spectra of both compounds exhibited well-resolved signals (Fig. 3, A and B), which were fully assigned using 2D NMR techniques (figs. S123, S124, S130, and S131) and clearly reflect their C_3_-symmetric bowl-shaped architecture in solution. Specifically, **1a** displayed six distinct aromatic signals corresponding to the 4-(trifluoromethyl)phenyl appendages (labeled H_c_, H_d_, H_e_, H_e′_, H_f_, and H_f′_; Fig. 3A), with splitting patterns reflecting their orthogonal orientation relative to the bowl framework. The convex-oriented aryl rings, perpendicular to the symmetry plane, gave two sets of signals, whereas the concave-oriented rings, aligned parallel to the plane and partitioned inward/outward relative to the bowl cavity, yielded four distinct resonances. Similarly, **1b** exhibited four characteristic signals in the aliphatic region, corresponding to the *gem*-diethyl groups (labeled H_c_, H_c′_, H_d_, and H_d′_ in Fig. 3B). The ^19^F NMR spectrum of **1a** exhibited two distinct resonance signals at δ = −62.78 and −62.86 parts per million (ppm), while the ^13^C NMR spectrum of compound **1b** displayed four signals in the range of δ = 8.30 to 42.05 ppm, further confirming the differentiating chemical environments of appendages on the concave and convex faces of the bowl system. Notably, the inwardly directed aromatic proton H_e′_ in **1a** (δ = 6.63 ppm) and the methyl group H_d′_ in **1b** (δ = −0.06 ppm) exhibited upfield shifts, consistent with the strong shielding effects of the curved π-surface. Moreover, the bowl rim proton H_b_ in **1a** was shifted downfield relative to H_a_, likely as a result of steric deshielding from the concave-oriented aryl groups.

### X-ray crystallographic analysis and structural properties

**Fig. 4.**
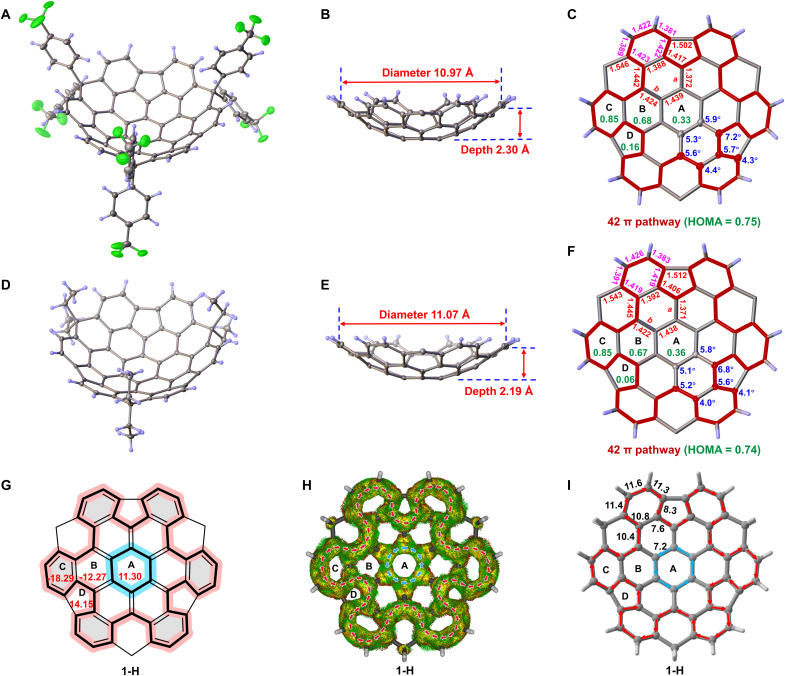
X-ray crystallographic structures and aromaticity. (**A** and **D**) Perspective views of **1a** and **1b** with thermal ellipsoids at 30 and 50% probability, respectively. (**B** and **E**) Side views of **1a** and **1b** showing bowl diameters and depths (red). (**C** and **F**) Top views of **1a** and **1b** displaying symmetry-averaged POAV angles (blue), bond lengths (in angstrom, red and pink), and HOMA values (green). Substituents are omitted in side and top views for clarity. (**G**) Calculated average NICS(1)*_zz_* values (in parts per million, red) for **1-H** [B3LYP/6-311G(d,p)] with Clar sextets shaded in gray. (**H**) ACID plot (isovalue = 0.04) of **1-H** [B3LYP/6-311G(d,p)] showing diatropic (red) and paratropic (blue) ring currents. (**I**) Current density strength (in nanoamperes per tesla) across selected bonds of **1-H** calculated using GIMIC [B3LYP/6-311G(d,p)].

X-ray crystallographic analysis unambiguously confirmed the *quasi*-C_3_-symmetric bowl-shaped geometries of **1a** and **1b** (Fig. 4, A and D). The observed deviations from ideal C_3_ symmetry can be attributed to intermolecular packing forces within the crystal lattice. Single crystals suitable for x-ray diffraction were obtained as orange needles via slow diffusion of methanol into **1a**/chloroform and **1b**/1,1,2,2-tetrachloroethane solutions, respectively. The depths and diameters of the bowls are 2.30 and 10.97 Å for **1a** (Fig. 4B) and 2.19 and 11.07 Å for **1b** (Fig. 4E). These measurements are defined by the perpendicular distance from the centroid of hub ring A to the mean plane of the peripheral aromatic carbons and the average horizontal distance between six pairs of carbon atoms at opposite vertices, respectively. The slightly greater bowl depth of **1a** compared to **1b** can be attributed to steric repulsion between the aryl groups and the TCTFC core, a finding consistent with the DFT-optimized structures (fig. S62). No π-π stacking interactions are observed in the molecular packing due to the steric hindrance imposed by the appendages. Instead, both compounds adopt an interlocking arrangement between adjacent molecules, which is stabilized by C─H…π interactions (figs. S9 and S13).

To quantitatively evaluate the molecular curvature, π-orbital axis vector (POAV) analysis (*57*) was performed on both bowls (Fig. 4, C and F). The central hub rings A displayed POAV angles ranging from 5.3° to 5.9° for **1a** and 5.1° to 5.8° for **1b**. Maximum POAV occurs at the carbon atoms of the five-membered rings adjacent to the hub, reaching 7.2° for **1a** and 6.8° for **1b**. These angles are smaller than those of classical buckybowls such as corannulene (8.4°) (*58*) and sumanene (8.7°) (*59*), indicating that the extended π-surface in the title compounds effectively disperses curvature strain, resulting in milder local bending.

Bond length analysis offers further insight into the structural distortions induced by the bowl-shaped geometry (Fig. 4, C and F), using the contorted tri-*tert*-butylhexabenzocoronene (*^t^*Bu_3_-HBC; Fig. 2) as a reference (see sections S1.2 and S2.4). In the two bowls, the C─C bonds within hub ring A (1.438 to 1.439 Å) closely match the standard C(sp^2^)─C(sp^2^) single bond length (1.45 Å) and are nearly identical to those in *^t^*Bu_3_-HBC (1.439 Å). In contrast, the radial bonds show clear deviations: bonds a (1.371 to 1.372 Å) are slightly shortened, while bonds b (1.422 to 1.424 Å) are elongated compared to typical aromatic C─C bonds (1.39 Å in benzene; 1.399 Å in *^t^*Bu_3_-HBC). The coronene rim displays moderately compressed bonds (**1a**: 1.388 to 1.442 Å; **1b**: 1.392 to 1.445 Å) relative to *^t^*Bu_3_-HBC (1.419 to 1.449 Å). Conversely, the bonds in the outer ring C (highlighted in pink) are generally elongated (**1a**: 1.381 to 1.423 Å; **1b**: 1.383 to 1.426 Å) compared to those in *^t^*Bu_3_-HBC (1.372 to 1.414 Å).

Correspondingly, the geometry-based aromaticity index, known as the “Harmonic Oscillator Model of Aromaticity” (HOMA) (*60*), was used to assess the aromaticity in **1a** and **1b** using crystallographic bond parameters (Fig. 4, C and F). The results revealed that the outer rings C exhibit dominant local aromaticity (HOMA = 0.85 for both compounds), while the middle rings B show moderate aromaticity (HOMA = 0.67 to 0.68). In contrast, hub rings A (HOMA = 0.33 to 0.36) and five-membered rings D (HOMA = 0.06 to 0.16) display anti/nonaromatic character. In addition, the peripheral 42π {4n + 2} electron conjugation pathway demonstrates Hückel global aromaticity (HOMA = 0.74 to 0.75). This aromatic distribution parallels that of *^t^*Bu_3_-HBC (fig. S15), likely due to the pronounced single-bond character (1.502 to 1.512 Å) of the C─C bonds connecting the outer benzene rings.

To further evaluate the aromaticity of the bowls, we conducted nucleus-independent chemical shift (NICS) (*61*), anisotropy of current-induced density (ACID) (*62*), and gauge-including magnetically induced current (GIMIC) (*63*) calculations on pristine *c*-HBC and model compound **1-H**, where the appendages were formally replaced by hydrogen atoms. The NICS(1)*_zz_* values of **1-H** validate strong aromaticity in the outer rings C (−18.29 ppm), moderate aromaticity in rings B (−12.27 ppm), and antiaromatic character in hub rings A (11.30 ppm) and pentagonal rings D (14.15 ppm) (Fig. 4G), consistent with HOMA trends. Relative to *c*-HBC, the bowl-shaped distortion in **1-H** reduces aromaticity in both the outer and middle rings (tables S18 and S19). ACID plot of **1-H** reveals a paratropic (anticlockwise) ring current in the central hub and a global diatropic (clockwise) current along the 42π periphery (Fig. 4H). This counter-rotating topological ring current pattern resembles that of *c*-HBC; however, the curvature in **1-H** diminishes electronic coupling between the hub and the rim (fig. S68). GIMIC calculations further support the weakened π-electron delocalization of **1-H**, as evidenced by its reduced global ring current strengths (7.6 to 11.6 nA/T; Fig. 4I) compared to those of *c*-HBC (10.4 to 13.2 nA/T; fig. S69).

### Conformational dynamics

**Fig. 5.**
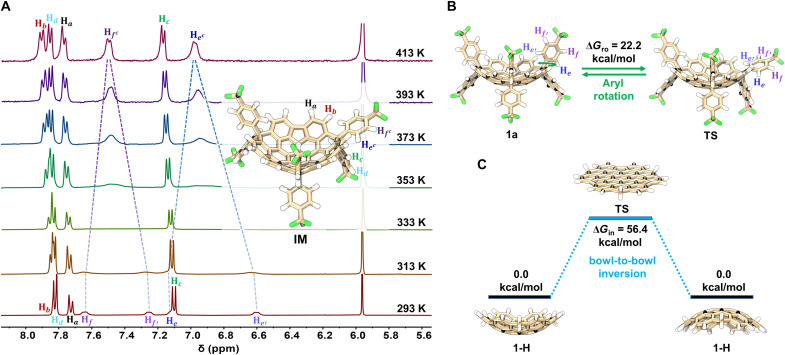
Conformational dynamics of 1a. (**A**) Variable-temperature ^1^H NMR spectra (400 MHz, C_2_D_2_Cl_4_, 293 to 413 K). Inset: Optimized structure of intermediate (IM) formed after 90° rotation of one concave aryl group [M062X/6-31G(d,p)]. (**B**) Calculated rotation barrier showing transition state (TS) for aryl group rotation [M062X/6-31G(d,p)]. (**C**) Calculated bowl-to-bowl inversion pathway and energy barrier for **1-H** [M062X/6-31G(d,p)].

We investigated the conformational dynamics of **1a** and **1b** by variable-temperature ^1^H NMR spectroscopy in combination with DFT calculations. The ^1^H NMR spectrum of **1a** in 1,1,2,2-tetrachloroethane-*d*_2_ at 293 K shows four distinct doublets at δ = 7.64, 7.25, 7.11, and 6.60 ppm, corresponding to the four magnetically inequivalent protons (H_f_, H_f′_, H_e_, and H_e′_) of the concave 4-(trifluoromethyl)phenyl substituents (Fig. 5, A and B). Upon heating, these signals broaden progressively and eventually coalesce at 413 K into two doublets at δ = 7.51 and 6.98 ppm, assigned to the time-averaged protons H_f_^c^ and H_e_^c^, respectively. Concurrently, the doublets associated with the convex aryl protons (H_c_ and H_d_) and rim aromatic protons (H_a_ and H_b_) shift downfield. These temperature-dependent spectral changes can be attributed to restricted rotation of the concave aryl groups around the inter-ring C─C bonds. In particular, even at 413 K, the aryl appendages of **1a** still give rise to four distinct signals (H_c_, H_d_, H_f_^c^, and H_e_^c^), rather than showing two equivalent *gem*-diaryls signals through bowl-to-bowl inversion. The experimental rotational barrier was determined to be 28.8 kcal/mol at 323 K using the Eyring equation (see section S3). DFT calculations further support a two-transition-state rotational pathway within a single potential energy well (fig. S64), yielding a calculated rotational barrier (Δ*G*_ro_) of 22.2 kcal/mol, in reasonable agreement with the experimental value.

Actually, both compounds exhibit exceptionally high bowl-to-bowl inversion barriers. Variable-temperature ^1^H NMR spectroscopy of **1b** in 1,2-dichlorobenzene-*d*_4_ showed no change in a signal pattern up to 443 K (fig. S17), confirming a rigid, inversion-locked conformation under ambient conditions. DFT calculations on simplified **1-H** reveal that the inversion mechanism proceeds through a planar transition state (Fig. 5C), analogous to those reported for corannulene (*64*) and sumanene (*2*). However, the calculated inversion barrier (Δ*G*_in_ = 56.4 kcal/mol) is considerably higher than those of corannulene (9.1 kcal/mol) and sumanene (16.3 kcal/mol). This marked increase can be rationalized by the deeper bowl depth of **1-H** (2.23 Å) compared to corannulene (0.87 Å) and sumanene (1.11 Å), resulting in greater strain accumulation during the inversion process.

### Photophysical and electrochemical properties

**Fig. 6.**
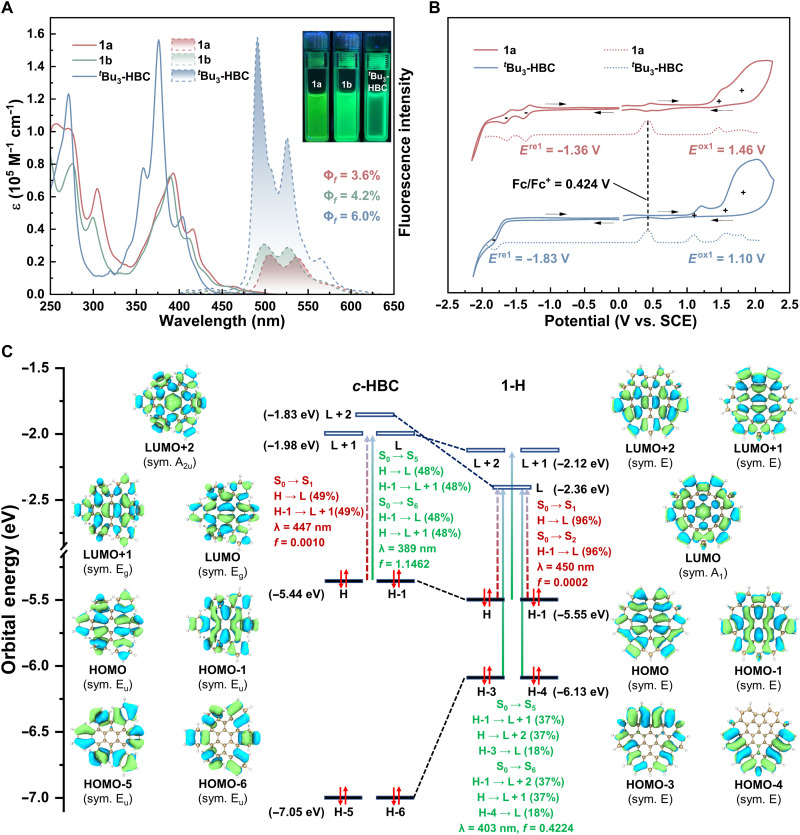
Photophysical and electrochemical characterization and TD-DFT calculations. (**A**) UV-Vis absorption (1.0 × 10^−5^ M, solid lines) and fluorescence emission spectra (1.0 × 10^−6^ M, dashed lines) of **1a**, **1b**, and ^*t*^Bu_3_-HBC measured in DCM. Inset: Fluorescence images under 365-nm irradiation. (**B**) Cyclic voltamograms (solid lines) and differential pulse voltamograms (dashed lines) of **1a** and ^*t*^Bu_3_-HBC in CH_2_Cl_2_ (0.1 M *n-*Bu_4_NPF_6_) at a scan rate of 0.1 V/s. All potentials were calibrated against the saturated calomel electrode (SCE) by the addition of ferrocene as an internal standard, taking *E*_1/2_(Fc/Fc^+^) = 0.424 V versus SCE (*73*). (**C**) Orbital correlation diagrams (isovalue = 0.02) and the TD-DFT results for S_0 → 1_/S_2_/S_5_/S_6_ transitions of *c*-HBC and **1-H** [B3LYP/6-311G(d,p)].

The photophysical characteristics of **1a** and **1b** were investigated in dichloromethane and compared with those of the reference compound *^t^*Bu_3_-HBC (Fig. 6A). Ultraviolet-Visible (UV-Vis) absorption spectra of **1a** and **1b** exhibit distinct β-band maxima at 393 nm (ε = 0.75 × 10^5^ M^−1^ cm^−1^) and 390 nm (ε = 0.73 × 10^5^ M^−1^ cm^−1^), respectively, which are red-shifted by 14 to 17 nm relative to that of *^t^*Bu_3_-HBC (376 nm, ε = 1.57 × 10^5^ M^−1^ cm^−1^). This bathochromic shift is accompanied by a ~50% decrease in absorption intensity. All three compounds display a weak, broad shoulder around 460 nm (p-bands) and a low-energy absorption tail (α-band) extending beyond 500 nm. On the basis of the onset wavelengths of the p-bands (*65*), the optical energy gaps ($E_{g}^{\text{opt}}$) were estimated to be 2.48 eV for **1a** and 2.52 eV for **1b**, both narrower than that of *^t^*Bu_3_-HBC (2.61 eV) (table S5).

To gain deeper electronic insight, time-dependent DFT (TD-DFT) calculations were performed on **1-H** and *c*-HBC at the B3LYP/6-311G(d,p) level (Fig. 6C). The results reveal different transition patterns: **1-H** exhibits symmetry-allowed transitions predominantly from highest occupied molecular orbital (HOMO)/HOMO-1 to lowest unoccupied molecular orbital (LUMO)+1/LUMO+2 and from HOMO-3/HOMO-4 to LUMO (i.e., S_0 → 5_/S_6_, *f* = 0.4224), alongside symmetry-forbidden HOMO/HOMO-1 to LUMO transitions (S_0 → 1_/S_2_, *f* = 0.0002). In contrast, *c*-HBC shows intense HOMO/HOMO-1 to LUMO/LUMO+1 transitions (S_0 → 5_/S_6_, *f* = 1.1462) and forbidden transitions (S_0 → 1_, *f* = 0.0010). The reduced oscillator strength and altered transition patterns in **1-H** align with the experimentally observed decrease in absorption intensity.

All three compounds exhibit similar yellow-green fluorescence in dichloromethane (Fig. 6A), with two emission maxima at 506 and 536 nm for **1a**, 499 and 527 nm for **1b**, and 491 and 526 nm for *^t^*Bu_3_-HBC. The bowls show slight red shifts but obviously decreased emission intensity compared to ^*t*^Bu_3_-HBC, mirroring the trend observed in their UV-Vis absorption spectra. Moreover, both the absolute quantum yields and fluorescence lifetimes are reduced for **1a** (Φ = 3.6%, τ = 4.7 ns) and **1b** (Φ = 4.2%, τ = 5.2 ns) compared to ^*t*^Bu_3_-HBC (Φ = 6.0%, τ = 18.7 ns).

Following the solution-phase studies, we investigated the aggregation-dependent emission behavior of **1a** and **1b** in THF/water mixtures by progressively increasing the water fraction under constant solute concentration conditions. Both compounds ultimately exhibit aggregation-induced emission enhancement (AIEE), characterized by a pronounced rise in their Φ values at high water fractions (figs. S21 and S22). This enhancement can be attributed to restricted molecular motion and the suppression of nonradiative decay pathways in the aggregated state. Notably, **1b** displays a more continuous and spectrally resolved AIEE profile compared to **1a**. Its Φ value rises steadily across the aggregation process, accompanied by a clear red shift in emission when the water content reaches 80 to 90 vol %. The AIEE behavior is further corroborated by solid-state measurements (fig. S20), which show higher Φ values for both compounds (**1a**: 5.6%; **1b**: 6.9%) than those obtained in solution.

Electrochemical properties were investigated only for **1a** using cyclic voltammetry (CV) and differential pulse voltammetry (DPV) measurements in dichloromethane. The CV curve of **1a** shows two irreversible oxidation waves at positive potentials and two reversible reduction waves at negative potentials, whereas *^t^*Bu_3_-HBC displayed three irreversible oxidation waves and one irreversible reduction wave (Fig. 6B). From DPV measurements, the first oxidation and reduction half-wave potentials of **1a** were determined to be *E*^ox1^ = 1.46 V and *E*^re1^ = −1.36 V, respectively. Both values are shifted anodically relative to those of *^t^*Bu_3_-HBC (*E*^ox1^ = 1.10 V and *E*^re1^ = −1.83 V). Using the onset potentials from the CV curves, the HOMO/LUMO energy levels were estimated to be −5.67/−3.14 eV for **1a** and −5.44/−2.72 eV for *^t^*Bu_3_-HBC. Thus, **1a** shows a slightly lowered HOMO and a notably stabilized LUMO, resulting in a smaller electrochemical energy gap ($E_{g}^{\text{ele}}$) of 2.53 eV compared to 2.72 eV for *^t^*Bu_3_-HBC (table S6).

DFT calculations on **1-H** and *c*-HBC further elucidate these electronic changes (Fig. 6C). The HOMO levels of **1-H** (−5.55 eV) and *c*-HBC (−5.44 eV) are comparable, consistent with their analogous orbital configurations. In contrast, the LUMO of **1-H** (−2.36 eV) is markedly stabilized compared to that of *c*-HBC (−1.98 eV). Orbital analysis indicates that the LUMO of **1-H** originates from the LUMO+2 of *c*-HBC, wherein the bowl-shaped architecture of **1-H** endows new local orbital overlaps that enhance electron affinity. As a result, the calculated HOMO-LUMO gap of **1-H** (3.19 eV) is smaller than that of *c*-HBC (3.46 eV), in qualitative agreement with the experimental electrochemical and optical energy gaps.

### Host-guest chemistry with C_60_

**Fig. 7.**
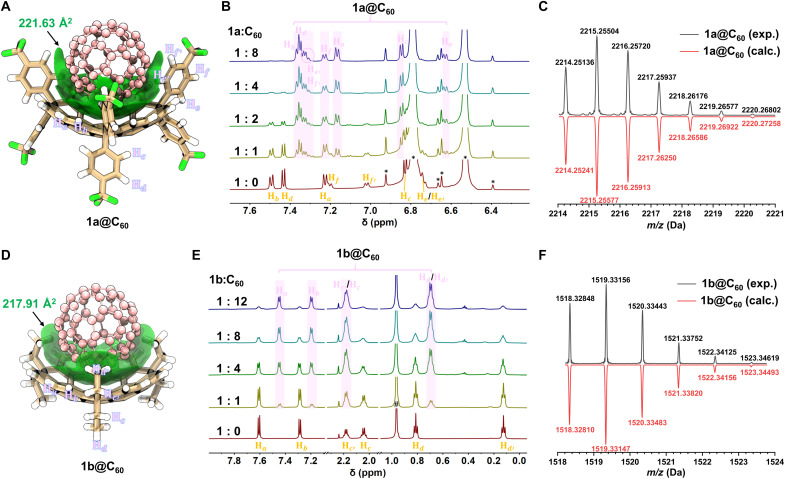
Host-guest interactions with C_60_. (**A** and **D**) DFT-optimized geometries of complexes **1a@C**_**60**_ and **1b@C**_**60**_, with the IGMH isosurface [0.002 atomic units (a.u.)] are shown to reveal main interaction regions between bowls and fullerene [B3LYP-D3(BJ)/6-31+G(d,p)]. (**B** and **E**) ^1^H NMR titration experiments showing spectral changes of **1a** and **1b** (0.5 mM in *o*-DCB-*d*_4_) upon addition of increasing amounts of C_60_ (600 MHz, 298 K).*Solvent and satellite peaks; #water peak. (**C** and **F**) MALDI-TOF-MS of **1a@C**_**60**_ and **1b@C**_**60**_.

The intrinsic shape and size complementarity between the concave surfaces of buckybowls **1a**/**1b** and the spherical fullerene C_60_ confers potential host-guest binding affinity, which was initially investigated through ^1^H NMR titration experiments (Fig. 7, B and E). When C_60_ was titrated into 1,2-dichlorobenzene-*d*_4_ solutions containing either receptor (*c* = 5.0 × 10^−4^ M), new sets of proton resonances distinct from those of free **1a** or **1b** appeared, indicating slow-exchange complexation on the NMR timescale. MALDI-TOF-MS analyses confirmed a 1:1 binding stoichiometry, with intense *m*/*z* peak signals at 2215.25631 (**1a@C**_60_; Fig. 7C and fig. S25) and 1519.33156 (**1b@C**_60_; Fig. 7F and fig. S31) detected. Furthermore, to further verify the formation of a well-defined, larger assembly in solution, diffusion-ordered spectroscopy (DOSY) experiments were performed in toluene-*d*_8_ (figs. S29 and S34). The DOSY spectra revealed a uniform decrease in the diffusion coefficients for the aromatic proton signals of **1a** and **1b** upon complexation with C_60_, directly confirming the formation of the respective **1a@C**_60_ and **1b@C**_60_ host-guest complexes. Noncovalent interaction regions were visualized using the independent gradient model based on Hirshfeld partition (IGMH) (Fig. 7, A and D) (*66*, *67*), which revealed extensive van der Waals contacts between C_60_ surface and both the concave π-surface and the inwardly oriented substituents of the hosts. These computational results align with the experimentally observed NMR chemical shift changes, whose assignments were confirmed by 2D NMR spectroscopy (figs. S26 to S28, S32, and S33). Specifically, bowl rim protons (H_a_ and H_b_) exhibited upfield shifts, while inward-oriented concave protons (H_e′_, H_f′_ in **1a**; H_d′_ in **1b**) showed downfield shifts. An earlier titration endpoint in the NMR experiments indicated that **1a** exhibits a superior binding affinity toward C_60_ compared to **1b**. This enhanced affinity can be structurally rationalized by a deeper cavity of **1a** offering improved geometric complementarity with C_60_, coupled with concave aryl groups that allow for spatially favorable C─H…π contacts. DFT calculations provide quantitative support for this binding affinity disparity, with the IGMH isosurface area of **1a@C**_60_ (221.63 Å^2^) larger than that of **1b@C**_60_ (217.91 Å^2^) (Fig. 7, A and D) and a more favorable interaction energy for **1a@C**_60_ (−45.7 kcal/mol) compared to **1b@C**_60_ (−41.9 kcal/mol) (table S22). Energy decomposition analysis using the sobEDAw method (*68*) further clarifies the origin of energy difference between the two complexes (table S22). The electrostatic interactions are nearly identical in both systems, ruling them out as a contributing factor. Although the **1a@C**_60_ complex exhibits slightly higher exchange-repulsion than **1b@C**_60_, this destabilizing effect is fully overcompensated by stronger dominant dispersion interactions, complemented by more favorable orbital interactions. Thus, the superior binding affinity of **1a** is electronically rooted in its ability to maximize both dispersion and orbital stabilization with the C_60_ guest.

To quantitatively assess the binding affinity for C_60_, we performed UV/Vis titration experiments on **1a** and **1b** in toluene (*c* = 3.0 × 10^−6^ M) (fig. S37). Although no clear isosbestic points or charge-transfer bands were observed during the titrations, distinct nonadditive spectral changes were evident. After subtracting the contribution of free C_60_ at each titration point, progressive variations at 307 nm (for **1a**) and 302 nm (for **1b**) could be discerned, indicating host-guest complexation. Job plot analyses supported a 1:1 binding stoichiometry in both cases (figs. S35 and S36). Considering that the Job plot method may not always be reliable to establish the stoichiometry of the host-guest complex (*69*), alternative 2:1 binding models were also evaluated (tables S8 and S9). However, these models were statistically excluded on the basis of insufficient goodness-of-fit parameters (*70*). Therefore, the titration data were fitted with a 1:1 model using the BindFit program (*71*), yielding binding constants *K*_a_ of (2.31 ± 0.01) × 10^3^ M^−1^ for **1a@C**_60_ and (1.59 ± 0.002) × 10^3^ M^−1^ for **1b@C**_60_. The higher affinity of **1a** aligns with trends from ^1^H NMR titrations and DFT calculations. Notably, the C_60_-binding capabilities are substantially higher than those of classical hydrocarbon bowls such as corannulene and sumanene, whereas the latter show negligible complexation in solution. This enhancement underscores that extending the π-conjugated surface of the bowl is an effective strategy for strengthening fullerene binding, positioning our *c*-HBC–based systems among the most promising hydrocarbon buckybowl receptors reported to date (*21*–*23*, *28*).

As a complement, fluorescence titration experiments were performed (fig. S51). Incremental addition of C_60_ into the solution of **1a** or **1b** in toluene (*c* = 1.5 × 10^−6^ M) resulted in gradual quenching of fluorescence intensity, indicating the formation of complexes. The quenching was more pronounced for **1a** than for **1b**. Job plot analyses from the fluorescence data also indicated a 1:1 stoichiometry (figs. S48 and S49). Fitting the fluorescence quenching data (*F*_0_/*F* versus [C_60_]) to a 1:1 Stern-Volmer model (*72*) gave apparent association constants substantially larger than those derived from UV/Vis titrations (table S11). This discrepancy is attributed to the concomitant dynamic collisional quenching and the inner filter effect of C_60_ at the excitation/emission wavelengths, which collectively amplify the observed quenching efficiency. Therefore, while the fluorescence titrations provide strong supplementary evidence for complexation, the derived constants are considered overestimated. The UV/Vis titration data are presented as the more reliable quantitative measure of the binding affinity.

### Solid-state cocrystal structures with C_60_

**Fig. 8.**
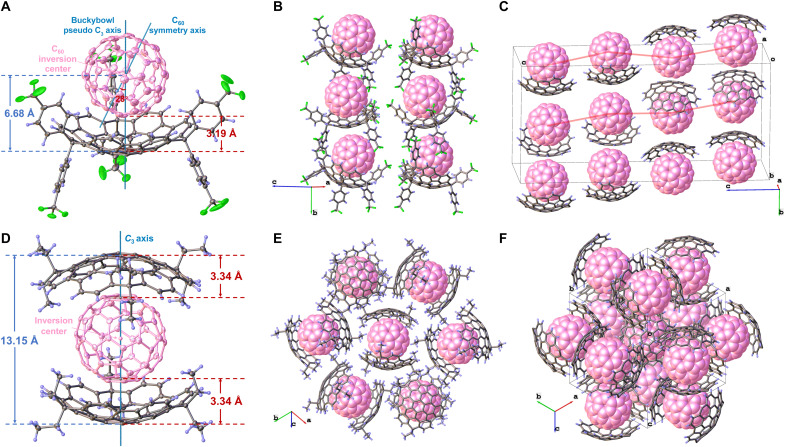
X-ray crystallographic structures of C_60_ complexes. (**A** and **D**) The side views of **1a@C**_**60**_ and **(1b)**_**2**_**@C**_**60**_ with 30% probability thermal ellipsoids. (**B** and **E**) Supramolecular packing arrangements of **1a@C**_**60**_ and **(1b)**_**2**_**@C**_**60**_ in the crystal lattice. (**C** and **F**) Asymmetric units of **1a@C**_**60**_ and **(1b)**_**2**_**@C**_**60**_ with substituents omitted for clarity.

Black-colored block cocrystals suitable for single-crystal x-ray diffraction were successfully obtained from equimolar mixtures of **1a** and C_60_ in benzene and **1b** and C_60_ in toluene. Crystallographic analysis revealed a 1:1 stoichiometric ratio for **1a@C**_60_ but a 2:1 ratio for **(1b)**_2_**@C**_60_ (Fig. 8). The solid-state 2:1 stoichiometry of **1b** and C_60_ differs from their 1:1 complexation in solution, suggesting that crystallization induces a molecular reorganization which is driven by optimized lattice packing effects that stabilize the **(1b)**_2_**@C**_60_ assembly.

In the **1a@C**_60_ cocrystal, the C_60_ guest is nestled near the center of the bowl-shaped cavity of **1a**, exhibiting rotational disorder with its symmetry axis tilted by 17° to 28° relative to the pseudo-C_3_ axis of the host (Fig. 8A and fig. S52). Concave-convex π-π interactions are evidenced by a penetration depth of 6.68 Å (hub ring centroid to C_60_ center) and a close contact of 3.19 Å (hub ring plane to C_60_ surface). Additional stabilization comes from multiple C─H…π contacts involving the inwardly oriented aryl protons H_e′_ and H_f′_, with average distances of 6.73 and 7.19 Å from their bonded carbon atoms to the C_60_ center (fig. S53). The **1a@C**_60_ cocrystal crystallizes in the orthorhombic system and Pbca space group, containing eight complete complexes per unit cell. Along the (100) crystallographic direction, adjacent **1a@C**_60_ complexes exhibit continuous staggered columnar stacking (Fig. 8B), a motif not previously reported in buckybowl-fullerene systems. This ordered stacking is stabilized by convex-convex π-π interactions, C─F…π interactions, and intermolecular C─F…H hydrogen bonds (fig. S54). Moreover, these C─F…π interactions induce conformational adaptation in the aryl appendages, causing two of the three aryl pairs to deviate from their orthogonal geometry in the uncomplexed **1a** (fig. S52). The columns further organize into a 2D zigzag lamellar structure through directional hydrogen bonding networks. Neighboring columns with the same orientation connect via dual C─F…H_a_ bonds, while centrosymmetrically related column pairs of opposite orientation are linked through three distinct hydrogen bond types (fig. S55). Collectively, these interactions propagate in three dimensions to form a highly ordered hierarchical framework (Fig. 8C and fig. S56).

In the **(1b)**_2_**@C**_60_ cocrystal, two crystallographically identical **1b** hosts adopt a sandwich-like arrangement above and below the C_60_ guest, forming a ternary complex with perfect I_3_ symmetry (Fig. 8D). The entire assembly shares a common C_3_ axis and an inversion center. Concave-convex π-π interactions are characterized by a centroid-to-centroid distance of 3.34 Å, measuring from the parallel alignment of the hub rings of **1b** with the six-membered rings of C_60_. The interhub distance is 13.15 Å, with a penetration depth of 6.58 Å. Additional C─H…π contacts, with an average distance of 3.35 Å, are observed between the inwardly directed methyl carbons and the C_60_ surface (fig. S58). These interactions collectively restrain the rotation of C_60_, leading to the absence of rotational disorder in the cocrystal. The cocrystal **(1b)**_2_**@C**_60_ adopts a cubic crystal system and Pa$\bar{3}$ space group, with four complete complexes existing in a unit cell. In the (110) crystal orientation, six cocrystal molecules surround a central one in a windmill-shaped symmetrical arrangement, forming a 2D hexagonal honeycomb sheet (Fig. 8E). These units are interconnected via C─H…π interactions (2.84 Å) involving methyl protons (H_d_) and the convex π-surface of C_60_ (fig. S59). In three dimensions, these honeycomb layers stack in different directions to generate a highly ordered and complex 3D honeycomb lattice that maximizes packing efficiency (Fig. 8F).

## DISCUSSION

In summary, we have developed a BAC strategy that enables efficient construction of a new class of large, compact, C_3_-symmetric, and coronene-bottomed nanographenic bowls. This solution-phase approach successfully addresses the long-standing challenge of constructing fully edge-closed buckybowls derived from the *c*-HBC skeleton. The resulting TCTFC derivatives (**1a** and **1b**) exhibit profound bowl depths and diameters, along with narrowed energy gaps, enhanced electron affinity, and red-shifted optical profiles relative to *c*-HBC analogs. Furthermore, both bowls display distinct AIEE in THF/water mixtures, a property stemming from restricted molecular motion in the aggregated state. These nanographenic bowls also form 1:1 complexes with C_60_ in solution, with binding affinity and solid-state packing motifs being finely modulated by their peripheral substituents. The BAC strategy, complemented by the fundamental insights gained from this study, opens avenues for the for designing triheteroannulated nanobowls, extended π-surface architectures, and other curved nanocarbons with tailored electronic and supramolecular properties.

## MATERIALS AND METHODS

All air- or moisture-sensitive reactions were performed under an argon atmosphere using standard Schlenk techniques. Unless otherwise indicated, all commercially available starting materials and dry solvents were purchased and used directly without further purification. Reaction progress was monitored by thin-layer chromatography on silica gel GF-254 plates. Column chromatography was performed with 200- to 300-mesh silica gel using a flash column. 2-Chloro-6-iodobenzaldehyde (**13**) and bis(4-(trifluoromethyl)phenyl)methanone (**10a**) were synthesized according to literature procedures (*73*, *74*). Detailed synthetic procedures for all intermediates and target compounds are provided in the Supplementary Materials. NMR spectra (^1^H, ^13^C, and ^19^F) were recorded on JEOL 400 MHz or Bruker Avance 600 MHz spectrometers. HRMS were obtained on a Bruker maXis spectrometer using atmospheric pressure chemical ionization (APCI) in TOF mode. MALDI-TOF-MS analyses were performed on a Bruker solariX XR FT-ICR mass spectrometer using *trans*-2-[3-(4-*tert*-butylphenyl)-2-methyl-2-propenylidene]malononitrile (DCTB) as matrix. Single-crystal x-ray diffraction data were collected on a Bruker D8 Venture diffractometer. UV-Vis absorption spectra were recorded on a PerkinElmer Lambda 1050 spectrophotometer. Fluorescence spectra were measured on a Horiba JY Fluorolog-3 spectrometer. Absolute fluorescence quantum yields were determined using a Hamamatsu C9920-02G system. Fluorescence lifetimes were measured on an FLSP920 spectrophotometer. CV and DPV were performed on a CHI 660B electrochemical analyzer at room temperature under an inert atmosphere. A three-electrode configuration was used in CH_2_Cl_2_ containing 0.1 M *n*-Bu_4_NPF_6_ as supporting electrolyte, with a platinum disc working electrode, platinum plate counter electrode, and silver wire quasi-reference electrode. Potentials were calibrated against a saturated calomel electrode (SCE) using ferrocene as an internal standard [*E*_1/2_(Fc/Fc^+^) = 0.424 V versus SCE] (*75*). DFT calculations were performed using the Gaussian 16, Revision A.03 program package (*76*).

## Supplementary Materials

### This PDF file includes:

Supplementary Text Figs. S1 to S141 Tables S1 to S32 References
