# Supplementary material for: Nanographenic bowls based on contorted hexabenzocoronene: Synthesis, structure, and supramolecular assembly with fullerene C60
Source: Sci Adv. 2026 May 1;12(18):eaed5921. doi: 10.1126/sciadv.aed5921 (PMC13134635; doi:10.1126/sciadv.aed5921)
Supplement: Supplementary file 1 — Supplementary Text Figs. S1 to S141 Tables S1 to S32 References [file sciadv.aed5921_sm.pdf]

Science Advances
AAAS

Supplementary Materials for

Nanographenic bowls based on contorted hexabenzocoronene: Synthesis, structure, and supramolecular assembly with fullerene C_{60}

Yixun Sun *et al.*

Corresponding author: Huaming Sun, hmsun@snnu.edu.cn; Junfa Wei, weijf@snnu.edu.cn

Sci. Adv. **12**, eaed5921 (2026)
DOI: 10.1126/sciadv.aed5921

This PDF file includes:

Supplementary Text
Figs. S1 to S141
Tables S1 to S32
References

1. Experimental Section

1.1 General information

5-chloro-2-iodobenzaldehyde (**13**) (*73*) and bis(4-(trifluoromethyl)phenyl)methanone (**10a**) (*74*) were synthesized according to literature procedures. All other reagents and starting materials were obtained from commercial suppliers and used without further purification unless otherwise noted. All air or moisture-sensitive reactions were carried out under an argon atmosphere by standard Schlenk techniques. Unless otherwise indicated, all commercially available starting materials and dry solvents were purchased and used directly without further purification. Reaction progress was monitored via analytical thin-layer chromatography (TLC, GF-254). Column chromatography was performed on silica gel (200-300 mesh) using freshly distilled solvents.

NMR spectra (^{1}H, ^{13}C, and ^{19}F) were recorded on a JEOL 400 MHz or a Bruker Avance 600 MHz spectrophotometer using chloroform-*d*, dichloromethane-d_2, 1,1,2,2-tetrachloroethane-d_2, or 1,2-dichlorobenzene-d_4 as a solvent. High-resolution mass spectra (HRMS) were recorded on a Bruker Avance spectrometer (maXis) operating in APCI positive/negative ion mode with TOF analysis. Matrix-assisted laser desorption/ionization time-of-flight mass spectra (MALDI-TOF-MS) were collected using a Bruker Fourier-transform Ion Cyclotron Resonance Mass Spectrometer (solariX XR) with trans-2-(3-(4-(*t*-butyl)phenyl)-2-methylallylidene)malononitrile (DCTB) in chloroform as the supporting matrix. Crystal structures were determined using a Bruker D8 Venture X-ray single crystal diffraction spectrometer.

UV-vis absorption spectra were measured on a PerkinElmer Lambda 1050 UV-visible near-infrared spectrometer. Fluorescence spectra were recorded on a Horiba JY Fluorolog-3 fluorescence spectrophotometer (USA). Fluorescence lifetimes were determined using a FLSP920 fluorescence spectrophotometer. Absolute fluorescence quantum yields were measured at room temperature using a HAMAMATSU Absolute PL Quantum Yield Measurement System C9920-02G.

Cyclic voltammetry (CV) and differential pulse voltammetry (DPV) were performed on a CHI 660B electrochemical analyzer at room temperature under an inert atmosphere. A three-electrode configuration was employed in CH_2Cl_2 solution (purchased from Sigma-Aldrich) containing the analyte (2×10^{-3} M) and 0.1 M tetrabutylammonium hexafluorophosphate (n-Bu_4NPF_6) as the supporting electrolyte. A platinum disc, a platinum plate, and a silver wire electrode served as the working electrode, the counter electrode, and the quasi-reference electrode (QRE), respectively. All potentials were calibrated against an aqueous SCE by the addition of ferrocene as an internal standard, taking $E_{1/2}(Fc/Fc^+) = 0.424$ V *vs*. SCE (*75*). The scan rate was 0.1 V/s. The HOMO and LUMO energy values were estimated from the onset potentials of the first oxidation and reduction events, respectively. The HOMO and LUMO energy levels were calculated according to the following equations:

$$E_{HOMO}\ (\text{eV}) = -\ [E_{onset}^{ox}\ (vs\ \text{SCE}) - E_{1/2}(\text{Fc/Fc}^+) + 4.8] = -\ [E_{onset}^{ox}\ (vs\ \text{SCE}) + 4.376]$$

$$E_{LUMO}\ (\text{eV}) = -\ [E_{onset}^{re}\ (vs\ \text{SCE}) - E_{1/2}(\text{Fc/Fc}^+) + 4.8] = -\ [E_{onset}^{re}\ (vs\ \text{SCE}) + 4.376]$$

Where $E_{1/2}(Fc/Fc^+)$ is the half-wave potential of the Fc/Fc^+ couple against the SCE electrode.

Theoretical calculations were carried out using the *Gaussian 16, Revision A.03* program (*76*).

1.2 Synthetic procedures and characterization data

Fig. S1. Synthesis route of building blocks 6a and 6b.

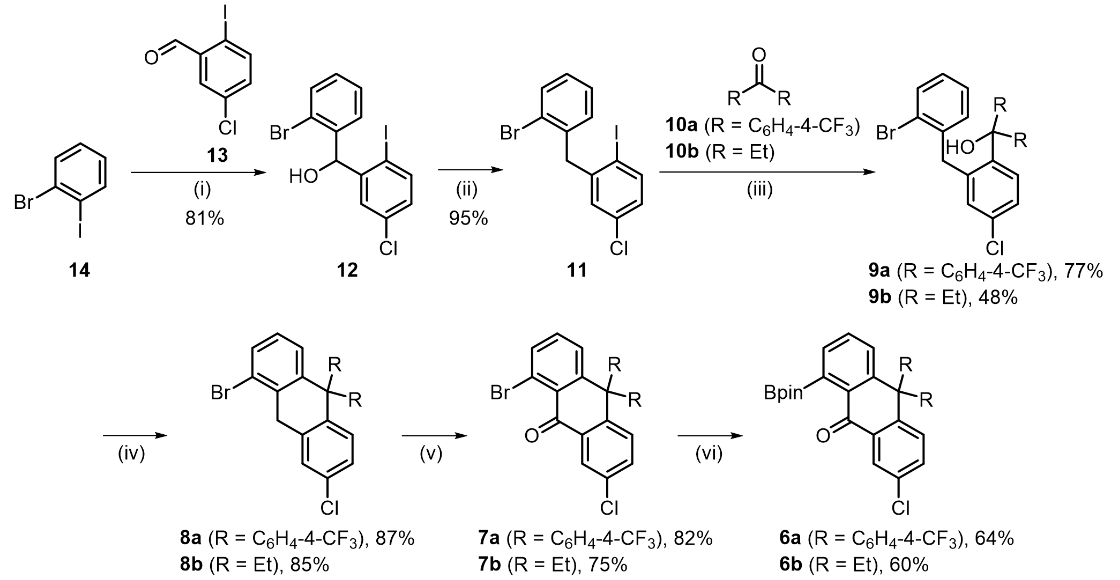

Reagents and conditions: (i) *i*-PrMgCl·LiCl, THF, -78 °C to -40 °C, 3 h, then 5-chloro-2-iodobenzaldehyde (**13**), THF, -40 °C to r.t., overnight; (ii) Et_3SiH, TfOH, DCM, r.t., 1 h; (iii) BuLi, THF, -78 °C, 2 h, then bis(4-(trifluoromethyl)phenyl)methanone (**10a**) or pentan-3-one (**10b**), THF, -78 °C to r.t., overnight; (iv) TfOH (10 mol%), DCM, 0 °C, 2 h; (v) CrO_3, 3,5-dimethylpyrazole, DCM, -10 °C, 3 h; (vi) $(Bpin)_2$, $PdCl_2(dppf)_2$, KOAc, DMF, 80 °C, overnight.

Fig. S2. Synthesis route of tricarbon annulated trifluorenocoronene (TCTFC) derivatives 1a and 1b.

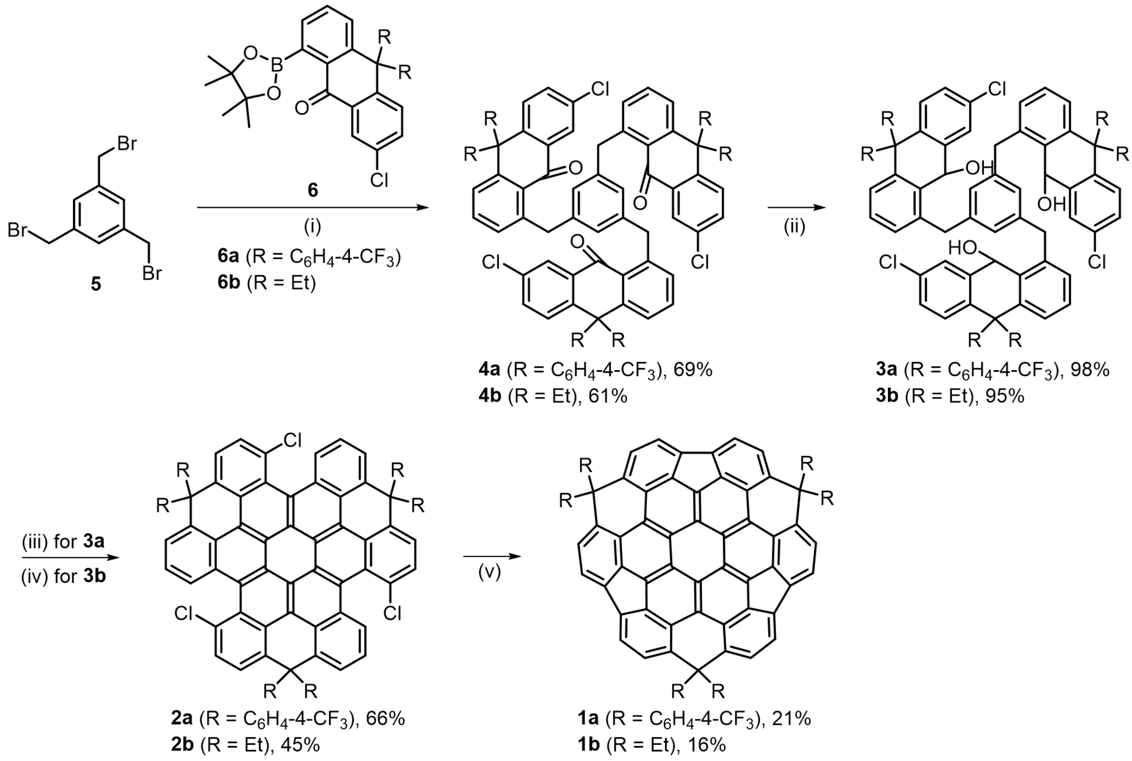

Reagents and conditions: (i) $Pd(PPh_3)_4$, Cs_2CO_3, toluene/H_2O, 80 °C, overnight; (ii) $NaBH_4$, THF/MeOH, r.t., 2 h; (iii) 1) $BF_3{\cdot}Et_2O$, DCM, r.t., 2 h; 2) DDQ, DCE, 80 °C, 30 min; 3) TfOH, 0 °C, 15 min; iv) 1) BBr_3, DCM, r.t., overnight; 2) DDQ, DCE, 80 °C, 30 min; 3) TfOH, 0 °C, 15 min; (v) $PdCl_2(PCy_3)_2$, DBU, DMAC, 145 °C, overnight.

Fig. S3. Synthesis route of reference compound tri-*tert*-butyl hexabenzocoronene (^{t}Bu$_3$-HBC).

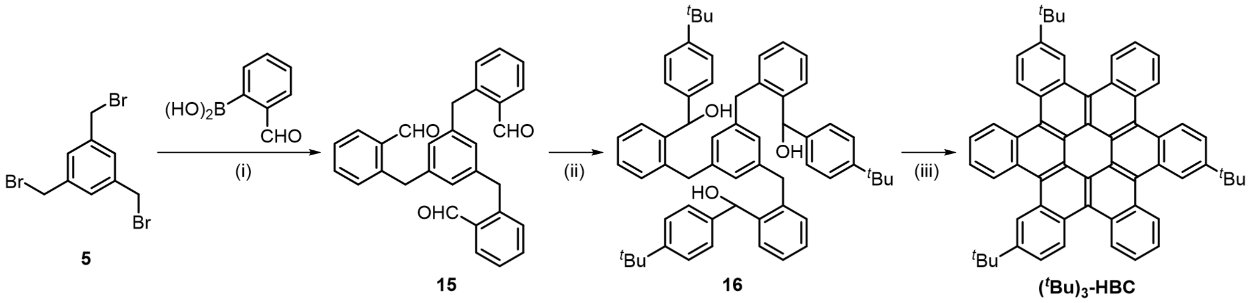

Reagents and conditions: (i) $Pd_3(PPh_3)_4$, Cs_2CO_3, toluene/EtOH/H_2O, 80 °C, overnight; (ii) 1) 4-*tert*-butylbromobenzene, Mg, I_2, THF, 80 °C, 2 h; 2) 1,3,5-tris(2-formylbenzyl)-benzene (**15**), r.t., 2 h; (iii) 1) $BF_3{\cdot}Et_2O$, DCM, r.t., 2h; 2) DDQ, 0 °C, 30 min.

Synthesis of (2-bromophenyl)(5-chloro-2-iodophenyl)methanol (**12**):

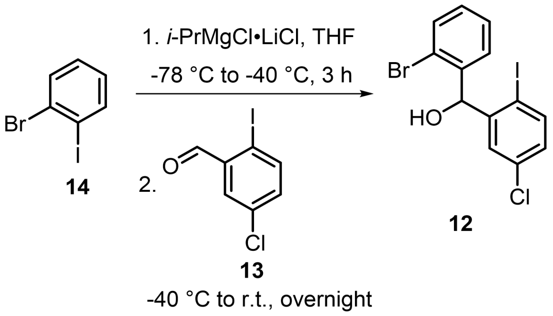

A flame-dried three-necked round-bottom flask equipped with a magnetic stir bar was charged with 1-bromo-2-iodobenzene (**14**) (20.00 g, 70.70 mmol) and anhydrous THF (150 mL) under an argon atmosphere. The solution was cooled to -78 °C, and *i*-PrMgCl·LiCl (1.3 M in THF, 60 mL, 78.00 mmol) was added dropwise via syringe. The reaction mixture was allowed to warm gradually to -40 °C over 3 h with continuous stirring. A solution of 5-chloro-2-iodobenzaldehyde (**13**) (12.56 g, 47.13 mmol) in anhydrous THF (100 mL) was then slowly added. After stirring at -40 °C for 30 min, the reaction was allowed to warm to ambient temperature and stirred overnight. The reaction was quenched by careful addition of aqueous HCl (1.0 M, 300 mL, 300 mmol), and the resulting mixture was extracted with ethyl acetate (2 × 300 mL). The combined organic extracts were washed with brine (150 mL), dried over anhydrous Na_2SO_4, filtered, and concentrated under reduced pressure. Purification by silica gel column chromatography (gradient elution: ethyl acetate/petroleum ether = 1/20 to 1/5, v/v) afforded **12** (16.21 g, 81% yield) as a white solid.

(2-bromophenyl)(5-chloro-2-iodophenyl)methanol (12)

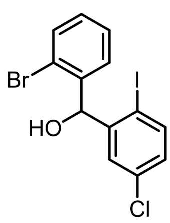

^{1}H NMR (600 MHz, $CDCl_3$): δ 7.76 (d, *J* = 8.4 Hz, 1H), 7.60 (d, *J* = 8.0 Hz, 1H), 7.36 (d, *J* = 2.2 Hz, 1H), 7.31 (t, *J* = 7.0 Hz, 1H), 7.21 (d, *J* = 7.2 Hz, 2H), 7.03 (dd, *J* = 8.3, 2.3 Hz, 1H), 6.18 (d, *J* = 3.0 Hz, 1H), 2.59 (ddd, *J* = 26.1, 12.3, 6.4 Hz, 1H); **^{13}C NMR** (150 MHz, $CDCl_3$): δ 144.6, 139.6, 139.2, 134.0, 132.1, 128.7, 128.7, 127.7, 126.8, 123.4, 95.4, 77.1; **HRMS** (ESI, negative mode, methanol/chloroform): *m/z* calcd for $C_{13}H_8BrClIO$ $(M-H)^-$: 420.8497, found: 420.8496.

Synthesis of 2-(2-bromobenzyl)-4-chloro-1-iodobenzene (**11**):

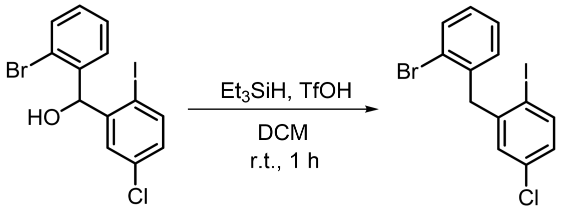

12 **11**

To a solution of (2-bromophenyl)(5-chloro-2-iodophenyl)methanol (**12**) (15.00 g, 35.42 mmol) and triethylsilane (17.00 mL, 106.26 mmol) in anhydrous CH_2Cl_2 (250 mL) at 0 °C under an argon atmosphere, trifluoromethanesulfonic acid (0.93 mL, 10.62 mmol) was added dropwise. The reaction mixture was allowed to warm to room temperature and stirred for 1 h, after which it was carefully poured into a cold saturated aqueous $NaHCO_3$ solution (300 mL). The organic layer was separated, dried over anhydrous Na_2SO_4, filtered, and concentrated under reduced pressure. Purification by silica gel column chromatography (eluent: petroleum ether) afforded **11** (13.71 g, 95%) as a white solid.

2-(2-bromobenzyl)-4-chloro-1-iodobenzene (11)

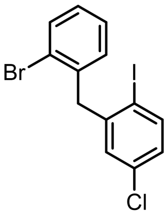

^{1}H NMR (600 MHz, $CDCl_3$): δ 7.79 (d, *J* = 8.4 Hz, 1H), 7.62 (d, *J* = 8.0 Hz, 1H), 7.26 (td, *J* = 7.5, 1.2 Hz, 1H), 7.15 (td, *J* = 7.8, 1.6 Hz, 1H), 6.98 (dd, *J* = 7.5, 0.7 Hz, 1H), 6.95 (dd, *J* = 8.4, 2.5 Hz, 1H), 6.90 (d, *J* = 2.0 Hz, 1H), 4.13 (s, 2H); **^{13}C NMR** (150 MHz, $CDCl_3$): δ 144.1, 140.5, 138.2, 134.8, 133.1, 130.8, 129.9, 128.5, 128.5, 127.8, 125.2, 98.5, 47.0; **HRMS** (APCI-TOF, negative mode, methanol/chloroform): *m/z* calcd for $C_{13}H_8BrClI$ $(M-H)^-$: 404.8537, found: 404.8541.

General procedure for the synthesis of tertiary alcohols **9a** and **9b**:

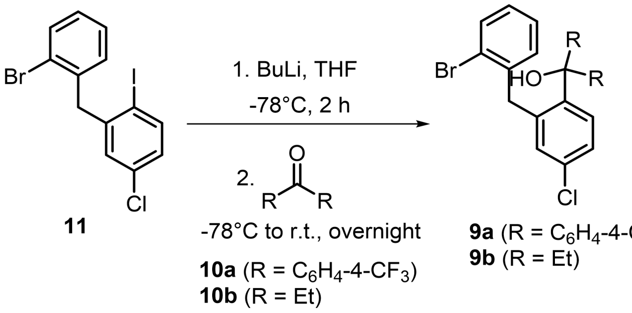

11

10a (R = C_6H_4-4-CF_3)
10b (R = Et)

9a (R = C_6H_4-4-CF_3)
9b (R = Et)

A flame-dried 100-mL Schlenk flask equipped with a magnetic stir bar was charged with 2-(2-bromobenzyl)-4-chloro-1-iodobenzene (**11**) (6.00 g, 14.72 mmol) and anhydrous

THF (15 mL) under an argon atmosphere. The solution was cooled to -78 °C, and *n*-BuLi (2.5 M in hexane, 5.90 mL, 14.72 mmol) was added dropwise via syringe. After stirring for 2 h at -78 °C, a solution of the appropriate ketone in anhydrous THF (20 mL) was slowly added. The reaction mixture was maintained at -78 °C for 30 min, then allowed to warm to ambient temperature and stirred overnight. The reaction was quenched with aqueous HCl (1.0 M, 50 mL, 50 mmol), and the mixture was extracted with ethyl acetate (2 × 200 mL). The combined organic extracts were washed with brine (100 mL), dried over anhydrous Na_2SO_4, filtered, and concentrated under reduced pressure. Purification by silica gel column chromatography (gradient elution: ethyl acetate/petroleum ether = 1/20 to 1/5, v/v) afforded **9a** or **9b**.

Synthesis of (2-(2-bromobenzyl)-4-chlorophenyl)bis(4-(trifluoromethyl)phenyl)methanol (**9a**):

Following the general procedure, using bis(4-(trifluoromethyl)phenyl)methanone (**10a**) (4.26 g, 13.40 mmol), **9a** was obtained as a white solid (6.21 g, 77% yield).

(2-(2-bromobenzyl)-4-chlorophenyl)bis(4-(trifluoromethyl)phenyl)methanol (9a)

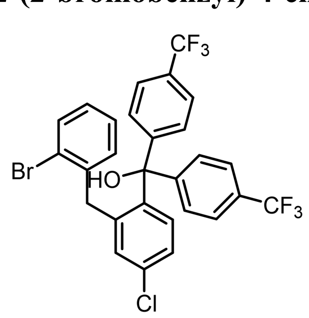

^{1}H NMR (600 MHz, $CDCl_3$): δ 7.60 (d, *J* = 8.3 Hz, 4H), 7.50 (dd, *J* = 7.9, 1.0 Hz, 1H), 7.41 (d, *J* = 8.2 Hz, 4H), 7.17 (td, *J* = 7.5, 1.1 Hz, 1H), 7.11 (dd, *J* = 8.5, 2.3 Hz, 1H), 7.07 (td, *J* = 7.8, 1.6 Hz, 1H), 6.93 (d, *J* = 2.2 Hz, 1H), 6.78 (dd, *J* = 7.6, 1.3 Hz, 1H), 6.72 (d, *J* = 8.5 Hz, 1H), 3.95 (s, 2H), 3.07 (s, 1H); **^{13}C NMR** (150 MHz, $CDCl_3$): δ 148.1, 140.6, 140.2, 138.2, 133.7, 131.9, 130.9, 130.0, 129.8, 129.0 (q, *J* = 32.6 Hz), 127.2, 126.9, 126.5, 124.9, 124.32 (q, *J* = 3.7 Hz), 123.8, 122.90 (q, *J* = 272.3 Hz), 81.29, 39.13; **HRMS** (APCI-TOF, negative mode, methanol/chloroform): *m/z* calcd for $C_{28}H_{18}BrCl_2F_6O$ $(M+Cl)^-$: 632.9828, found: 632.9832.

Synthesis of 3-(2-(2-bromobenzyl)-4-chlorophenyl)pentan-3-ol (**9b**):

Following the general procedure, using pentan-3-one (**10b**) (7.10 mL, 66.93 mmol), **9b** was obtained as a white solid (2.38 g, 48% yield).

3-(2-(2-bromobenzyl)-4-chlorophenyl)pentan-3-ol (9b)

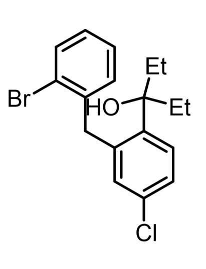

^{1}H NMR (600 MHz, $CDCl_3$): δ 7.59 (d, *J* = 7.9 Hz, 1H), 7.33 (d, *J* = 8.6 Hz, 1H), 7.22 – 7.18 (m, 2H), 7.11 – 7.07 (m, 1H), 6.92 (d, *J* = 2.2 Hz, 1H), 6.85 (d, *J* = 7.6 Hz, 1H), 4.37 (s, 2H), 1.99 (dq, *J* = 14.9, 7.5 Hz, 2H), 1.82 (dq, *J* = 14.7, 7.4 Hz, 2H), 1.57 (s, 1H), 0.77 (t, *J* = 7.4 Hz, 6H); **^{13}C NMR** (150 MHz, $CDCl_3$): δ 141.6, 141.0, 139.3, 132.8, 132.5, 132.0, 130.6, 129.5, 127.8, 127.5, 126.2, 124.8, 79.1, 40.7, 34.4, 8.2; **HRMS** (APCI-TOF, negative mode, methanol/chloroform): *m/z* calcd for $C_{18}H_{20}BrCl_2O$ $(M+Cl)^-$: 401.0080, found: 401.0077.

General procedure for the synthesis of dihydroanthracenes **8a** and **8b**:

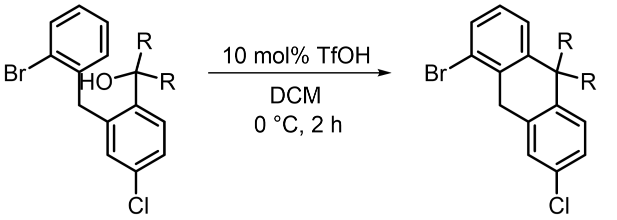

9a (R = C_6H_4-4-CF_3)
9b (R = Et)

8a (R = C_6H_4-4-CF_3)
8b (R = Et)

A solution of the appropriate tertiary alcohol (10.00 mmol) in anhydrous CH_2Cl_2 (50 mL) was cooled to 0 °C under an argon atmosphere. Trifluoromethanesulfonic acid (TfOH, 10 mol%) was added dropwise, and the reaction mixture was stirred at 0 °C for 2 h. The reaction was carefully quenched with saturated aqueous $NaHCO_3$ solution (100 mL). The mixture was extracted with dichloromethane (2 × 50 mL), and the combined organic layers were dried over anhydrous Na_2SO_4, filtered, and concentrated under reduced pressure. Purification by silica gel column chromatography (petroleum ether) afforded **8a** or **8b**.

Synthesis of 1-bromo-7-chloro-10,10-bis(4-(trifluoromethyl)phenyl)-9,10-dihydroanthracene (**8a**):

Following the general procedure, using tertiary alcohol **9a** (6.00 g, 10.00 mmol), **8a** was obtained as a white solid (5.08 g, 87% yield).

1-bromo-7-chloro-10,10-bis(4-(trifluoromethyl)phenyl)-9,10-dihydroanthracene (8a)

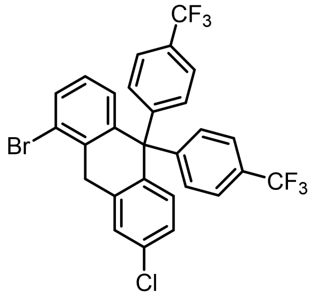

^{1}H NMR (600 MHz, $CDCl_3$): δ 7.57 (dd, J = 7.9, 0.9 Hz, 1H), 7.52 (d, J = 8.3 Hz, 4H), 7.41 (d, J = 2.1 Hz, 1H), 7.20 (dd, J = 8.4, 2.2 Hz, 1H), 7.08 (t, J = 7.9 Hz, 1H), 6.93 (d, J = 8.3 Hz, 4H), 6.77 (d, J = 7.8 Hz, 1H), 6.72 (d, J = 8.4 Hz, 1H), 3.79 (s, 2H); **^{13}C NMR** (150 MHz, $CDCl_3$: δ 146.8, 144.2, 140.4, 137.5, 135.1, 132.2, 130.5, 129.6, 129.6, 128.5 (q, J = 32.7 Hz), 127.7, 127.1, 126.3, 125.5, 124.1 (q, J = 3.7 Hz), 123.1, 122.9 (q, J = 272.1 Hz), 59.4, 34.6; **HRMS** (APCI-TOF, negative mode, methanol/chloroform): *m/z* $C_{28}H_{15}BrClF_6$ $(M-H)^-$: 578.9944, found: 578.9969.

Synthesis of 1-bromo-7-chloro-10,10-diethyl-9,10-dihydroanthracene (**8b**):

Following the general procedure, using tertiary alcohol **9b** (2.00 g, 5.44 mmol), **8b** was obtained as a white solid (1.62 g, 85% yield).

1-bromo-7-chloro-10,10-diethyl-9,10-dihydroanthracene (8b)

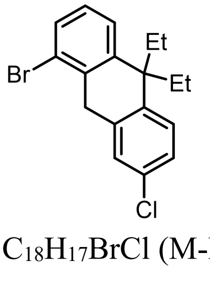

^{1}H NMR (600 MHz, $CDCl_3$): δ 7.48 (dd, J = 7.8, 0.7 Hz, 1H), 7.37 (d, J = 7.9 Hz, 1H), 7.31 (d, J = 8.5 Hz, 1H), 7.29 (d, J = 2.1 Hz, 1H), 7.24 (dd, J = 8.5, 2.2 Hz, 1H), 7.15 (t, J = 7.9 Hz, 1H), 4.06 (s, 2H), 2.05 – 2.00 (m, 4H), 0.40 (t, J = 7.3 Hz, 6H); **^{13}C NMR**: (150 MHz, $CDCl_3$) δ 140.8, 136.2, 135.7, 133.2, 130.4, 129.0, 126.9, 126.7, 126.5, 126.0, 124.3, 123.6, 47.2, 36.7, 33.7, 7.9; **HRMS** (APCI-TOF, negative mode, methanol/chloroform): m/z $C_{18}H_{17}BrCl$ $(M-H)^-$: 347.0197, found: 347.0220.

General procedure for the synthesis of anthracenones **7a** and **7b**:

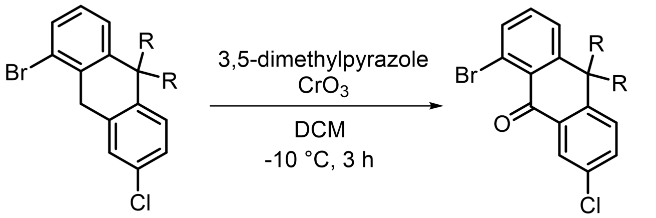

8a (R = C_6H_4-4-CF_3) **7a** (R = C_6H_4-4-CF_3)
8b (R = Et) **7b** (R = Et)

A 250-mL round-bottom flask equipped with a magnetic stir bar was charged with 3,5-dimethylpyrazole (7.44 g, 77.35 mmol) in CH_2Cl_2 (60 mL) and cooled to -15 °C. To the cooled solution was added CrO_3 (7.73 g, 77.35 mmol) portionwise, and the resulting mixture was stirred at -15 °C for 15 min. A solution of the appropriate dihydroanthracene (4.30 mmol) in CH_2Cl_2 (10 mL) was then added dropwise, and the dark reaction mixture was stirred at -10 °C for 3 h. Upon completion, the reaction mixture was diluted with dichloromethane (100 mL), washed with aqueous NaOH (1.0 M, 3 × 100 mL), dried over anhydrous Na_2SO_4, filtered, and concentrated under reduced pressure. Purification by silica gel column chromatography (gradient elution: dichloromethane/petroleum ether = 1/4 to 1/2, v/v) afforded **7a** or **7b**.

Synthesis of 1-bromo-7-chloro-10,10-bis(4-(trifluoromethyl)phenyl)anthracen-9(10H)-one (**7a**):

Following the general procedure, using dihydroanthracene **8a** (2.50 g, 4.30 mmol), **7a** was obtained as a pale-yellow solid (2.10 g, 82% yield).

1-bromo-7-chloro-10,10-bis(4-(trifluoromethyl)phenyl)anthracen-9(10H)-one (7a)

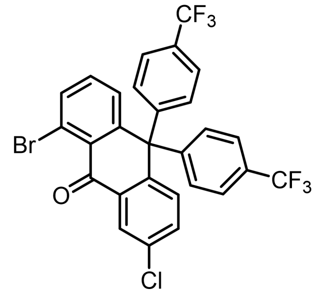

^{1}H NMR (600 MHz, $CDCl_3$): δ 8.11 (d, J = 2.3 Hz, 1H), 7.77 (dd, J = 7.9, 0.9 Hz, 1H), 7.54 (d, J = 8.4 Hz, 4H), 7.47 (dd, J = 8.4, 2.4 Hz, 1H), 7.31 (t, J = 8.0 Hz, 1H), 7.00 (d, J = 8.3 Hz, 4H), 6.97 (dd, J = 7.9, 0.9 Hz, 1H), 6.90 (d, J = 8.4 Hz, 1H); **^{13}C NMR** (150 MHz, $CDCl_3$): δ 181.9, 148.6, 147.5, 143.1, 134.7, 134.2, 133.8, 131.5, 131.4, 130.2, 129.9, 129.3, 129.0 (q, J = 32.8 Hz), 128.8 126.7, 124.5 (q, J = 3.7 Hz), 122.7 (q, J = 272.1 Hz), 121.5, 57.4; **HRMS** (APCI-TOF, positive mode, methanol/chloroform): *m/z* calcd for $C_{28}H_{15}BrClF_6O$ $(M+H)^+$: 594.9894, found: 594.9903.

Synthesis of 1-bromo-7-chloro-10,10-diethylanthracen-9(10H)-one (**7b**):

Following the general procedure, using dihydroanthracene **8b** (1.50 g, 4.30 mmol), **7b** was obtained as a pale-yellow solid (1.17 g, 75% yield).

1-bromo-7-chloro-10,10-diethylanthracen-9(10H)-one (7b)

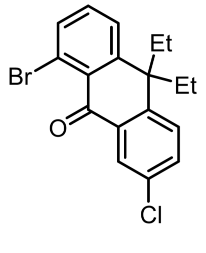

^{1}H NMR (600 MHz, $CDCl_3$): δ 8.28 (d, J = 2.4 Hz, 1H), 7.73 (dd, J = 7.8, 0.9 Hz, 1H), 7.59 (ddd, J = 8.4, 5.3, 1.6 Hz, 2H), 7.50 (d, J = 8.6 Hz, 1H), 7.43 (t, J = 7.9 Hz, 1H), 2.24 – 2.17 (m, 4H), 0.29 (t, J = 7.3 Hz, 6H); **^{13}C NMR** (150 MHz, $CDCl_3$): δ 182.1, 149.9, 143.4, 135.3, 134.5, 133.6, 133.4, 133.2, 130.8, 127.3, 127.2, 125.8, 122.5, 48.2, 38.3, 8.7; **HRMS** (APCI-

TOF, positive mode, methanol/chloroform): *m/z* calcd for $C_{18}H_{17}BrClO$ $(M+H)^+$: 363.0146, found: 363.0156.

General procedure for the synthesis of building blocks **6a** and **6b**:

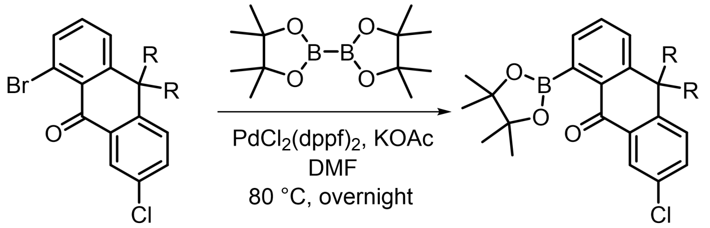

7a (R = C_6H_4-4-CF_3)
7b (R = Et)

6a (R = C_6H_4-4-CF_3)
6b (R = Et)

A 250-mL Schlenk flask equipped with a magnetic stir bar was charged with the appropriate brominated anthracenone (2.75 mmol), bis(pinacolato)diboron (1.40 g, 5.50 mmol), Pd(dppf)Cl_2 (0.20 g, 0.27 mmol), and KOAc (1.62 g, 16.5 mmol). The flask was evacuated and backfilled with argon three times before anhydrous, degassed DMF (60 mL) was added. The reaction mixture was stirred at 80 °C overnight under an argon atmosphere. After cooling to room temperature, the mixture was diluted with ethyl acetate (120 mL) and water (120 mL). The layers were separated, and the aqueous phase was extracted with ethyl acetate (2 × 50 mL). The combined organic extracts were washed with brine (120 mL), dried over anhydrous Na_2SO_4, filtered, and concentrated under reduced pressure. Purification by silica gel column chromatography (gradient elution: ethyl acetate/petroleum ether = 1/6 to 1/4, v/v) afforded **6a** or **6b**.

Synthesis of 7-chloro-1-(4,4,5,5-tetramethyl-1,3,2-dioxaborolan-2-yl)-10,10-bis(4-(trifluoromethyl)phenyl)-anthracen-9(10H)-one (**6a**):

Following the general procedure, using brominated anthracenone **7a** (1.64 g, 2.75 mmol), **6a** was obtained as a pale-yellow solid (1.14 g, 64% yield).

7-chloro-1-(4,4,5,5-tetramethyl-1,3,2-dioxaborolan-2-yl)-10,10-bis(4-(trifluoromethyl)phenyl)-anthracen-9(10H)-one (6a)

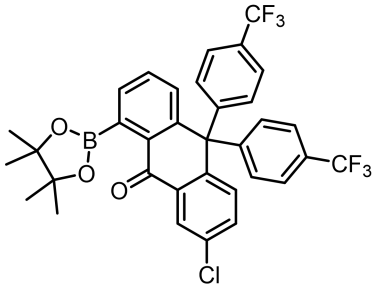

^{1}H NMR (600 MHz, $CDCl_3$): δ 8.30 (d, *J* = 2.4 Hz, 1H), 7.53 (s, 1H), 7.52 (d, *J* = 1.6 Hz, 1H), 7.50 (d, *J* = 8.5 Hz, 4H), 7.48 (dd, *J* = 8.6, 2.4 Hz, 1H), 7.14 (d, *J* = 8.3 Hz, 4H), 7.08 (dd, *J* = 8.7, 3.0 Hz, 2H), 1.51 (s, 12H); **^{13}C NMR** (150 MHz, $CDCl_3$: δ 182.7, 148.1, 145.7, 145.4, 133.6, 133.4, 132.5, 132.0, 131.4, 135.0, 130.7, 129.8, 129.2, 128.6 (q, *J* = 32.7 Hz), 126.7, 122.8 (q, *J* = 272.3 Hz), δ 124.4 (q, *J* = 3.5 Hz), 83.1, 56.9, 23.9; **HRMS** (APCI-TOF, positive mode, methanol/chloroform): *m/z* calcd for $C_{34}H_{27}BClF_6O_3$ $(M+H)^+$: 643.1640, found: 643.1654.

Synthesis of 7-chloro-10,10-diethyl-1-(4,4,5,5-tetramethyl-1,3,2-dioxaborolan-2-yl)anthracen-9(10H)-one (**6b**):

Following the general procedure, using brominated anthracenone **7b** (1.00 g, 2.75 mmol), **6b** was obtained as a pale-yellow solid (0.68 g, 60% yield).

7-chloro-10,10-diethyl-1-(4,4,5,5-tetramethyl-1,3,2-dioxaborolan-2-yl)anthracen-9(10H)-one (6b)

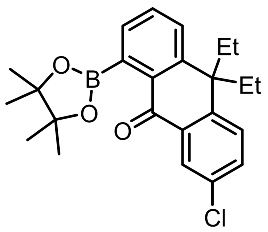

^{1}H NMR (600 MHz, $CDCl_3$): δ 8.33 (d, *J* = 2.4 Hz, 1H), 7.66 (dd, *J* = 7.9, 7.1 Hz, 1H), 7.61 (dd, *J* = 8.6, 2.4 Hz, 1H), 7.54 (t, *J* = 8.4 Hz, 2H), 7.48 (dd, *J* = 7.0, 0.8 Hz, 1H), 2.21 (ddt, *J* = 21.2, 13.8, 7.1 Hz, 4H), 1.52 (s, 12H), 0.20 (t, *J* = 7.3 Hz, 6H); **^{13}C NMR** (150 MHz, $CDCl_3$): δ 183.1, 145.1, 135.0, 133.0, 132.6, 132.5, 131.8, 129.3, 126.8, 126.2, 125.1, 82.7, 47.0, 37.0, 24.0, 7.7; **HRMS** (APCI-TOF, positive mode, methanol/chloroform): *m/z* calcd for $C_{24}H_{29}BClO_3$ $(M+H)^+$: 411.1893, found: 411.1908.

General procedure for the synthesis of dendrimer triketones **4a** and **4b**:

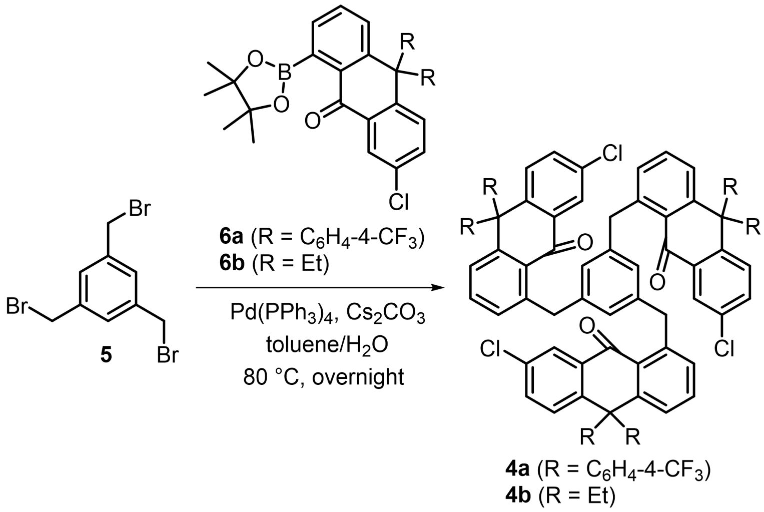

A 50-mL Schlenk flask was charged with 1,3,5-tris(bromomethyl)benzene (**5**) (0.15 g, 0.42 mmol), the appropriate anthracenone pinacol borate ester (1.51 mmol), $Pd(PPh_3)_4$ (0.15 g, 0.13 mmol), and Cs_2CO_3 (1.23 g, 3.78 mmol). After evacuating and backfilling with argon three times, degassed toluene (8 mL) and water (4 mL) were added. The reaction mixture was stirred at 80 °C overnight, then cooled to room temperature before the addition of ethyl acetate (50 mL) and water (50 mL). Following separation of the organic phase, the aqueous layer was extracted with ethyl acetate (2 × 20 mL). The combined organic layers were washed with brine (50 mL), dried over Na_2SO_4, filtered, and concentrated under reduced pressure. Purification by silica gel column chromatography (dichloromethane/petroleum ether = 1/4, v/v) afforded **4a** or **4b**.

Synthesis of 8,8',8''-(benzene-1,3,5-triyltris(methylene))tris(2-chloro-10,10-bis(4-(trifluoromethyl)phenyl)-anthracen-9(10H)-one) (**4a**):

Following the general procedure, using anthracenone pinacol borate ester **6a** (0.97 g, 1.51 mmol), **4a** was obtained as a pale-yellow solid (0.48 g, 69% yield).

8,8',8''-(benzene-1,3,5-triyltris(methylene))tris(2-chloro-10,10-bis(4-(trifluoromethyl)phenyl)-anthracen-9(10H)-one) (4a)

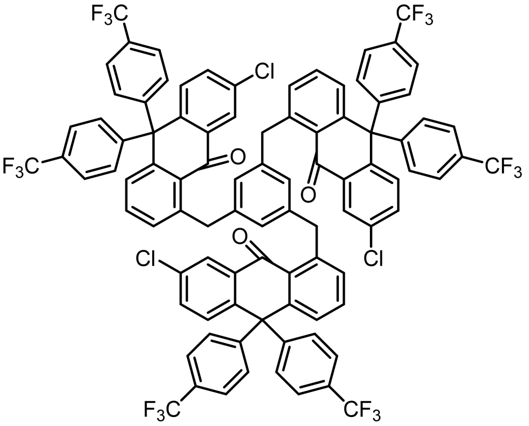

^{1}H NMR (600 MHz, $CDCl_3$): ^{1}H NMR (600 MHz, $CDCl_3$) δ 7.95 (d, J = 2.3 Hz, 3H), 7.49 (d, J = 8.4 Hz, 12H), 7.44 (dd, J = 8.4, 2.4 Hz, 3H), 7.30 (t, J = 7.8 Hz, 3H), 7.12 (d, J = 7.5 Hz, 3H), 7.01 (d, J = 8.3 Hz, 12H), 6.92 (d, J = 8.4 Hz, 3H), 6.87 (s, 3H), 6.83 (d, J = 7.8 Hz, 3H), 4.47 (s, 6H); **^{13}C NMR** (150 MHz, $CDCl_3$): δ 184.3, 148.2, 147.4, 143.6, 143.1, 139.5, 135.5, 133.4, 131.0, 131.0, 130.2, 129.9, 129.8, 129.3, 128.6 (q, J = 32.6 Hz), 127.8, 127.2, 126.3, 124.3 (q, J = 3.6 Hz), 122.8 (q, J = 272.1 Hz) 57.5, 39.0; **HRMS** (APCI-TOF, positive mode, methanol/chloroform): *m/z* calcd for $C_{93}H_{52}Cl_3F_{18}O_3$ $(M+H)^+$: 1663.2650, found: 1663.2687.

Synthesis of 8,8',8''-(benzene-1,3,5-triyltris(methylene))tris(2-chloro-10,10-diethylanthracen-9(10H)-one) (**4b**):

Following the general procedure, using anthracenone pinacol borate ester **6b** (0.62 g, 1.51 mmol), **4b** was obtained as a pale-yellow solid (0.25 g, 61% yield).

8,8',8''-(benzene-1,3,5-triyltris(methylene))tris(2-chloro-10,10-diethylanthracen-9(10H)-one) (4b)

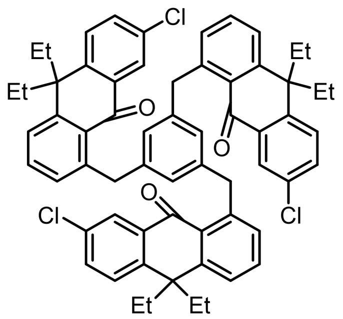

^{1}H NMR (600 MHz, $CDCl_3$): δ 8.18 (d, J = 2.4 Hz, 3H), 7.54 (dd, J = 8.5, 2.4 Hz, 3H), 7.46 (d, J = 8.6 Hz, 3H), 7.42 (d, J = 7.6 Hz, 3H), 7.38 (t, J = 7.7 Hz, 3H), 6.99 (d, J = 7.3 Hz, 3H), 6.80 (s, 3H), 4.60 (s, 2H), 2.25 – 2.07 (m, 4H), 0.21 (t, J = 7.3 Hz, 6H); **^{13}C NMR** (150 MHz, $CDCl_3$): δ 183.5, 147.1, 143.3, 142.9, 139.9, 135.3, 131.9, 131.7, 130.2, 129.1, 126.9, 126.3, 125.9, 123.3, 46.9, 40.3, 37.3, 7.6; **HRMS** (APCI-TOF, positive mode, methanol/chloroform): *m/z* calcd for $C_{63}H_{58}Cl_3O_3$ $(M+H)^+$: 967.3446, found: 967.3447.

General procedure for the synthesis of dendrimer triols **3a** and **3b**:

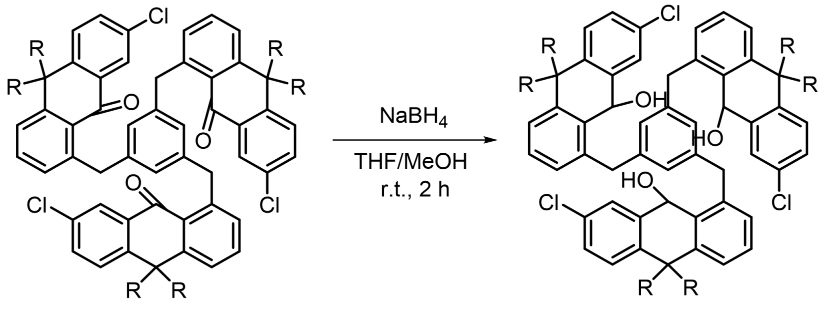

4a (R = C_6H_4-4-CF_3)
4b (R =Et)

3a (R = C_6H_4-4-CF_3)
3b (R =Et)

The dendrimer triketone (0.24 mmol) was dissolved in a mixture of methanol (10 mL) and THF (10 mL), followed by slow addition of $NaBH_4$ (0.61 g, 16.22 mmol) at 0 °C under an argon atmosphere. After stirring at room temperature for 2 h, the reaction was quenched

with H_2O (50 mL) at 0 °C, and the mixture was extracted with dichloromethane (2 × 50 mL). The combined organic layers were washed with brine (50 mL), dried over Na_2SO_4, filtered, and concentrated under reduced pressure. Purification by silica gel column chromatography (dichloromethane/petroleum ether = 1/2, v/v) afforded **3a** or **3b**.

Synthesis of 8,8',8''-(benzene-1,3,5-triyltris(methylene))tris(2-chloro-10,10-bis(4-(trifluoromethyl)phenyl)-9,10-dihydroanthracen-9-ol) (**3a**):

Following the general procedure, using dendrimer triketone **4a** (0.40 g, 0.24 mmol), **3a** was obtained as a white solid (0.39 g, 97% yield).

8,8',8''-(benzene-1,3,5-triyltris(methylene))tris(2-chloro-10,10-bis(4-(trifluoromethyl)phenyl)-9,10-dihydroanthracen-9-ol) (3a)

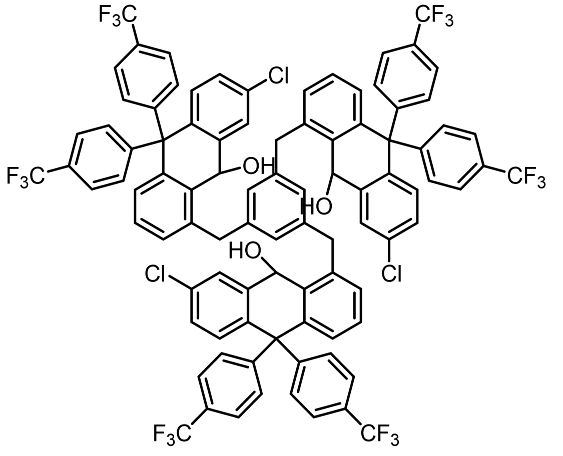

^{1}H NMR (600 MHz, $CDCl_3$): δ 7.51 (t, *J* = 8.7 Hz, 6H), 7.49 – 7.46 (m, 6H), 7.39 (dd, *J* = 14.3, 2.1 Hz, 3H), 7.29 – 7.26 (m, 3H), 7.17 (t, *J* = 7.7 Hz, 1H), 7.13 – 7.08 (m, 3H), 7.04 (dd, *J* = 11.1, 8.3 Hz, 9H), 6.95 – 6.91 (m, 9H), 6.80 (dd, *J* = 8.4, 4.6 Hz, 3H), 6.73 (dd, *J* = 11.6, 8.0 Hz, 3H), 5.76 – 5.73 (m, 2H), 4.30 (dd, *J* = 15.9, 6.0 Hz, 3H), 4.17 (d, *J* = 16.0 Hz, 3H), 1.00 (t, *J* = 8.1 Hz, 3H); **^{13}C NMR** (150 MHz, $CDCl_3$): δ 150.2, 150.2, 148.9, 148.8, 143.6, 143.5, 141.7, 140.2, 140.1, 138.9, 138.9, 138.48, 138.5, 134.6, 134.5, 132.6, 132.6, 130.8, 130.1, 128.9, 128.8, 128.7, 128.7, 128.6, 128.5 – 128.1 (m) 128.1, 127.2, 127.1, 127.1, 126.8, 126.6, 124.7 – 124.4 (m), 124.07 – 123.8 (m), 123.8 (q, *J* = 272.3 Hz), 122.8 (q, *J* = 272.3 Hz), 66.1, 57.9, 37.4, 37.3; **HRMS** (APCI-TOF, positive mode, methanol/chloroform): *m/z* calcd for $C_{93}H_{57}Cl_4F_{18}O_3$ $(M+Cl)^+$: 1706.2784, found: 1706.2791.

Synthesis of 8,8',8''-(benzene-1,3,5-triyltris(methylene))tris(2-chloro-10,10-diethyl-9,10-dihydroanthracen-9-ol) (**3b**):

Following the general procedure, using dendrimer triketone **4b** (0.23 g, 0.24 mmol), **3b** was obtained as a white solid (0.22 g, 95% yield).

8,8',8''-(benzene-1,3,5-triyltris(methylene))tris(2-chloro-10,10-diethyl-9,10-dihydroanthracen-9-ol) (3b)

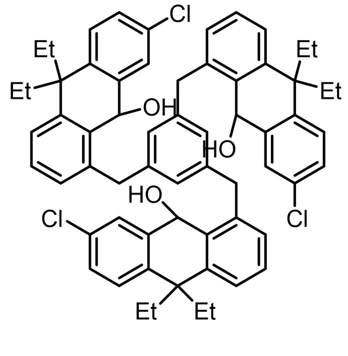

^{1}H NMR (600 MHz, $CDCl_3$): δ 7.43 (dd, *J* = 14.0, 1.7 Hz, 3H), 7.37 – 7.34 (m, 3H), 7.33 – 7.31 (m, 3H), 7.30 (d, *J* = 4.4 Hz, 3H), 7.21 (td, *J* = 7.6, 3.4 Hz, 3H), 6.95 (dd, *J* = 12.1, 7.2 Hz, 3H), 6.92 – 6.86 (m, 3H), 5.70 (dd, *J* = 7.7, 3.0 Hz, 3H), 4.38 (dd, *J* = 15.9, 6.7 Hz, 3H), 4.16 (q, *J* = 14.1, 13.3 Hz, 3H), 2.07 (dddt, *J* = 60.2, 31.6, 13.7, 7.1 Hz, 12H), 1.55 (dd, *J* = 7.8, 2.5 Hz, 3H), 0.45 (q, *J* = 6.6, 6.1 Hz, 9H), 0.14 (dt, *J* = 14.6, 7.2 Hz, 7H), 0.08 (t, *J* = 3.6 Hz, 2H); **^{13}C NMR** (150 MHz, $CDCl_3$): δ 141.5, 141.4, 140.2, 140.2, 139.9, 139.9, 139.8, 139.6, 138.3, 138.3, 138.2, 135.2, 135.2, 131.8, 131.8, 129.3, 128.9, 128.8, 128.8, 128.7, 128.7, 127.5, 127.4, 124.4, 124.3, 124.3, 65.3, 47.6, 38.9, 38.5, 38.4, 38.4, 36.8, 36.7,

36.6, 9.7, 8.6, 8.6; **HRMS** (APCI-TOF, negative mode, methanol/chloroform): *m/z* calcd for $C_{63}H_{63}Cl_4O_3$ $(M+Cl)^-$ 1009.3507, found: 1009.3510.

Synthesis of bowl precursors **2a**:

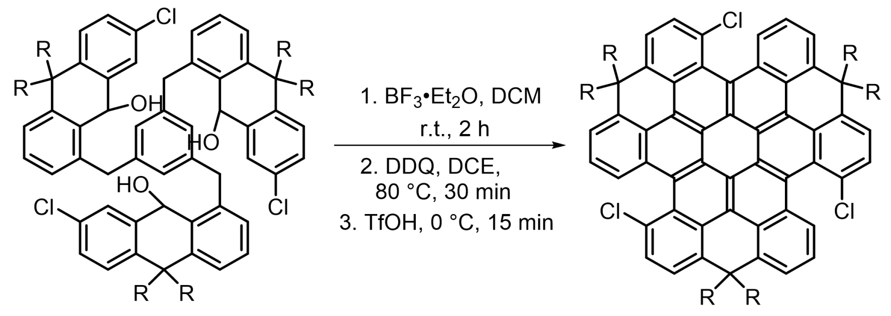

3a (R = C_6H_4-4-CF_3) **2a** (R = C_6H_4-4-CF_3)

A solution of dendrimer triol **3a** (0.24 g, 0.14 mmol) in dry CH_2Cl_2 (10 mL) was treated with $BF_3 \cdot Et_2O$ (0.15 mL), added dropwise at 0 °C under argon atmosphere. After stirring at room temperature for 2 h, the reaction was quenched with saturated $NaHCO_3$ solution (20 mL), and the mixture was extracted with dichloromethane (2 × 20 mL). The combined organic layers were dried over Na_2SO_4, filtered, and concentrated under reduced pressure. The residue was used directly in the next step.

The crude residue and DDQ (0.20 g, 0.89 mmol) were added to a 50-mL Schlenk flask, which was evacuated and backfilled with argon three times before the addition of dry 1,2-dichloroethane (20 mL). After heating the mixture at 80 °C for 30 min and cooling to 0 °C, TfOH (0.2 mL, 2.3 mmol) was added dropwise under an argon atmosphere. Following 15 min of stirring at 0 °C, the reaction was quenched by the addition of triethylamine (1.0 mL). After removal of volatiles under vacuum, methanol (30 mL) was added, and the resulting precipitate was collected by filtration and washed with methanol (20 mL). Further purification by silica gel column chromatography (dichloromethane/petroleum ether = 1/5, v/v) afforded precursor **2a** (152 mg, 66% yield) as a yellow solid.

3,10,17-trichloro-7,7,14,14,21,21-hexakis(4-(trifluoromethyl)phenyl)-14,21-dihydro-7*H*-triphenaleno[1,2,3,4,5-*fghij*:1',2',3',4',5'-*pqrst*:1'',2'',3'',4'',5''-*za*$_1$*b*$_1$*c*$_1$*d*$_1$]trinaphthylene (2a)

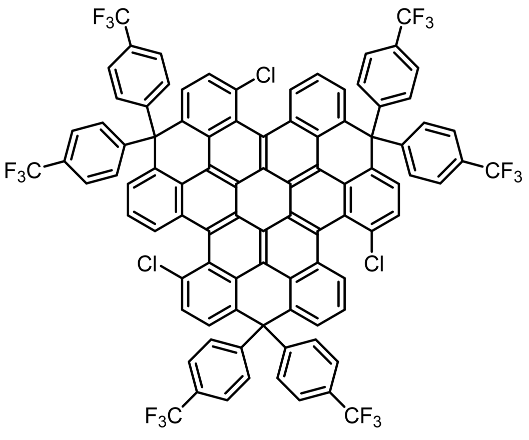

^{1}H NMR (600 MHz, $CDCl_3$): δ 8.65 (d, *J* = 8.2 Hz, 1H), 8.59 (t, *J* = 7.2 Hz, 2H), 7.92 – 7.83 (m, 4H), 7.78 (t, *J* = 9.9 Hz, 4H), 7.73 (d, *J* = 8.1 Hz, 2H), 7.70 (d, *J* = 8.0 Hz, 2H), 7.66 (d, *J* = 7.8 Hz, 2H), 7.62 (d, *J* = 7.7 Hz, 1H), 7.56 (d, *J* = 7.8 Hz, 1H), 7.48 (d, *J* = 8.1 Hz, 2H), 7.46 (d, *J* = 8.4 Hz, 3H), 7.42 (d, *J* = 8.3 Hz, 2H), 7.37 (d, *J* = 9.2 Hz, 2H), 7.36 (d, *J* = 8.8 Hz, 1H), 7.33 (d, *J* = 7.2 Hz, 1H), 7.30 (d, *J* = 7.1 Hz, 1H), 7.22 (t, *J* = 8.5 Hz, 4H), 7.18 (d, *J* = 8.3 Hz, 2H), 6.91 (d, *J* = 8.3 Hz, 2H); **HRMS** (APCI-TOF, positive mode, methanol/chloroform): *m/z* calcd for $C_{93}H_{40}Cl_3F_{18}$ $(M+H)^+$: 1603.1908, found: 1603.1902. Due to the limited solubility of compound **8a**, well-resolved ^{13}C NMR spectra can not be obtained.

Synthesis of bowl precursors **2b**:

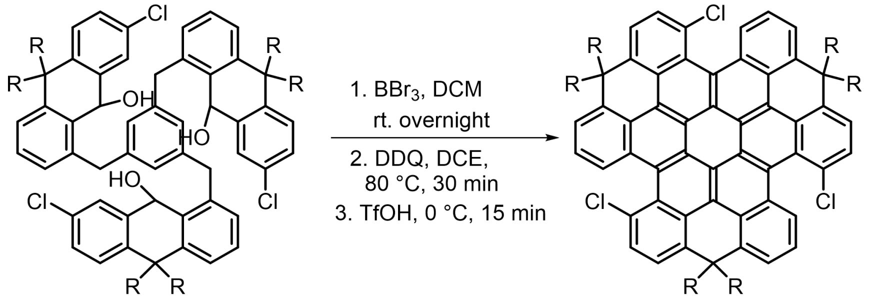

3b (R = Et) **2b** (R = Et)

A solution of dendrimer triol **3b** (0.20 g, 0.21 mmol) in dry CH_2Cl_2 (10 mL) was treated with BBr_3 solution in CH_2Cl_2 (1.0 M, 2.05 mL), added dropwise at 0 °C under argon atmosphere. After stirring at room temperature overnight, the reaction was quenched with saturated $NaHCO_3$ solution (20 mL), and the mixture was extracted with dichloromethane (2 × 20 mL). The combined organic layers were dried over Na_2SO_4, filtered, and concentrated under reduced pressure. The residue was used directly in the next step.

The crude residue and DDQ (0.29 g, 1.27 mmol) were added to a 50-mL Schlenk flask, which was evacuated and backfilled with argon three times before the addition of dry 1,2-dichloroethane (20 mL). After heating the mixture at 80 °C for 30 min and cooling to 0 °C, TfOH (0.2 mL, 2.3 mmol) was added dropwise under an argon atmosphere. Following 15 min of stirring at 0 °C, the reaction was quenched by the addition of triethylamine (1.0 mL). After removal of volatiles under vacuum, methanol (30 mL) was added, and the resulting precipitate was collected by filtration and washed sequentially with MeOH (20 mL), saturated $NaHCO_3$ solution (20 mL), water (20 mL), and methanol (20 mL). Further purification by recrystallization from chloroform afforded precursor **2b** (84 mg, 45% yield) as a yellow solid.

3,10,17-trichloro-7,7,14,14,21,21-hexaethyl-14,21-dihydro-7*H*-triphenaleno[1,2,3,4,5-*fgh ij*:1',2',3',4',5'-*pqrst*:1'',2'',3'',4'',5''-*za*$_1$*b*$_1$*c*$_1$*d*$_1$]trinaphthylene (2b)

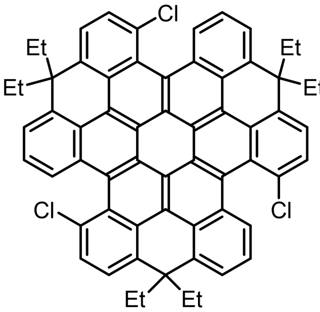

HRMS (APCI-TOF, positive mode, methanol/chloroform) *m/z* calcd for $C_{63}H_{46}Cl_3$ $(M+H)^+$: 907.2660, found: 907.2657. Due to the poor solubility of compound **2b**, a well-resolved ^1H NMR and ^{13}C NMR spectrum can not be obtained.

General procedure for the synthesis of tricarbon annulated trifluorenocoronene (TCTFC) derivatives **1a** and **1b**:

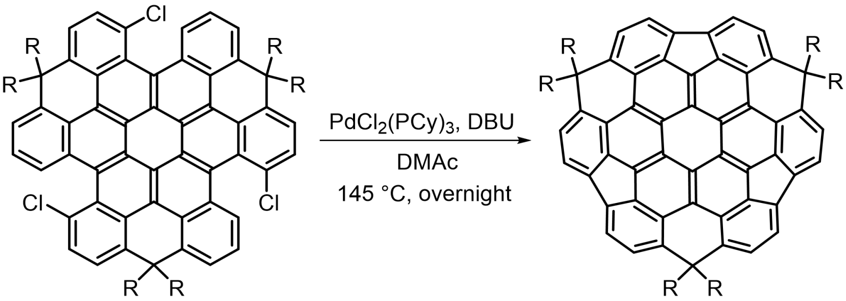

2a (R = C_6H_4-4-CF_3)
2b (R = Et)

1a (R = C_6H_4-4-CF_3)
1b (R = Et)

A Schlenk flask was charged with the appropriate precursor and $PdCl_2(PCy_3)_2$ (1.0 equiv), followed by the addition of 1,8-diazabicyclo[5.4.0]undec-7-ene (DBU) (1 mL) and *N*,*N* -dimethylacetamide (DMAc, 5 mL). The resulting mixture was subjected to three freeze-pump-thaw cycles and stirred at 145 °C overnight. After cooling to room temperature, the reaction mixture was diluted with dichloromethane (20 mL), washed with 1.0 M HCl solution (3 × 20 mL), dried over Na_2SO_4, filtered, and concentrated under reduced pressure. Purification as described below afforded **1a** or **1b**.

Synthesis of TCTFC derivative (**1a**):

Following the general procedure, using precursor **2a** (50 mg, 31 μmol) and $PdCl_2(PCy_3)_2$ (23 mg, 31 μmol). Purification by multiple preparative thin-layer chromatography (PTLC) (dichloromethane/*n*-hexane = 1/5, v/v) afforded **1a** as a yellow solid (10 mg, 21% yield).

TCTFC derivative 1a

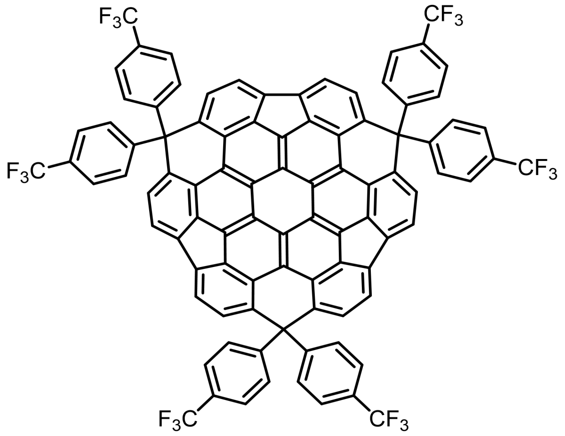

^{1}H NMR (600 MHz, CD_2Cl_2): δ 7.89 (d, *J* = 8.5 Hz, 6H), 7.87 (d, *J* = 7.4 Hz, 6H), 7.74 (d, *J* = 8.6 Hz, 6H), 7.67 (d, *J* = 7.1 Hz, 3H), 7.28 (d, *J* = 7.2 Hz, 3H), 7.22 (d, *J* = 6.9 Hz, 3H), 7.17 (d, *J* = 7.4 Hz, 6H), 6.63 (d, *J* = 7.3 Hz, 3H); **^{13}C NMR** (150 MHz, $CDCl_3$): δ 149.4, 147.5, 145.2, 137.4, 136.1, 135.6, 131.1, 130.6, 129.8, 129.6, 129.5, 129.4, 128.7 – 127.7 (m), 127.1, 124.6 – 124.3 (m), 123.8 – 123.6 (m), δ 123.0 (q, *J* = 272.5 Hz), 123.0 (q, *J* = 272.3 Hz), 59.4; **^{19}F NMR** (375 MHz, CD_2Cl_2): δ -62.78, -62.86; **HRMS** (APCI-TOF, positive mode, methanol/chloroform): *m/z* calcd for $C_{93}H_{37}F_{18}$ $(M+H)^+$: 1495.2608, found: 1495.2604; **HRMS** (MALDI-TOF, positive mode, chloroform/DCTB): *m/z* calcd for $C_{93}H_{36}F_{18}$ $(M)^+$: 1494.25241, found: 1494.25169.

Synthesis of TCTFC derivative (**1b**):

Following the general procedure, using precursor **2b** (50 mg, 55 μmol) and $PdCl_2(PCy_3)_2$ (41 mg, 55 μmol). Purification by multiple recrystallizations from chloroform afforded **1b** as a yellow solid (7 mg, 16% yield).

TCTFC derivative 1b

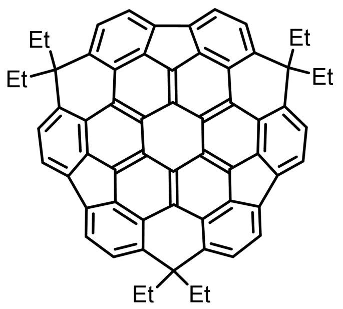

^{1}H NMR (600 MHz, 353 K, $C_2D_2Cl_4$): δ 8.01 (d, *J* = 7.3 Hz, 6H), 7.70 (d, *J* = 7.5 Hz, 6H), 2.53 (dq, *J* = 15.1, 7.2 Hz, 12H), 1.28 (t, *J* = 7.4 Hz, 9H), -0.00 (t, *J* = 7.1 Hz, 9H); **^{13}C NMR** (151 MHz, $CDCl_3$): δ 143.9, 136.2, 135.6, 134.9, 130.9, 128.0, 127.9, 125.1, 122.1, 48.9, 42.1, 27.9, 8.8, 8.3; **HRMS** (APCI-TOF, positive mode, methanol/chloroform): *m/z* calcd for $C_{63}H_{43}$ $(M+H)^+$: 799.3359, found: 799.3357; **HRMS** (MALDI-TOF, positive mode, chloroform/DCTB): *m/z* calcd for $C_{63}H_{42}$ $(M)^+$: 798.32810, found: 798.32776.

Synthesis of 1,3,5-tris(2-formylbenzyl)-benzene (**15**):

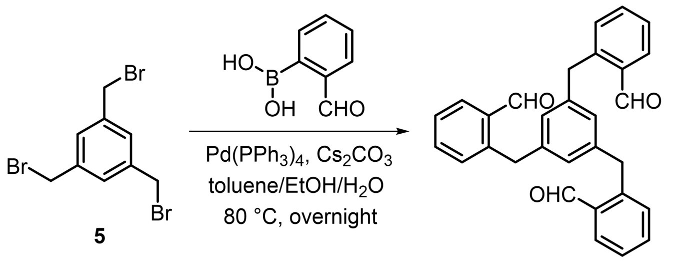

A 50-mL Schlenk flask was charged with 1,3,5-tris(bromomethyl)benzene (**5**) (0.50 g, 1.40 mmol), (2-formylphenyl)boronic acid (0.76 g, 5.10 mmol), $Pd(PPh_3)_4$ (0.24 g, 0.21 mmol), and Cs_2CO_3 (4.11 g, 12.61 mmol). After evacuating and backfilling with argon three times, degassed toluene (18 mL), ethanol (6 mL), and water (6 mL) were added. The mixture was stirred at 80 °C overnight, then cooled to room temperature before the addition of ethyl acetate (50 mL) and water (50 mL). Following separation of the organic phase, the aqueous layer was extracted with ethyl acetate (2 × 20 mL). The combined organic layers were washed with brine (50 mL), dried over Na_2SO_4, filtered, and concentrated under reduced pressure. Purification by silica gel column chromatography (dichloromethane/petroleum ether = 1/2, v/v) afforded **15** (0.51 g, 84% yield) as a white solid.

1,3,5-tris(2-formylbenzyl)-benzene (15)

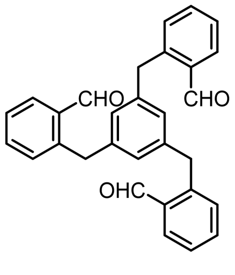

^{1}H NMR (600 MHz, $CDCl_3$) δ 10.16 (s, 3H), 7.82 (d, *J* = 7.6 Hz, 3H), 7.49 (t, *J* = 7.5 Hz, 3H), 7.39 (t, *J* = 7.5 Hz, 3H), 7.15 (d, *J* = 7.6 Hz, 3H), 6.73 (s, 3H), 4.32 (s, 6H); **^{13}C NMR** (151 MHz, $CDCl_3$) δ 191.3, 141.7, 139.8, 132.9, 132.8, 131.0, 130.4, 126.3, 125.9, 36.8;

Synthesis of dendrimer triol (**16**):

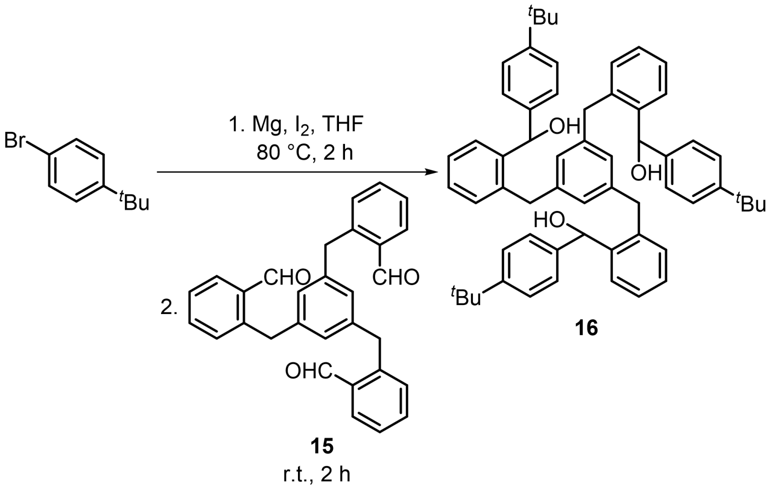

A solution of I_2 (70 mg, 0.28 mmol) and 1-bromo-4-(tert-butyl)benzene (1.30 g, 6.10 mmol) in dry THF (5 mL) was added dropwise to a stirred suspension of magnesium turnings (135 mg, 5.55 mmol) in dry THF (5 mL) under an argon atmosphere. The reaction mixture was stirred at 80 °C for 2 h until nearly complete consumption of the magnesium turnings. After Grignard reagent formation, the reaction mixture was cooled to 0 °C, and a solution of 2,2',2''-(benzene-1,3,5-triyltris(methylene))tribenzaldehyde (**15**) (0.40 g, 0.92 mmol) in dry THF (10 mL) was added slowly. The resulting mixture was warmed to room temperature and stirred for an additional 2 h. The reaction was quenched with saturated NH_4Cl solution (100 mL), and the mixture was extracted with ethyl acetate (2 × 50 mL). The combined organic layers were washed with brine (100 mL), dried over anhydrous Na_2SO_4, filtered, and concentrated under reduced pressure. Purification by silica gel column chromatography (dichloromethane/petroleum ether = 1/2, v/v) afforded **16** (0.73 g, 95% yield) as a white solid.

((benzene-1,3,5-triyltris(methylene))tris(benzene-2,1-diyl))tris((4-(tert-butyl)phenyl)methanol) (16)

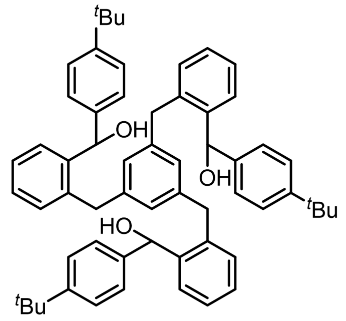

^{1}H NMR (600 MHz, $CDCl_3$) δ 7.57 (d, *J* = 7.7 Hz, 2H), 7.49 (d, *J* = 7.7 Hz, 1H), 7.33 – 7.26 (m, 6H), 7.26 – 7.20 (m, 6H), 7.12 (d, *J* = 7.5 Hz, 2H), 7.08 (t, *J* = 8.1 Hz, 3H), 7.02 (d, *J* = 8.0 Hz, 4H), 6.72 (s, 1H), 6.66 (s, 2H), 5.78 (s, 1H), 5.75 (s, 2H), 3.90 (d, *J* = 16.0 Hz, 1H), 3.82 (d, *J* = 9.0 Hz, 5H), 2.55 (s, 2H), 2.30 (s, 1H), 1.29 (s, 27H); **^{13}C NMR** (151 MHz, $CDCl_3$) δ 149.3, 140.9, 140.3, 140.2, 138.9, 136.5, 129.8, 129.8, 126.5, 126.5, 126.4, 126.2, 125.9, 125.9, 125.8, 125.8, 125.5, 124.3, 71.2, 70.9, 38.0, 37.9, 33.4, 30.3; **HRMS** (APCI-TOF, negative mode, methanol/chloroform): *m/z* calcd for $C_{60}H_{66}ClO_3$ $(M+Cl)^-$: 869.4706, found: 869.4701.

Synthesis route of reference compound tri-*tert*-butyl hexabenzocoronene (^{t}Bu$_3$-HBC):

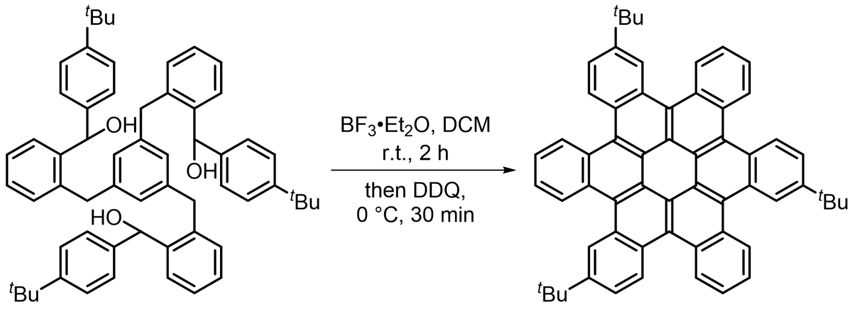

16 **^{t}Bu$_3$-HBC**

A solution of dendrimer triol **16** (0.70 g, 0.84 mmol) in dry CH_2Cl_2 (20 mL) was treated with $BF_3 \cdot Et_2O$ (1.5 mL) added dropwise at 0 °C under argon atmosphere. After stirring at room temperature for 2 h, the reaction mixture was diluted with dichloromethane (30 mL), cooled to 0 °C, and treated with DDQ (1.16 g, 5.11 mmol) under an argon atmosphere. After stirring at 0 °C for 30 min, saturated $NaHCO_3$ solution (50 mL) was added to quench the reaction. The mixture was extracted with dichloromethane (2 × 50 mL), and the combined organic layers were washed with saturated $NaHCO_3$ solution (2 × 100 mL), dried over anhydrous Na_2SO_4, filtered, and concentrated under reduced pressure. Purification by silica gel column chromatography (dichloromethane/petroleum ether = 1/6, v/v) afforded ^{t}Bu$_3$-HBC (0.53 g, 82% yield) as a yellow solid.

2,10,18-tri-tert-butyltrinaphtho[1,2,3,4-*fgh*:1',2',3',4'-*pqr*:1'',2'',3'',4''-*za$_1$b$_1$*]trinaphthylene (tri-*tert*-butylhexabenzocoronene, ^{t}Bu$_3$-HBC)

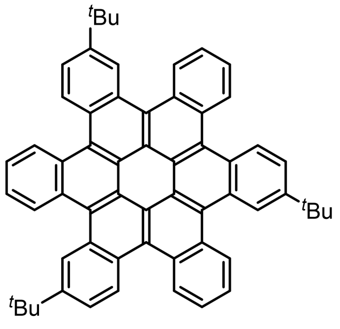

^{1}H NMR (600 MHz, $CDCl_3$): δ 9.32 – 9.26 (m, 9H), 9.22 (d, J = 8.6 Hz, 3H), 7.91 (dd, J = 8.6, 2.0 Hz, 3H), 7.83 (qd, J = 6.7, 3.5 Hz, 6H), 1.60 (s, 27H); **^{13}C NMR** (150 MHz, $CDCl_3$) : δ 147.9, 129.0, 128.9, 128.6, 128.0, 127.7, 127.7, 126.8, 125.1, 125.0, 124.9, 124.7, 124.0, 123.3, 120.5, 120.1, 76.2, 76.0, 75.7, 34.2, 30.5; **HRMS** (APCI-TOF, positive mode, methanol/chloroform): *m/z* calcd for $C_{60}H_{49}(M+H)^+$ 769.3829, found: 769.3827.

2. Crystallographic details

The X-ray diffraction data were collected on a Bruker D8 Venture X-ray single crystal diffractometer using Cu radiation at 150–237 K. Absorption correction was carried out by a multi-scan method. The structure was solved by direct methods with the SHELXT (*77*) program and refined by least-square methods with the SHELXL (*78*) program contained in the Olex2 suite (*79*). The crystallographic data reported in this work have been collected at Cambridge Crystallographic Data Centre (CCDC), under collecting numbers, CCDC 2444403 (for **2b**), 2444404 (for **1b**), 2444405 (for **1a**), 2444406 (for ^{t}Bu$_3$-HBC), 2444407 (for **1a@C$_{60}$**), and 2444408 (for **(1b)$_2$@C$_{60}$**). All data of those six crystalline structures can be obtained free of charge from CCDC via https://www.ccdc.cam.ac.uk/structures/.

2.1 X-ray crystallography for 2b

Single crystals of compound **2b** suitable for X-ray structural determination were obtained as yellow needles by slowly diffusing methanol vapor into its solution in chloroform.

Table S1. Crystallographic data and structure refinement details for 2b (CCDC: 2444403).

Empirical formula	$C_{63}H_{45}Cl_3$
Formula weight	908.34
Temperature/K	182.00
Crystal system	trigonal
Space group	R-3
a/Å	23.7020(5)
b/Å	23.7020(5)
c/Å	17.0199(5)
α/°	90
β/°	90
γ/°	120
Volume/Å^3	8280.5(4)
Z	6
ρ_{calc}g/cm^3	1.093
μ/mm^{-1}	1.769
F(000)	2844.0
Crystal size/mm^3	0.2 × 0.16 × 0.16
Radiation	CuKα (λ = 1.54178)
2Θ range for data collection/°	12.538 to 133.162
Index ranges	$-28 \le h \le 28$, $-20 \le k \le 28$, $-20 \le l \le 20$
Reflections collected	16672
Independent reflections	3227 [R_{int} = 0.0325, R_{sigma} = 0.0239]
Data/restraints/parameters	3227/0/199
Goodness-of-fit on F^2	1.137
Final R indexes [I>=2σ (I)]	R_1 = 0.0514, wR_2 = 0.1559
Final R indexes [all data]	R_1 = 0.0539, wR_2 = 0.1582
Largest diff. peak/hole / e Å^{-3}	0.28/-0.24

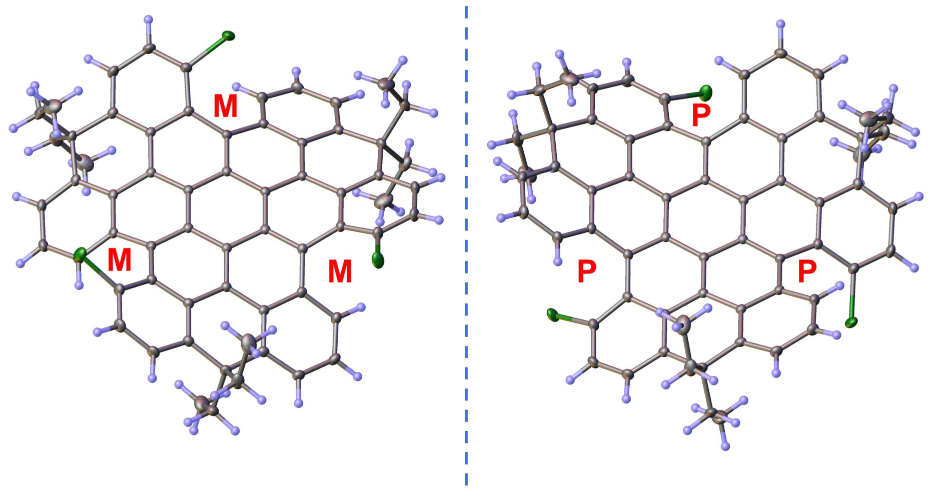

Fig. S4. Crystal structure of 2b. Top view of enantiomer pair of **2b**, MMM-isomer (left) and PPP-isomer (right). Thermal ellipsoids are shown at 30% probability.

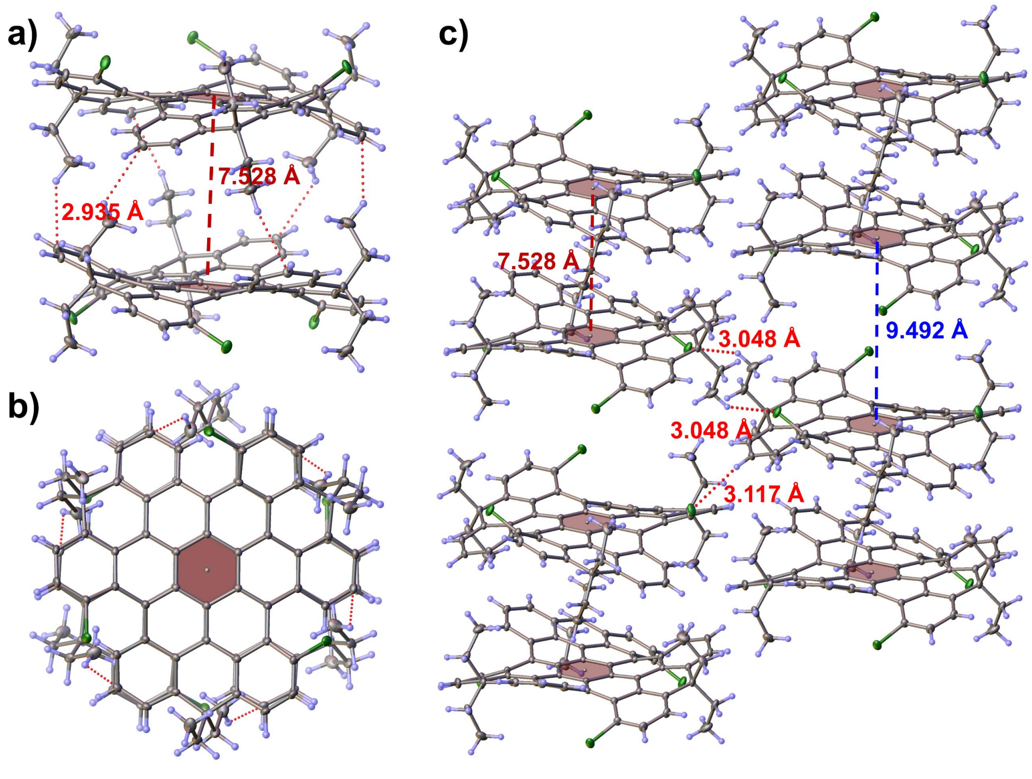

Fig. S5. Packing structure of 2b. (a and b) Side and top views of the staggered dimer stabilized by six C–H···C contacts. (c) Segregated dimer packing with intercolumnar C–H···Cl interactions.

2.2 X-ray crystallography for 1a

Single crystals of compound **1a** suitable for X-ray structural determination were obtained as orange needles by slowly diffusing methanol vapor into its solution in chloroform.

Table S2. Crystallographic data and structure refinement details for 1a (CCDC: 2444405).

Empirical formula	$C_{93}H_{36}F_{18}\cdot(CHCl_3)_4$
Formula weight	1972.69
Temperature/K	153.00
Crystal system	monoclinic
Space group	$P2_1/c$
a/Å	25.5432(7)
b/Å	17.3571(4)
c/Å	19.8526(5)
α/°	90
β/°	100.944(2)
γ/°	90
Volume/Å^3	8641.7(4)
Z	4
ρ_{calc}g/cm^3	1.516
μ/mm^{-1}	4.286
F(000)	3952.0
Crystal size/mm^3	0.5 × 0.4 × 0.3
Radiation	CuKα (λ = 1.54178)
2Θ range for data collection/°	6.192 to 136.976
Index ranges	$-30 \leq h \leq 30$, $-20 \leq k \leq 20$, $-23 \leq l \leq 23$
Reflections collected	142561
Independent reflections	15815 [R_{int} = 0.0658, R_{sigma} = 0.0309]
Data/restraints/parameters	15815/964/1362
Goodness-of-fit on F^2	1.040
Final R indexes [I>=2σ (I)]	R_1 = 0.0837, wR_2 = 0.2421
Final R indexes [all data]	R_1 = 0.0932, wR_2 = 0.2512
Largest diff. peak/hole / e Å^{-3}	1.31/-0.66

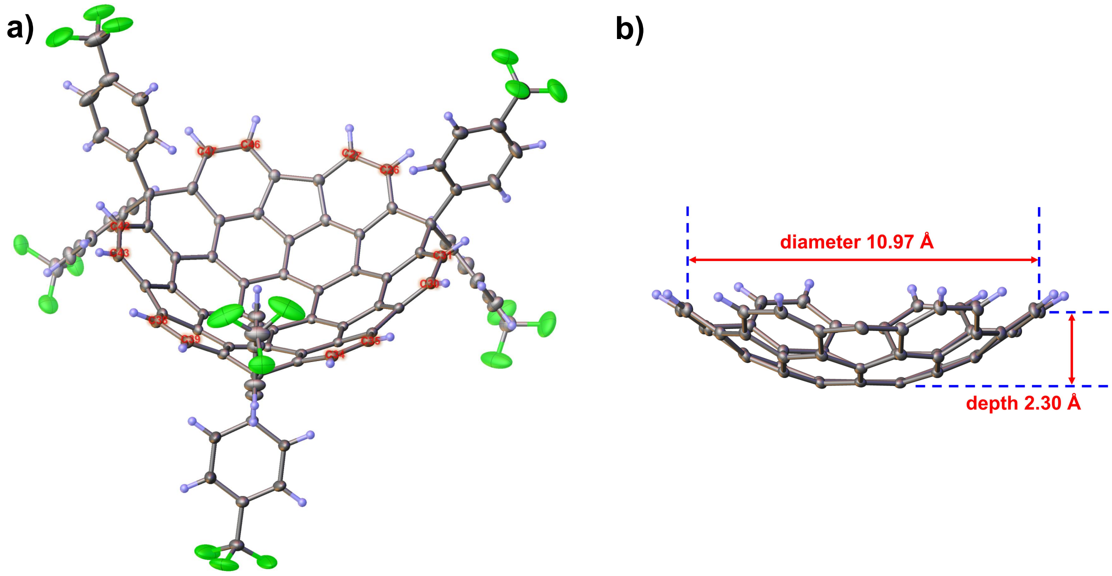

Fig. S6. Crystal structure of 1a. (a) Perspective view. (b) Side view showing diameter and depth. Solvent molecules and *p*-trifluoromethylphenyl groups omitted for clarity. Thermal ellipsoids at 30% probability. Diameter: average distance between opposite vertices (C26–C38, C27–C39, C30–C42, C31–C43, C34–C46, C35–C47). Depth: perpendicular distance from rim carbon mean plane to hub ring centroid.

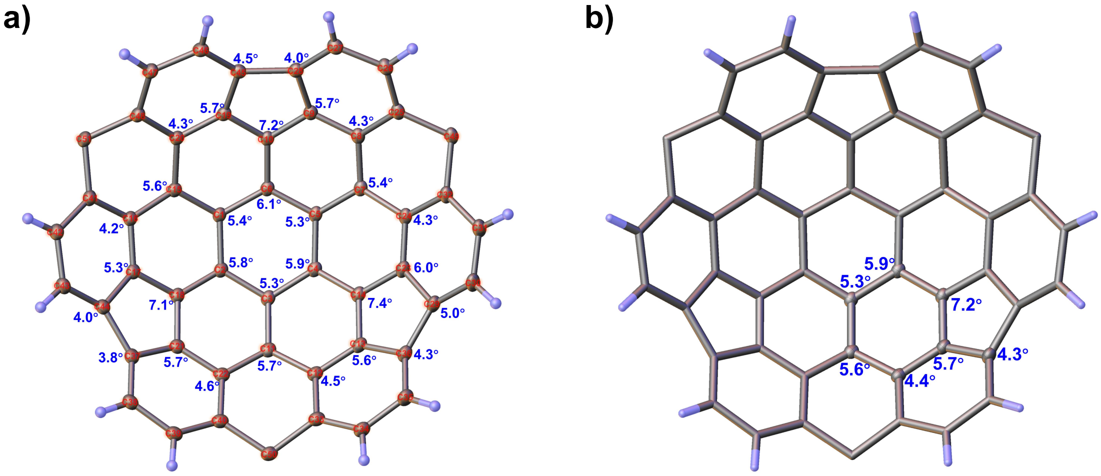

Fig. S7. POAV angles (blue) of 1a from X-ray structure. (a) Individual angles for each carbon atom. (b) Symmetry-averaged angles. The *p*-trifluoromethylphenyl groups are omitted for clarity. POAV angles calculated from three sigma bond angles at each conjugated carbon (*57*).

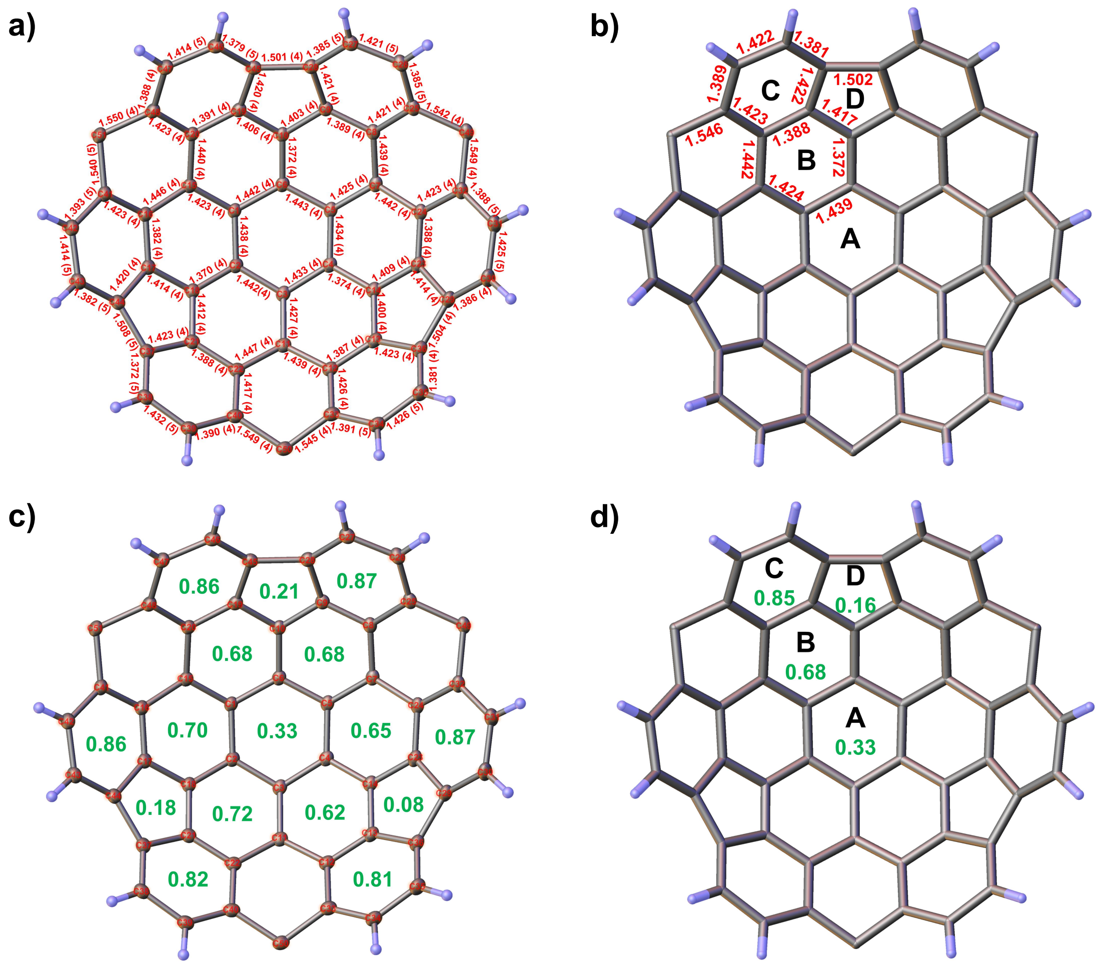

Fig. S8. Bond lengths (in Å) and HOMA values of 1a from X-ray structure. (a) Individual C-C bond lengths (red). (b) Symmetry-averaged bond lengths. (c) Individual HOMA values (green) for each ring. (d) Symmetry-averaged HOMA values. The *p*-trifluoromethylphenyl groups are omitted for clarity. HOMA = $1-\alpha/N\ \Sigma(R_f-R_i)^2$, where N = number of atoms, $\alpha = 257.7$, $R_f = 1.388$ Å, and R_i = individual bond length (*60*).

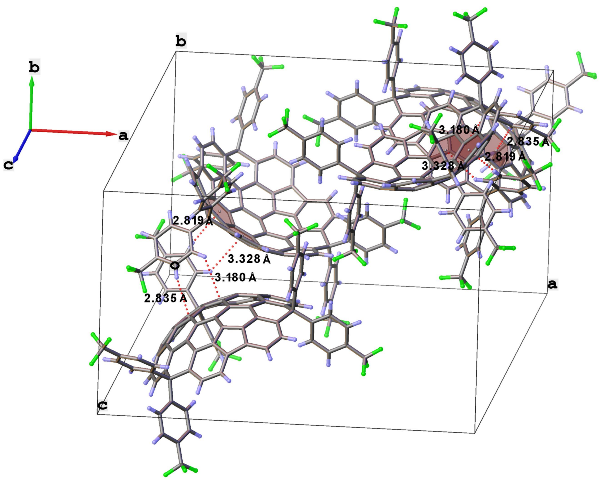

Fig. S9. Unit cell of 1a. Containing four molecules arranged as two pairs of interlocked dimers in a centrosymmetric pattern.

2.3 X-ray crystallography for 1b

Single crystals of compound **1b** suitable for X-ray structural determination were obtained as yellow needles by slowly diffusing methanol vapor into its solution in 1,1,2,2-tetrachloroethane.

Table S3. Crystallographic data and structure refinement details for 1b (CCDC: 2444404).

Empirical formula	$C_{63}H_{42}(C_2H_2Cl_4)_2$
Formula weight	1134.64
Temperature/K	150.15
Crystal system	orthorhombic
Space group	Pnma
a/Å	14.6835(3)
b/Å	18.7025(4)
c/Å	18.2159(4)
α/°	90
β/°	90
γ/°	90
Volume/Å^3	5002.42(18)
Z	4
ρ_{calc}g/cm^3	1.507
μ/mm^{-1}	4.476
F(000)	2336.0
Crystal size/mm^3	0.5 × 0.3 × 0.2
Radiation	CuKα (λ = 1.54178)
2Θ range for data collection/°	7.734 to 136.6
Index ranges	$-17 \le h \le 16$, $-22 \le k \le 22$, $-21 \le l \le 21$
Reflections collected	32040
Independent reflections	4713 [R_{int} = 0.0631, R_{sigma} = 0.0351]
Data/restraints/parameters	4713/280/464
Goodness-of-fit on F^2	1.039
Final R indexes [I>=2σ (I)]	R_1 = 0.0366, wR_2 = 0.0977
Final R indexes [all data]	R_1 = 0.0464, wR_2 = 0.1000
Largest diff. peak/hole / e Å^{-3}	0.35/-0.26

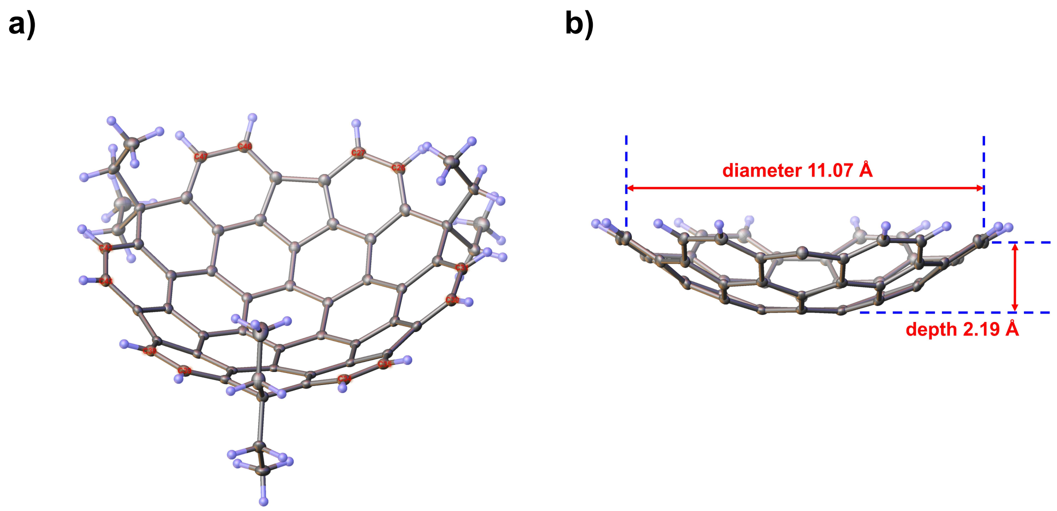

Fig. S10. Crystal structure of 1b. (a) Perspective view. (b) Side view showing diameter and depth. Solvent molecules and ethyl groups omitted for clarity. Thermal ellipsoids at 50% probability.

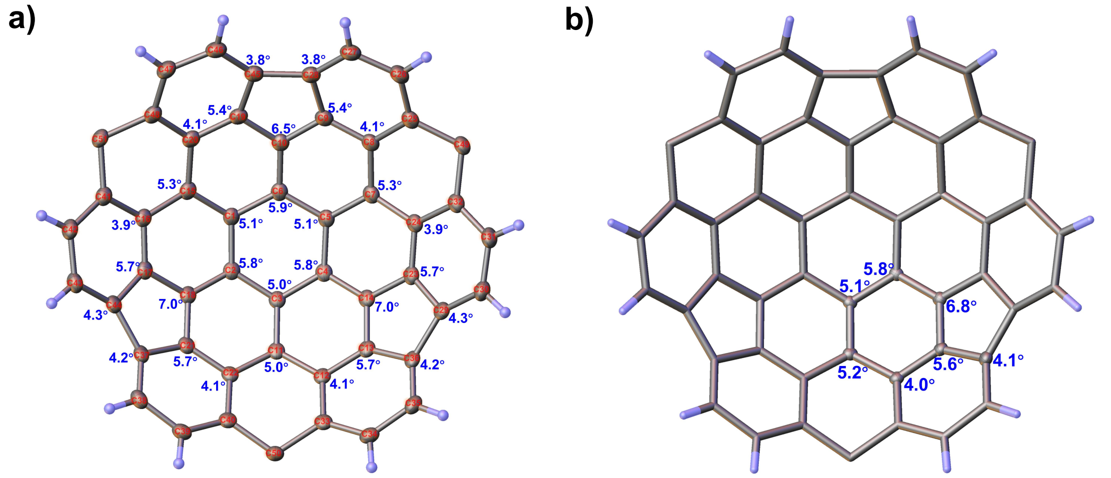

Fig. S11. POAV angles (blue) of 1b from X-ray structure. (a) Individual angles for each carbon atom. (b) Symmetry-averaged angles. Ethyl groups omitted for clarity.

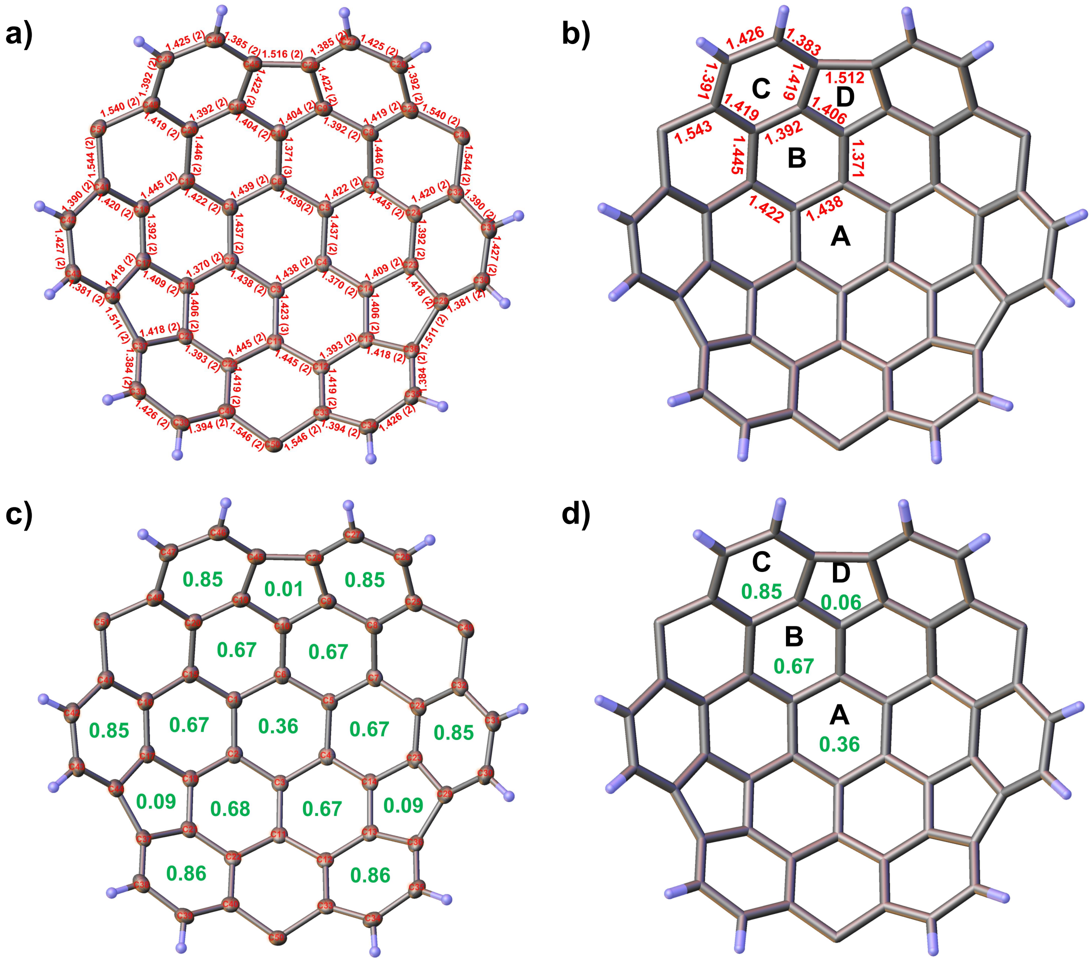

Fig. S12. Bond lengths (in Å) and HOMA values of 1b from X-ray structure. (a) Individual C-C bond lengths (red). (b) Symmetry-averaged bond lengths. (c) Individual HOMA values (green) for each ring. (d) Symmetry-averaged HOMA values. Ethyl groups omitted for clarity.

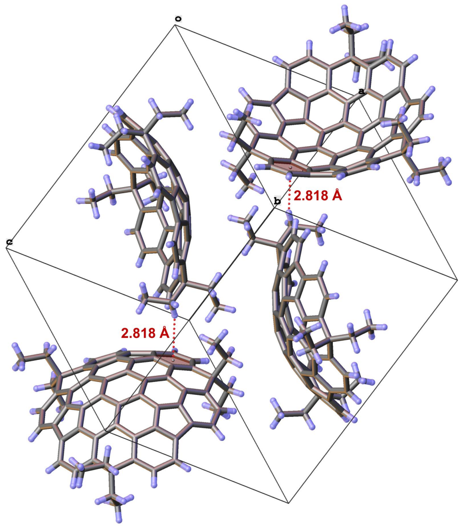

Fig. S13. Unit cell of 1b. Containing four molecules arranged as two pairs of dimers in a centrosymmetric pattern.

2.4 X-ray crystallography for ^{t}Bu$_3$-HBC

Single crystals of compound ^{t}Bu$_3$-HBC suitable for X-ray structural determination were obtained as orange needles by slowly diffusing methanol vapor into its solution in chloroform.

Table S4. Crystallographic data and structure refinement details for ^{t}Bu$_3$-HBC (CCDC: 2444406).

Empirical formula	$C_{60}H_{48}$
Formula weight	768.98
Temperature/K	237.00
Crystal system	triclinic
Space group	P-1
a/Å	13.8043(5)
b/Å	16.3637(7)
c/Å	21.0543(8)
α/°	72.487(2)
β/°	83.276(2)
γ/°	82.218(2)
Volume/Å^3	4479.0(3)
Z	4
ρ_{calc}g/cm^3	1.140
μ/mm^{-1}	0.485
F(000)	1632.0
Crystal size/mm^3	0.3 × 0.2 × 0.1
Radiation	CuKα (λ = 1.54178)
2Θ range for data collection/°	4.416 to 136.866
Index ranges	$-16 \leq h \leq 16$, $-19 \leq k \leq 19$, $-25 \leq l \leq 25$
Reflections collected	141110
Independent reflections	16407 [R_{int} = 0.0593, R_{sigma} = 0.0272]
Data/restraints/parameters	16407/75/1130
Goodness-of-fit on F^2	1.045
Final R indexes [I>=2σ (I)]	R_1 = 0.0508, wR_2 = 0.1430
Final R indexes [all data]	R_1 = 0.0682, wR_2 = 0.1566
Largest diff. peak/hole / e Å^{-3}	0.37/-0.31

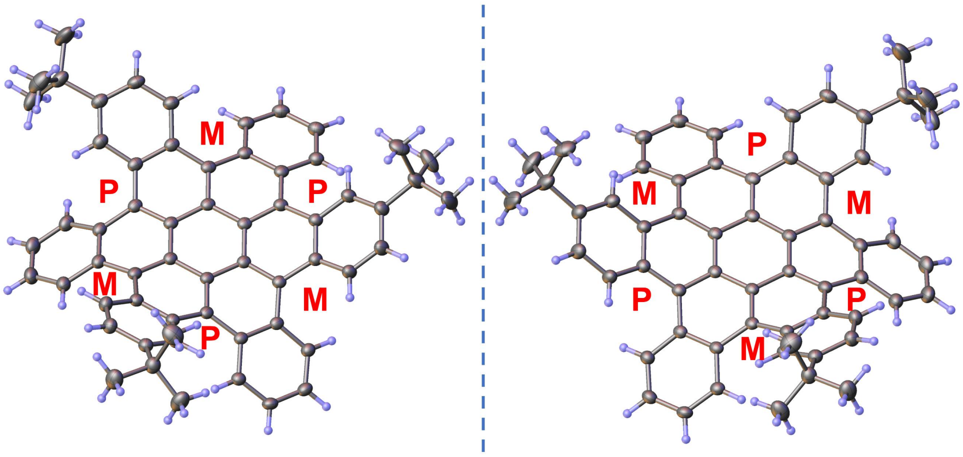

Fig. S14. Crystal structure of ^{t}Bu$_3$-HBC. Top view of enantiomer pair of ^{t}Bu$_3$-HBC, MPMPMP-isomer (left) and PMPMPM-isomer (right). Thermal ellipsoids are shown at 30% probability.

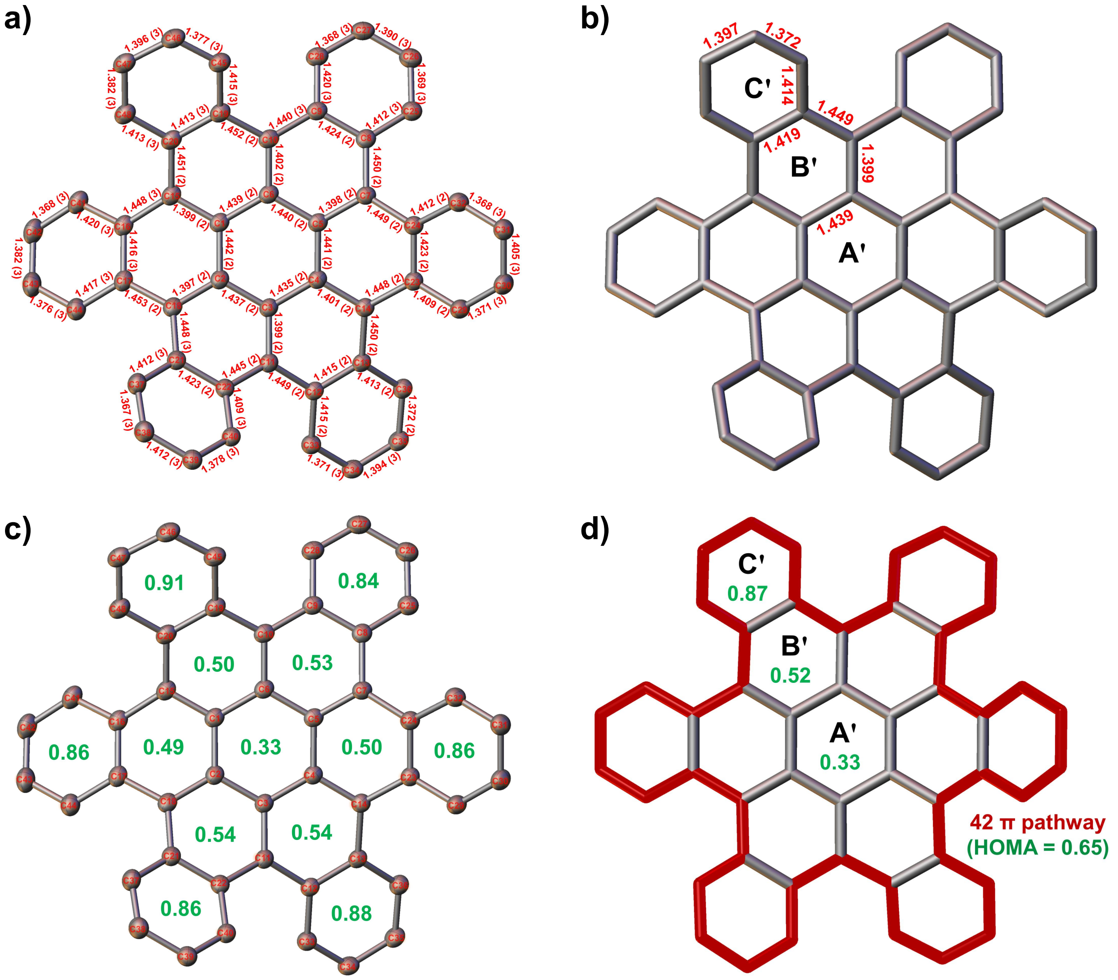

Fig. S15. Bond lengths (in Å) and HOMA values of 1b from X-ray structure. (a) Individual C-C bond lengths (red). (b) Symmetry-averaged bond lengths. (c) Individual HOMA values (green) for each ring. (d) Symmetry-averaged HOMA values. The *tert*-butyl groups and hydrogen atoms are omitted for clarity.

3. Variable-temperature NMR studies

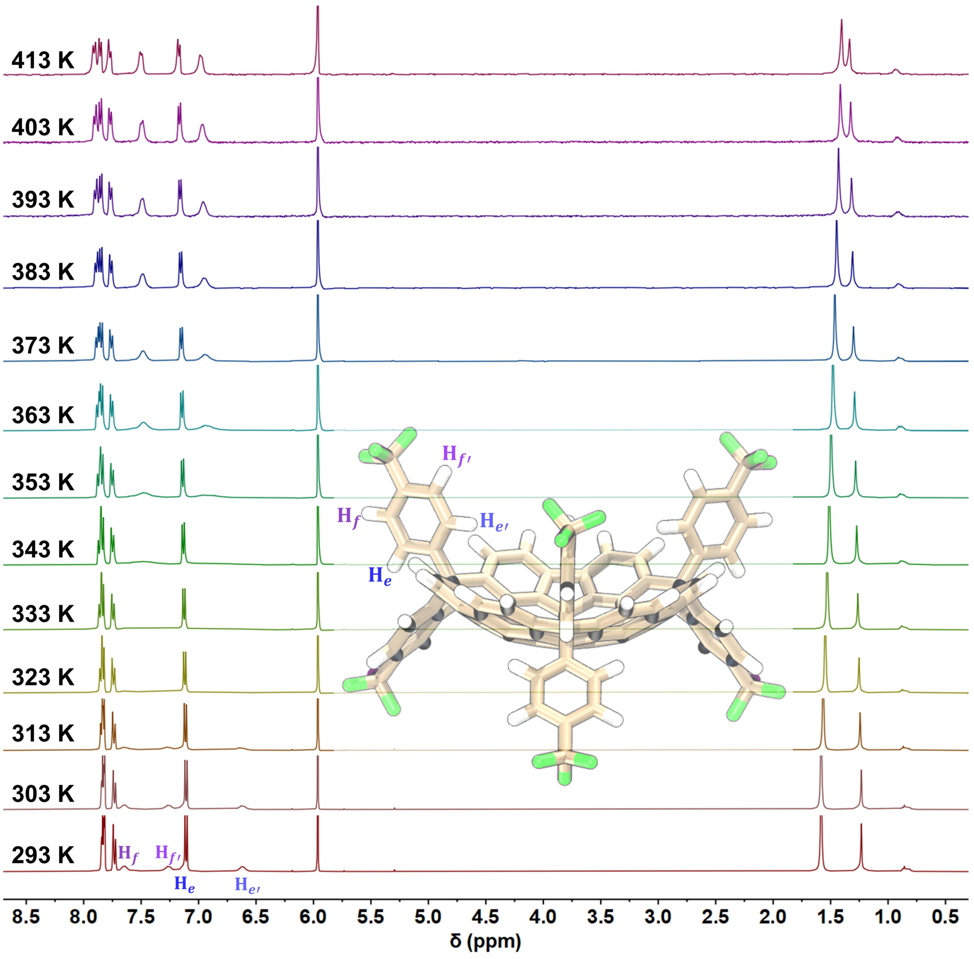

Fig. S16. Variable-temperature ^{1}H NMR spectra of 1a (400 MHz, $C_2D_2Cl_4$, 293–413 K).

According to the Eyring equation, the rotational energy barrier $\Delta G^{\ddagger}$ for **1a** at the coalescence temperature T_c can be derived from the exchange rate constant k_{exch} and the T_c, where Planck's constant h = 6.626 × 10^{-34} J·s, Boltzmann constant k_b = 1.381 × 10^{-23} J/K, and the gas constant R = 8.314 J/mol·K.

$$\Delta G^{\ddagger} = -RT_c \ln\left(\frac{hk_{exch}}{k_b T_c}\right)$$

For the exchange rate constant k_{exch} for **1a** can be calculated using:

$$k_{exch} = \frac{\pi \Delta v_0}{\sqrt{2}}$$

Here, Δv_0 is defined as the chemical shift difference (in Hz) between two sets of proton signals (H_f and $H_{f'}$, H_e and $H_{e'}$) in the slow exchange process at low temperature (293 K). In this particular case, Δv_0 (H_e &$H_{e'}$) = 204 Hz, Δv_0 (H_f &$H_{f'}$) = 156 Hz, T_c = 323 K. Finally, $\Delta G^{\ddagger}$(H_f &$H_{f'}$) = 29.7 kcal·mol^{-1}, $\Delta G^{\ddagger}$(H_e &$H_{e'}$) = 27.8 kcal·mol^{-1}, the rotational energy barrier $\Delta G_{ro}^{\ddagger}$ = (29.7 + 27.8) / 2 = 28.8 (± 0.7) kcal·mol^{-1}.

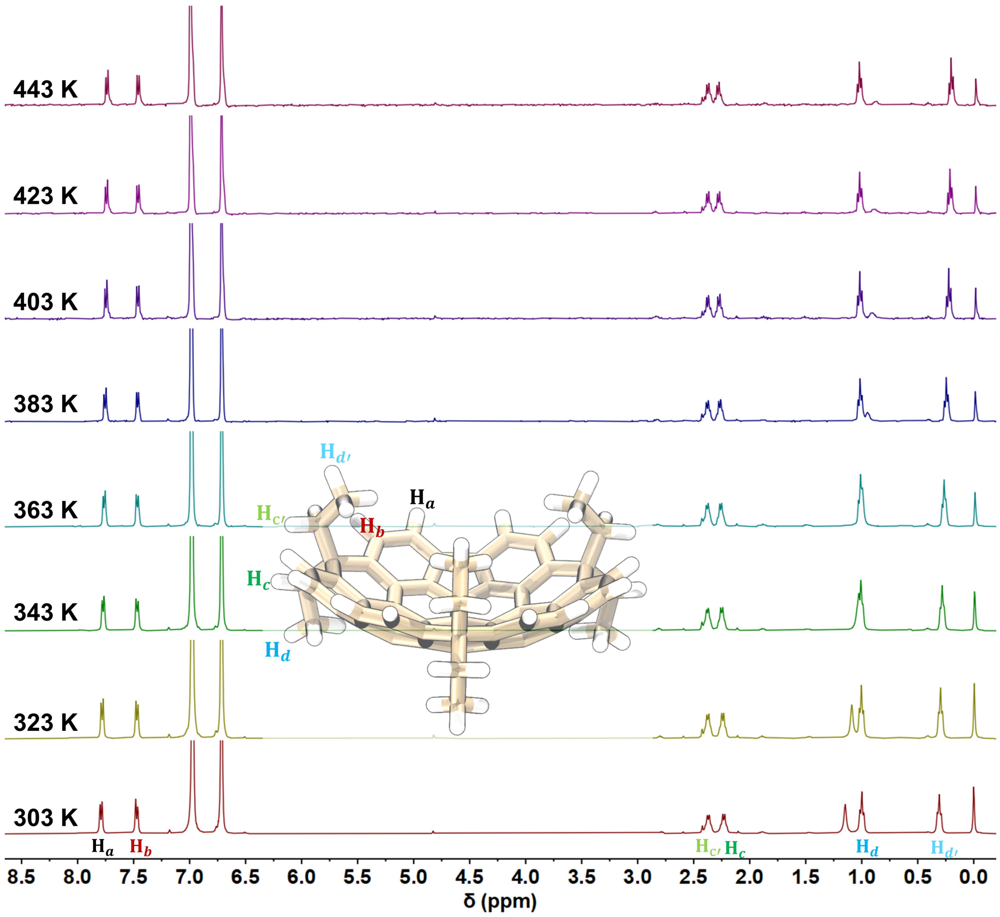

Fig. S17. Variable-temperature ^{1}H NMR spectra of 1b (400 MHz, 1,2-dichlorobenzene-*d*$_4$, 303–443 K).

4. Photophysical properties

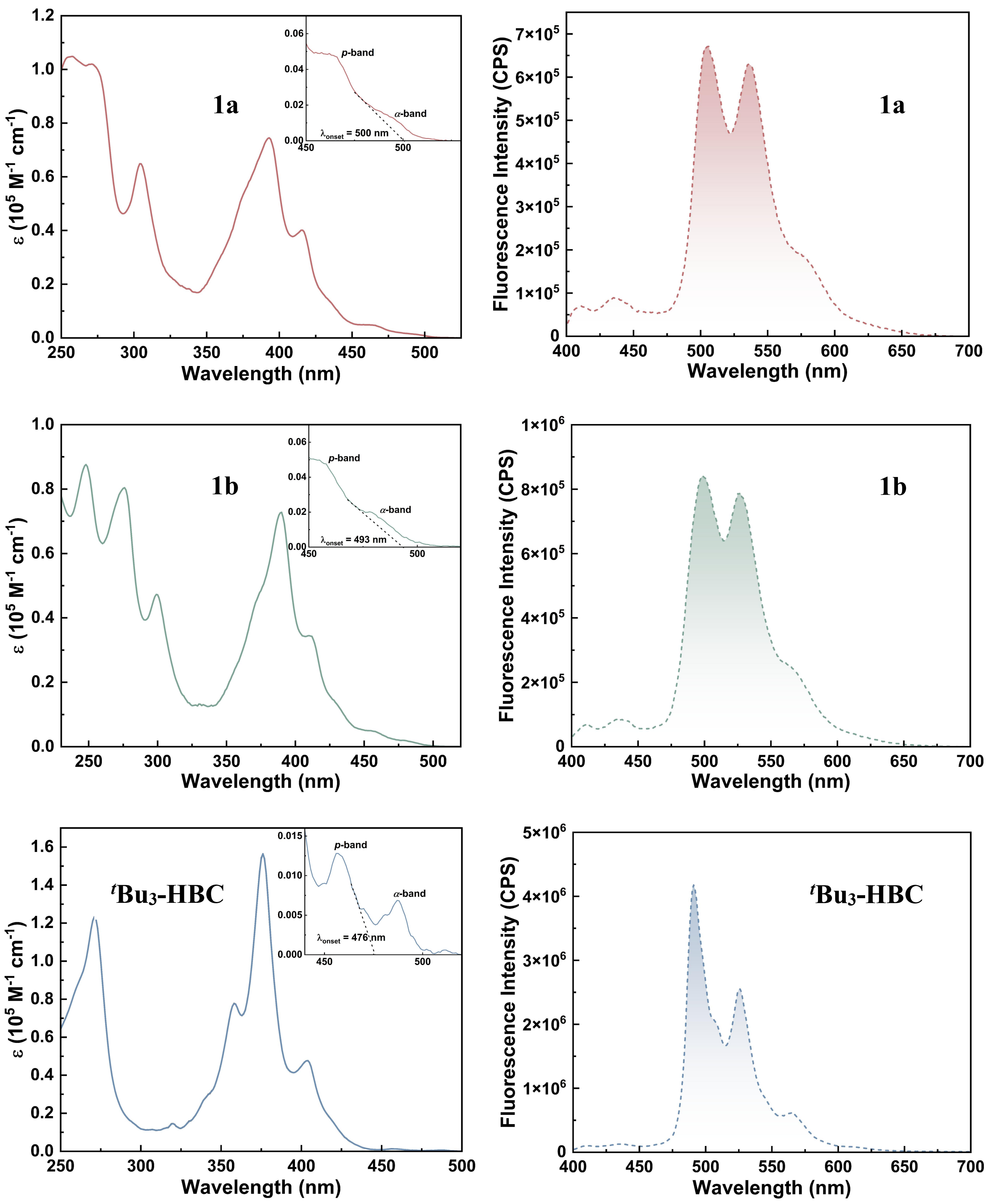

Fig. S18. Optical absorption and emission spectra. UV-Vis absorption (solid curves, 1.0×10^{-5} M in CH_2Cl_2), and emission (dotted curves, 1.0×10^{-6} M in CH_2Cl_2) spectra.

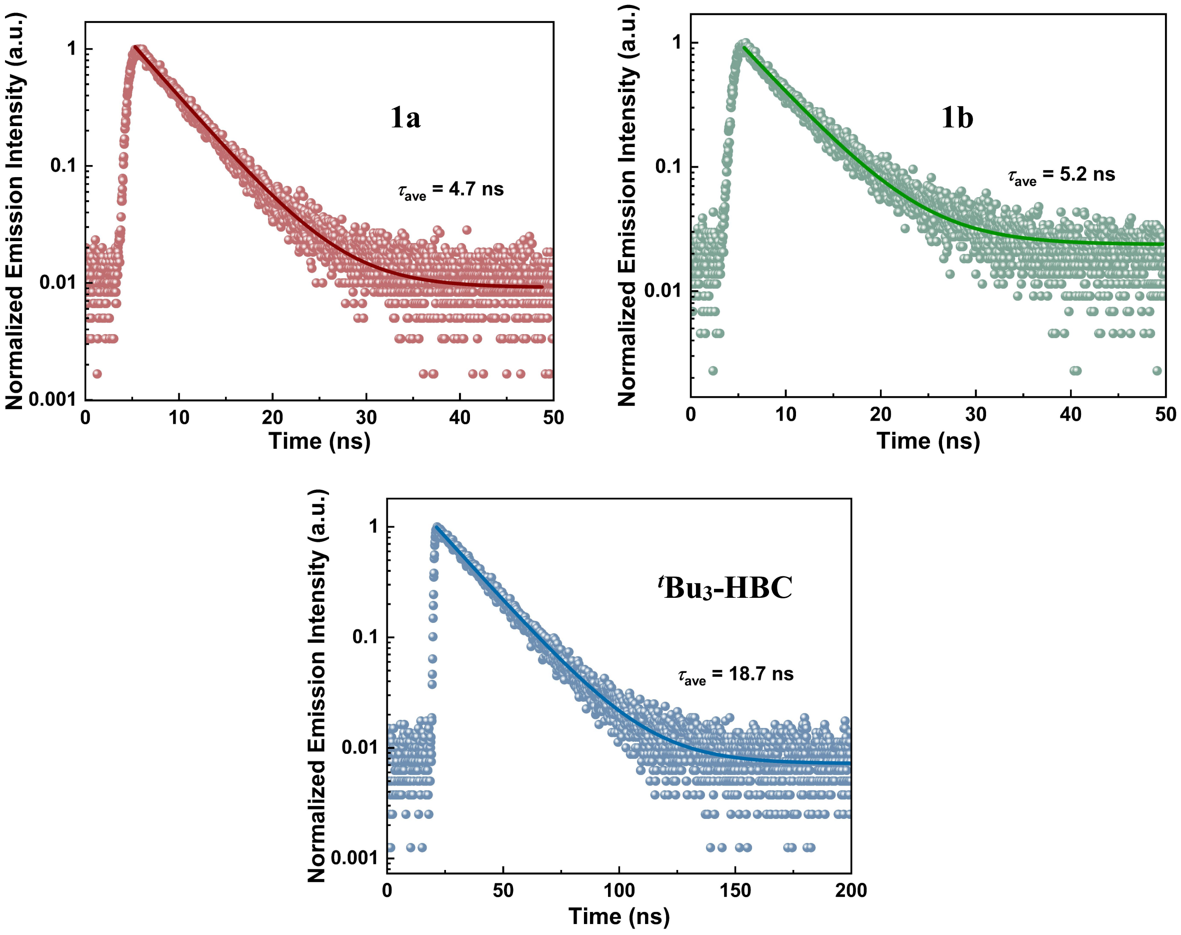

Fig. S19. Time-resolved emission decay spectra (10^{-6} M order in CH_2Cl_2).

Table S5. Summary of optical properties of 1a, 1b, and ^{t}Bu3-HBC.

Compd	λ_{max}^{abs} [nm][a]	λ_{onset} [nm]	λ_{max}^{em} [nm][a]	Stokes shift [nm][b]	Φ_f[c]	τ_f [ns][d]	k_f [ns^{-1}][e]	k_{nr} [ns^{-1}][f]	E_g^{opt} [ev][g]
**1a**	393	500	506,537	113	3.6%	4.7	7.7×10^{-3}	2.1×10^{-1}	2.48
**1b**	390	493	499,527	109	4.2%	5.2	8.1×10^{-3}	1.8×10^{-1}	2.52
**^{t}Bu3-HBC**	376	476	491,526	115	6.0%	18.7	3.2×10^{-3}	5.0×10^{-2}	2.61

[a] Measured in dilute CH_2Cl_2 solution (1.0×10^{-5} M for absorption and 1.0×10^{-6} M for emission). [b] Stokes shift = (λ_{max}^{em} - λ_{max}^{abs}) nm. [c] Absolute fluorescence quantum yield determined with a calibrated integrating sphere system. [d] τ_f: area-weighted mean fluorescence lifetime, $\tau_f = \Sigma(A_n\tau_n^2)/\Sigma(A_n\tau_n)$, where A_n is the coefficient of the exponential functions for the n-th component. [e] k_f: fluorescence emission rate constant, $k_f = \Phi_f/\tau_f$. [f] k_{nr}: nonradiative decay rate constant, $k_{nr} = (1-\Phi_f)/\tau_f$. [g] Calculated by the onset of absorption according to $E_g^{opt} = (1240/\lambda_{onset})$ eV.

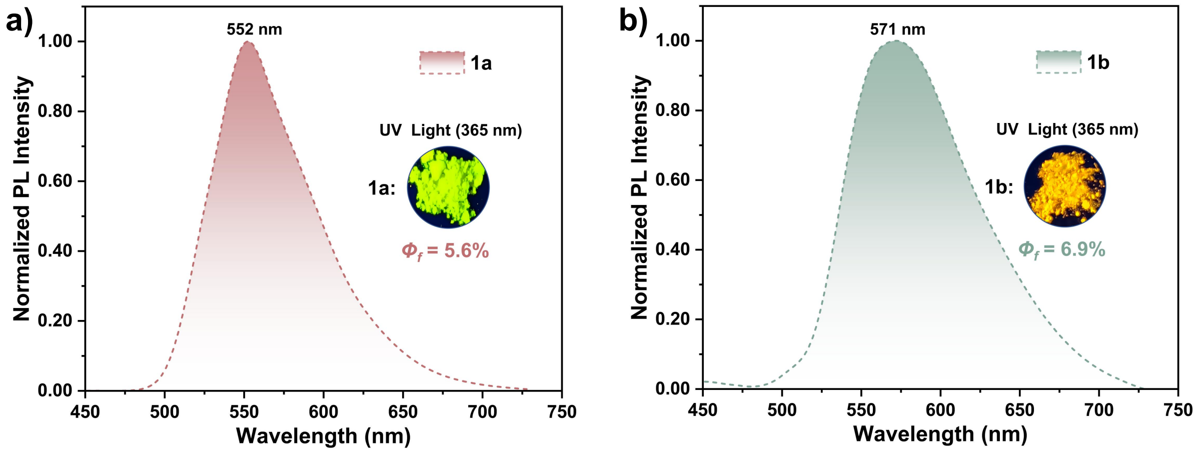

Fig. S20. Solid-state fluorescence spectra of (a) 1a and (b) 1b. Inset: The corresponding photographs under 365 nm UV irradiation, along with the measured absolute fluorescence quantum yield (Φ_f).

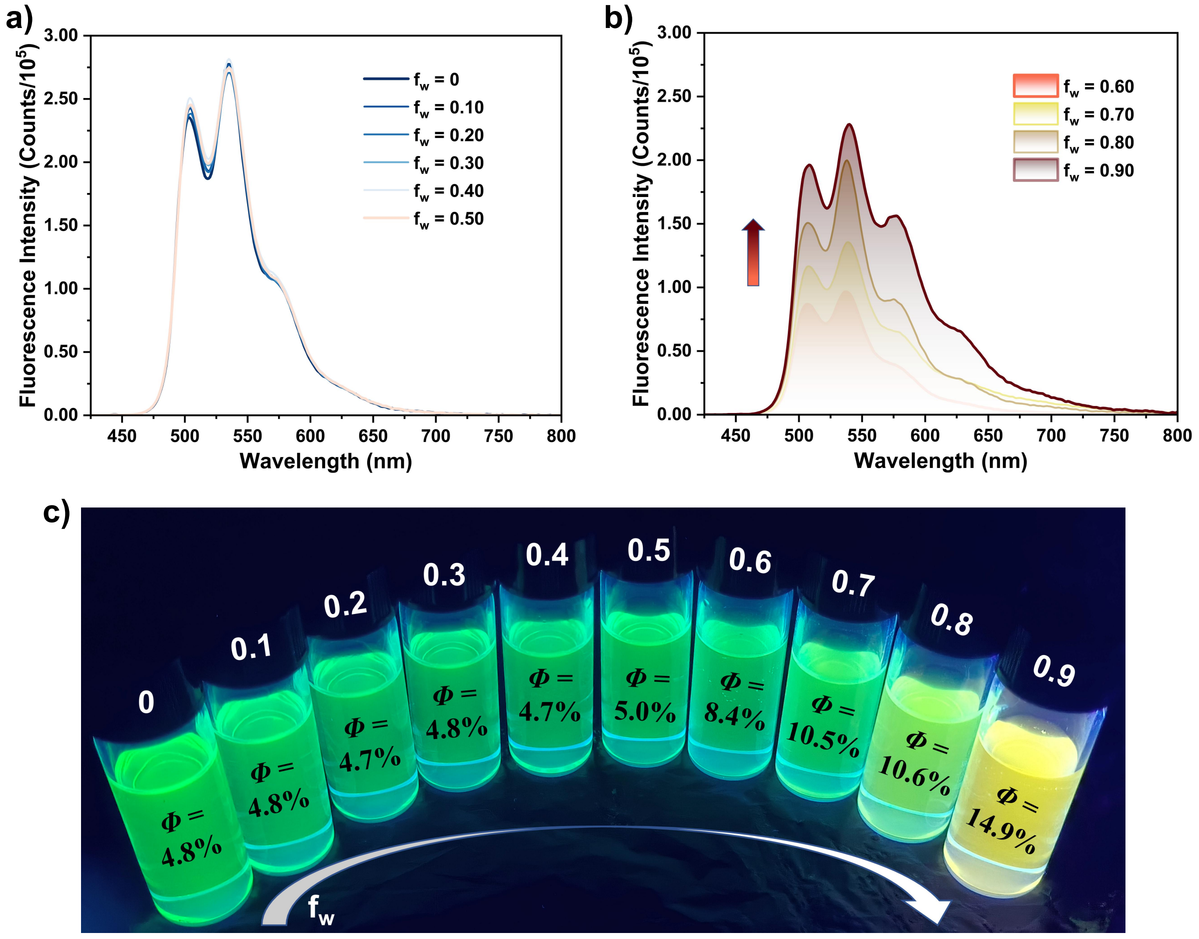

Fig. S21. Aggregation-dependent fluorescence of 1a in THF/water mixtures (1.0 × 10^{-5} M). (a) Emission spectra at low water fractions (0–0.5 vol%). (b) Emission spectra at high water fractions (0.6–0.9 vol%). The legend in (a) and (b) indicates the water fraction (vol%). (c) Photographs of the corresponding solutions under 365 nm UV irradiation, with the measured absolute fluorescence quantum yield (Φ) labeled for each sample.

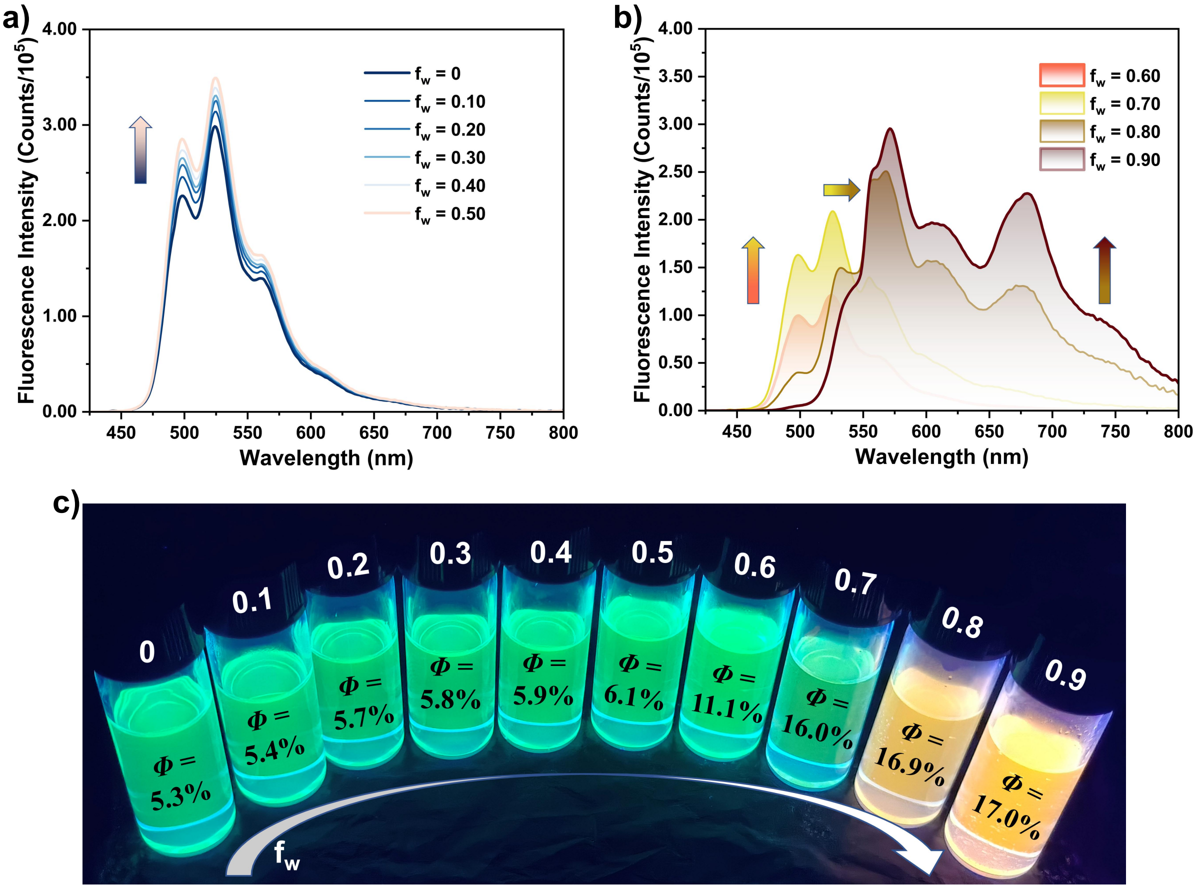

Fig. S22. Aggregation-dependent fluorescence of 1b in THF/water mixtures (1.0 × 10^{-5} M). (a) Emission spectra at low water fractions (0–0.5 vol%). (b) Emission spectra at high water fractions (0.6–0.9 vol%). The legend in (a) and (b) indicates the water fraction (vol%). (c) Photographs of the corresponding solutions under 365 nm UV irradiation, with the measured absolute fluorescence quantum yield (Φ) labeled for each sample.

5. Electrochemical properties

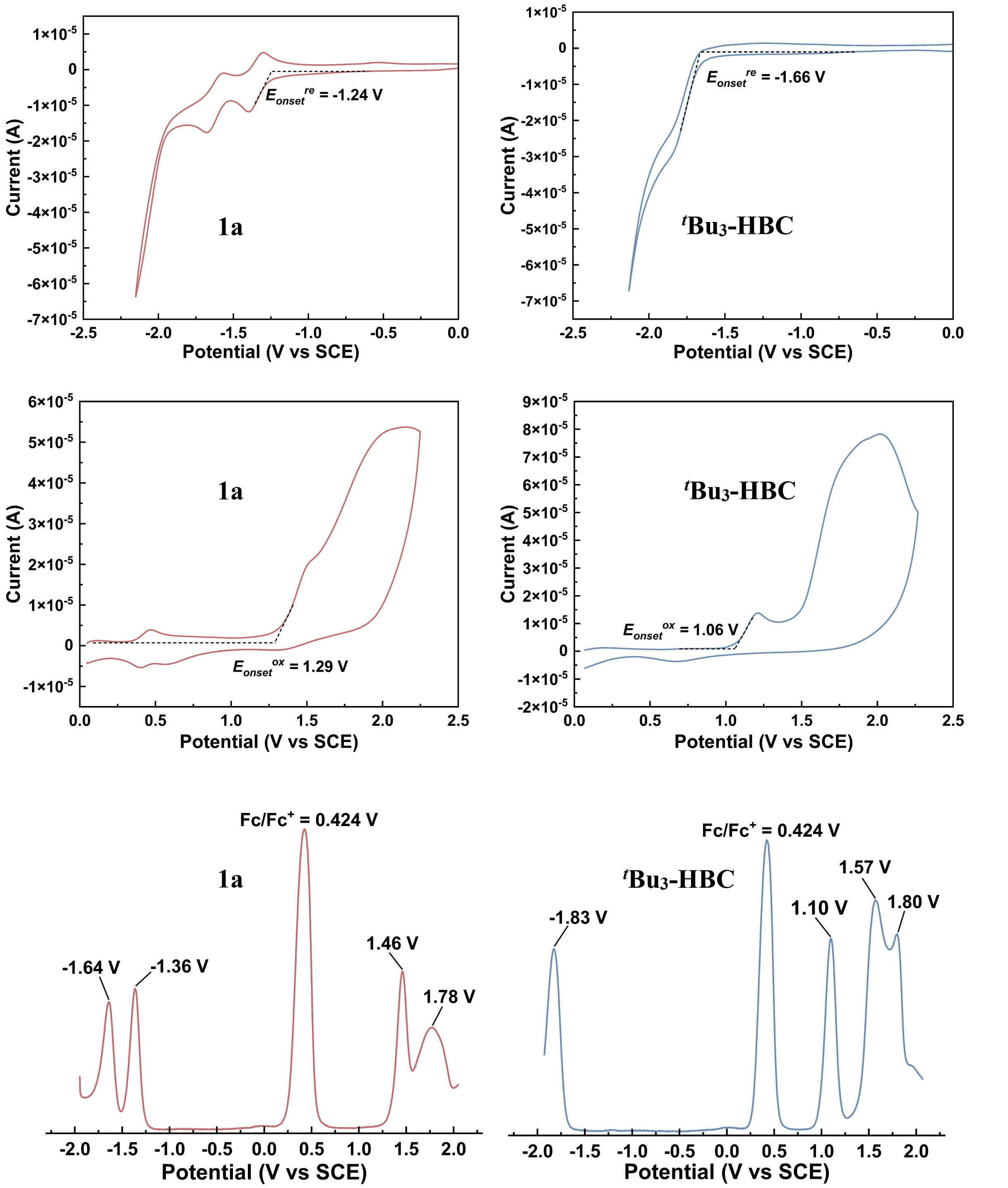

Fig. S23. Electrochemical characterization. Cyclic voltammogram and differential pulse voltammogram of **1a** and ^{t}Bu$_3$-HBC in CH_2Cl_2 (0.1 mol/L n-Bu_4NPF_6) at a scan rate of 0.1 V/s.

Table S6. Summary of electrochemical properties of 1a and ^{t}Bu$_3$-HBC.

Compd	$E_{1/2}^{re}$ [V][a]	E_{onset}^{re} [V]	E_{LUMO} [eV][b]	$E_{1/2}^{ox1}$ [V][a]	E_{onset}^{ox} [V]	E_{HOMO} [eV][c]	E_g^{ele} [eV][d]
**1a**	-1.36	-1.24	-3.14	1.46	1.29	-5.67	2.53
***t*Bu$_3$-HBC**	-1.83	-1.66	-2.72	1.10	1.06	-5.44	2.72

[a] The half-wave potential (V vs SCE) measured in CH_2Cl_2 solution (2×10^{-3} M) by DPV at a scan rate of 0.1 V/s using ferrocene as an internal standard, taking $E_{1/2}$ (Fc/Fc^+) = 0.424 V vs SCE. [b] LUMO estimated by the onset of the first reduction peaks and calculated according to $E_{LUMO} = -(4.376 + E_{onset}^{re})$ eV. [c] HOMO estimated by the onset of the first oxidation peaks and calculated according to $E_{HOMO} = -(4.376 + E_{onset}^{ox})$ eV. [d] Calculated according to $E_g^{ele} = (E_{LUMO} - E_{HOMO})$ eV.

6. Host-Guest chemistry of 1a and 1b towards C_{60}

6.1 ^{1}H NMR spectroscopy titration analyses

^{1}H NMR titration experiments were carried out in 1,2-dichlorobenzene-d_4 solutions. To maintain a constant host concentration throughout the titration, two stock solutions were prepared: Solution A containing host compound (**1a** or **1b**) at 0.5 mM, and Solution B containing host compound (0.5 mM) with C_{60} (15 mM). The titration experiments were performed by successive addition of solution B into 0.4 mL of solution A. After each addition, the solutions were allowed to equilibrate for at least 5 min.

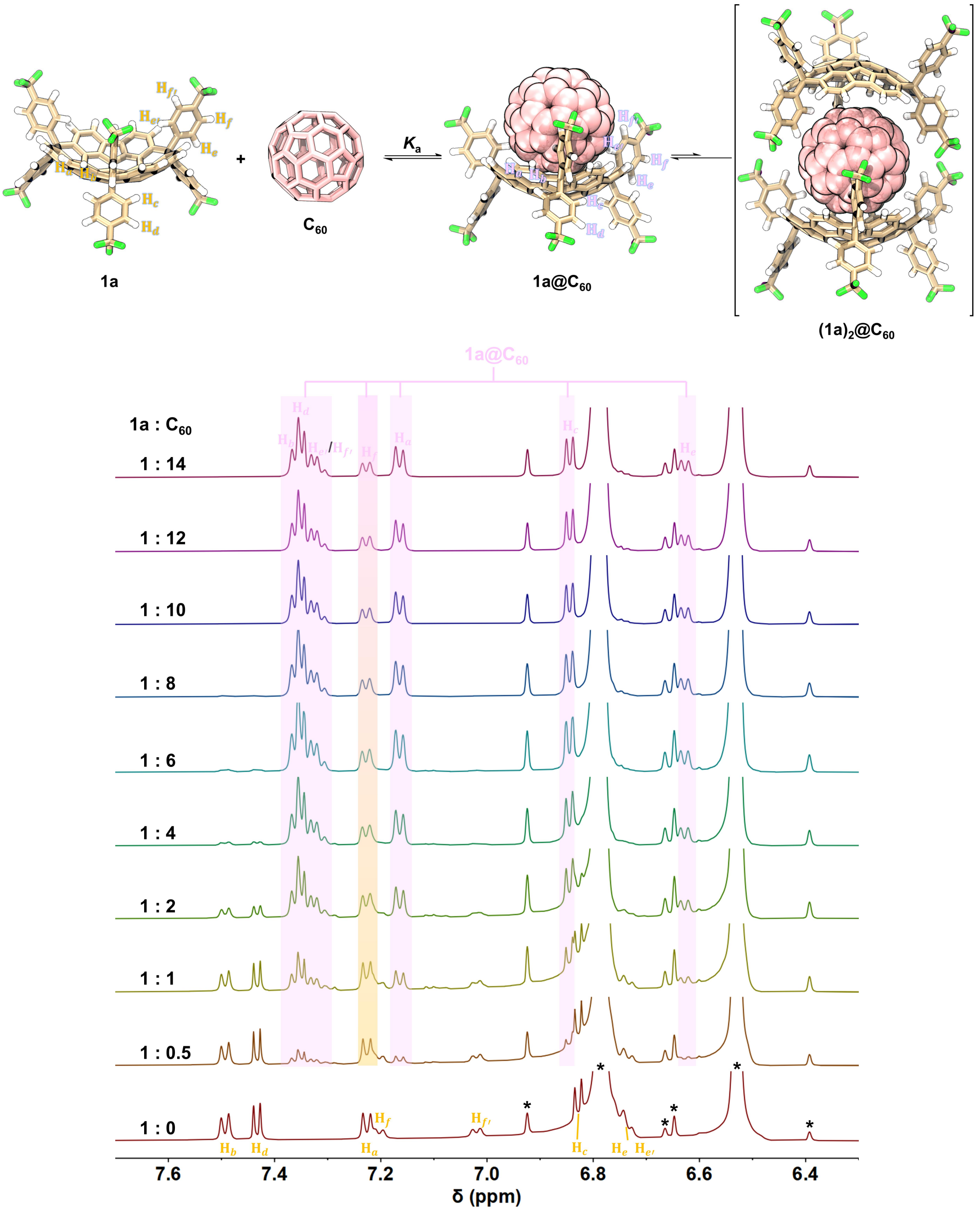

Fig. S24. NMR titration of 1a with C_{60}. ^{1}H NMR spectra titration of **1a** (0.5 mM) with C_{60} from 0 to 14 equivalents (600 MHz, 1,2-dichlorobenzene-d_4, 298 K). ⋆ The solvent and satellite peaks of *o*-DCB-d_4.

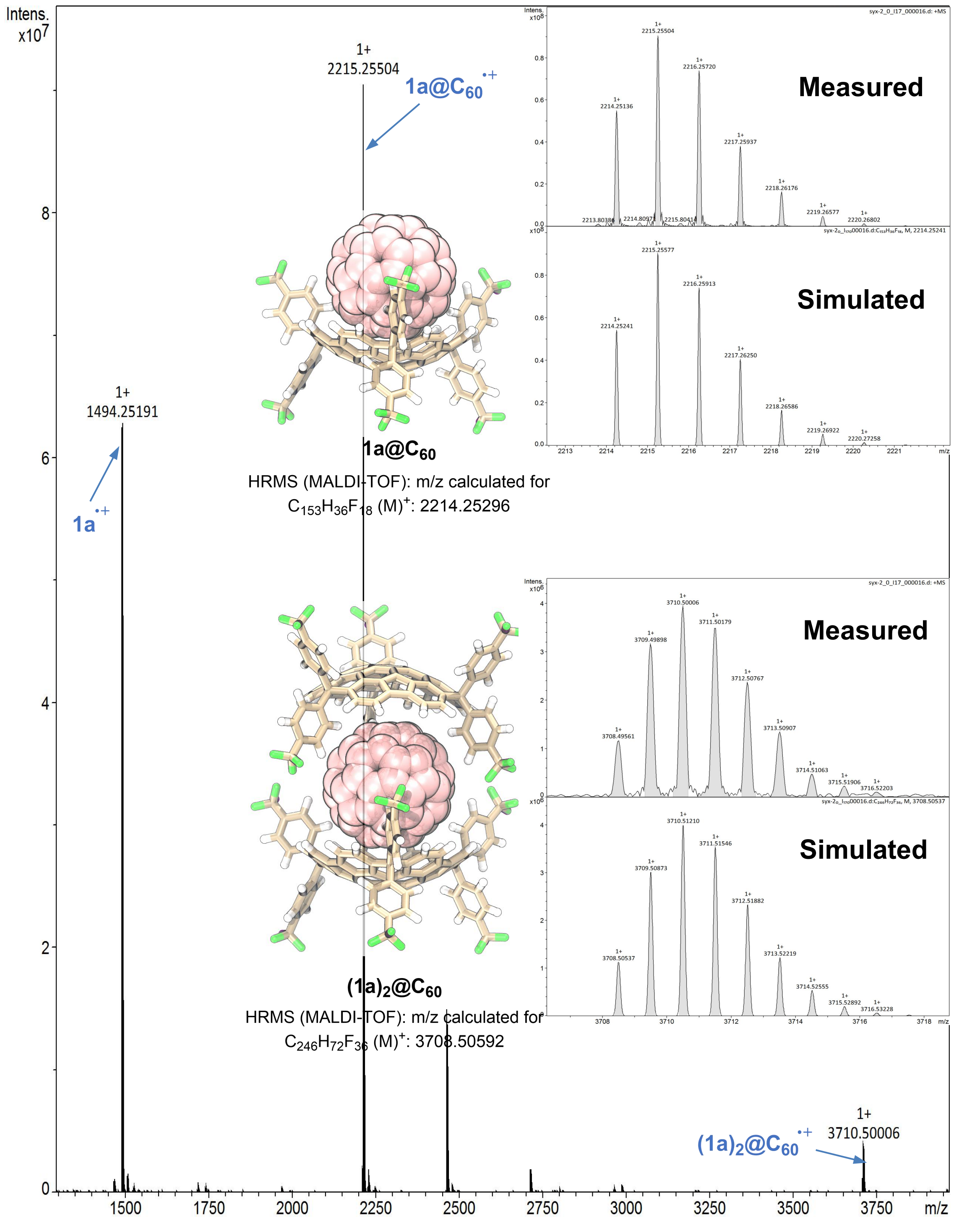

Fig. S25. MALDI-TOF-Mass spectrum of a mixed sample of 1a and C_{60} with a mole ratio of 1:1 in 1,2-dichlorobenzene.

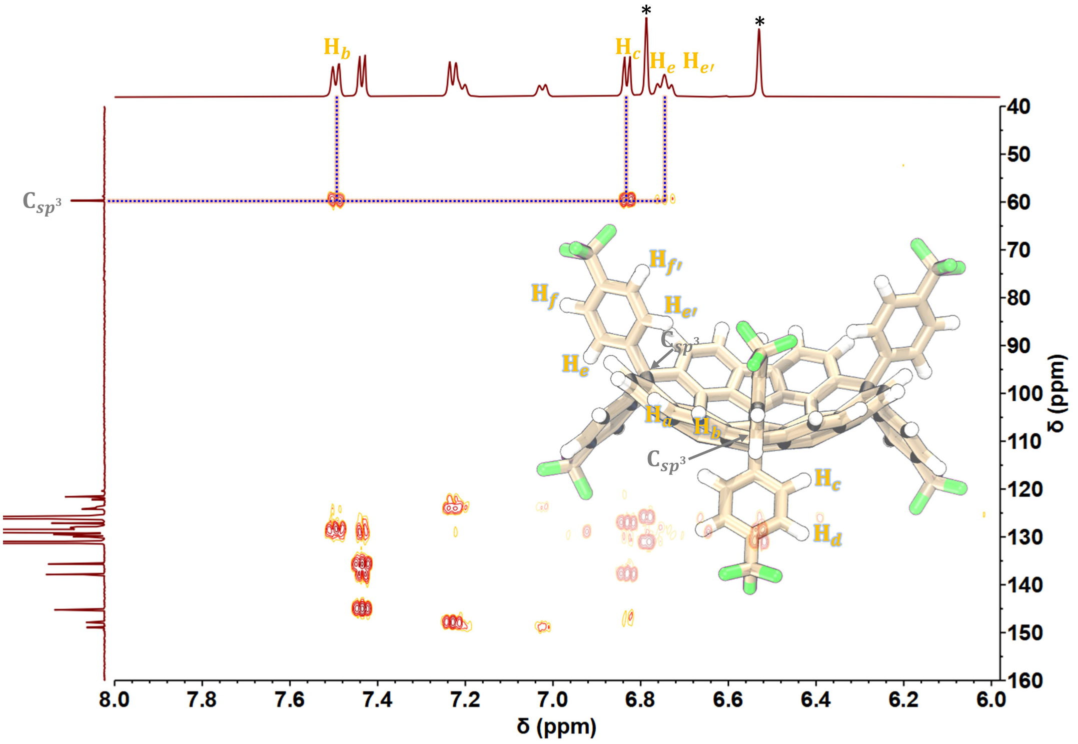

Fig. S26. ^{1}H-^{13}C HMBC NMR spectrum of 1a (600 MHz, 1,2-dichlorobenzene-d_4, 298 K).

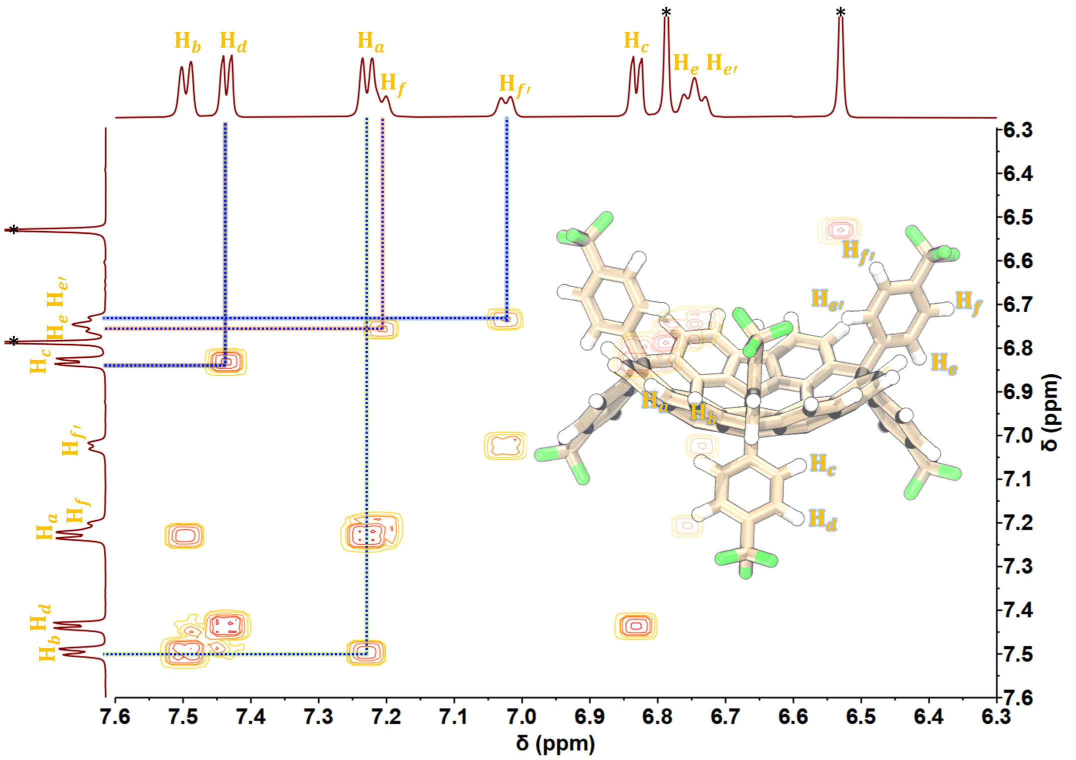

Fig. S27. ^{1}H-^{1}H COSY NMR spectrum of 1a (600 MHz, 1,2-dichlorobenzene-d_4, 298 K).

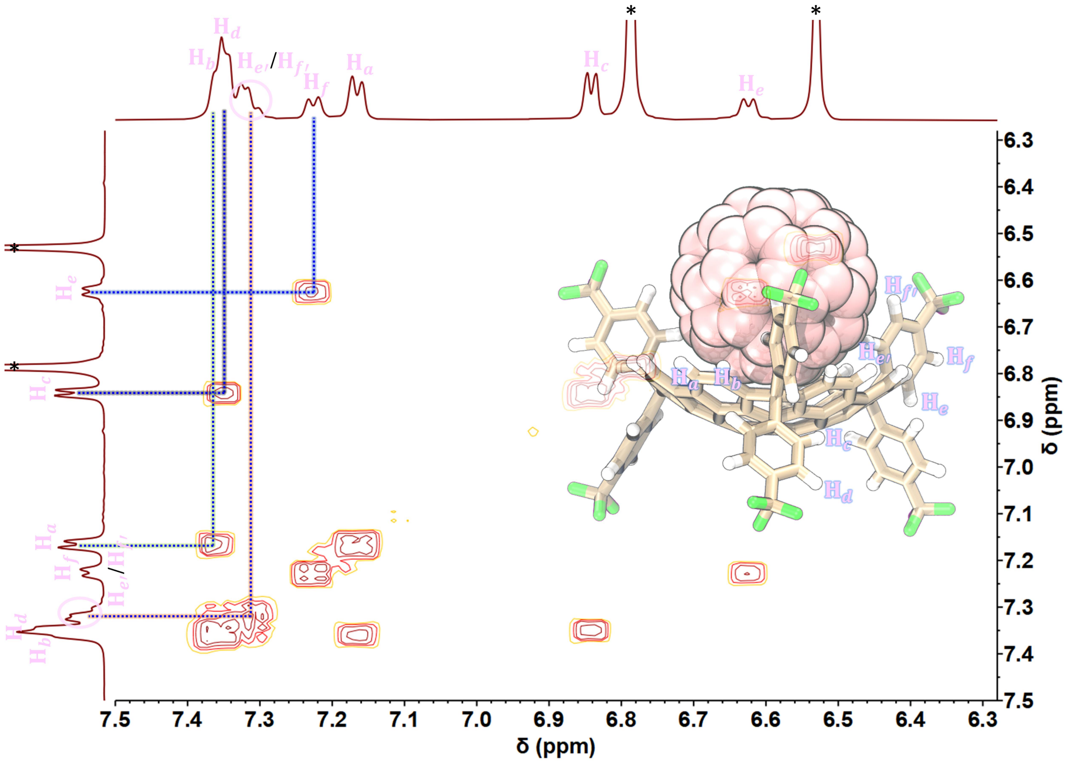

Fig. S28. ^{1}H-^{1}H COSY NMR spectrum of 1a@C_{60} (600 MHz, 1,2-dichlorobenzene-d_4, 298 K).

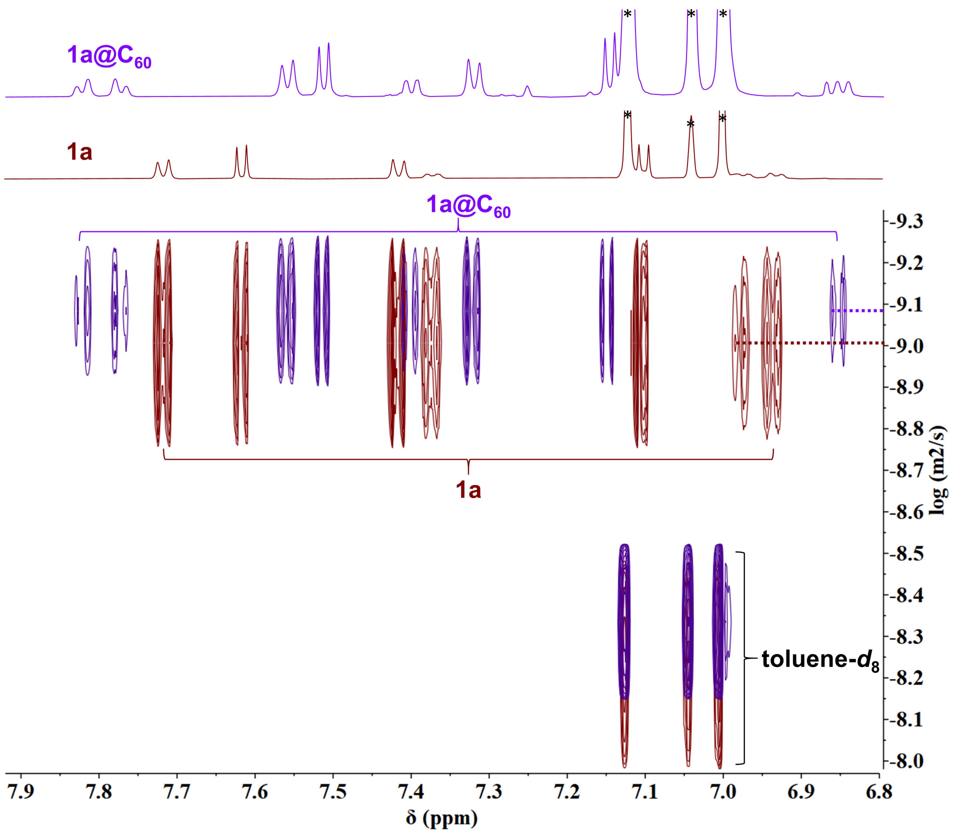

Fig. S29. DOSY NMR spectrum of 1a and 1a@C_{60} (600 MHz, toluene-d_8, 298 K).

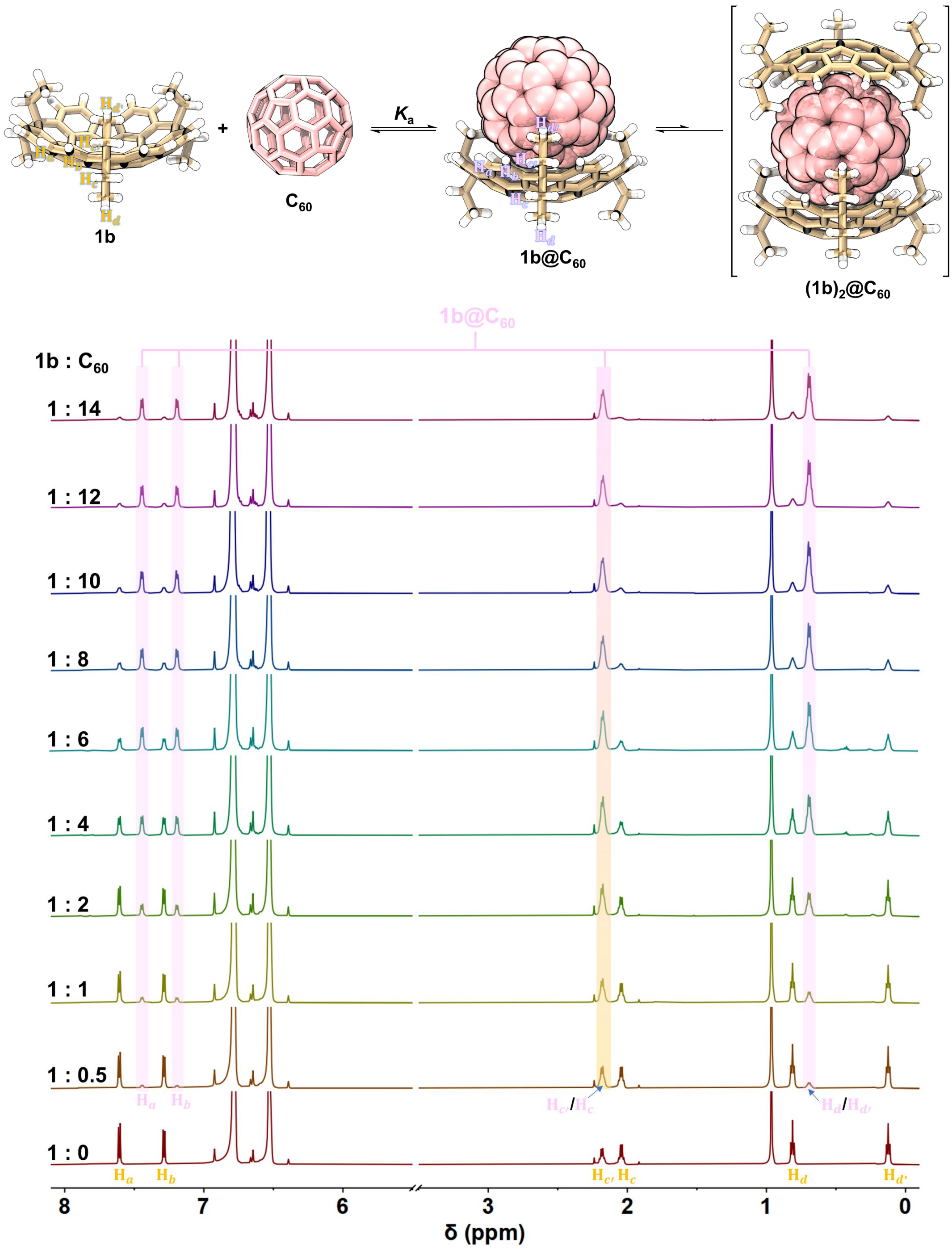

Fig. S30. NMR titration of 1b with C_{60}. ^{1}H NMR spectra titration of **1b** (1.0 mM) with C_{60} from 0 to 14 equivalents (600 MHz, 1,2-dichlorobenzene-*d*4, 298 K).

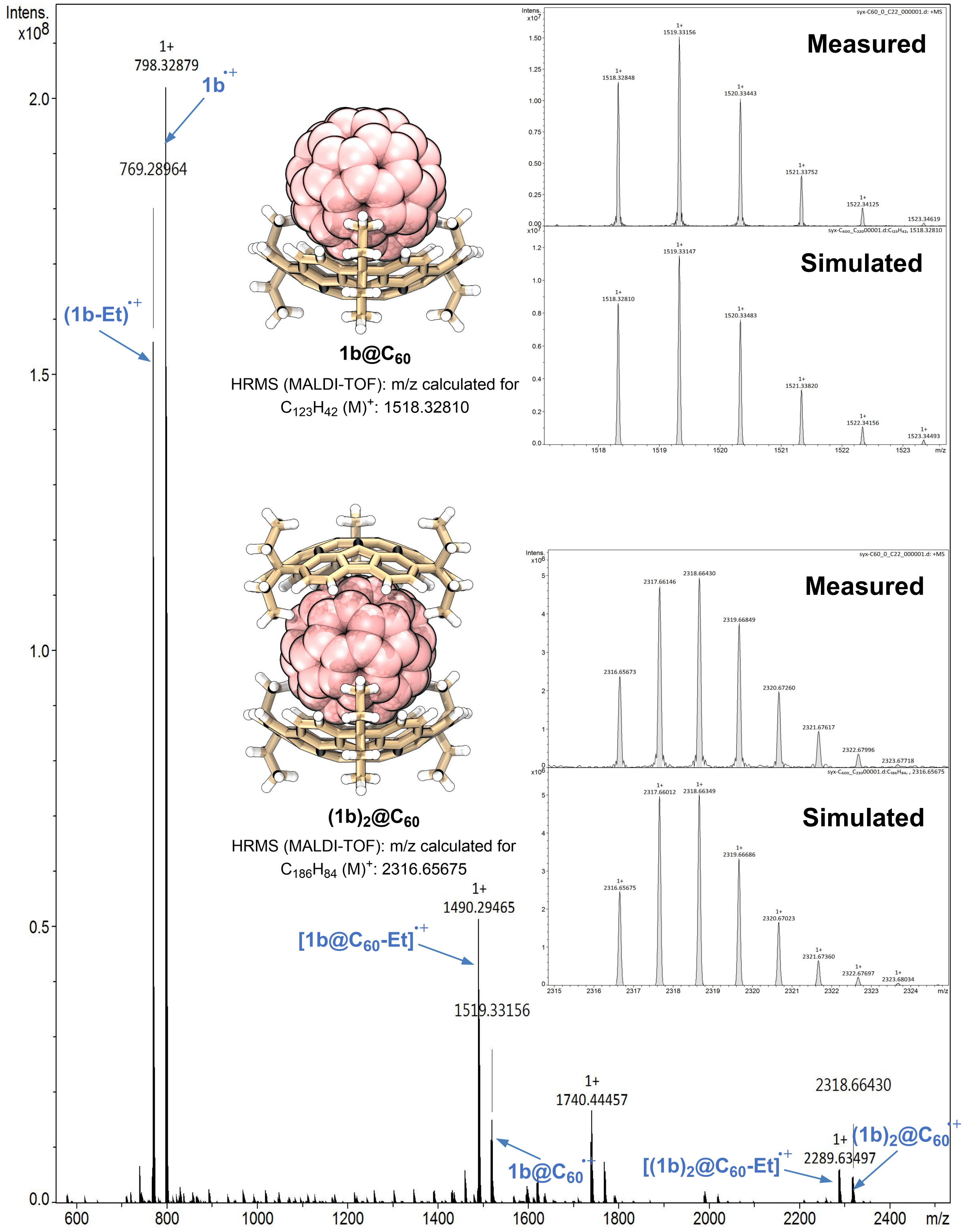

Fig. S31. MALDI-TOF-Mass spectrum of a mixed sample of 1b and C_{60} with a mole ratio of 1:1 in 1,2-dichlorobenzene.

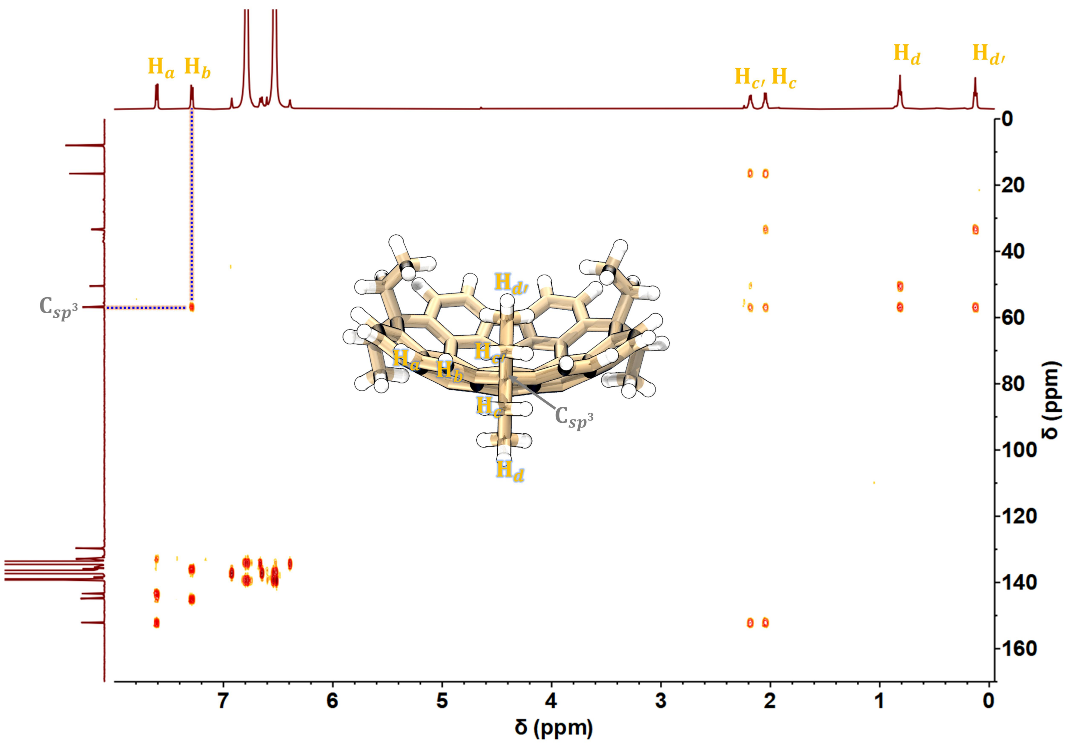

Fig. S32. ^{1}H-^{13}C HMBC NMR spectrum of 1b (600 MHz, 1,2-dichlorobenzene-d_4, 298 K).

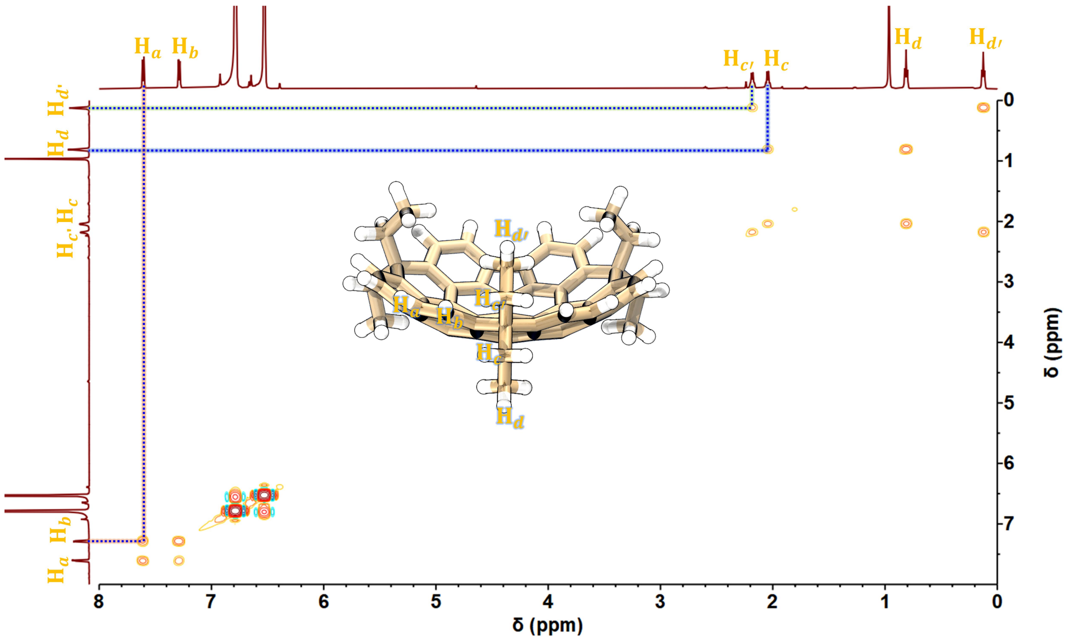

Fig. S33. ^{1}H-^{1}H COSY NMR spectrum of 1b (600 MHz, 1,2-dichlorobenzene-d_4, 298 K).

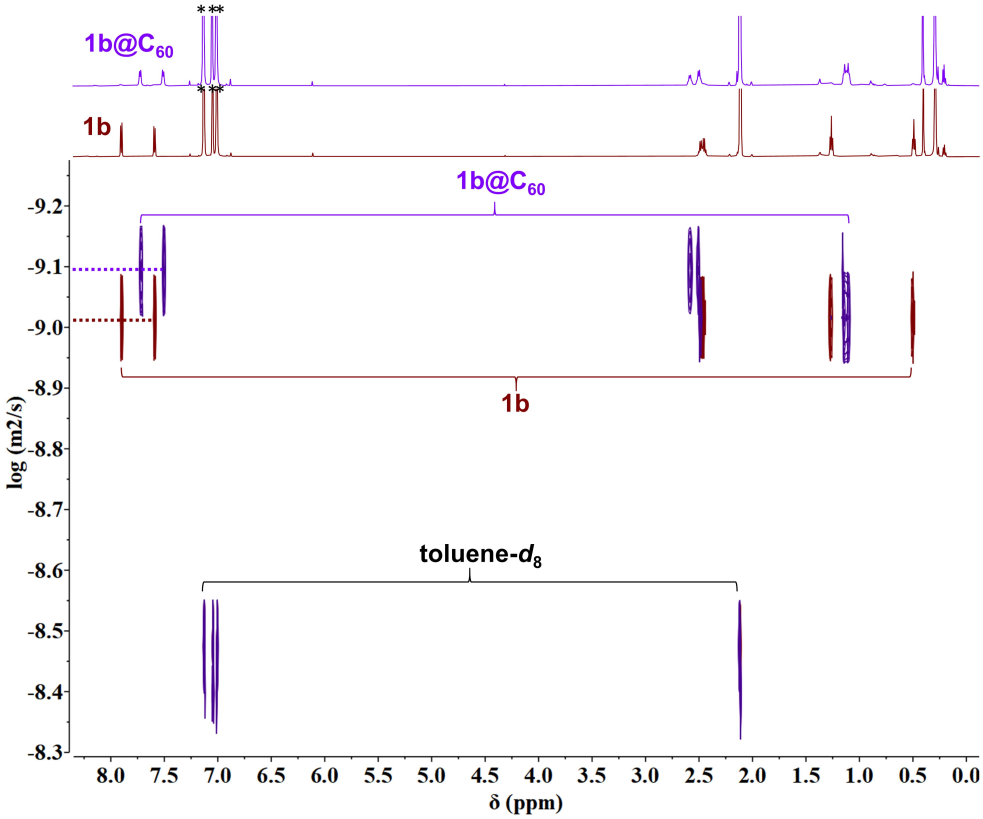

Fig. S34. DOSY NMR spectrum of **1b** and **1b@C_{60}** (600 MHz, toluene-d_8, 298 K).

6.2 Job plot analyses by UV/Vis spectroscopy

To determine the binding stoichiometry between host **1a** (or **1b**) and guest C_{60} in solution, Job's plot experiments based on UV/Vis spectroscopy were carried out in toluene. A solution of guest C_{60} in toluene (1.5×10^{-5} M) and a solution of host in toluene (1.5×10^{-5} M) were mixed in different ratios to prepare 12 samples, while the sum of the concentrations of host and C_{60} was kept constant at 1.5×10^{-5} M. After thorough mixing and equilibration, the UV/Vis spectrum of each sample was recorded. The resulting absorbance data at selected wavelengths were used to construct Job's plots, from which the host–guest stoichiometry was derived.

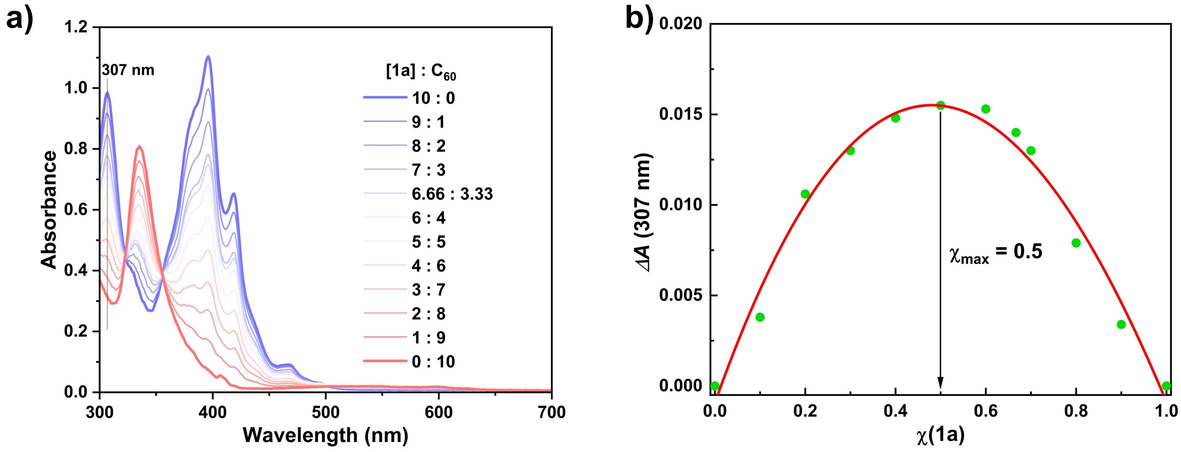

Fig. S35. Job plot analyses for 1a with C_{60} by UV/Vis spectroscopy. (a) UV/Vis spectra of **1a** and C_{60} mixed in different ratios in toluene at 298 K. (b) Job's plot based on the absorbance change at 307 nm, indicating a 1:1 binding stoichiometry between **1a** and C_{60} in toluene.

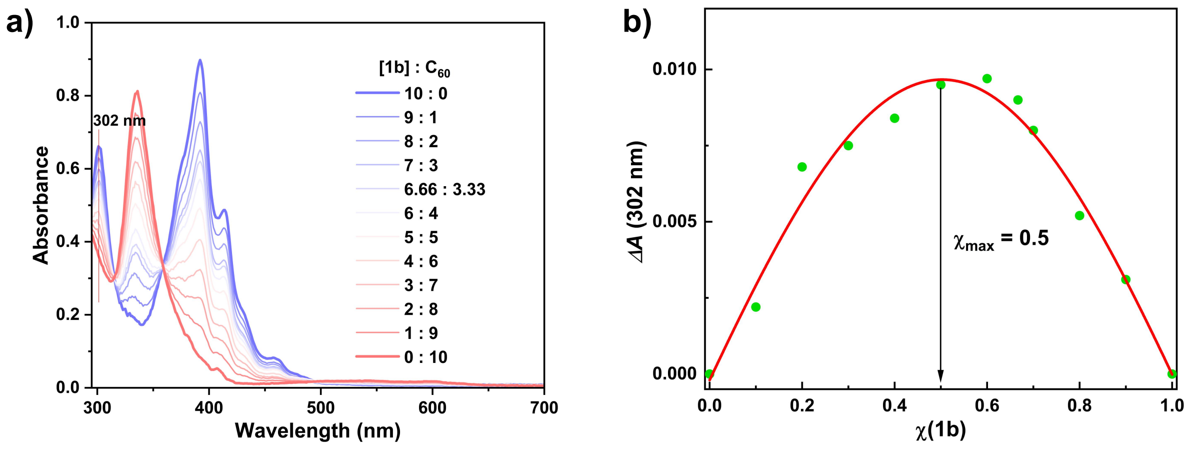

Fig. S36. Job plot analyses for 1b with C_{60} by UV/Vis spectroscopy. (a) UV/Vis spectra of **1b** and C_{60} mixed in different ratios in toluene at 298 K. (b) Job's plot based on the absorbance change at 302 nm, indicating a 1:1 binding stoichiometry between **1b** and C_{60} in toluene.

6.3 UV/Vis titration analyses

UV/Vis titration experiments were carried out in toluene solutions. To maintain a constant host concentration throughout the titration, Stock solution A and B were prepared as shown in Table S7. The titration was performed by successive additions of solution B into 2.0 mL of solution A. After each addition, the solutions were allowed to equilibrate for at least 3 min.

Table S7. Concentrations of stock solutions for UV/Vis titration experiments in toluene.

Host molecule	C_{60}		
	Solution A	Solution B	
	[host] µM	[host] µM	[C_{60}] µM
**1a**	3	3	153
**1b**	5	5	150

Binding constants were determined by fitting UV/Vis titration data using the BindFit program (*71*). To evaluate fitting quality, the cov_{fit} parameter (*70*) was employed, defined as the ratio of residual (co)variance ($y_{\text{data}} - y_{\text{calc}}$) to raw data (co)variance. Global nonlinear regression analysis indicated that the 1:1 binding model provided better fitting results than the 2:1 model.

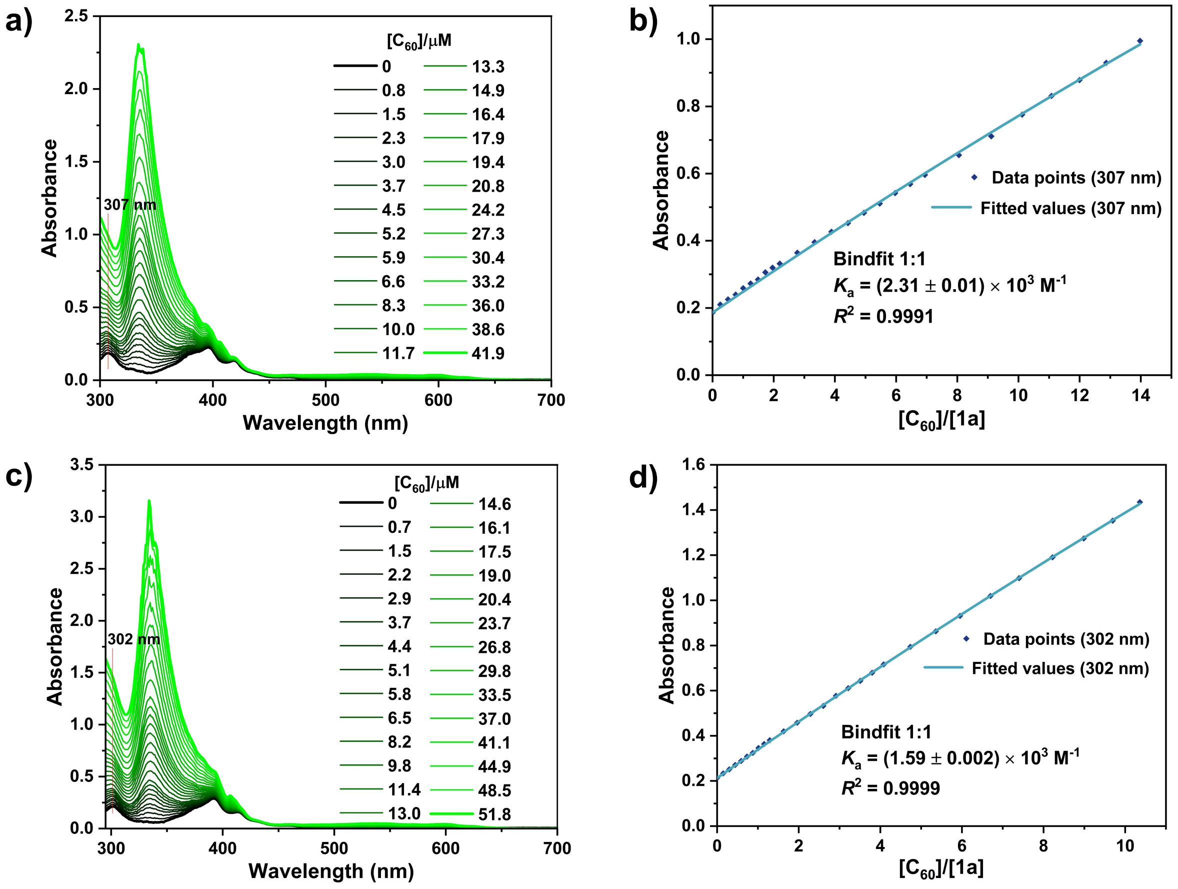

Fig. S37. UV/Vis titration of 1a and 1b with C_{60}. (a) Spectral changes of **1a** (3 µM) upon addition of C_{60}. (b) Non-linear curve fitting for the binding of **1a** with C_{60}. (c) Spectral changes of **1b** (5 µM) upon addition of C_{60}. (d) Non-linear curve fitting for the binding of **1b** with C_{60}.

Table S8. Comparison of different binding modes for the host 1a towards the guest C_{60} in toluene (the appropriate binding mode is highlighted in bold).

Binding mode	cov_{fit} (10^{-4})	cov_{fit} factor[a]	K_1 [M^{-1}]	K_2 [M^{-1}]
**1:1**	**8.19**	**1.00**	**$(2.31 \pm 0.01) \times 10^3$**	–
Full 2:1	7.92	1.03	6.10 ± 0.24	$(1.14 \pm 0.66) \times 10^8$
Non-cooperative 2:1	8.19	1.00	$(2.29 \pm 0.01) \times 10^3$	5.73×10^2 [b]
Additive 2:1	8.15	1.00	91.20 ± 20.32	$(2.05 \pm 21.12) \times 10^6$
Statistical 2:1	8.19	1.00	$(2.29 \pm 0.01) \times 10^3$	5.73×10^2 [b]

[a] The cov_{fit} factor = cov_{fit} for the 1:1 binding model divided by the cov_{fit} for the binding model under study. [b] For the non-cooperative and statistical binding modes, K_2 is calculated as $K_2 = K_1/4$ from the K_1 value obtained.

Conclusion: The cov_{fit} values of the 1:1, full 2:1, non-cooperative 2:1, additive 2:1, and statistical 2:1 binding models are very similar. However, when selecting a binding model, it is justified to choose a more complex model that yields a significantly improved cov_{fit} factor (3-5 fold) compared to a less complex model (*70*). Based on this criterion, we conclude that the 1:1 binding model is the appropriate choice.

1:1 binding mode (1a:C_{60})

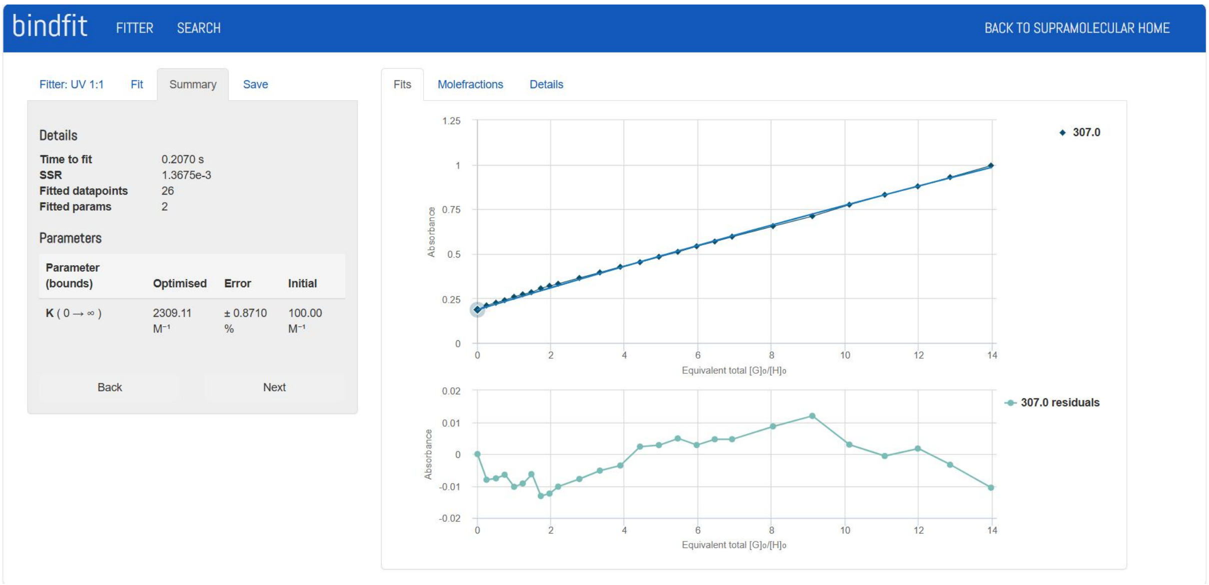

Fig. S38. UV/Vis titration data fitted to a 1:1 binding model. Screenshot of the results window from *http://supramolecular.org*, displaying experimental UV/Vis titration data fitted to a 1:1 binding model. The corresponding dataset is archived at the unique URL: *http://app.supramolecular.org/bindfit/view/3fad9c1f-022d-4985-b3b0-10ce6b831d08*.

Full 2:1 binding mode (1a:C_{60})

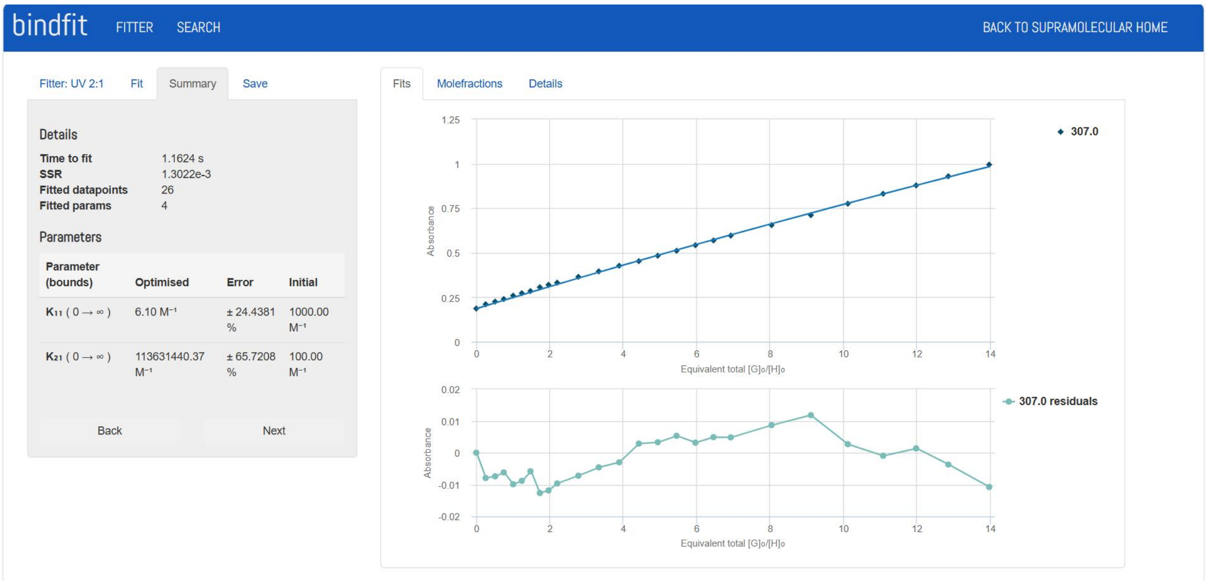

Fig. S39. UV/Vis titration data fitted to a 2:1 binding model. Screenshot of the results window from *http://supramolecular.org*, displaying experimental UV/Vis titration data fitted to a full 2:1 binding model. The corresponding dataset is archived at the unique URL: *http://app.supramolecular.org/bindfit/view/310c0b39-e657-469c-b318-cc67e1a9ed42*.

Non-cooperative 2:1 binding mode (1a:C_{60})

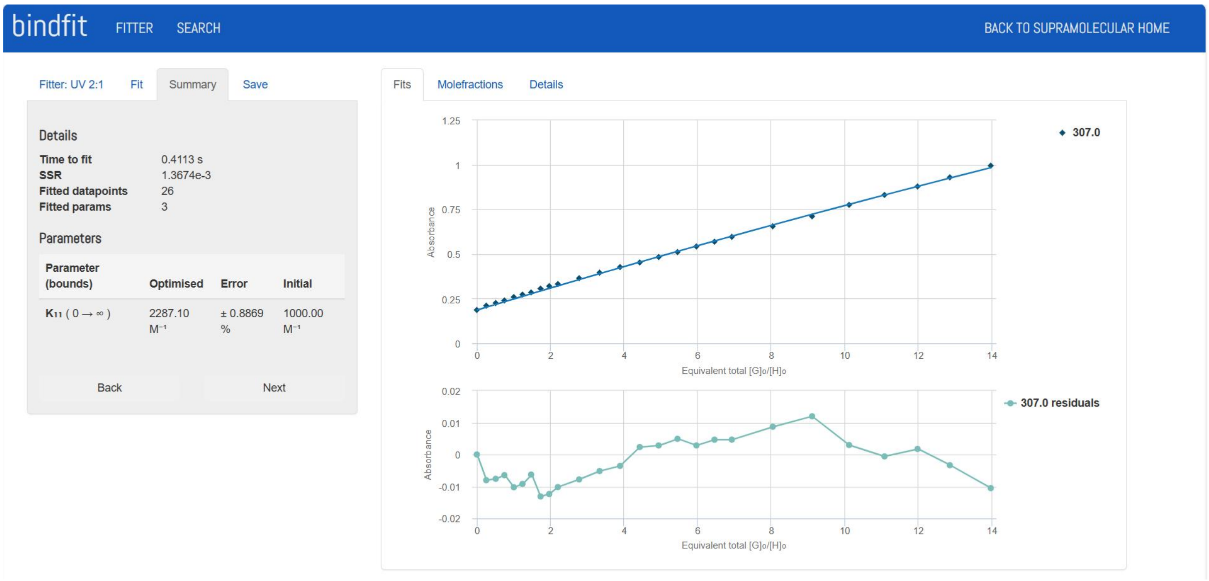

Fig. S40. UV/Vis titration data fitted to a 2:1 binding model. Screenshot of the results window from *http://supramolecular.org*, displaying experimental UV/Vis titration data fitted to a non-cooperative 2:1 binding model. The corresponding dataset is archived at the unique URL: *http://app.supramolecular.org/bindfit/view/3745519b-d0a4-47ba-b9c0-9f9d1a8d9e70*.

Additive 2:1 binding mode (1a:C_{60})

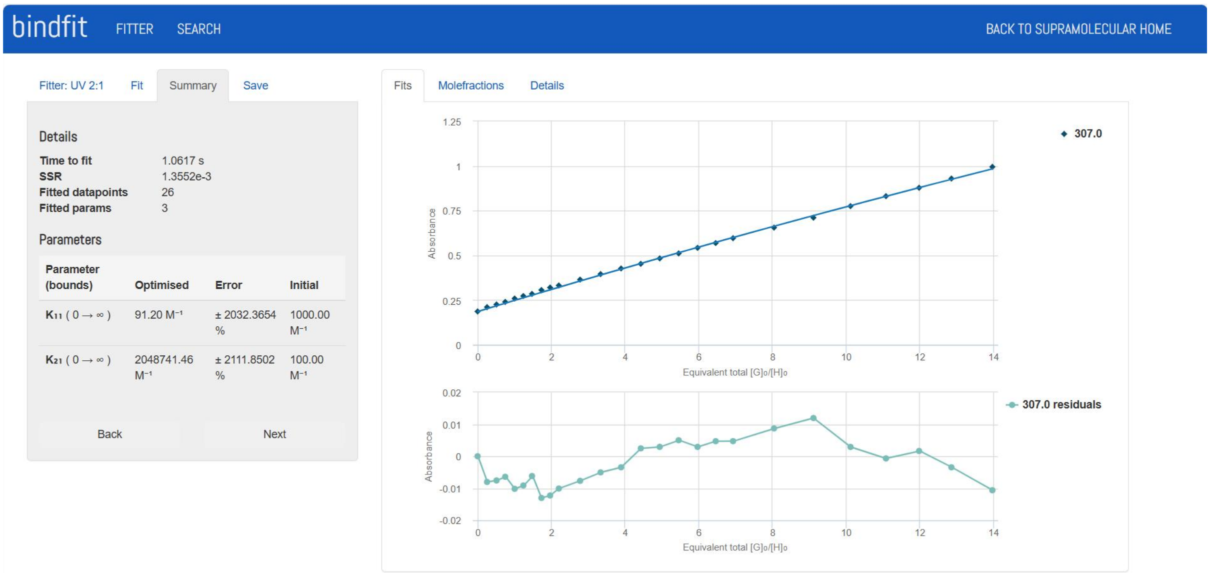

Fig. S41. UV/Vis titration data fitted to a 2:1 binding model. Screenshot of the results window from *http://supramolecular.org*, displaying experimental UV/Vis titration data fitted to an additive 2:1 binding model. The corresponding dataset is archived at the unique URL: *http://app.supramolecular.org/bindfit/view/2dd45b0c-76e2-447b-aa1d-8394f4149b61*.

Statistical 2:1 binding mode (1a:C_{60})

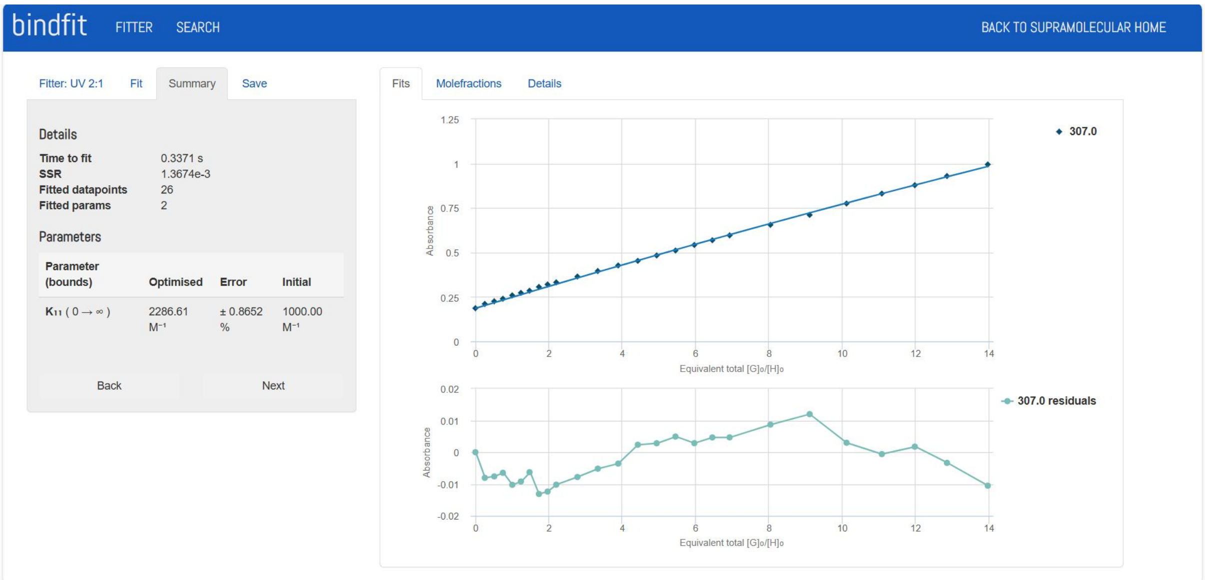

Fig. S42. UV/Vis titration data fitted to a 2:1 binding model. Screenshot of the results window from *http://supramolecular.org*, displaying experimental UV/Vis titration data fitted to a statistical 2:1 binding model. The corresponding dataset is archived at the unique URL: *http://app.supramolecular.org/bindfit/view/607f819f-3ab4-4609-a846-d5b661e691ef*.

Table S9. Comparison of different binding modes for the host 1b towards the guest C_{60} in toluene (the appropriate binding mode is highlighted in bold).

Binding mode	cov_{fit} (10^{-5})	cov_{fit} factor[a]	K_1 [M^{-1}]	K_2 [M^{-1}]
**1:1**	**6.07**	**1.00**	**$(1.59 \pm 0.002) \times 10^3$**	**–**
Full 2:1	5.63	1.08	2.38 ± 0.07	$(1.12 \pm 0.19) \times 10^8$
Non-cooperative 2:1	6.07	1.00	$(1.57 \pm 0.002) \times 10^3$	3.93×10^2 [b]
Additive 2:1	6.00	1.01	282.74 ± 1.99	$(2.13 \pm 2.44) \times 10^5$
Statistical 2:1	6.06	1.00	$(1.57 \pm 0.002) \times 10^3$	3.93×10^2 [b]

[a] The cov_{fit} factor = cov_{fit} for the 1:1 binding model divided by the cov_{fit} for the binding model under study. [b] For the non-cooperative and statistical binding modes, K_2 is calculated as $K_2 = K_1/4$ from the K_1 value obtained.

Conclusion: The cov_{fit} values of the 1:1, full 2:1, non-cooperative 2:1, additive 2:1, and statistical 2:1 binding models are very similar. However, when selecting a binding model, it is justified to choose a more complex model that yields a significantly improved cov_{fit} factor (3-5 fold) compared to a less complex model (*70*). Based on this criterion, we conclude that the 1:1 binding model is the appropriate choice.

1:1 binding mode (1b:C_{60})

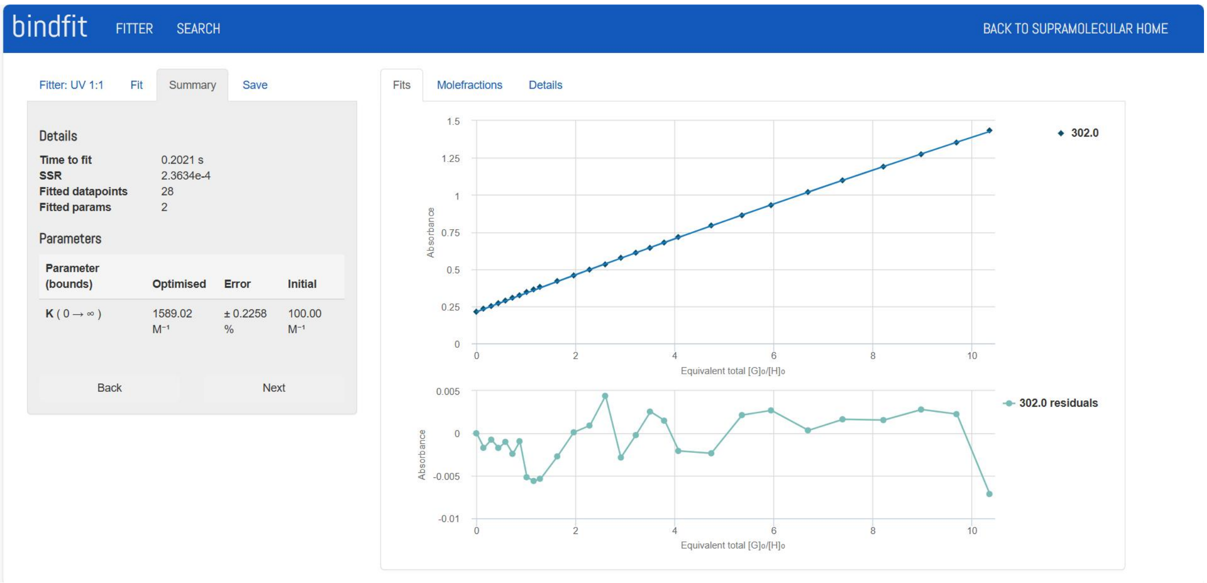

Fig. S43. UV/Vis titration data fitted to a 1:1 binding model. Screenshot of the results window from *http://supramolecular.org*, displaying experimental UV/Vis titration data fitted to a 1:1 binding model. The corresponding dataset is archived at the unique URL: *http://app.supramolecular.org/bindfit/view/c5142a85-4986-44f7-97c3-ffb24ec7355f*.

Full 2:1 binding mode (1b:C_{60})

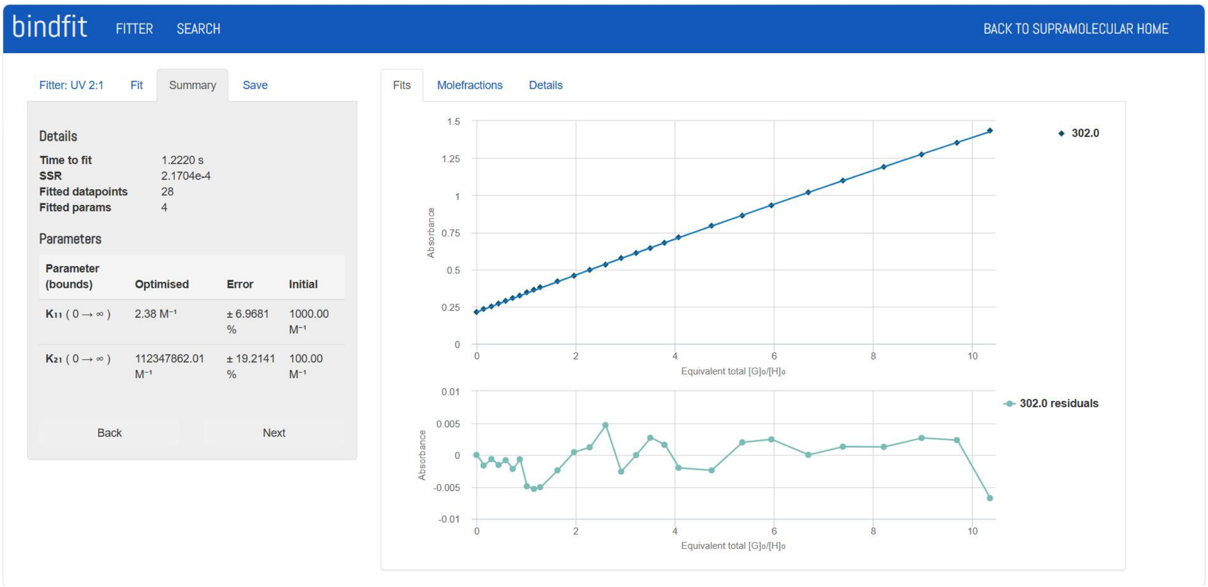

Fig. S44. UV/Vis titration data fitted to a 2:1 binding model. Screenshot of the results window from *http://supramolecular.org*, displaying experimental UV/Vis titration data fitted to a full 2:1 binding model. The corresponding dataset is archived at the unique URL: *http://app.supramolecular.org/bindfit/view/b8b2b6c1-813c-4f40-b14e-a24268f26a64*.

Non-cooperative 2:1 binding mode (1b:C_{60})

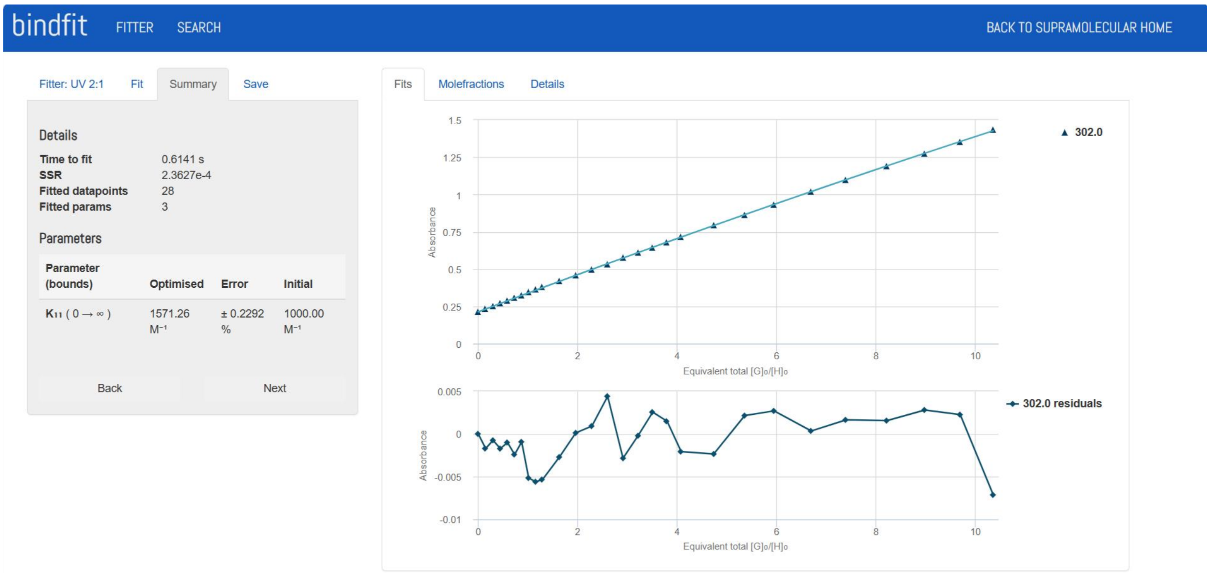

Fig. S45. UV/Vis titration data fitted to a 2:1 binding model. Screenshot of the results window from *http://supramolecular.org*, displaying experimental UV/Vis titration data fitted to a non-cooperative 2:1 binding model. The corresponding dataset is archived at the unique URL: *http://app.supramolecular.org/bindfit/view/bf97c2f0-455f-49c1-b045-267ee4fa7970*.

Additive 2:1 binding mode (1b:C_{60})

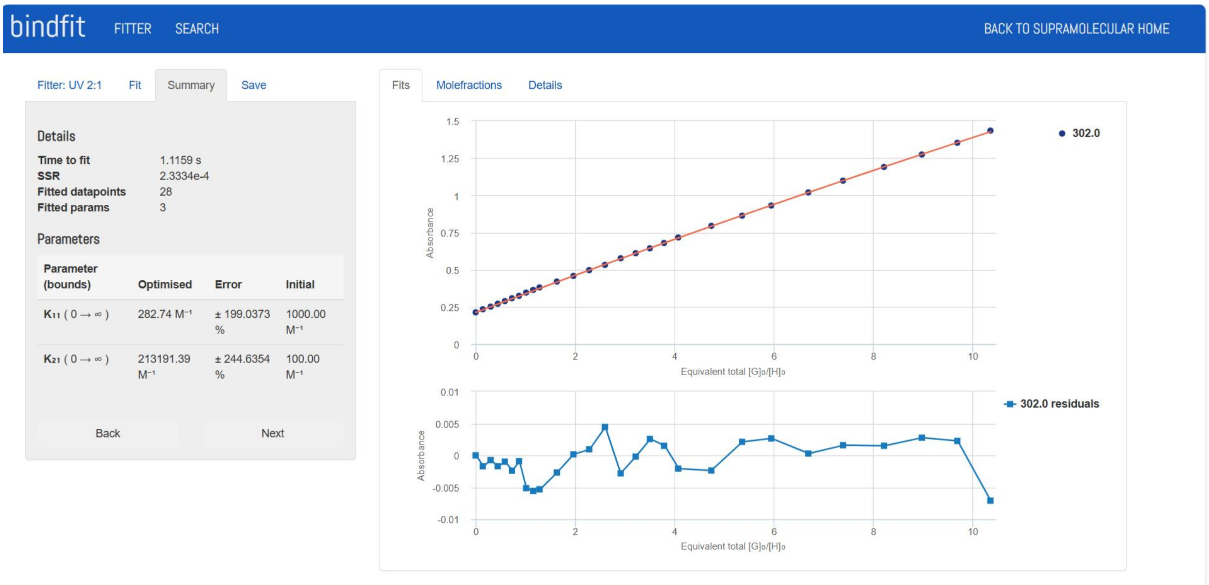

Fig. S46. UV/Vis titration data fitted to a 2:1 binding model. Screenshot of the results window from *http://supramolecular.org*, displaying experimental UV/Vis titration data fitted to an additive 2:1 binding model. The corresponding dataset is archived at the unique URL: *http://app.supramolecular.org/bindfit/view/9bba5ed7-7b6b-40d3-8dd9-0f4686c87e7a*.

Statistical 2:1 binding mode (1b:C_{60})

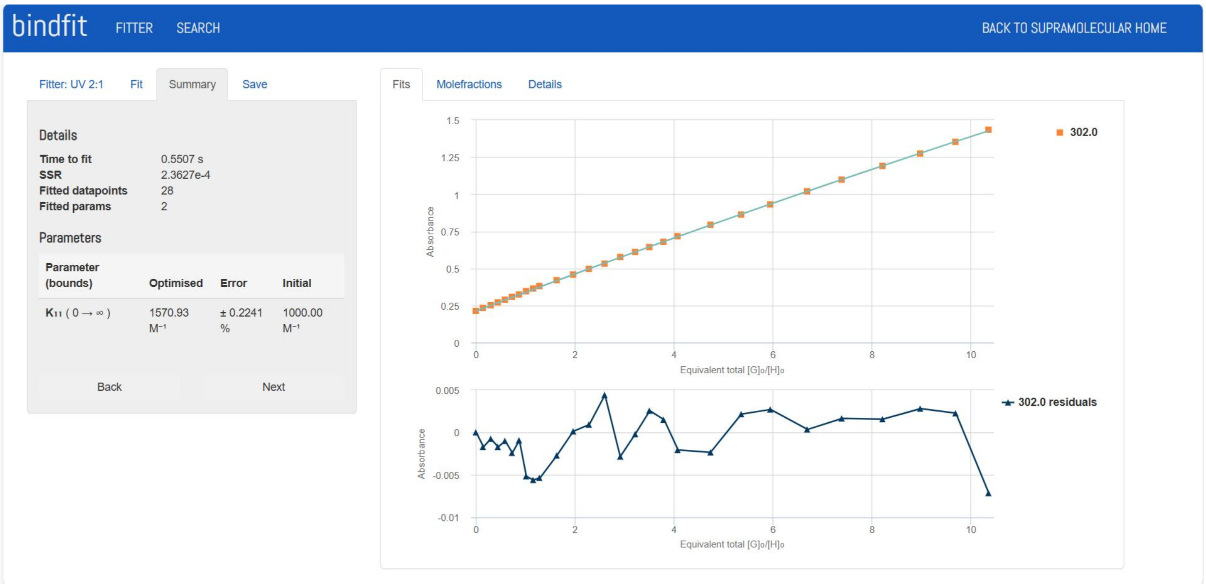

Fig. S47. UV/Vis titration data fitted to a 2:1 binding model. Screenshot of the results window from *http://supramolecular.org*, displaying experimental UV/Vis titration data fitted to a statistical 2:1 binding model. The corresponding dataset is archived at the unique URL: *http://app.supramolecular.org/bindfit/view/3b25e0b0-1bbd-4a29-8d90-c9856b8f7e1a*.

6.2 Job plot analyses by fluorescence spectroscopy

To determine the binding stoichiometry between host **1a** (or **1b**) and guest C_{60} in solution, Job's plot experiments based on fluorescence spectroscopy were carried out in toluene. A solution of guest C_{60} in toluene (1.5×10^{-6} M) and a solution of host in toluene (1.5×10^{-6} M) were mixed in different ratios to prepare 12 samples, while the sum of the concentrations of host and C_{60} was kept constant at 1.5×10^{-6} M. After thorough mixing and equilibration, the UV/Vis spectrum of each sample was recorded. The resulting absorbance data at selected wavelengths were used to construct Job's plots, from which the host–guest stoichiometry was derived.

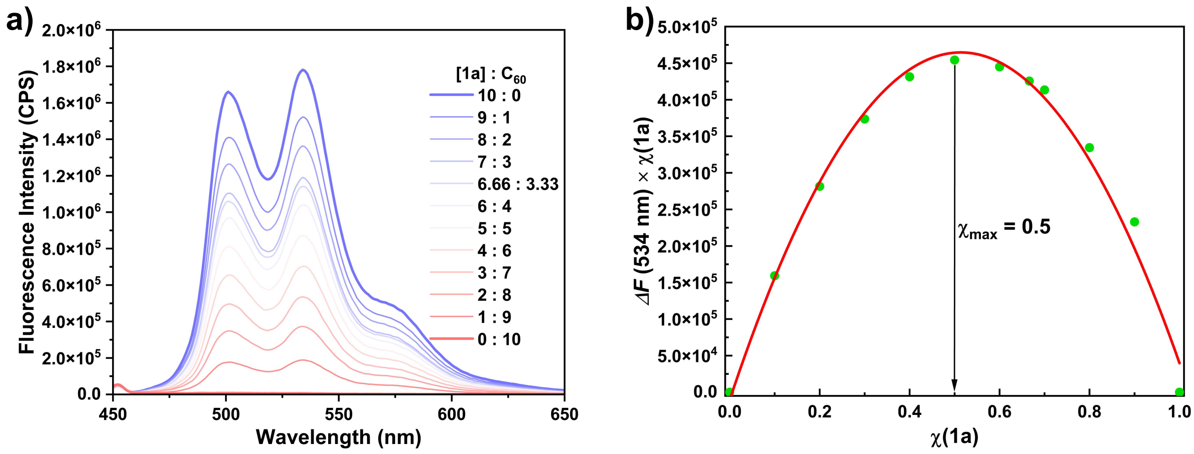

Fig. S48. Job plot analyses for 1a with C_{60} by fluorescence spectroscopy. (a) Fluorescence spectra of **1a** and C_{60} mixed in different ratios in toluene at 298 K. (b) Job's plot based on the emission change at 534 nm, indicating a 1:1 binding stoichiometry between **1a** and C_{60} in toluene.

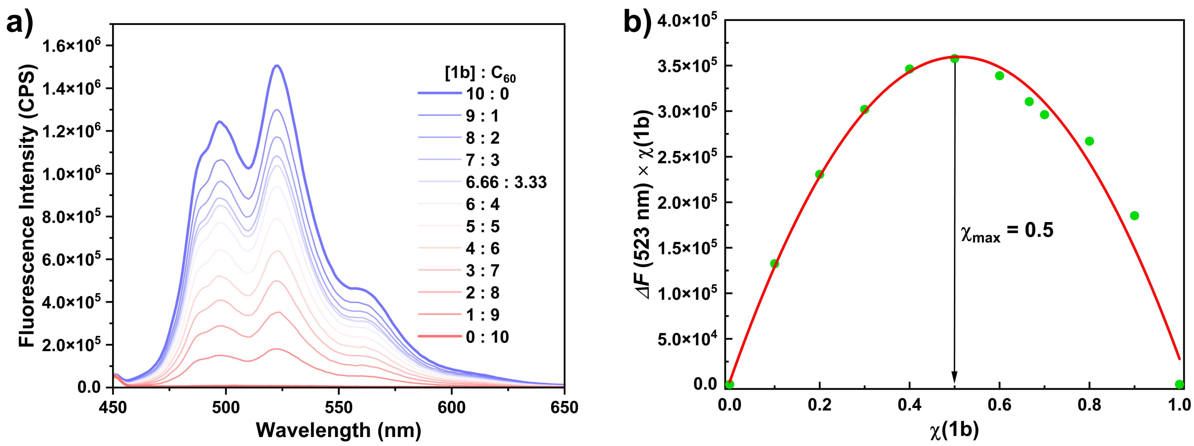

Fig. S49. Job plot analyses for 1b with C_{60} by fluorescence spectroscopy. (a) Fluorescence spectra of **1b** and C_{60} mixed in different ratios in toluene at 298 K. (b) Job's plot based on the emission change at 523 nm, indicating a 1:1 binding stoichiometry between **1b** and C_{60} in toluene.

6.4 Fluorescence titration analyses

Fluorescence titration experiments were carried out in toluene solutions. To maintain a constant host concentration throughout the titration, Stock solutions A and B were prepared as shown in Table S10. The titration was performed by successive additions of solution B into 2.0 mL of solution A. After each addition, the solutions were allowed to equilibrate for at least 3 min. Due to competitive absorption of C_{60} at both excitation and emission wavelengths of the host (Fig. S50), the fluorescence intensity F_{exp} was calibrated to F_{cal} according to a well-established method (*80*):

$$F_{cal} = F_{exp} \times \frac{1 - e^{-\varepsilon_1 c_1 l}}{\varepsilon_1 c_1 l} \times \frac{\varepsilon_1 c_1 l + \varepsilon_2 c_2 l}{1 - e^{(-\varepsilon_1 c_1 l - \varepsilon_2 c_2 l)}} \times \frac{\varepsilon_3 c_3 l}{1 - e^{-\varepsilon_3 c_3 l}}$$

where $\varepsilon_1 c_1 l$ represents the absorbance of the host at the excitation wavelength (λ_{ex} = 400 nm), $\varepsilon_2 c_2 l$ represents the absorbance of C_{60} at the excitation wavelength, and $\varepsilon_3 c_3 l$ represents the absorbance of C_{60} at the emission wavelength (λ_{em} = 534 nm for **1a**; λ_{em} = 523 nm for **1b**).

Table S10. Concentrations of stock solutions for fluorescence titration experiments in toluene.

Host molecule	C_{60}		
	Solution A	Solution B	
	[host] µM	[host] µM	[C_{60}] µM
**1a**	1.5	1.5	150
**1b**	1.5	1.5	150

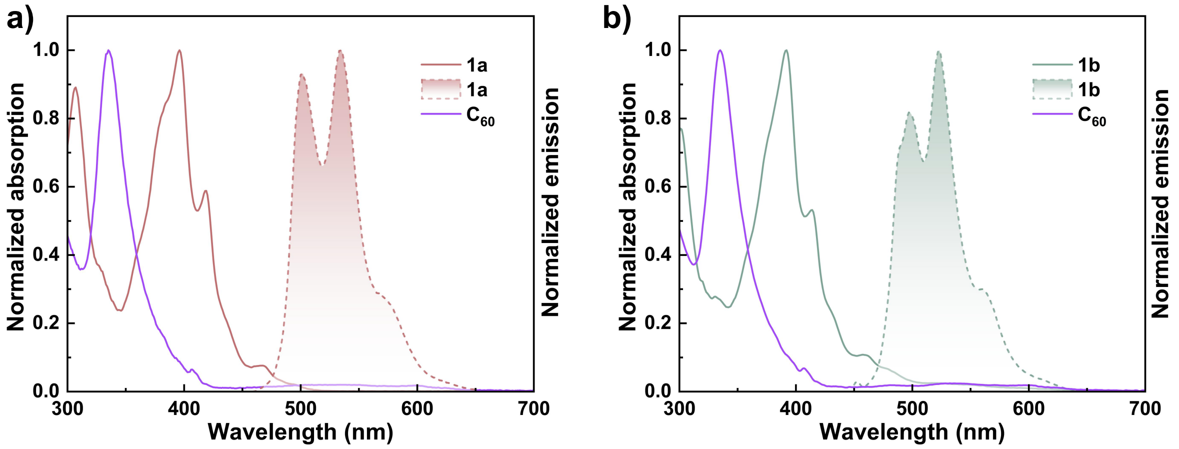

Fig. S50. Spectral comparison of 1a, 1b, and C_{60} in toluene. Normalized UV/Vis absorption spectrum (solid line) and fluorescence emission spectrum (dashed line) of (a) **1a** and (b) **1b**, with the normalized UV/Vis absorption spectrum of C_{60} for comparison.

Based on the 1:1 complex model, association constant K_a is calculated by non-linear curve fitting (1stopt software with universal global optimization) using the following equation (*72*):

$$\frac{F_0}{F_{cal}} = \frac{2}{1 - \frac{1}{[H]_0}\left([G]_0 + \frac{1}{K_a} - \sqrt{\left([H]_0 + [G]_0 + \frac{1}{K_a}\right)^2 - 4[H]_0[G]_0}\right)}$$

where F_0, F_{cal}, H_0, G_0, and K_a are fluorescence intensity of the **1a** (λ_{em} = 534 nm) or **1b** (λ_{em} = 523 nm) before the addition of C_{60}, fluorescence intensity after calibration, initial concentration of **1a** or **1b**, initial concentration of C_{60}, and the association constant respectively.

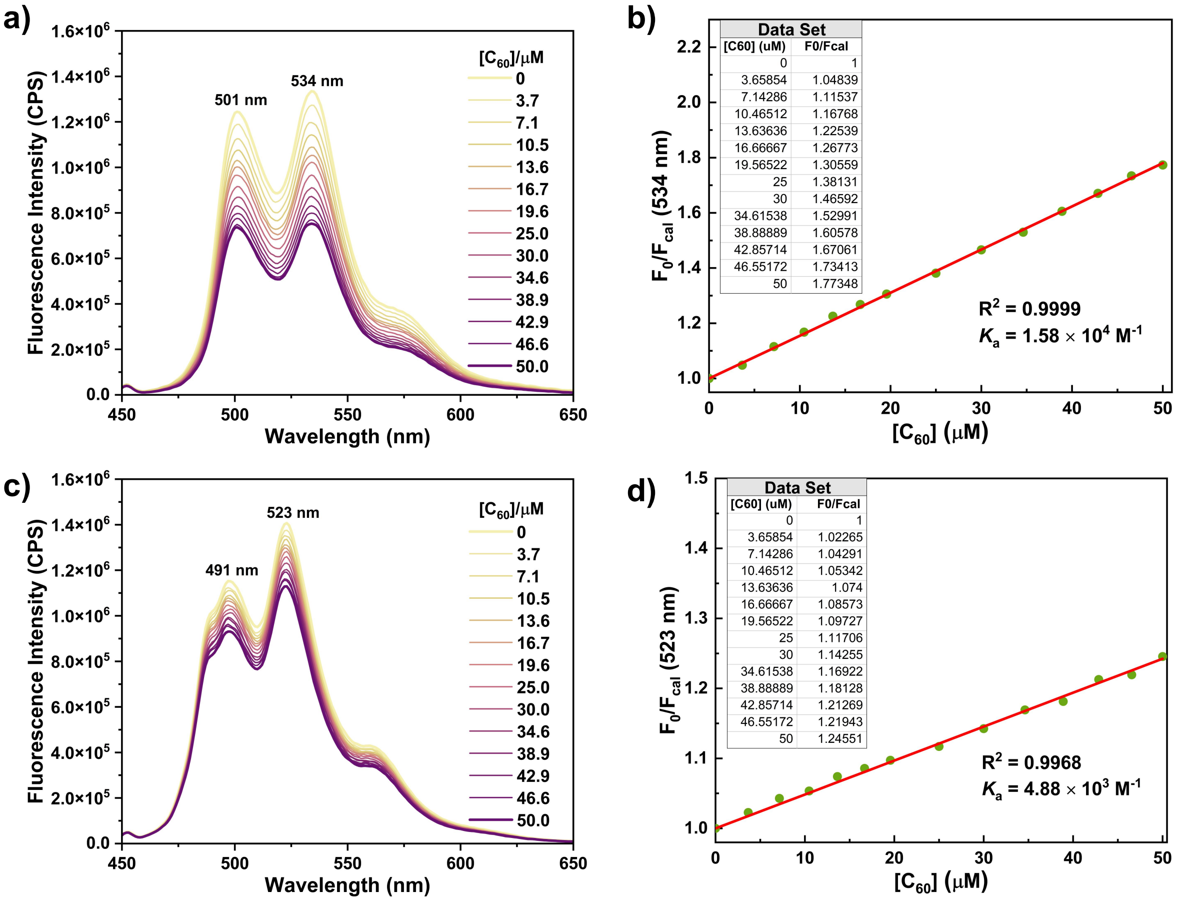

Fig. S51. Fluorescence titration of 1a and 1b with C_{60}. (a) Spectral changes of **1a** (1.5 μM) upon addition of C_{60}. (b) Nonlinear curve fitting for the binding of **1a** with C_{60}. (c) Spectral changes of **1b** (1.5 μM) upon addition of C_{60}. (d) Nonlinear curve fitting for the binding of **1b** with C_{60}.

Table S11. Association constants (K_a) for 1a and 1b with C_{60} in toluene.

Complex	Stoichiometry (H:G)	K_a (M^{-1}) UV/Vis titration	K_a (M^{-1}) fluorescence quenching
**1a@C_{60}**	1:1	2.31×10^3	1.58×10^4
**1b@C_{60}**	1:1	1.59×10^3	4.88×10^3

Conclusion: The association constants derived from fluorescence quenching are significantly larger than those from UV/Vis titration for both complexes. This discrepancy is attributed to the concurrent dynamic quenching caused by collisional processes and the inner filter effect (IFE) of C_{60}, which collectively inflate the observed fluorescence quenching efficiency.

6.5 X-ray crystallography for 1a@C_{60}

Single crystals of compound **1a@C_{60}**, suitable for X-ray structural determination, were obtained as black-colored blocks by slow diffusion of *n*-hexane vapor into a solution of equimolar mixtures of **1a** and C_{60} in benzene.

Table S12. Crystallographic data and structure refinement details for 1a@C_{60} (CCDC: 2444407).

Empirical formula	$C_{165}H_{48}F_{18}$
Formula weight	2372.03
Temperature/K	200.00
Crystal system	orthorhombic
Space group	Pbca
a/Å	18.7580(4)
b/Å	25.9963(6)
c/Å	54.2471(13)
α/°	90
β/°	90
γ/°	90
Volume/Å^3	26453.0(10)
Z	8
ρ_{calc}g/cm^3	1.191
μ/mm^{-1}	0.721
F(000)	9600.0
Crystal size/mm^3	0.3 × 0.2 × 0.1
Radiation	CuKα (λ = 1.54178)
2Θ range for data collection/°	6.034 to 136.61
Index ranges	$-22 \leq h \leq 22$, $-31 \leq k \leq 31$, $-65 \leq l \leq 65$
Reflections collected	362493
Independent reflections	24118 [R_{int} = 0.0552, R_{sigma} = 0.0215]
Data/restraints/parameters	24118/48742/2189
Goodness-of-fit on F^2	1.925
Final R indexes [I>=2σ (I)]	R_1 = 0.1211, wR_2 = 0.3991
Final R indexes [all data]	R_1 = 0.1273, wR_2 = 0.4084
Largest diff. peak/hole / e Å^{-3}	1.01/-0.76

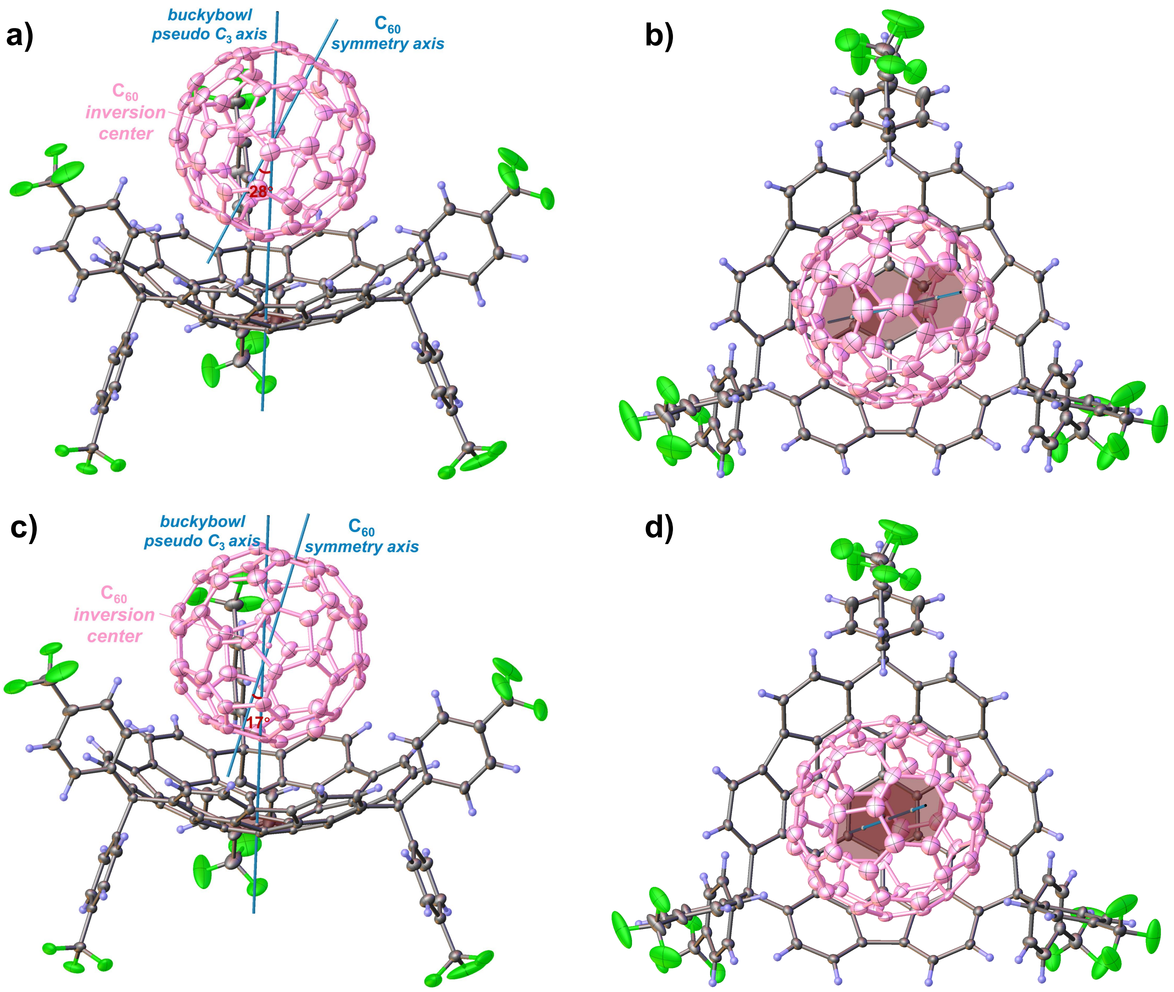

Fig. S52. Crystal structures of 1a@C_{60}. (a and c) Side views. (b and d) Top views. Thermal ellipsoids are shown at 30% probability. Solvent molecules are omitted for clarity. The C_{60} molecule shows rotational disorder modeled with two orientations (occupancies 0.564:0.436).

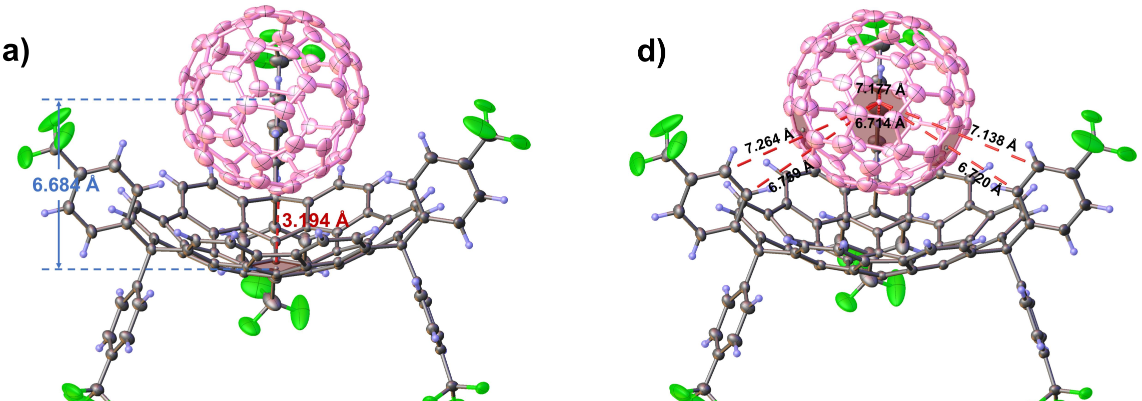

Fig. S53. Interactions in 1a@C_{60} crystal. (a) π···π interactions between **1a** and C_{60}. (b) C-H···π interactions between **1a** and C_{60}. Major C_{60} disorder component (0.564 occupancy) shown.

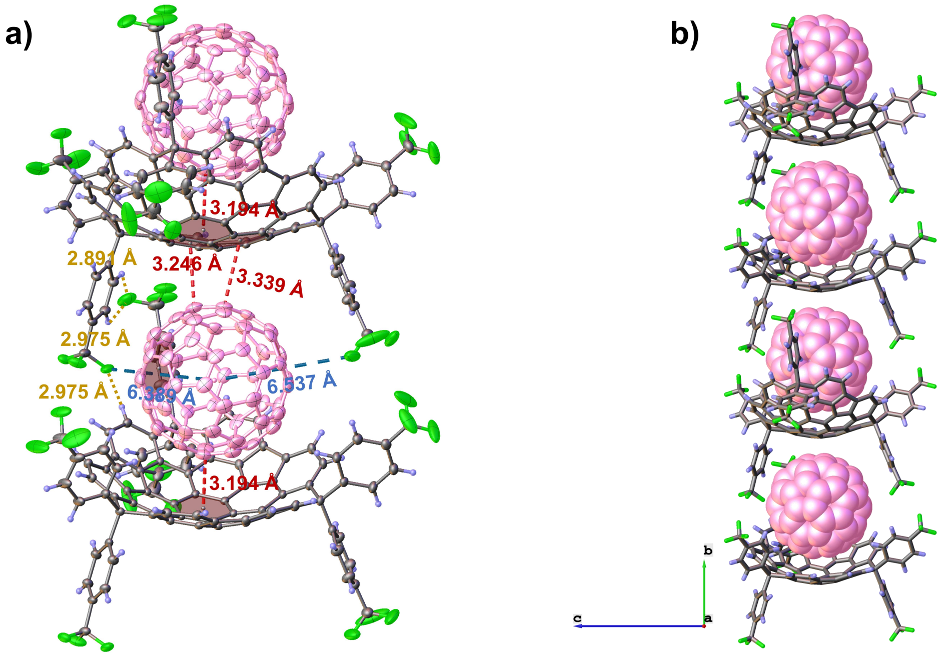

Fig. S54. Supramolecular assembly of 1a@C$_{60}$. (a) Adjacent complex units stabilized by π-π interactions (red), F···π interactions (blue), and C–F···H hydrogen bonds (yellow). (b) Continuous staggered columnar stacking.

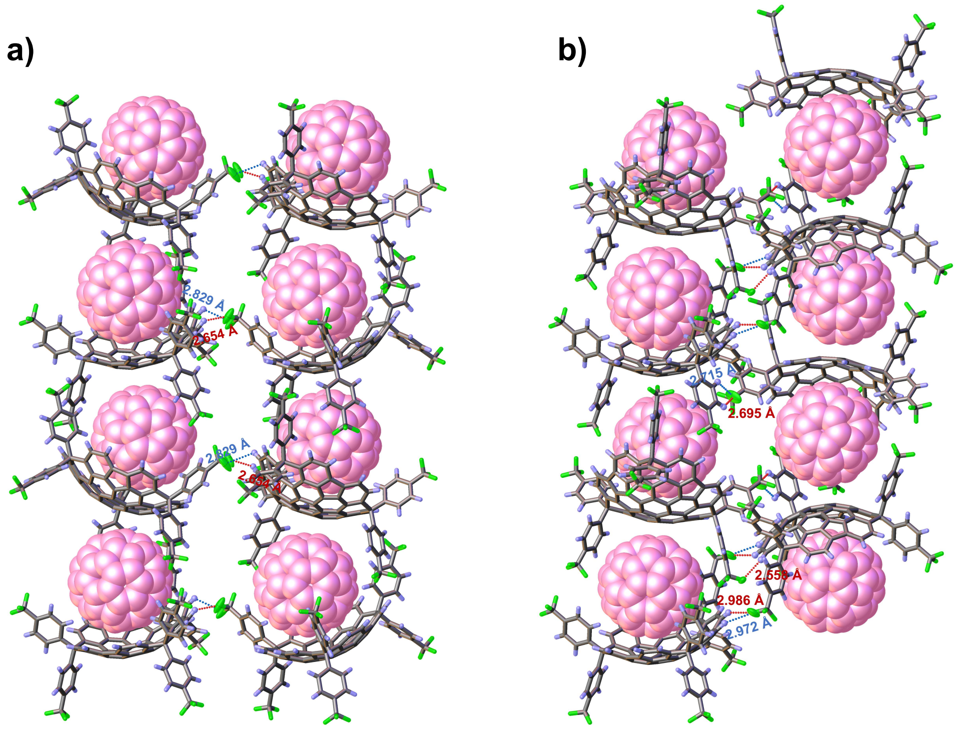

Fig. S55. Intercolumnar interactions in 1a@C$_{60}$. (a) Column pairs with the same orientation stabilized by C–F···H hydrogen bonds. (b) Centrosymmetric column pairs with opposite orientation.

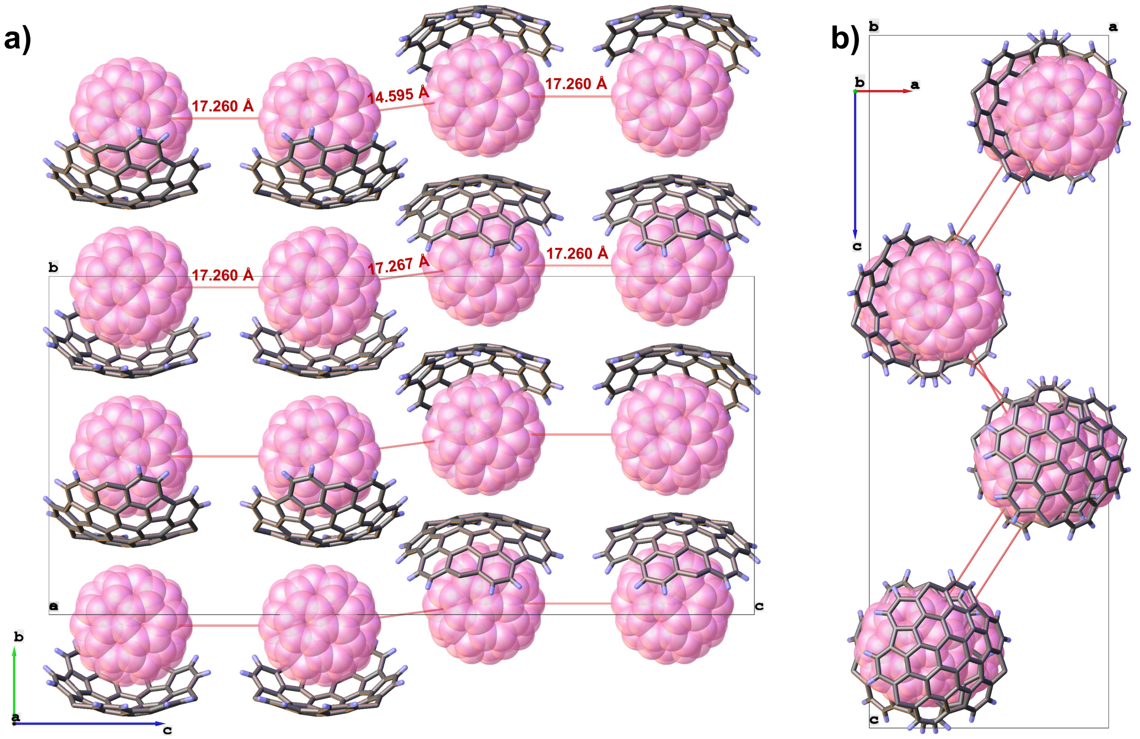

Fig. S56. 2D zigzag packing of 1a@C_{60}. The staggered arrangement with C_{60} center-to-center distances (red lines). Views along (a) a-axis and (b) b-axis. The aryl groups were omitted for clarity.

6.6 X-ray crystallography for (1b)$_2$@C_{60}

Single crystals of compound **(1b)$_2$@C_{60}**, suitable for X-ray structural determination, were obtained as black-colored blocks by slow diffusion of *n*-hexane vapor into a solution of equimolar mixtures of **1b** and C_{60} in toluene.

Table S13. Crystallographic data and structure refinement details for (1b)$_2$@C_{60} (CCDC: 2444408)

Empirical formula	$C_{186}H_{84}$
Formula weight	2318.53
Temperature/K	244.00
Crystal system	cubic
Space group	Pa-3
a/Å	21.8964(4)
b/Å	21.8964(4)
c/Å	21.8964(4)
α/°	90
β/°	90
γ/°	90
Volume/Å^3	10498.3(6)
Z	4
ρ_{calc}g/cm^3	1.467
μ/mm^{-1}	0.639
F(000)	4800.0
Crystal size/mm^3	0.2 × 0.2 × 0.2
Radiation	CuKα (λ = 1.54178)
2Θ range for data collection/°	8.076 to 136.912
Index ranges	$-26 \leq h \leq 26$, $-26 \leq k \leq 26$, $-26 \leq l \leq 26$
Reflections collected	293679
Independent reflections	3230 [R_{int} = 0.0607, R_{sigma} = 0.0205]
Data/restraints/parameters	3230/35/282
Goodness-of-fit on F^2	1.095
Final R indexes [I>=2σ (I)]	R_1 = 0.0673, wR_2 = 0.1805
Final R indexes [all data]	R_1 = 0.0746, wR_2 = 0.1861
Largest diff. peak/hole / e Å^{-3}	0.95/-0.44

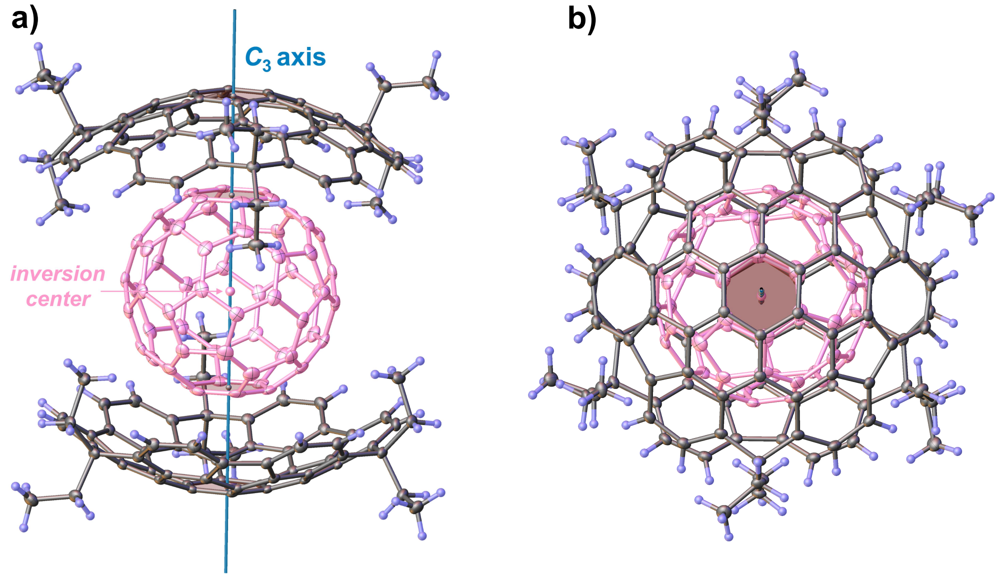

Fig. S57. Crystal structures of (1b)$_2$@C$_{60}$. (a) Side view and (b) top view. Thermal ellipsoids are shown at 30% probability.

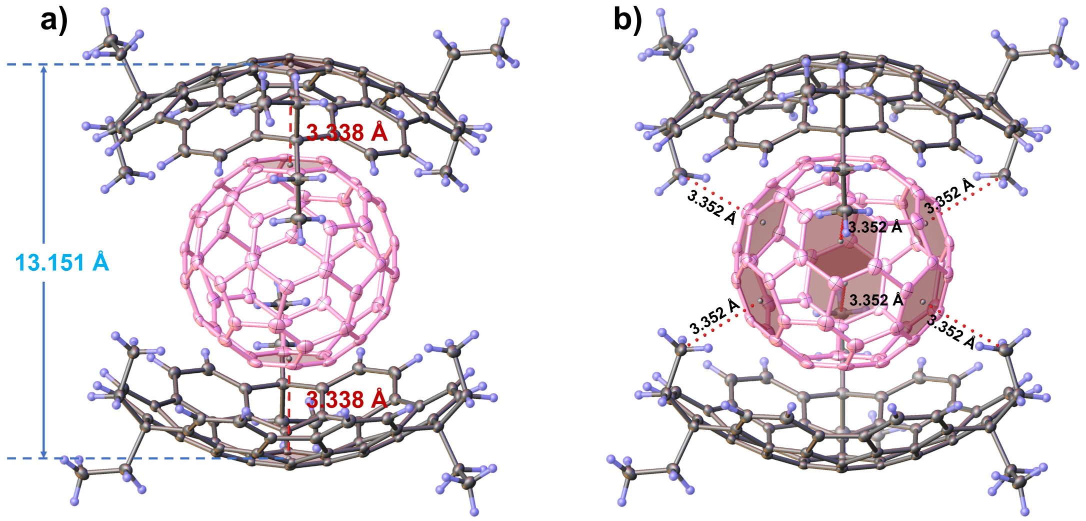

Fig. S58. Interactions in (1b)$_2$@C$_{60}$ crystal. (a) π···π and (b) C-H···π interactions between **1b** and C$_{60}$.

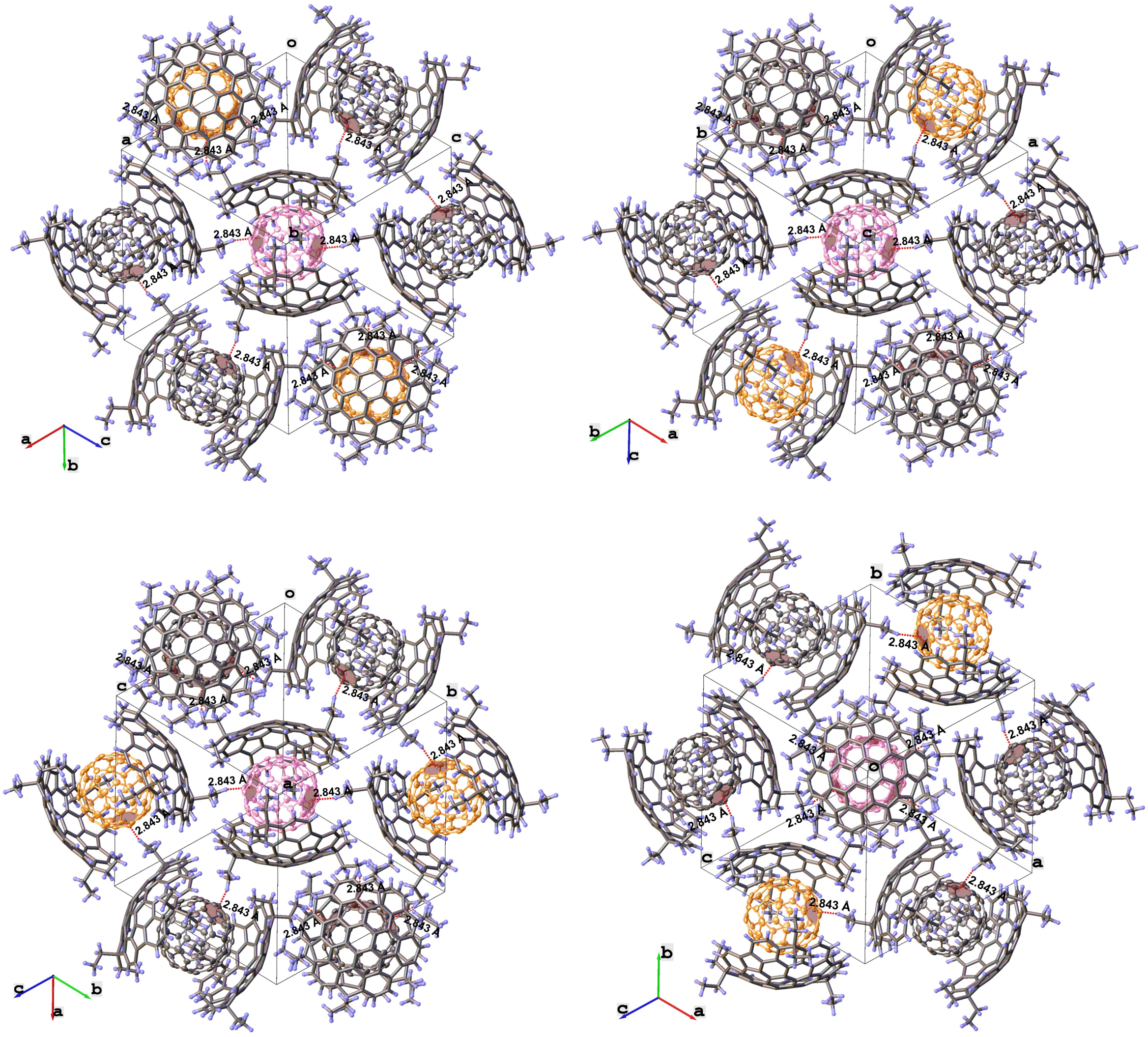

Fig. S59. 2D hexagonal honeycomb sheet packing structure arranged in multiple orientations.

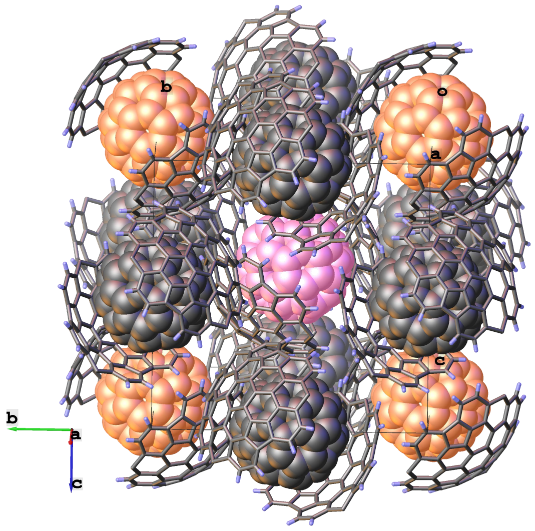

Fig. S60. Unit cell of $(1b)_2@C_{60}$ with ethyl groups omitted. One crystallographically independent molecule type with four complete complex molecules per unit cell.

7. DFT calculations

Geometric optimization was performed at M062x/6-31G(d,p) or B3LYP-D3(BJ)/6-31+G(d,p) level of theory using the Gaussian 16, Revision A.03 package (*76*), and some calculated results were processed by Multiwfn (*66*). Rotation and inversion barrier calculations were conducted at the M062x/6-31G(d,p) level. NICS(1)$_{zz}$ (*61*) were calculated using the gauge invariant atomic orbital (GIAO) approach at the GIAO-B3LYP/6-311G(d,p) level. Due to the curved structures of target bowls and reference compound, we used the average of NICS(1)$_{zz}$ (concave) and NICS(-1)$_{zz}$ (convex) to evaluate the aromaticity character of individual rings. AICD plot (*62*) was calculated at B3LYP/6-311G(d,p) level. Strength of the current density (nA/T) passing selected chemical bonds calculated using the GIMIC (*63*) program (B3LYP/6-311G(d,p)). TD-DFT calculations were conducted at B3LYP/6-311G(d,p) level. An independent gradient model based on the Hirshfeld partition (IGMH) (*67*) was calculated at B3LYP-D3(BJ)/6-31+G(d,p) level to visualize noncovalent interaction regions. Energy decomposition analysis was carried out by utilizing the sobEDAw method (*68*) at the B3LYP-D3(BJ)/6-31+G(d,p) level to assess the intermolecular interaction.

7.1 Optimized structures and calculated HOMO-LUMO energy gaps

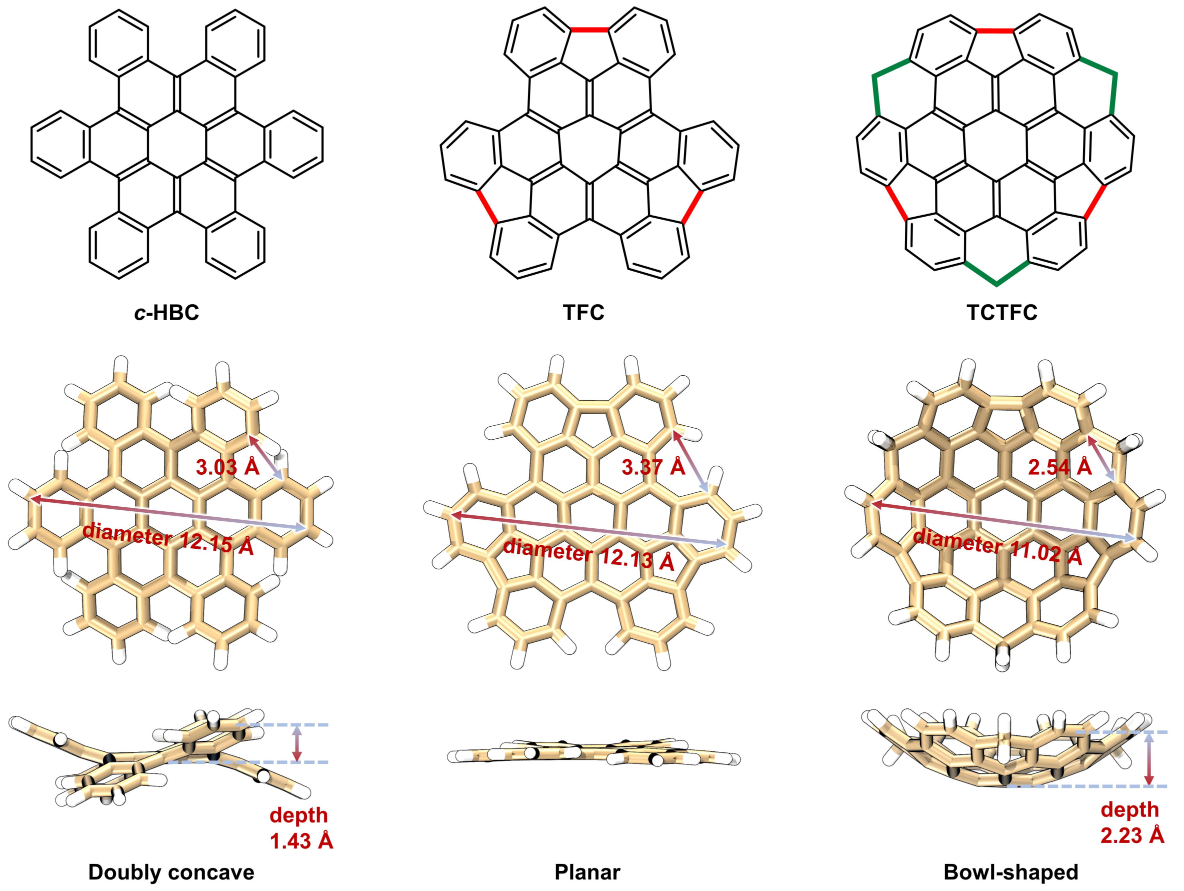

Fig. S61. Optimized geometry of *c*-HBC, TFC, and TCTFC (1-H) (M062x/6-31G(d,p)).

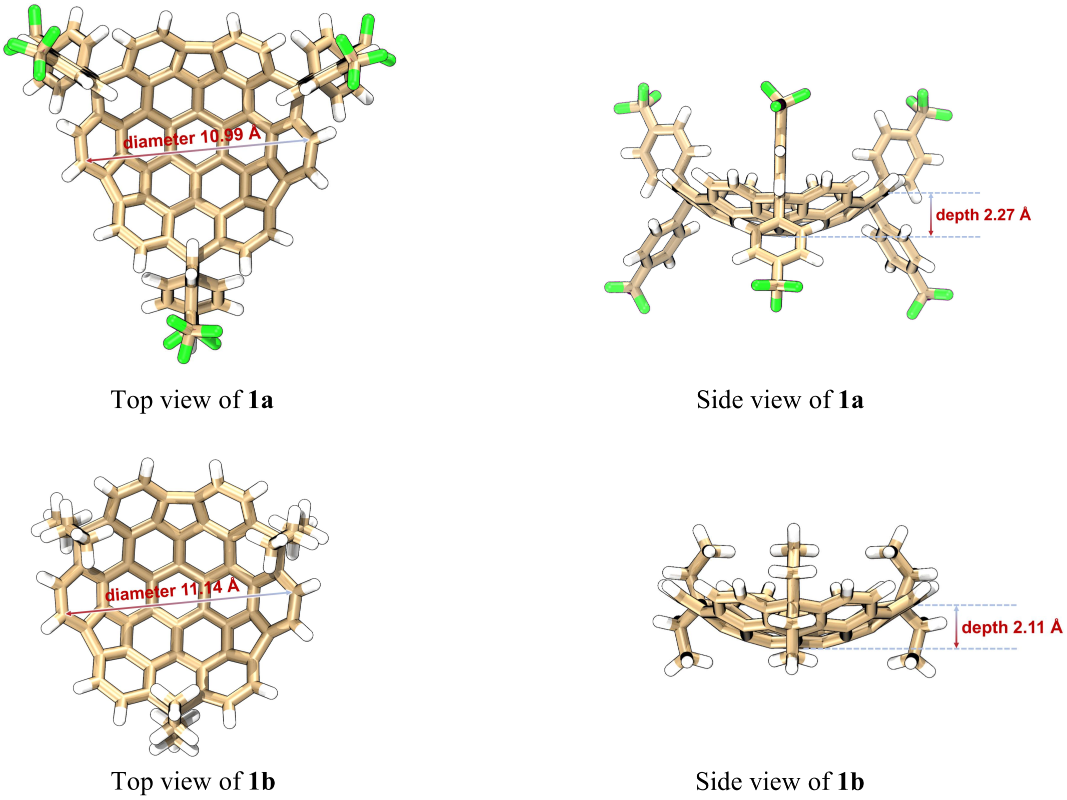

Fig. S62. Optimized geometry of 1a and 1b (M062x/6-31G(d,p)).

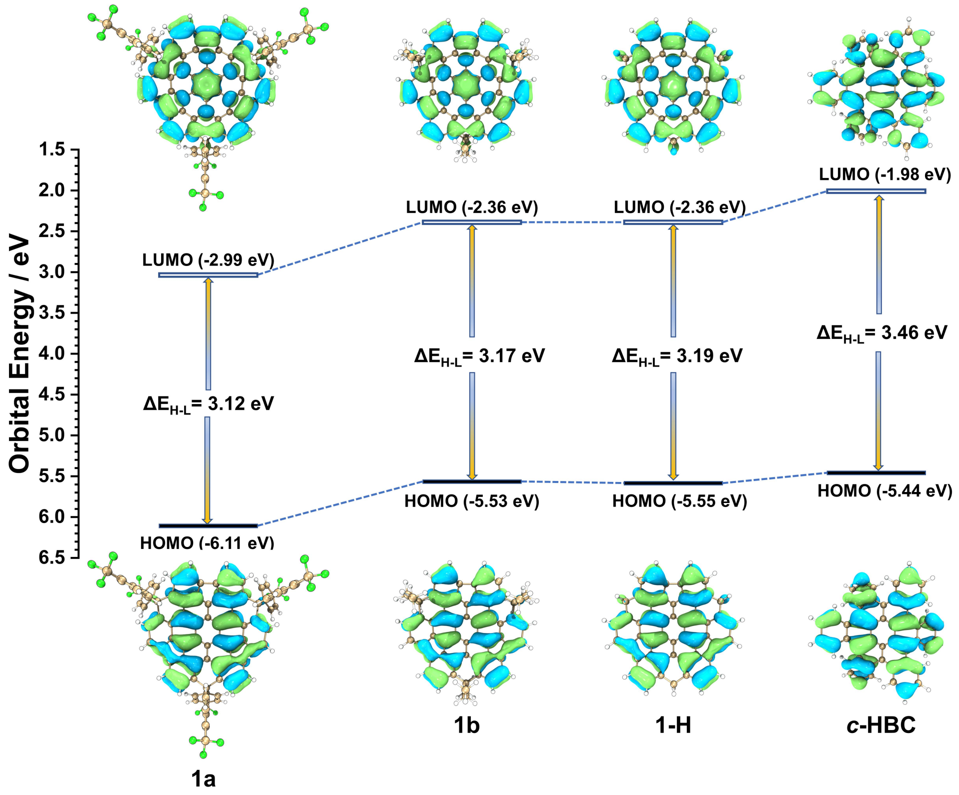

Fig. S63. Frontier molecular orbital correlation diagrams and HOMO-LUMO gaps. Comparison for **1a**, **1b**, **1-H** and *c*-HBC (B3LYP/6-311g(d,p)).

7.2 Rotation and bowl-to-bowl inversion barriers

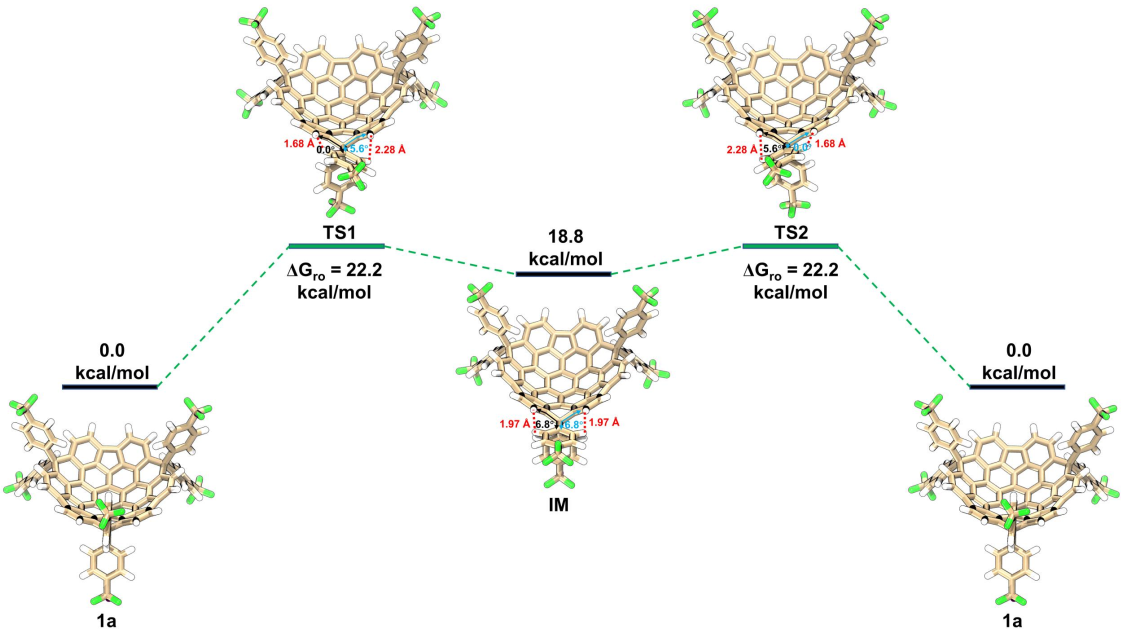

Fig. S64. Calculated rotation process and rotation barrier of the concave aryl groups in 1a (M062x/6-31G(d,p)).

Table S14. Calculated total energies (M062x/6-31G(d,p)) for 1a.

Parameter	Ground state (*GS*) (Hartree)	Intermediate (*IM*) (Hartree)	Transition state (*TS1&TS2*) (Hartree)
Zero-point correction	1.043283	1.043837	1.043461
Thermal correction to Energy	1.121142	1.121489	1.119460
Thermal correction to Enthalpy	1.122086	1.122433	1.120404
**Thermal correction to Gibbs Free Energy**	**0.915278**	**0.917588**	**0.921988**
Sum of electronic and zero-point Energies	-5360.025310	-5359.997127	-5359.996537
Sum of electronic and thermal Energies	-5359.947450	-5359.919475	-5359.920538
Sum of electronic and thermal Enthalpies	-5359.946506	-5359.918531	-5359.919593
Sum of electronic and thermal Free Energies	-5360.153314	-5360.123377	-5360.118010
**Single point energy**	**-5361.068592**	**-5361.040964**	**-5361.039998**

Table S15. Calculated rotation barrier (ΔG_{ro}) in 1a.

Compd	G(*GS*) (Hartree)	G(*IM*) (Hartree)	G(*TS1&TS2*) (Hartree)	ΔG (*IM* - *GS*) (kcal mol^{-1})	ΔG_{ro} (*TS* - *GS*) (kcal mol^{-1})
**1a**	-5,360.153314	-5,360.123376	-5,360.118010	18.8	22.2

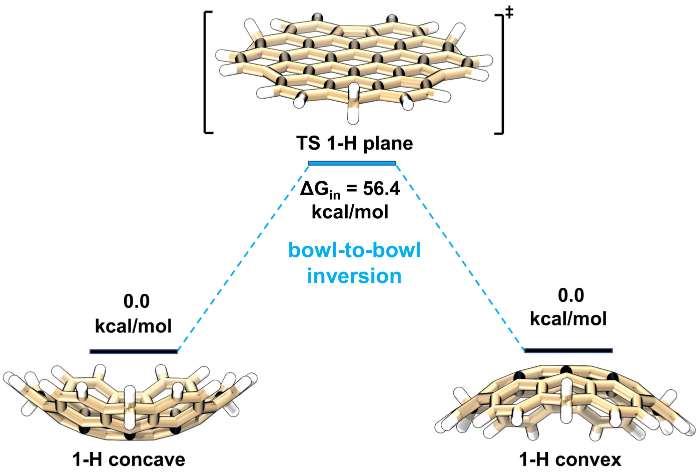

Fig. S65. Calculated bowl-to-bowl inversion process and inversion barrier of 1-H at the M062x/6-31G(d,p) level.

Table S16. Calculated total energies (M062x/6-31G(d,p)) for 1-H.

Parameter	Ground state (*GS*) (Hartree)	Transition state (*TS*) (Hartree)
Zero-point correction	0.522367	0.519502
Thermal correction to Energy	0.549647	0.547288
Thermal correction to Enthalpy	0.550591	0.548232
**Thermal correction to Gibbs Free Energy**	**0.470327**	**0.465278**
Sum of electronic and zero-point Energies	-1953.175262	-1953.083135
Sum of electronic and thermal Energies	-1953.147983	-1953.055349
Sum of electronic and thermal Enthalpies	-1953.147039	-1953.054404
Sum of electronic and thermal Free Energies	-1953.227302	-1953.137359
**Single point energy**	**-1953.697630**	**-1953.602637**

Table S17. Calculated inversion barrier (ΔG_{in}) in 1-H.

**Compd**	**G (*GS*) (Hartree)**	**G (*TS*) (Hartree)**	**ΔG_{in} (*TS* - *GS*) (Hartree)**	**ΔG_{in} (*TS* - *GS*) (kcal mol^{-1})**
**1-H**	-1,953.227303	-1,953.137359	0.089944	56.4

7.3 Aromaticity calculations

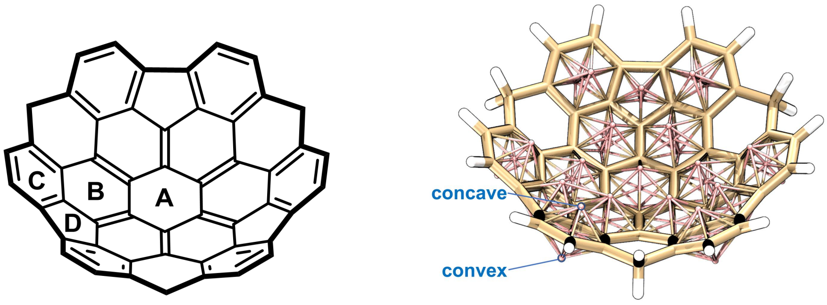

Fig. S66. The input file geometry of 1-H for NICS calculations.

Table S18. NICS calculation on 1-H (GIAO-B3LYP/6-311G(d,p)).

	$NICS_{iso}$			$NICS_{zz}$			
	NICS(0)	$NICS(1)_{convex}$	$NICS(1)_{concave}$	NICS(0)	$NICS(1)_{convex}$	$NICS(1)_{concave}$	$NICS(1)_{av}$
**A**	4.84	3.62	-4.78	37.72	9.49	13.11	**11.30**
**B**	-5.31	-11.94	-4.25	7.65	-8.47	-16.07	**-12.27**
**C**	-6.24	-6.24	-10.91	-1.02	-16.09	-20.49	**-18.29**
**D**	6.75	3.19	-2.42	47.09	13.59	14.71	**14.15**

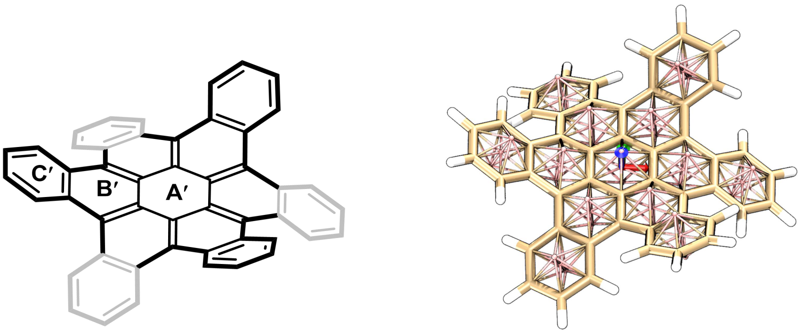

Fig. S67. The input file geometry of *c*-HBC for NICS calculations.

Table S19. NICS calculation on *c*-HBC (GIAO-B3LYP/6-311G(d,p)).

	$NICS_{iso}$			$NICS_{zz}$			
	NICS(0)	$NICS(1)_{convex}$	$NICS(1)_{concave}$	NICS(0)	NICS(1)	NICS(-1)	$NICS(1)_{av}$
**A'**	5.08	0.11	0.11	37.58	10.40	10.40	**10.4**
**B'**	-4.16	-6.95	-8.45	4.82	-14.45	-16.09	**-15.27**
**C'**	-8.46	-8.77	-12.60	-9.90	-22.90	-29.66	**-26.28**

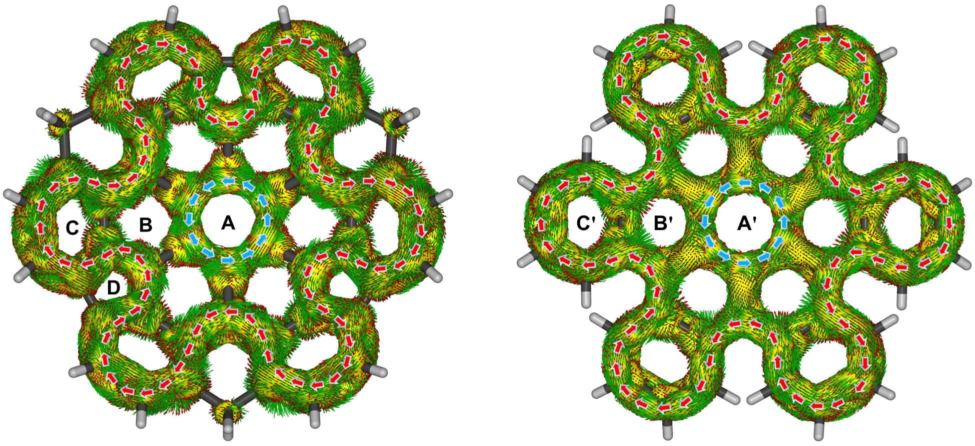

1-H **c-HBC**

Fig. S68. Calculated AICD (B3LYP/6-311G(d,p)) plots of 1-H (left) and *c*-HBC (right) (isovalue = 0.03). Only contributions from π-electrons of the aromatic cores are considered. The magnetic field vector is perpendicular to the ring plane and points outward. Red and blue arrows indicate the diatropic and paratropic ring currents, respectively.

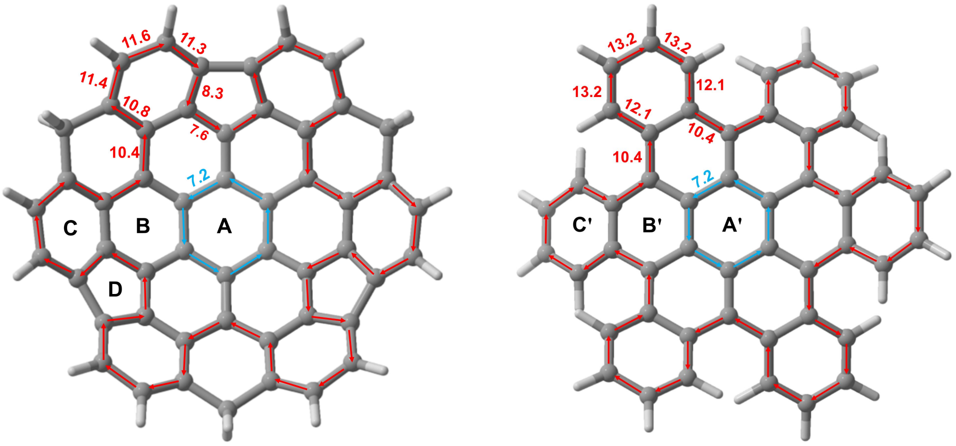

1-H ***c*-HBC**

Fig. S69. Strength of the current density (nA/T) of 1-H (left) and *c*-HBC (right) calculated using the GIMIC program (B3LYP/6-311G(d,p)). Red and blue arrows indicate the diatropic and paratropic ring currents, respectively.

7.4 UV-Vis absorption spectra calculations

Table S20. Major transitions corresponding to the UV spectrum of 1-H calculated by TD-DFT (B3LYP/6-311G(d,p)).

Excited state	Energy (eV)	Wavelength (nm)	Description	μ_{tr} (a.u.)			Oscillator strength (a.u.)
				X	Y	Z	
S_1	2.7581	449.53	H→L (95.7%)	0.0000	-0.0490	-0.0001	0.0002
S_2	2.7583	449.50	H-1→L (95.7%)	0.0439	0.0000	0.0000	0.0001
S_3	2.8015	442.56	H→L+1 (48.3%)<br>H-1→L+2 (48.3%)	0.0009	0.0000	0.0000	0.0000
S_4	2.8688	432.18	H→L+2 (49.0%)<br>H-1→L+1 (49.0%)	0.0000	0.0005	-0.0195	0.0000
S_5	3.0749	403.21	H-1→L+1 (36.9%)<br>H→L+2 (36.9%)<br>H-3→L (17.9%)	0.0000	-2.3695	-0.0005	0.4230
S_6	3.0750	403.20	H-1→L+2 (37.1%)<br>H→L+1 (37.0%)<br>H-4→L (17.8%)	2.3746	0.0000	0.0000	0.4248
S_7	3.2077	386.52	H-3→L (60.0%)<br>H→L+2 (11.2%)<br>H-1→L+1 (11.2%)<br>H-3→L+2 (5.6%)<br>H-4→L+1 (5.6%)	0.0000	2.0744	0.0000	0.3382
S_8	3.2079	386.50	H-4→L (60.2%)<br>H→L+1 (11.2%)<br>H-1→L+2 (11.0%)<br>H-3→L+1 (5.9%)<br>H-4→L+1 (5.1%)	-2.0686	0.0000	0.0000	0.3363
S_{27}	4.1507	298.71	H-1→L+6 (31.4%)<br>H→L+5 (31.0%)<br>H-7→L (22.8%)	1.5873	0.0000	0.0000	0.2562
S_{28}	4.1507	298.71	H→L+6 (40.8%)<br>H-6→L (22.3%)<br>H-1→L+5 (21.7%)	0.0000	1.5721	0.0070	0.2513

S_{36}	4.4507	278.57	H-2→L+3 (70.3%)<br>H-8→L+1 (11.3%)	0.0000	0.9658	0.0003	0.1017
S_{37}	4.4511	278.55	H-2→L+4 (70.6%)<br>H-8→L+2 (11.2%)	-0.9559	0.0000	0.0000	0.0996
S_{38}	4.5346	273.42	H-3→L+3 (28.3%)<br>H-4→L+4 (23.2%)<br>H-2→L+3 (15.1%)<br>H-2→L+6 (11.2%)<br>H-8→L+1 (8.3%)	0.0000	-1.9661	-0.0057	0.4294
S_{39}	4.5351	273.39	H-3→L+4 (26.2%)<br>H-4→L+3 (25.7%)<br>H-2→L+4 (14.8%)<br>H-2→L+5 (11.2%)<br>H-8→L+2 (8.2%)	-1.9711	0.0000	0.0000	0.4317
S_{52}	4.8773	254.21	H-5→L+5 (41.9%)<br>H-9→L+1 (18.2%)<br>H-10→L+2 (18.0%)<br>H-4→L+5 (5.6%)<br>H-3→L+6 (5.5%)	0.0000	1.3414	0.0012	0.2150
S_{53}	4.8776	254.19	H-5→L+6 (41.5%)<br>H-9→L+2 (19.3%)<br>H-10→L+1 (18.1%)<br>H-3→L+5 (5.3%)<br>H-4→L+6 (5.2%)	1.2968	0.0000	0.0000	0.2010
S_{54}	4.9039	252.83	H-9→L+2 (20.9%)<br>H-2→L+5 (18.8%)<br>H-10→L+1 (14.3%)<br>H-5→L+6 (11.7%)<br>H-9→L (6.5%)<br>H→L+7 (6.2%)	2.4924	0.0000	0.0000	0.7463
S_{55}	4.9040	252.82	H-2→L+6 (18.5%)<br>H-9→L+1 (18.5%)<br>H-10→L+2 (17.8%)<br>H-5→L+5 (10.9%)<br>H-10→L (6.6%)<br>H-1→L+7 (6.1%)	0.0000	2.4750	0.0003	0.7360

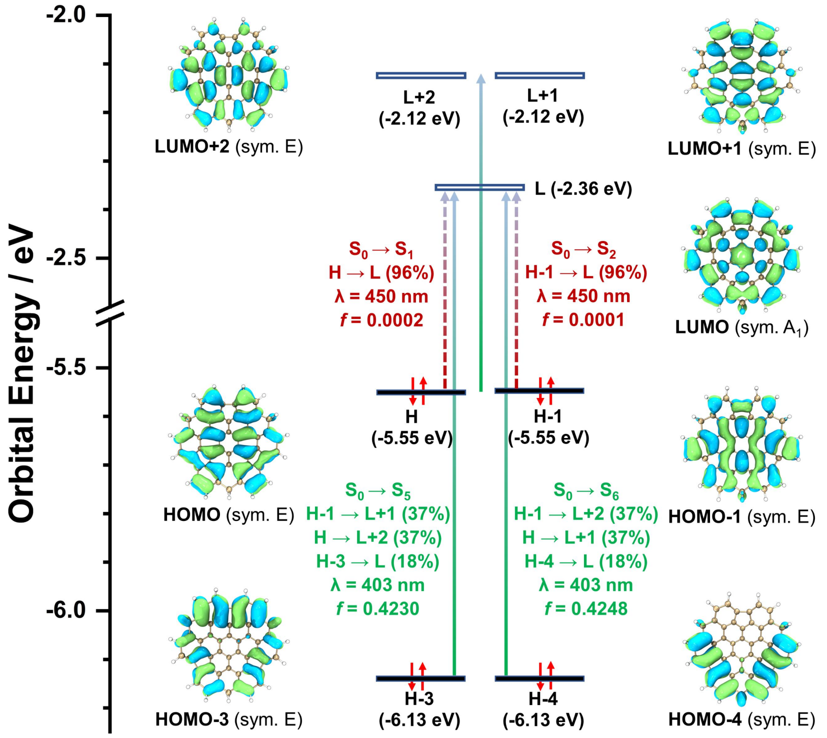

Fig. S70. Orbital correlation diagram and transition composition of the $S_0 \rightarrow (S_1, S_2, S_5, S_6)$ excited states for 1-H calculated at B3LYP/6-311G(d,p) level of theory.

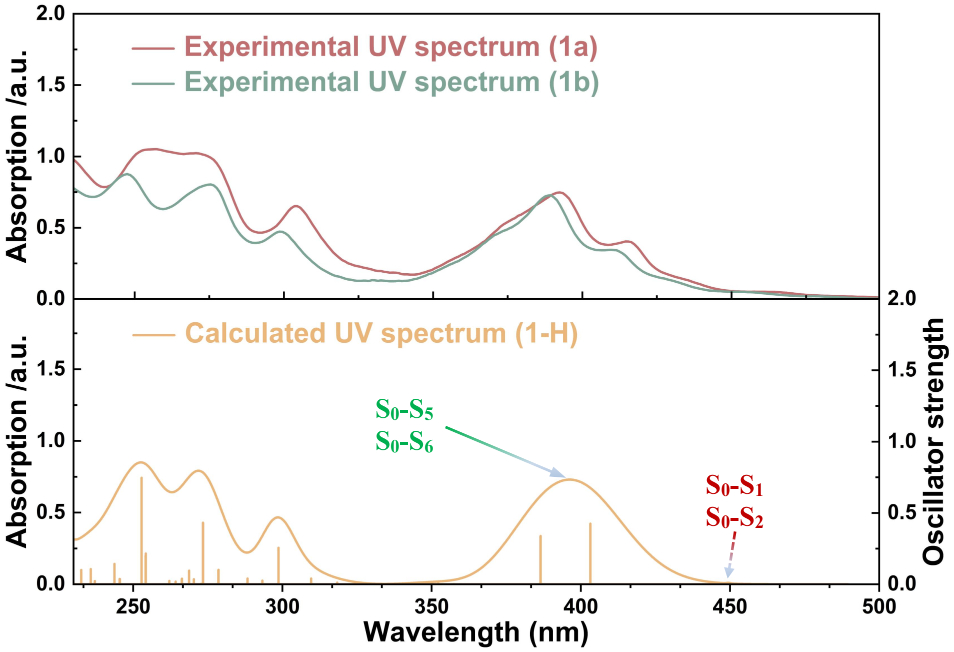

Fig. S71. Experimental and calculated UV-vis absorption spectra of 1a and 1b. The experimental (top) and calculated (bottom) spectra.

Table S21. Major transitions corresponding to the UV spectrum of *c*-HBC calculated by TD1-DFT (B3LYP/6-311G(d,p)).

Excited state	Energy (eV)	Wavelength (nm)	Description	μ_{tr} (a.u.)			Oscillator strength (a.u.)
				X	Y	Z	
S_1	2.7734	447.05	H-1→L+1 (49.4%)<br>H→L (49.4%)	0.0000	0.0005	0.1217	0.0010
S_2	2.8941	428.40	H→L+1 (49.5%)<br>H-1→L (49.5%)	-0.0002	0.0000	0.0000	0.0000
S_3	3.0782	402.78	H-1→L+2 (65.3%)<br>H-2→L (32.7%)	0.0000	0.0000	0.0000	0.0000
S_4	3.0782	402.78	H→L+2 (65.2%)<br>H-2→L+1 (32.8%)	0.0000	0.0000	0.0000	0.0000
S_5	3.1848	389.30	H-1→L (49.4%)<br>H→L+1 (49.4%)	-3.8337	0.0000	0.0000	1.1467
S_6	3.1850	389.28	H→L (49.4%)<br>H-1→L+1 (49.4%)	0.0000	-3.8318	-0.0004	1.1457
S_7	3.2864	377.26	H-2→L (65.3%)<br>H-1→L+2 (32.5%)	0.0000	0.0000	0.0000	0.0000
S_8	3.2865	377.25	H-2→L+1 (65.3%)<br>H→L+2 (32.6%)	0.0000	0.0000	0.0000	0.0000
S_9	3.3800	366.82	H-2→L+2 (98.8%)	-0.0005	0.0000	0.0000	0.0000
S_{10}	3.9989	310.05	H→L+3 (44.4%)<br>H-1→L+4 (44.3%)	0.0000	0.0000	0.0000	0.0000
S_{19}	4.2945	288.70	H→L+5 (86.9%)<br>H-10→L+1 (5.0%)	0.0000	-0.8013	0.0010	0.0676
S_{20}	4.2945	288.70	H-1→L+5 (86.9%)<br>H-10→L (5.1%)	0.8013	0.0000	0.0000	0.0676
S_{22}	4.4382	279.36	H-4→L+2 (51.8%)<br>H-2→L+4 (13.9%)<br>H-5→L (8.8%)<br>H-6→L+1 (8.7%)<br>H-8→L+2 (6.8%)<br>H-10→L (5.6%)	1.8606	0.0000	0.0000	0.3764
S_{23}	4.4383	279.35	H-3→L+2 (51.9%)<br>H-2→L+3 (14.0%)<br>H-6→L (8.7%)<br>H-5→L+1 (8.6%)<br>H-9→L+2 (6.7%)	0.0000	-1.8666	0.0000	0.3788

			H-10→L+1 (5.6%)				
S_{28}	4.4847	276.46	H-5→L (28.3%)<br>H-6→L+1 (28.0%)<br>H-2→L+4 (23.1%)<br>H-4→L+2 (7.8%)<br>H-8→L+2 (7.2%)	2.0938	0.0000	0.0000	0.4817
S_{29}	4.5351	276.45	H-6→L (28.7%)<br>H-5→L+1 (27.9%)<br>H-2→L+3 (23.1%)<br>H-3→L+2 (7.6%)<br>H-9→L+2 (7.2%)	0.0000	-2.0835	-0.0001	0.4770
S_{40}	4.6443	266.96	H-10→L+1 (66.4%)<br>H-9→L+2 (6.3%)<br>H→L+5 (5.5%)	0.0000	1.5272	-0.0017	0.2654
S_{41}	4.6443	266.96	H-10→L (66.5%)<br>H-8→L+2 (6.3%)<br>H-1→L+5 (5.5%)	1.5257	0.0000	0.0000	0.2649
S_{43}	4.6743	265.25	H→L+8 (25.2%)<br>H-1→L+7 (25.0%)<br>H-5→L+1 (24.0%)<br>H-6→L (24.0%)	0.0000	0.0015	1.4709	0.2478
S_{53}	4.8423	256.04	H-7→L+2 (50.8%)<br>H-2→L+6 (48.3%)	0.0000	-0.0026	1.2150	0.1751
S_{55}	4.9242	251.79	H-2→L+9 (83.3%)<br>H-9→L+2 (6.7%)	0.0000	-1.8557	-0.0019	0.4154
S_{56}	4.9247	251.76	H-2→L+10 (83.4%)<br>H-8→L+2 (6.6%)	1.8538	0.0000	0.0000	0.4146

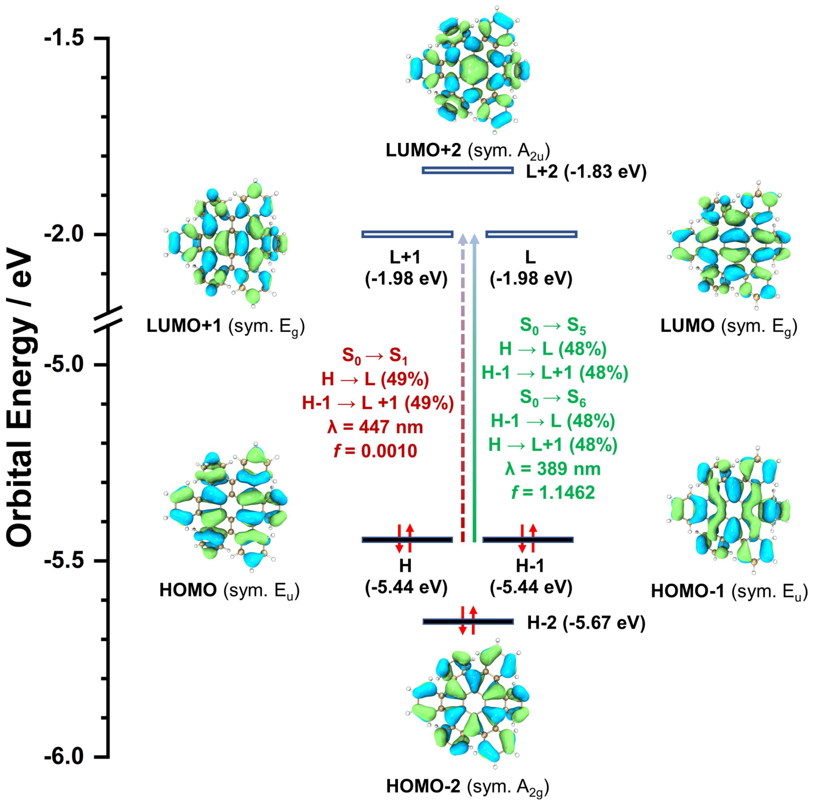

Fig. S72. Orbital correlation diagram and transition composition of the S_0→(S_1, S_2, S_5, S_6) excited states for *c*-HBC calculated at B3LYP/6-311G(d,p) level of theory.

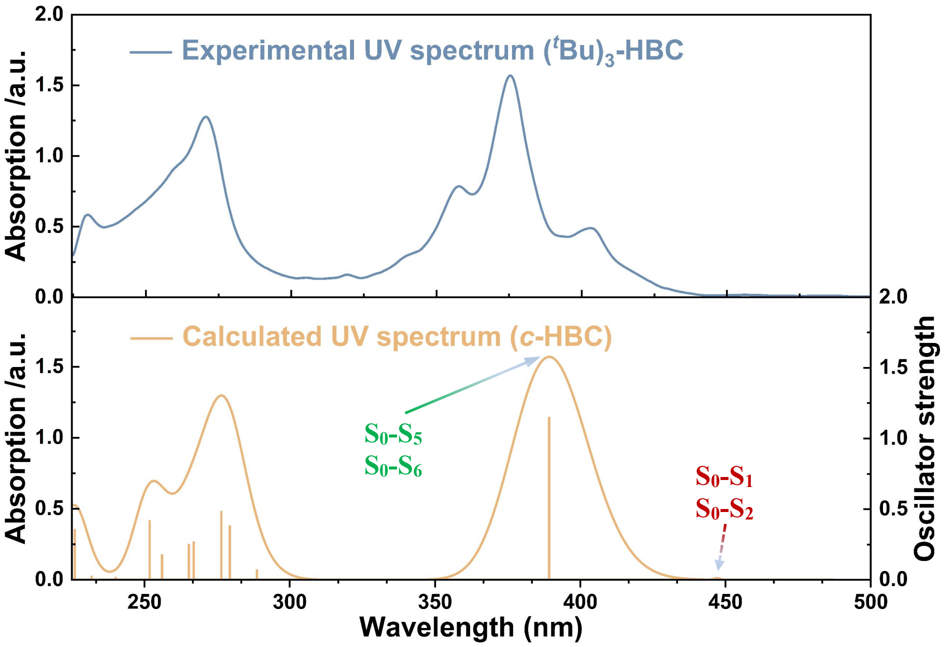

Fig. S73. Experimental and calculated UV-vis absorption spectra of (^{t}Bu)$_3$-HBC. The experimental (top) and calculated (bottom) spectra.

7.5 IGMH maps, and energy decomposition analysis of 1a@C_{60} and 1b@C_{60}

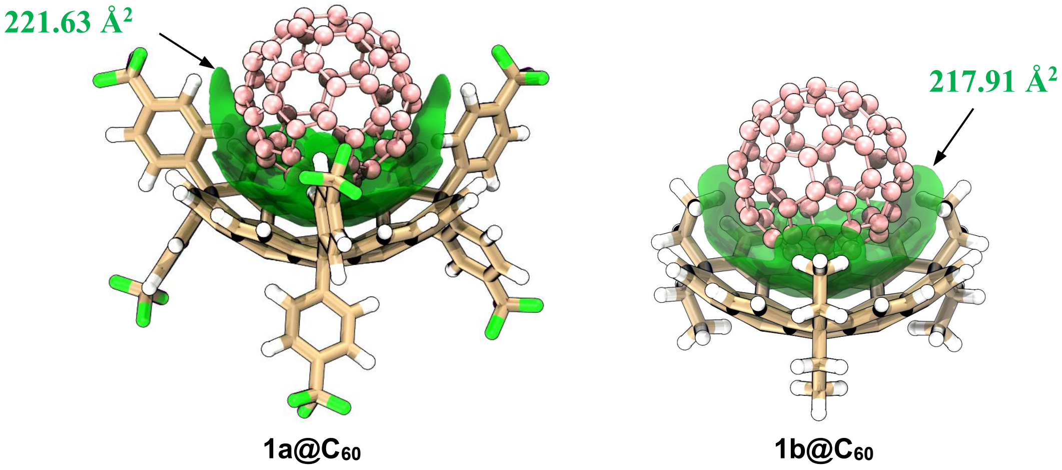

Fig. S74. Calculated IGMH maps of complex 1a@C_{60} and 1b@C_{60} (B3LYP-D3(BJ)/6-31+G(d,p)). Isosurfaces of δ_g^{inter} function with an isovalue of 0.002 a.u. are shown to reveal main interaction regions (colored green).

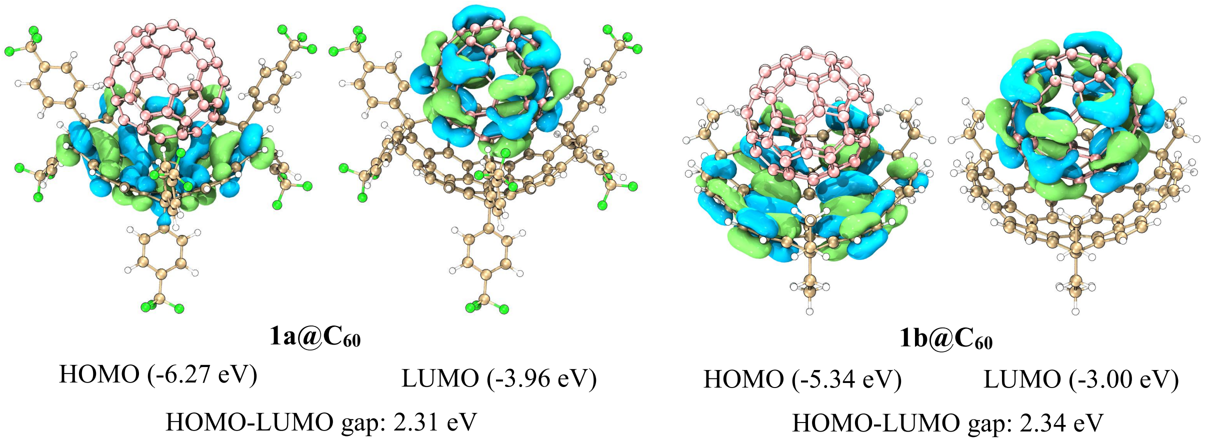

Fig. S75. Frontier molecular orbitals of complex 1a@C_{60} and 1b@C_{60} (B3LYP-D3(BJ)/6-31+G(d,p)).

Table S22. Energy decomposition of intermolecular interactions (kcal/mol) of complex 1a@C_{60} and 1b@C_{60} based on the sobEDAw method (B3LYP-D3(BJ)/6-31+G(d,p)).

Complex	E_{els}[a]	$E_{x\text{-}rep}$[b]	E_{disp}[c]	E_{orb}[d]	E_{int}[e]
**1a@C_{60}**	-25.46	77.46	-86.85	-10.80	-45.66
**1b@C_{60}**	-25.51	74.53	-81.23	-9.72	-41.93
**Deference (1a-1b)**	+0.05	+2.92	-5.62	-1.08	-3.73

[a] Average electrostatic interaction energy; [b] Average exchange-repulsion interaction energy; [c] Average dispersion interaction energy; [d] Average orbital interaction energy; [e] Average total interaction energy.

8. Attached NMR spectra and HRMS spectra

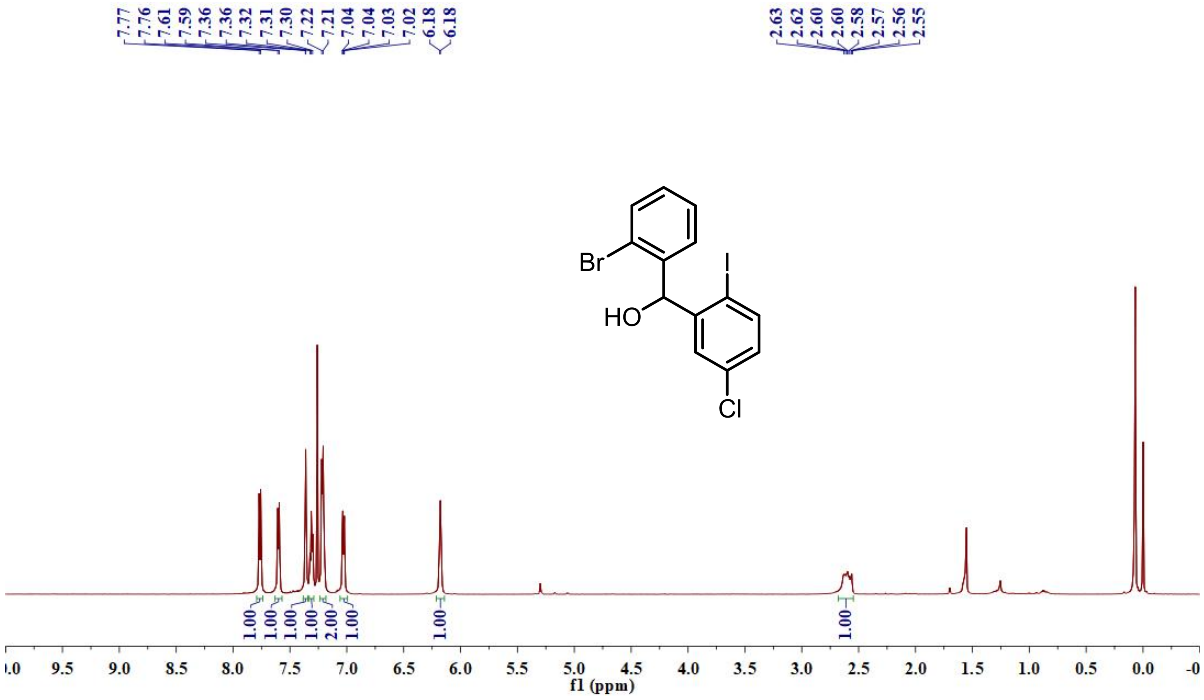

Fig. S76. ^{1}H **NMR spectrum of (2-bromophenyl)(5-chloro-2-iodophenyl)methanol (12) (600 MHz, $CDCl_3$, 298 K).**

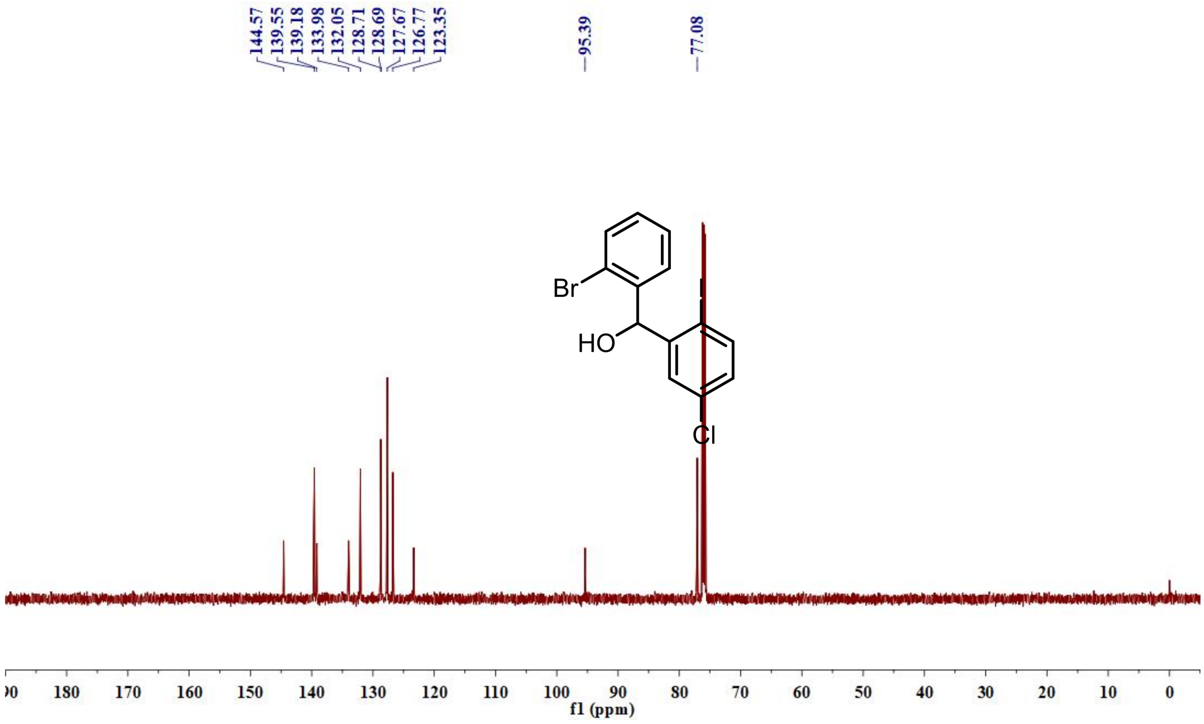

Fig. S77. ^{13}C **NMR spectrum of (2-bromophenyl)(5-chloro-2-iodophenyl)methanol (12) (151 MHz, $CDCl_3$, 298 K).**

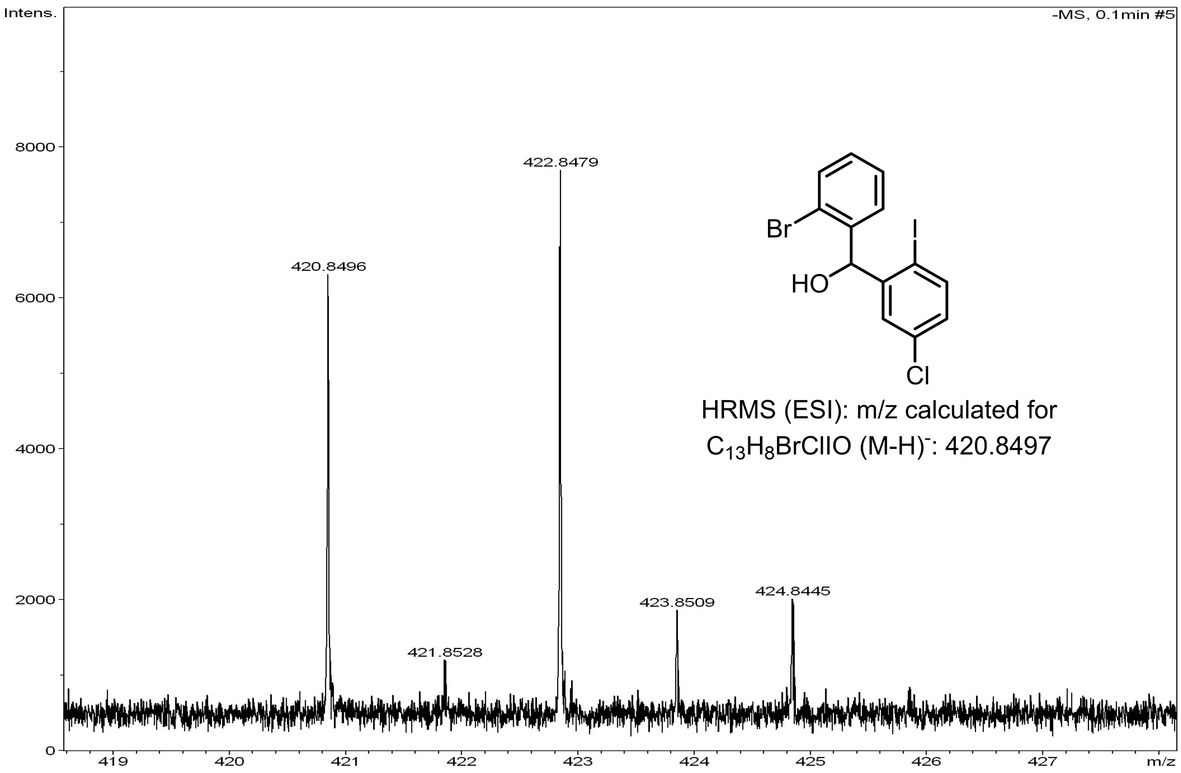

Fig. S78. HRMS (ESI, negative mode, methanol/chloroform) spectrum of (2-bromophenyl)(5-chloro-2-iodophenyl)methanol (12).

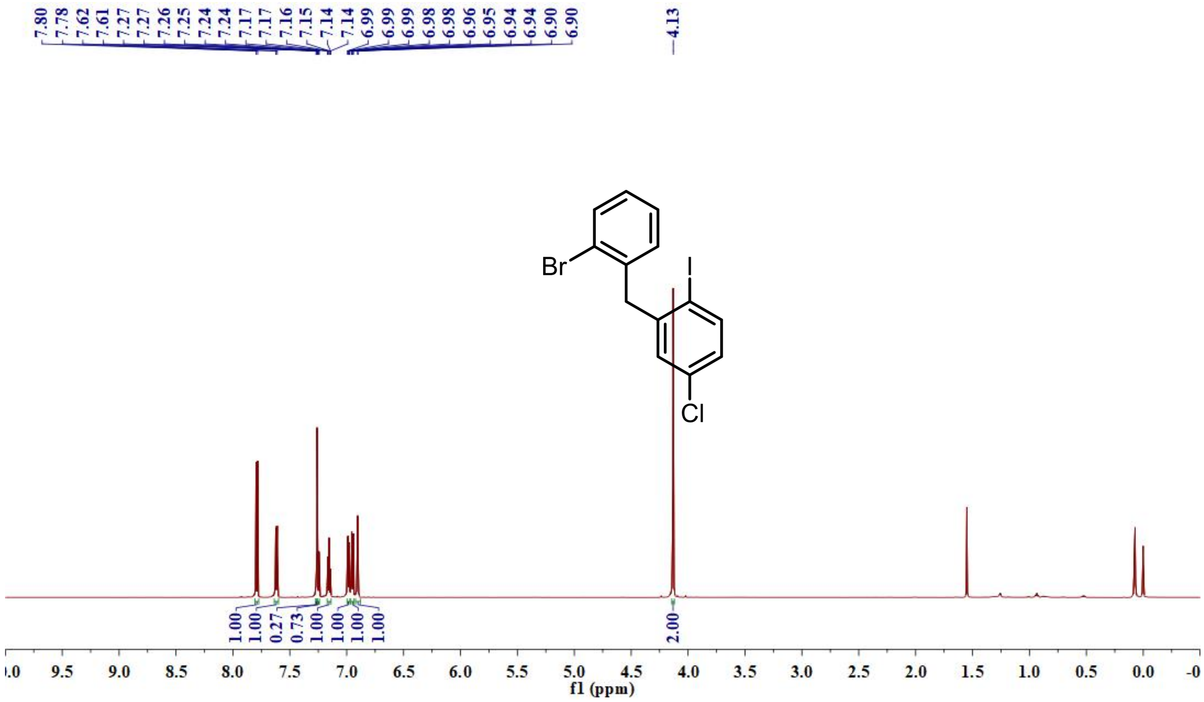

Fig. S79. ^{1}H NMR spectrum of 2-(2-bromobenzyl)-4-chloro-1-iodobenzene (11) (600 MHz, $CDCl_3$, 298 K).

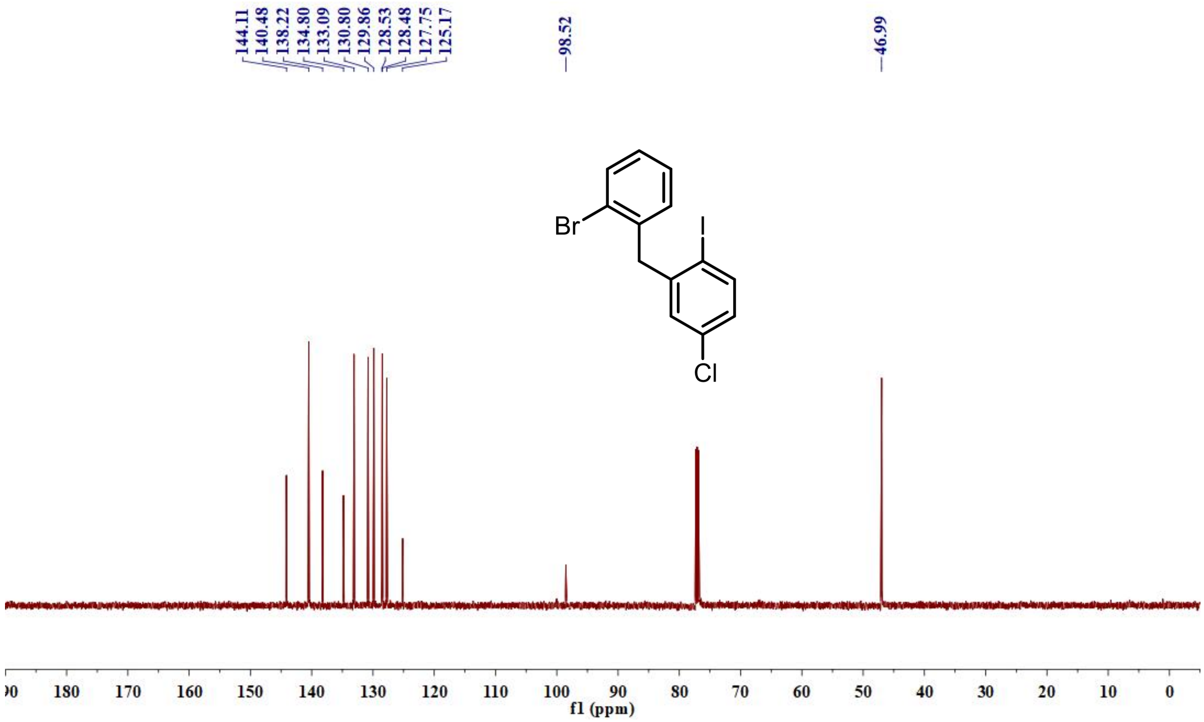

Fig. S80. ^{13}C NMR spectrum of of 2-(2-bromobenzyl)-4-chloro-1-iodobenzene (11) (151 MHz, $CDCl_3$, 298 K).

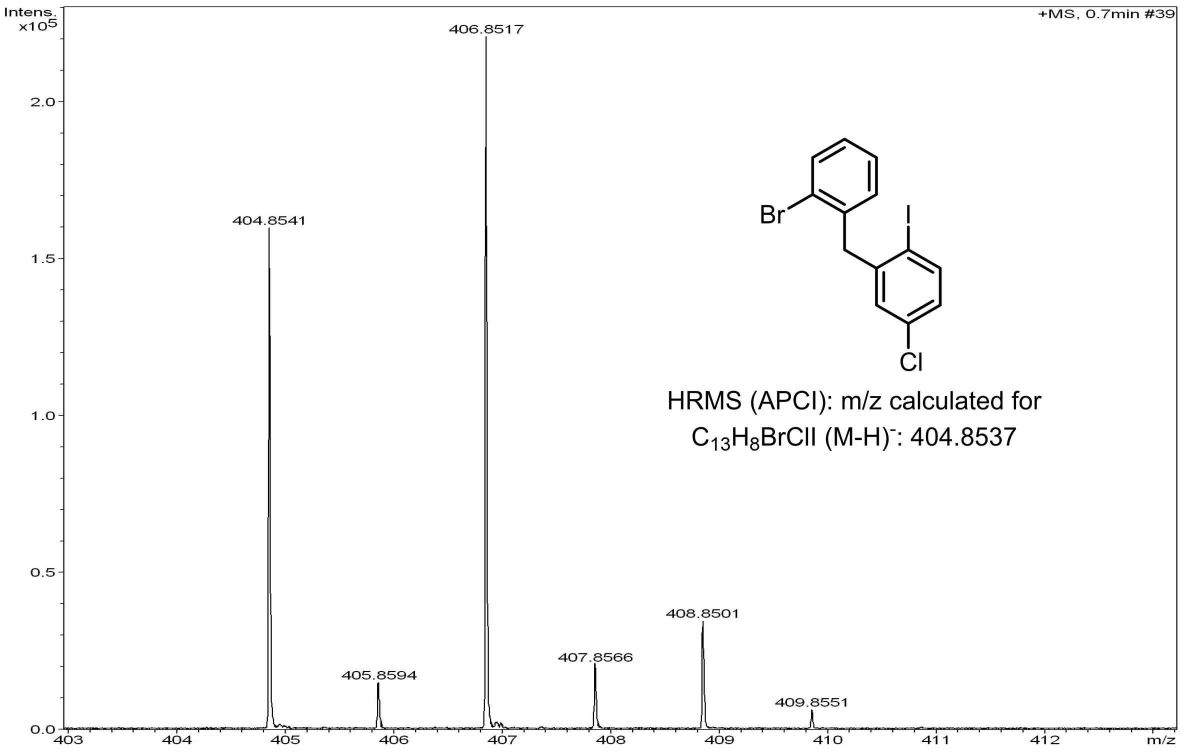

Fig. S81. HRMS (APCI, negative mode, methanol/chloroform) spectrum of 2-(2-bromobenzyl)-4-chloro-1-iodobenzene (11).

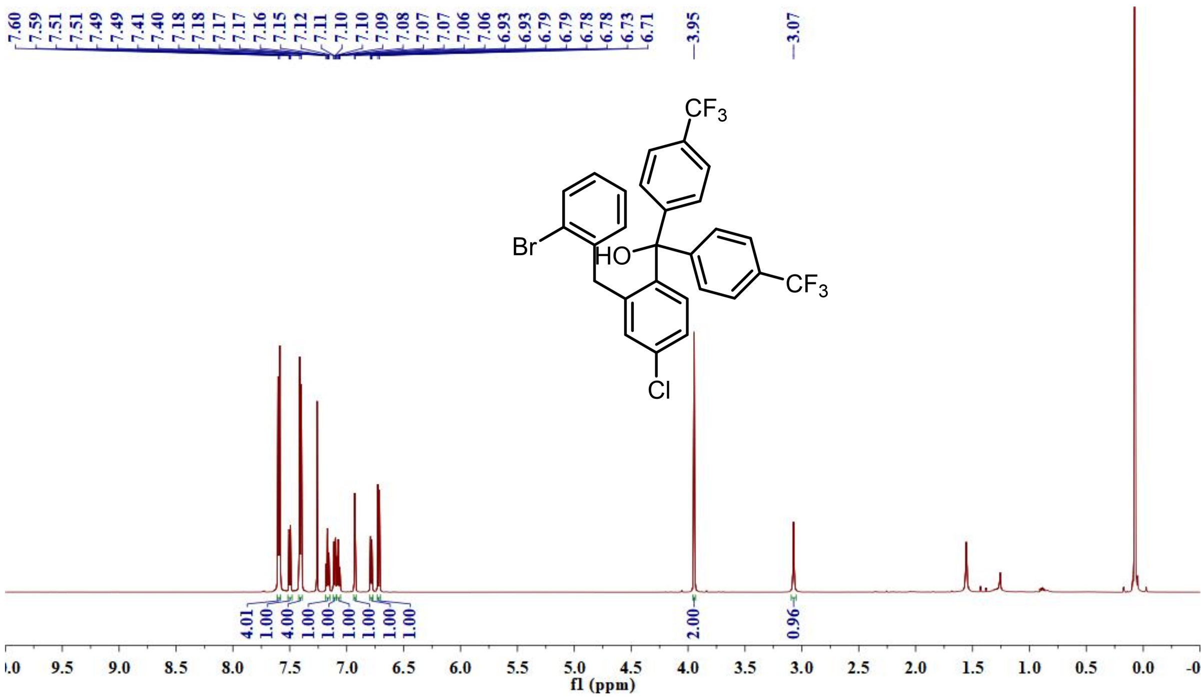

Fig. S82. ^{1}H **NMR spectrum of (2-(2-bromobenzyl)-4-chlorophenyl)bis(4-(trifluoromethyl)phenyl)methanol (9a) (600 MHz,** $CDCl_3$**, 298 K).**

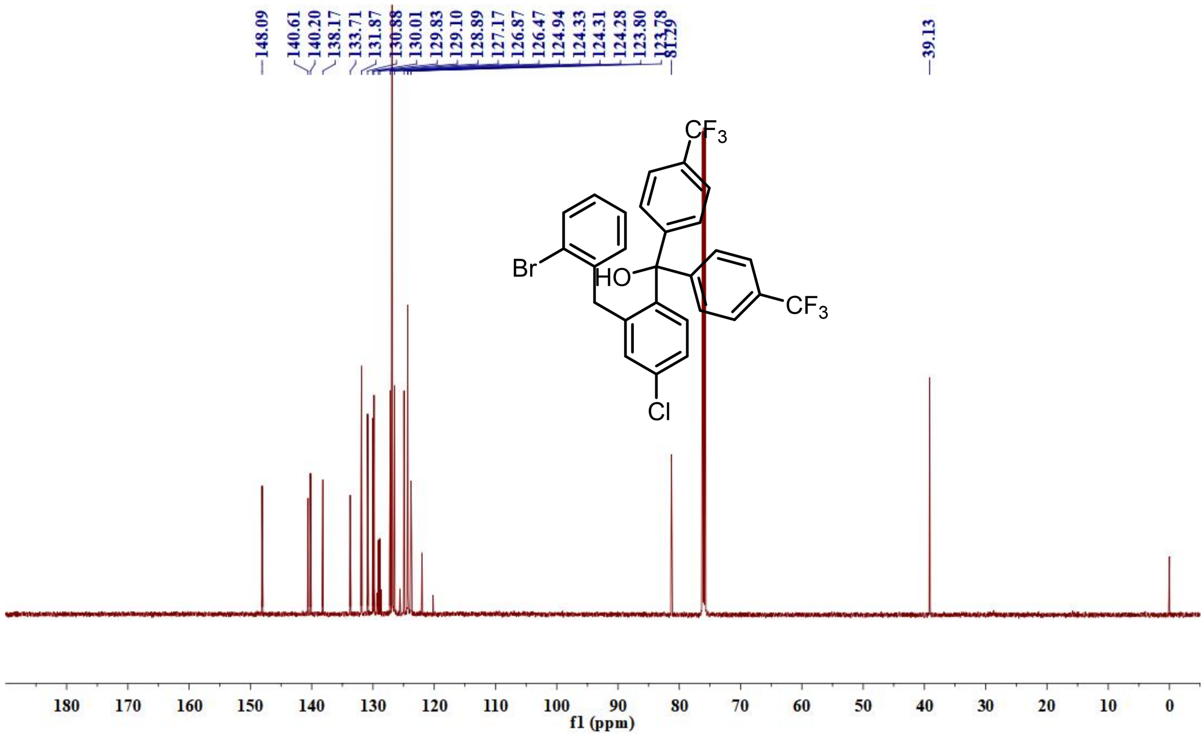

Fig. S83. ^{13}C **NMR spectrum of (2-(2-bromobenzyl)-4-chlorophenyl)bis(4-(trifluoromethyl)phenyl)methanol (9a) (151 MHz,** $CDCl_3$**, 298 K).**

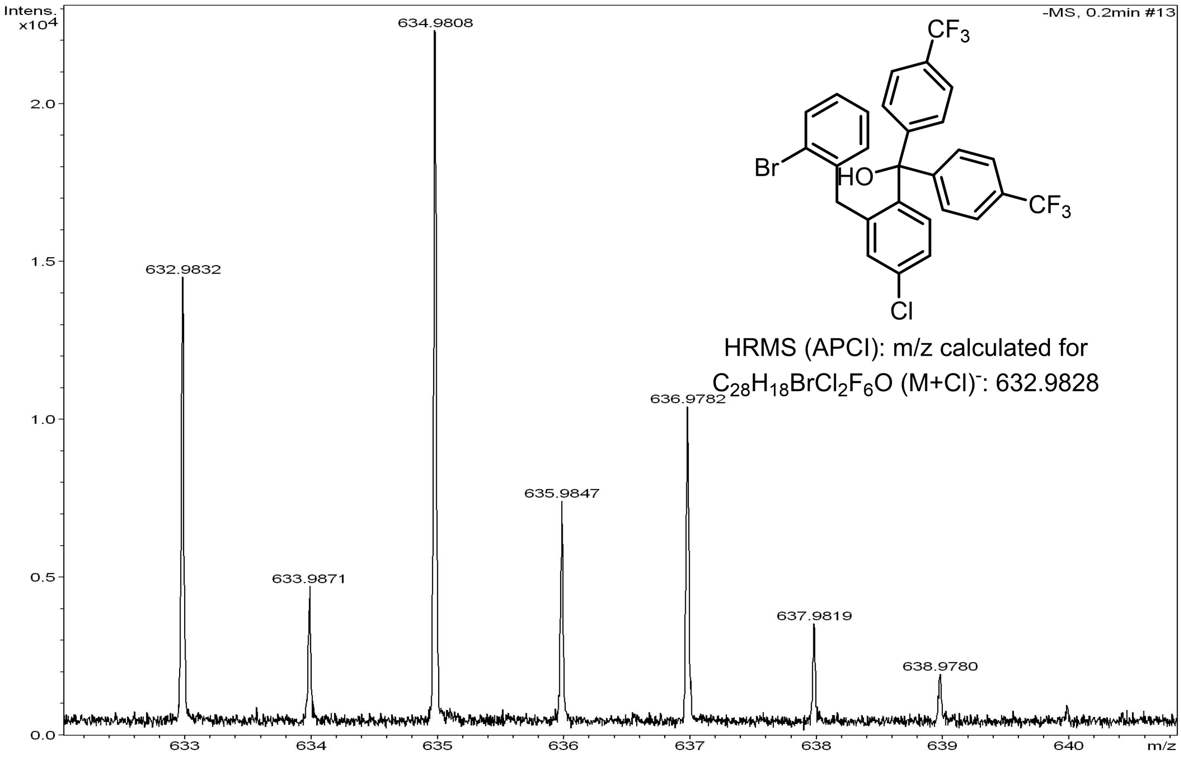

Fig. S84. HRMS (APCI, negative mode, methanol/chloroform) spectrum of (2-(2-bromobenzyl)-4-chlorophenyl)bis(4-(trifluoromethyl)phenyl)methanol (9a).

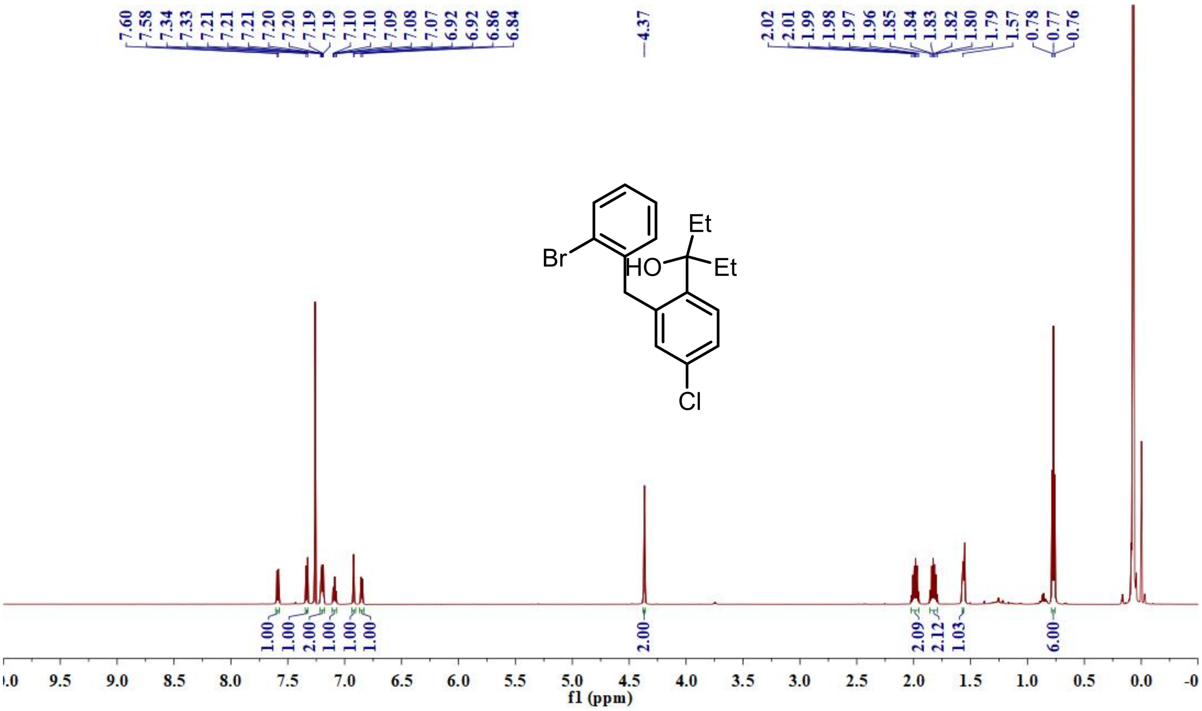

Fig. S85. ^{1}H NMR spectrum of 3-(2-(2-bromobenzyl)-4-chlorophenyl)pentan-3-ol (9b) (600 MHz, $CDCl_3$, 298 K).

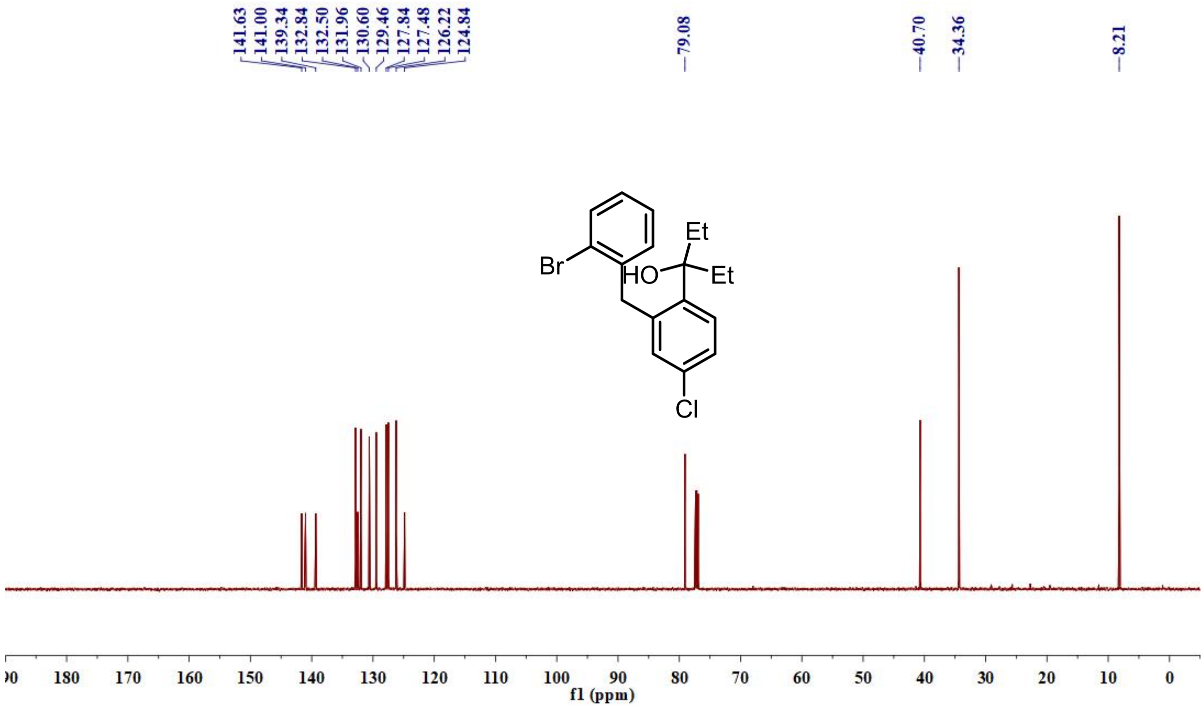

Fig. S86. ^{13}C NMR spectrum of 3-(2-(2-bromobenzyl)-4-chlorophenyl)pentan-3-ol (9b) (151 MHz, $CDCl_3$, 298 K).

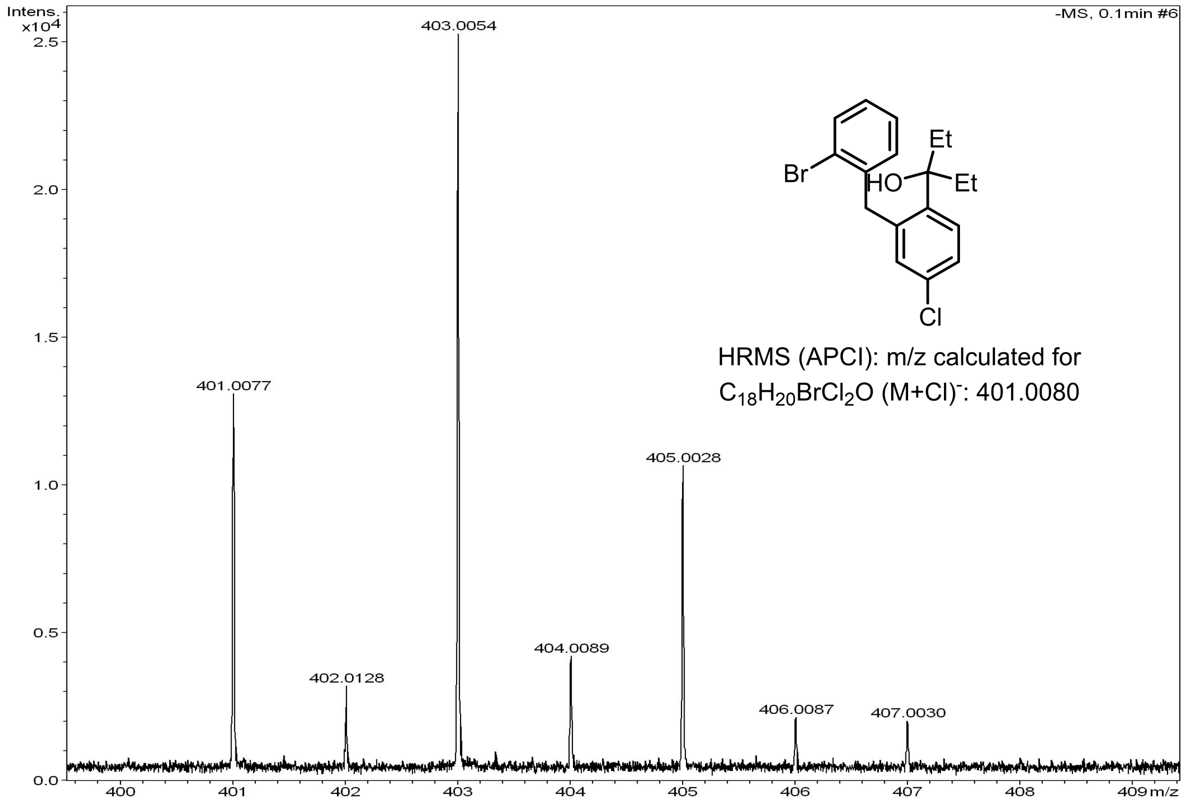

Fig. S87. HRMS (APCI, negative mode, methanol/chloroform) spectrum of 3-(2-(2-bromobenzyl)-4-chlorophenyl)pentan-3-ol (9b).

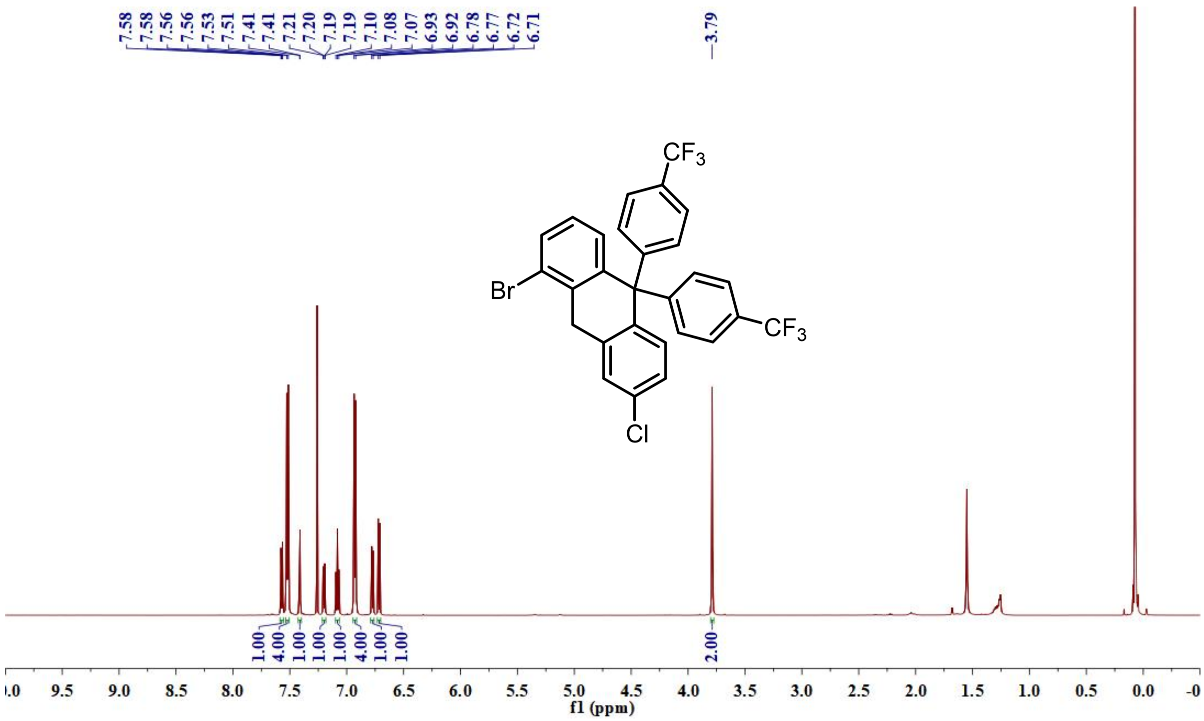

Fig. S88. ^{1}H NMR spectrum of 1-bromo-7-chloro-10,10-bis(4-(trifluoromethyl)phenyl)-9,10-dihydroanthracene (8a) (600 MHz, $CDCl_3$, 298 K).

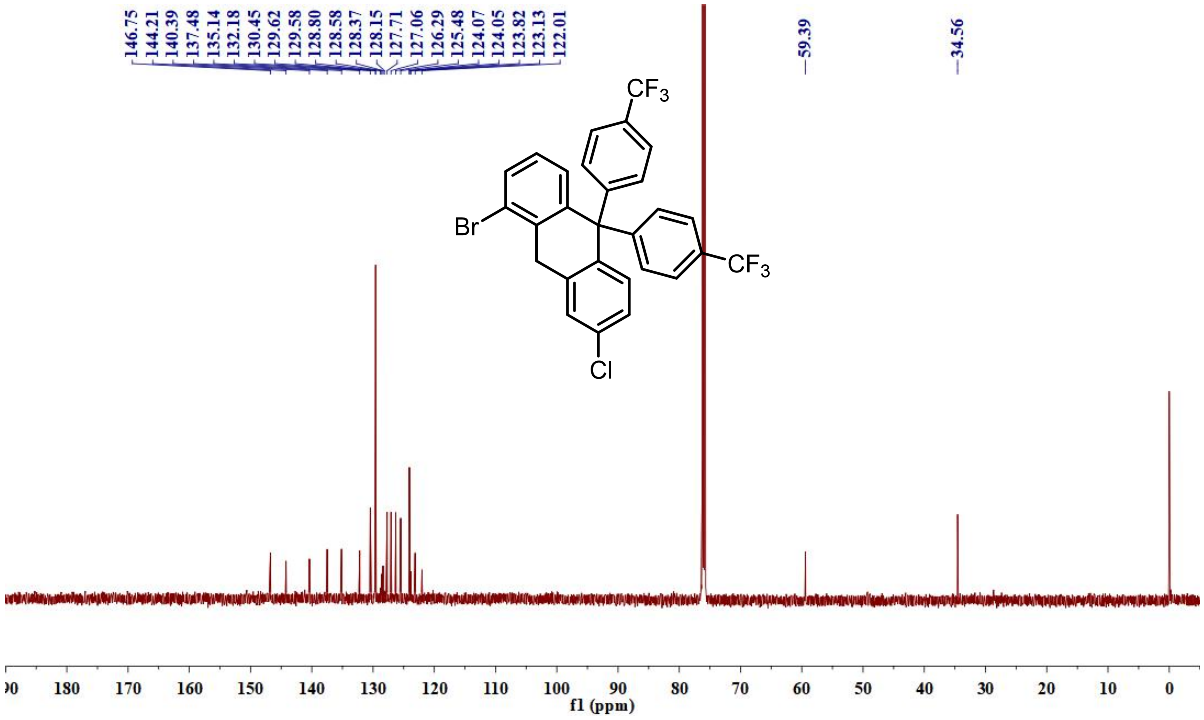

Fig. S89. ^{13}C NMR spectrum of 1-bromo-7-chloro-10,10-bis(4-(trifluoromethyl)phenyl)-9,10-dihydroanthracene (8a) (151 MHz, $CDCl_3$, 298 K).

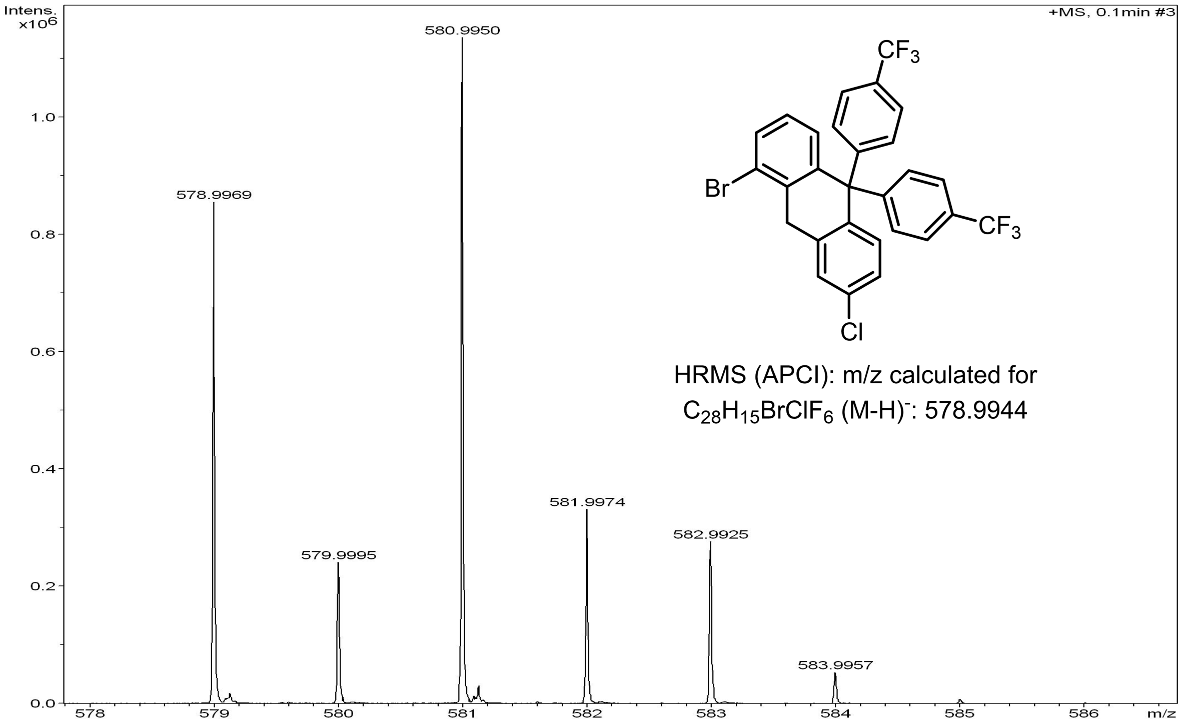

Fig. S90. HRMS (APCI, negative mode, methanol/chloroform) spectrum of 1-bromo-7-chloro-10,10-bis(4-(trifluoromethyl)phenyl)-9,10-dihydroanthracene (8a).

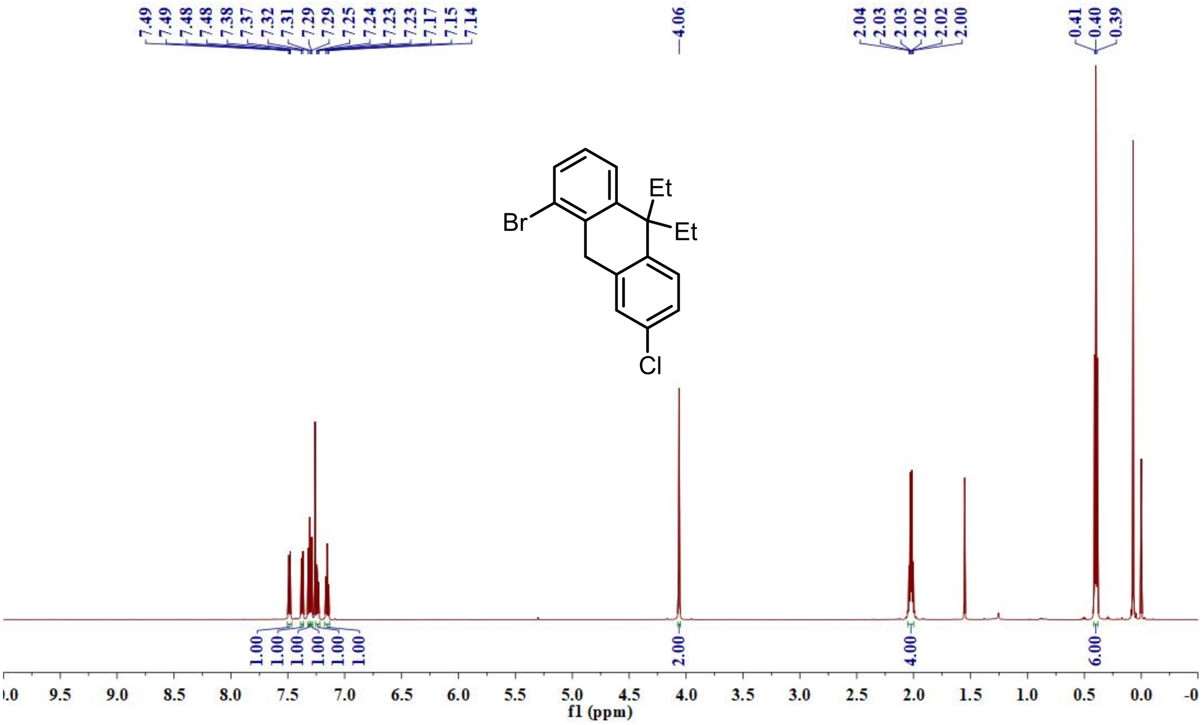

Fig. S91. ^{1}H NMR spectrum of 1-bromo-7-chloro-10,10-diethyl-9,10-dihydroanthracene (8b) (600 MHz, $CDCl_3$, 298 K).

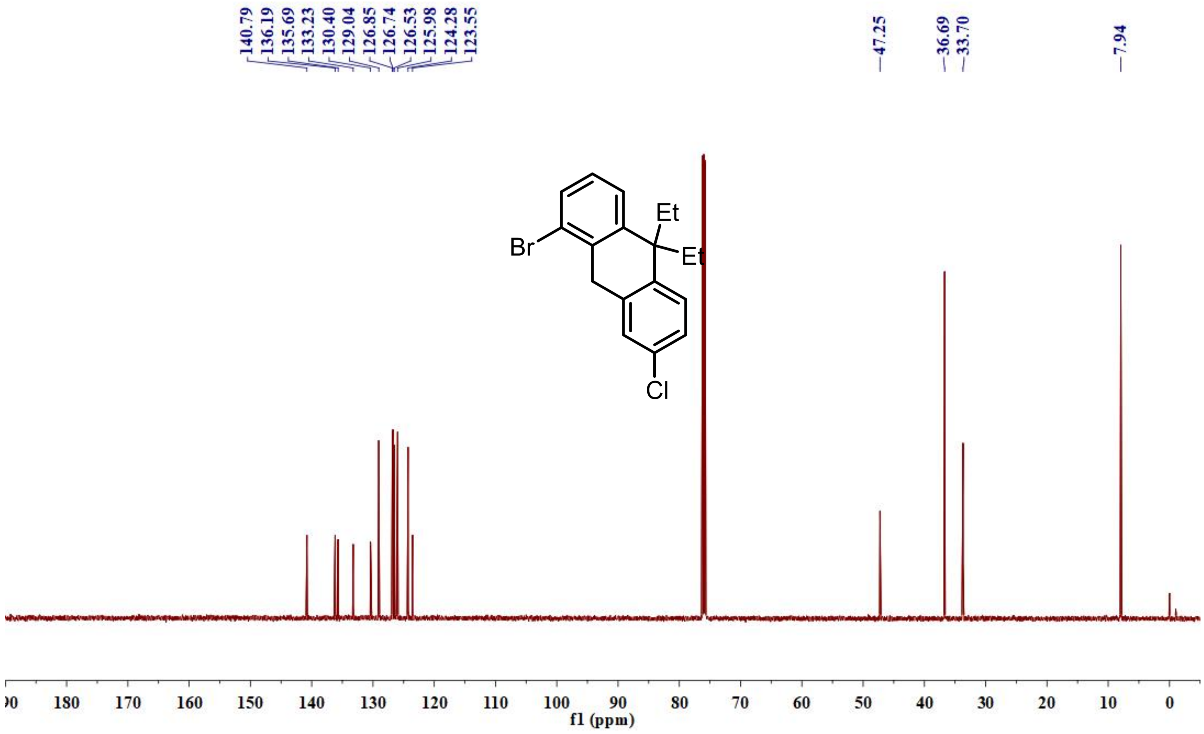

Fig. S92. ^{13}C NMR spectrum of 1-bromo-7-chloro-10,10-diethyl-9,10-dihydroanthracene (8b) (151 MHz, $CDCl_3$, 298 K).

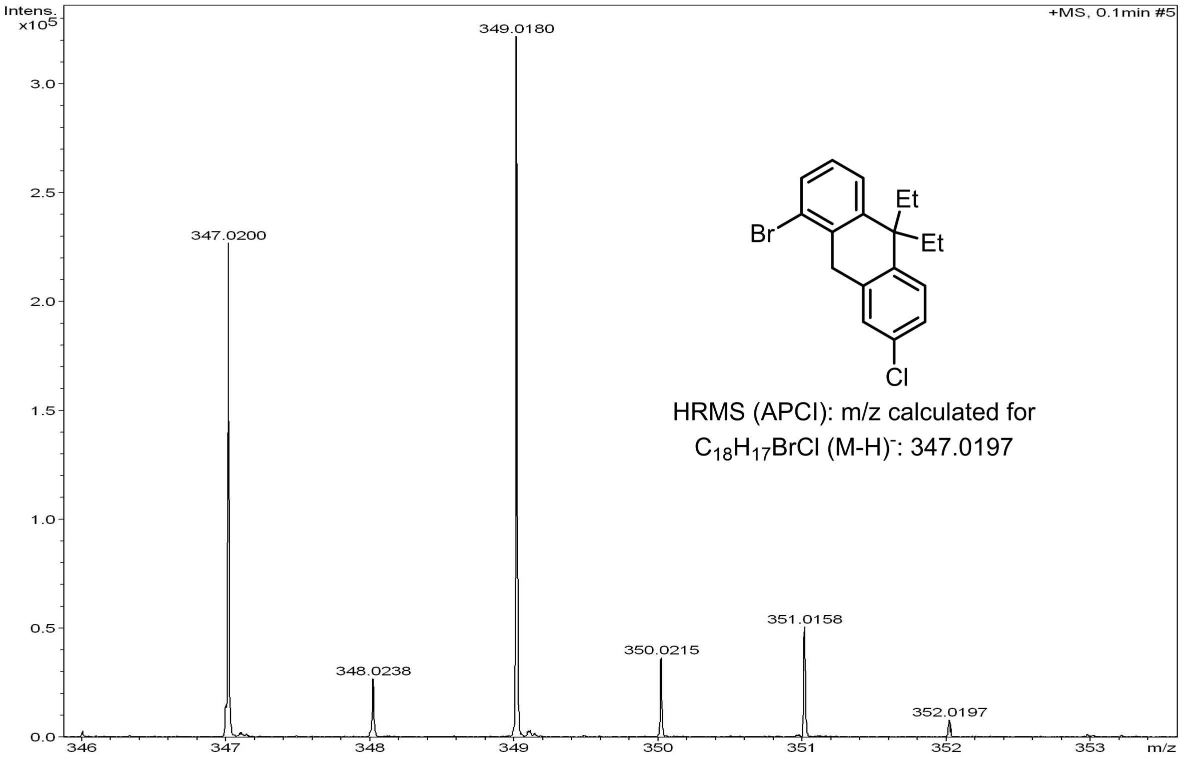

Fig. S93. HRMS (APCI, negative mode, methanol/chloroform) spectrum of 1-bromo-7-chloro-10,10-diethyl-9,10-dihydroanthracene (8b).

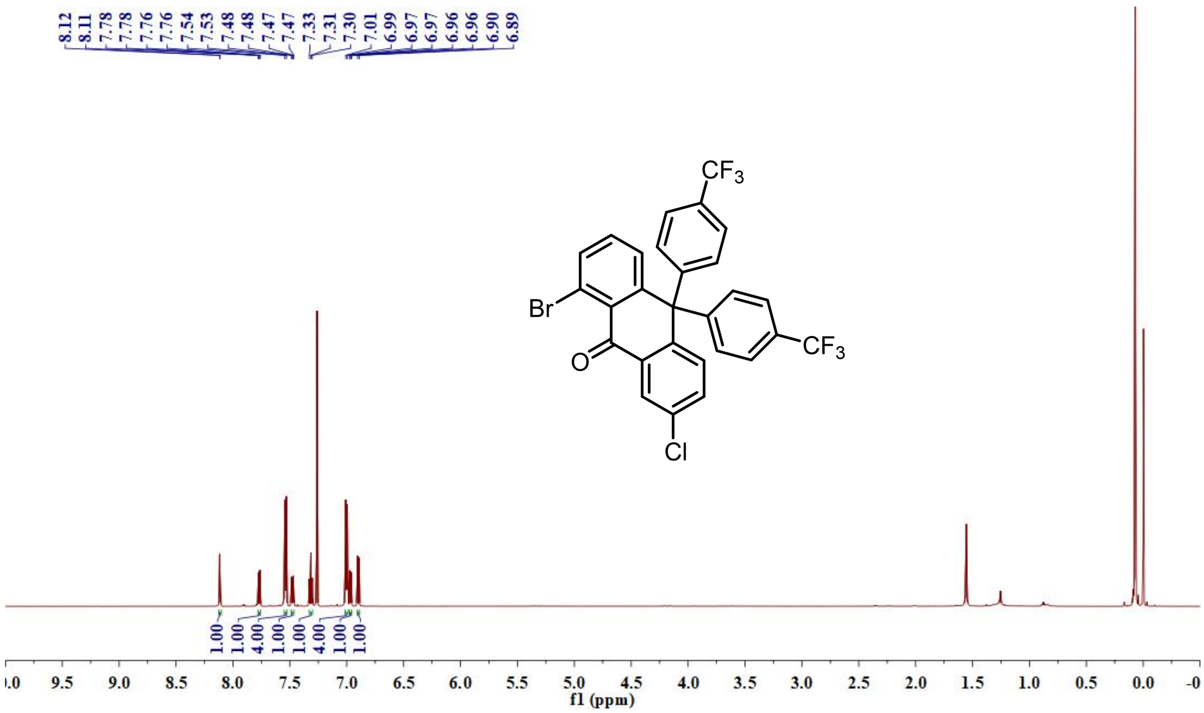

Fig. S94. **^{1}H NMR spectrum of 1-bromo-7-chloro-10,10-bis(4-(trifluoromethyl)phenyl)anthracen-9(10*H*)-one (7a) (600 MHz, $CDCl_3$, 298 K).**

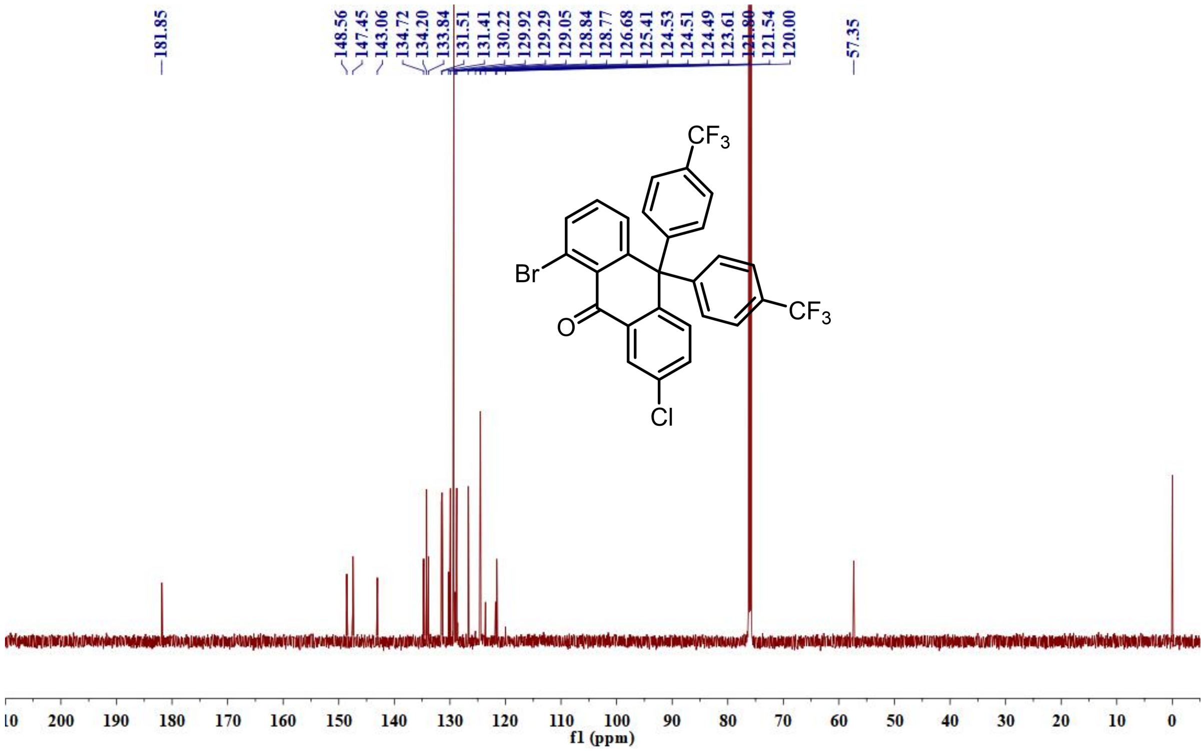

Fig. S95. **^{13}C NMR spectrum of 1-bromo-7-chloro-10,10-bis(4-(trifluoromethyl)phenyl)anthracen-9(10*H*)-one (7a) (151 MHz, $CDCl_3$, 298 K).**

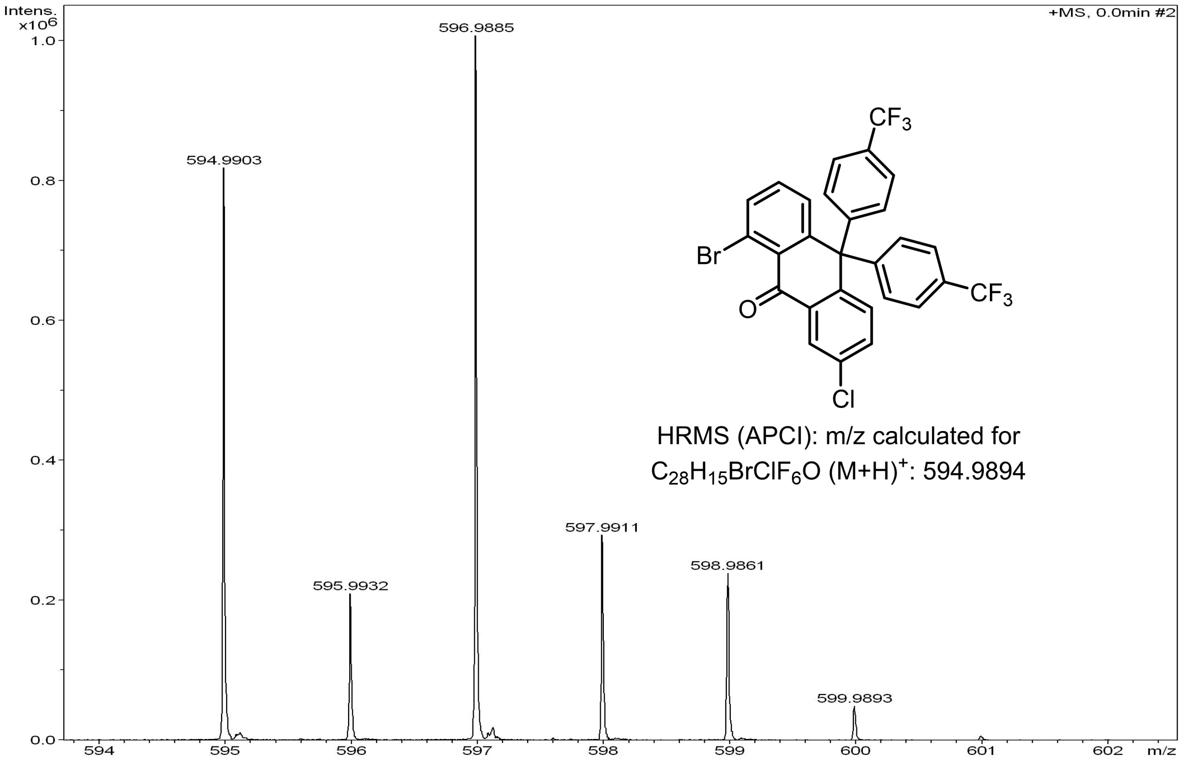

Fig. S96. HRMS (APCI, positive mode, methanol/chloroform) spectrum of 1-bromo-7-chloro-10,10-bis(4-(trifluoromethyl)phenyl)anthracen-9(10*H*)-one (7a).

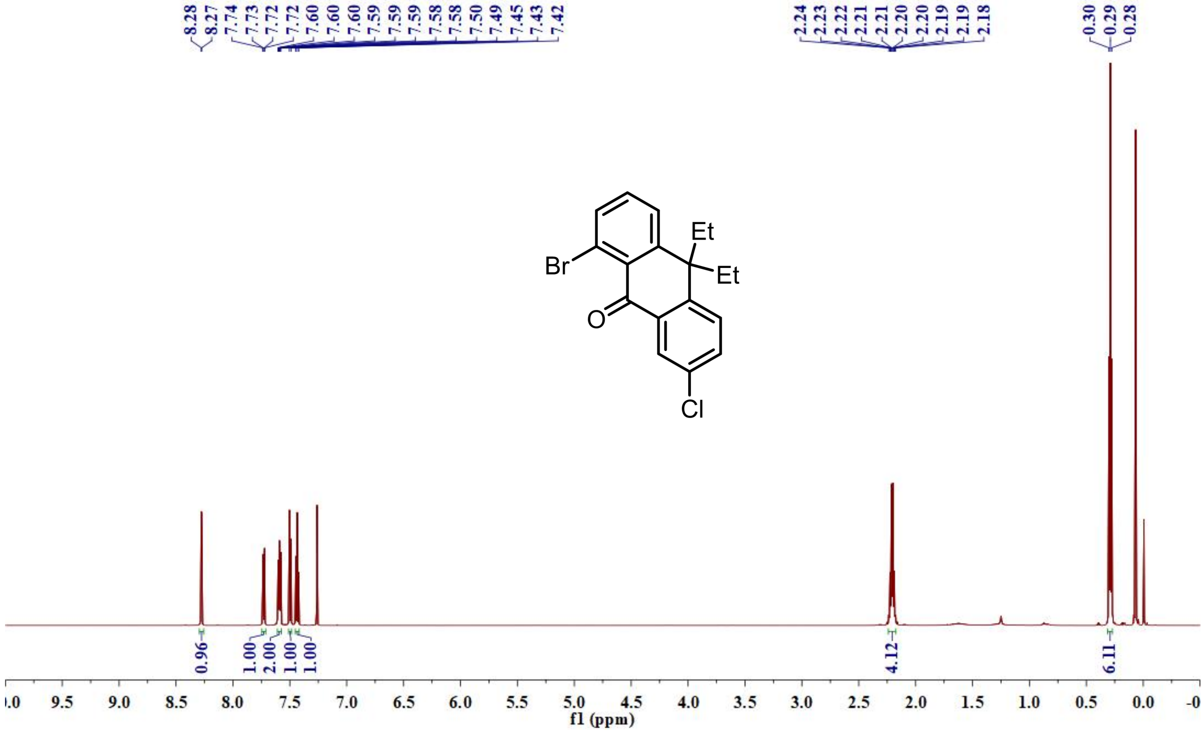

Fig. S97. ^{1}H NMR spectrum of 1-bromo-7-chloro-10,10-diethylanthracen-9(10*H*)-one (7b) (600 MHz, $CDCl_3$, 298 K).

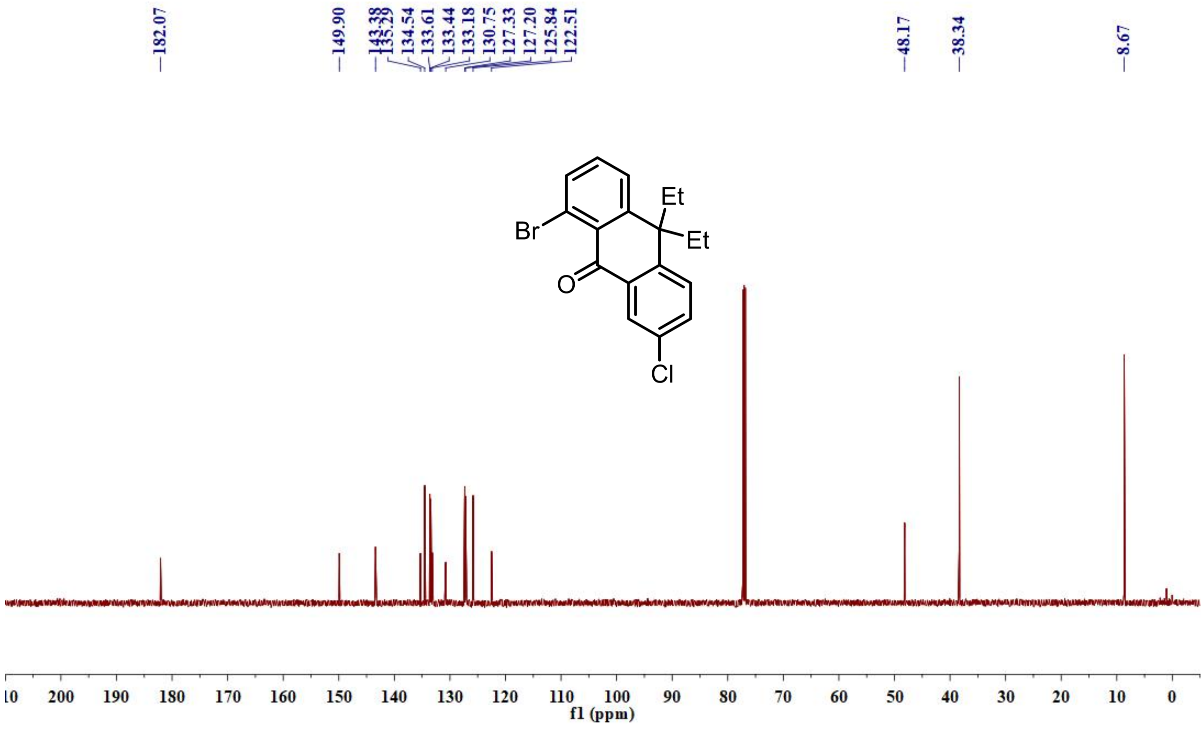

Fig. S98. ^{13}C **NMR spectrum of 1-bromo-7-chloro-10,10-diethylanthracen-9(10*H*)-one (7b) (151 MHz, $CDCl_3$, 298 K).**

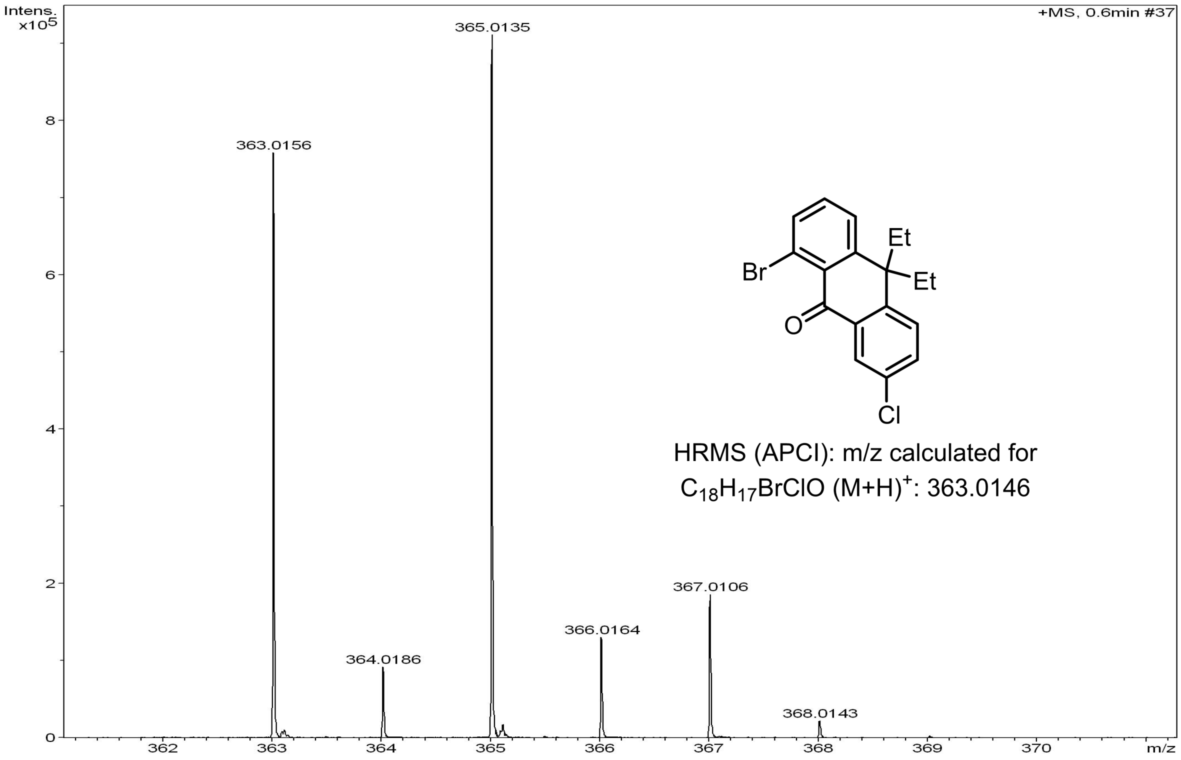

Fig. S99. HRMS (APCI, positive mode, methanol/chloroform) spectrum of 1-bromo-7-chloro-10,10-diethylanthracen-9(10*H*)-one (7b).

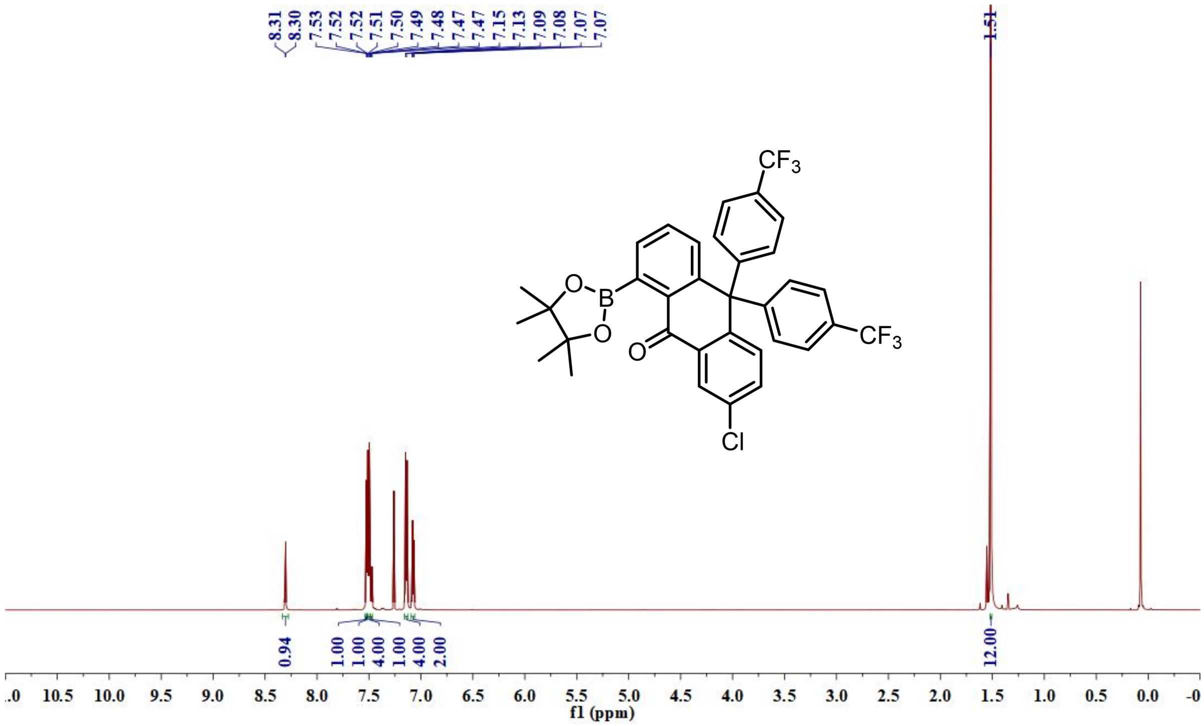

Fig. S100. ^{1}**H NMR spectrum of 7-chloro-1-(4,4,5,5-tetramethyl-1,3,2-dioxaborolan-2-yl)-10,10-bis(4-(trifluoromethyl)phenyl)anthracen-9(10*H*)-one (6a) (600 MHz, CDCl₃, 298 K).**

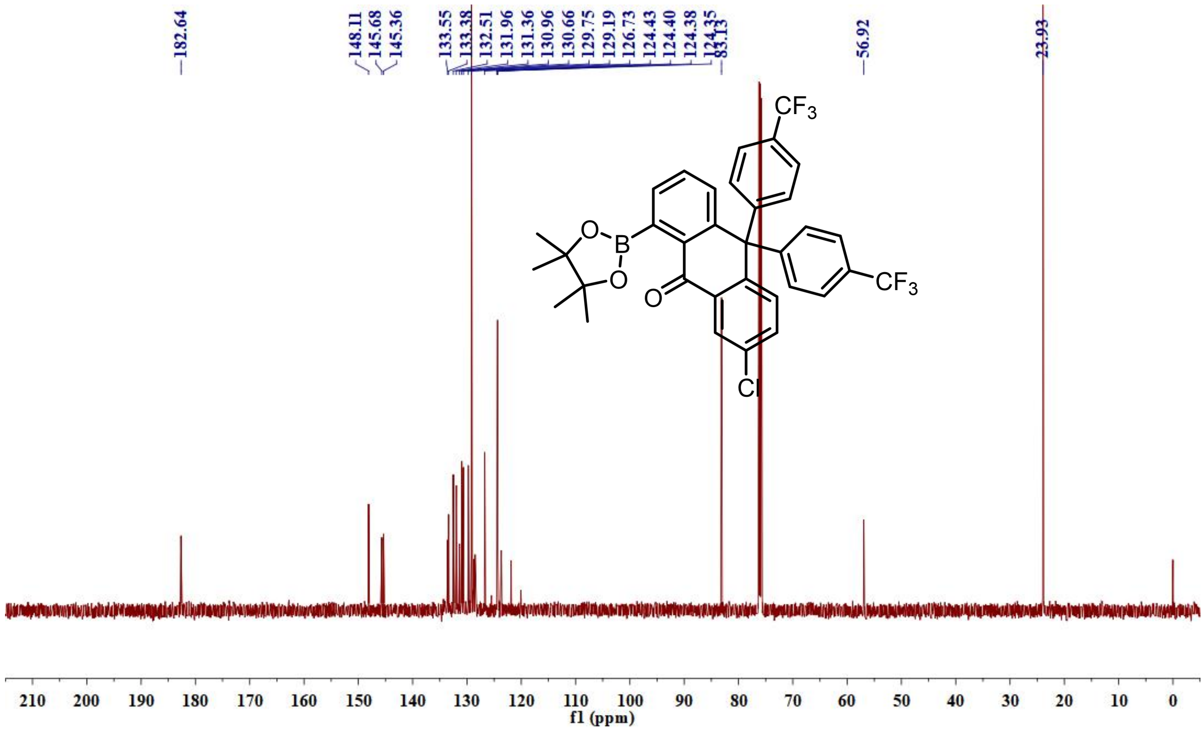

Fig. S101. ^{13}**C NMR spectrum of 7-chloro-1-(4,4,5,5-tetramethyl-1,3,2-dioxaborolan-2-yl)-10,10-bis(4-(trifluoromethyl)phenyl)anthracen-9(10*H*)-one (6a) (151 MHz, CDCl₃, 298 K).**

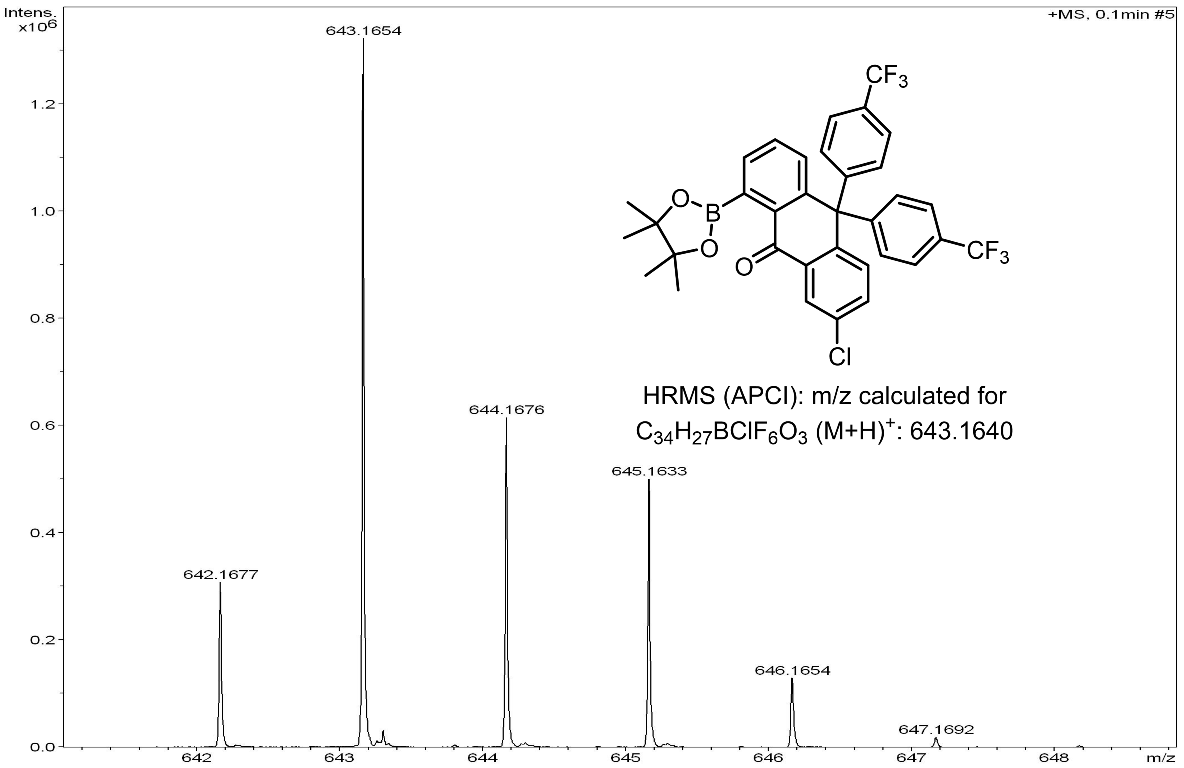

Fig. S102. HRMS (APCI, positive mode, methanol/chloroform) spectrum of 7-chloro-1-(4,4,5,5-tetramethyl-1,3,2-dioxaborolan-2-yl)-10,10-bis(4-(trifluoromethyl)phenyl)anthracen-9(10*H*)-one (6a).

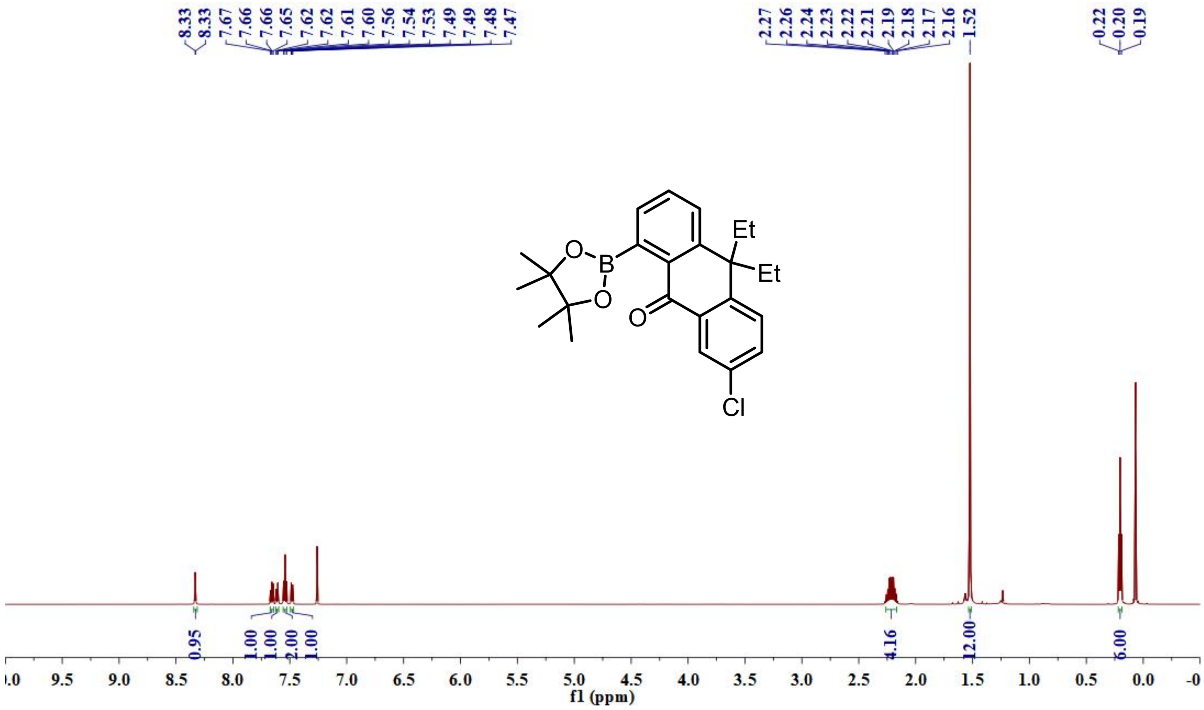

Fig. S103. ^{1}H NMR spectrum of 7-chloro-10,10-diethyl-1-(4,4,5,5-tetramethyl-1,3,2-dioxaborolan-2-yl)anthracen-9(10*H*)-one (6b) (600 MHz, $CDCl_3$, 298 K).

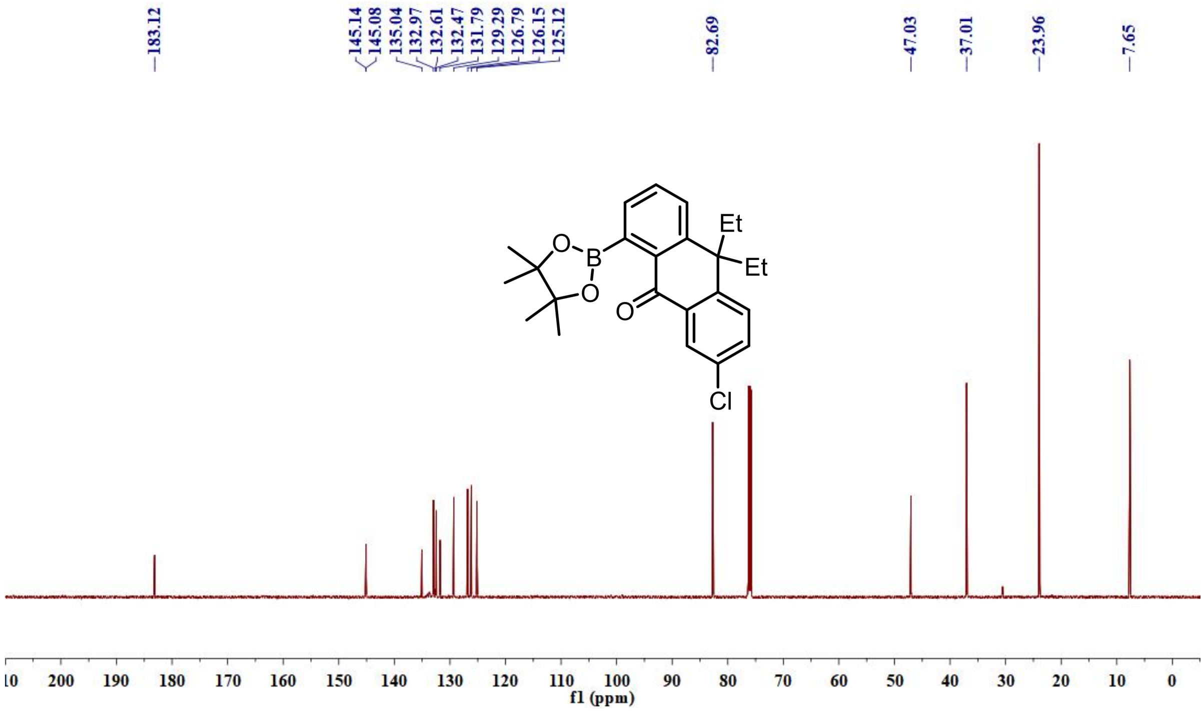

Fig. S104. ^{13}C NMR spectrum of 7-chloro-10,10-diethyl-1-(4,4,5,5-tetramethyl-1,3,2-dioxaborolan-2-yl)anthracen-9(10*H*)-one (6b) (151 MHz, $CDCl_3$, 298 K).

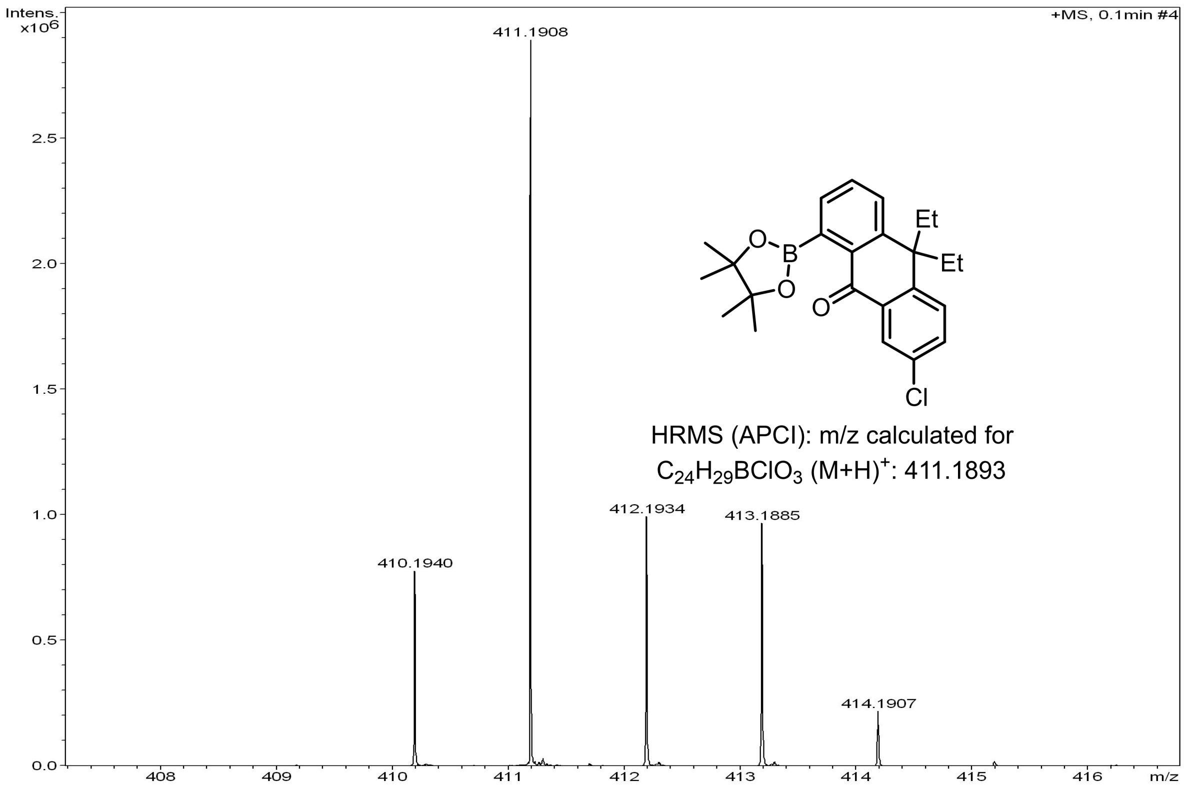

Fig. S105. HRMS (APCI, positive mode, methanol/chloroform) spectrum of 7-chloro-10,10-diethyl-1-(4,4,5,5-tetramethyl-1,3,2-dioxaborolan-2-yl)anthracen-9(10*H*)-one (6b).

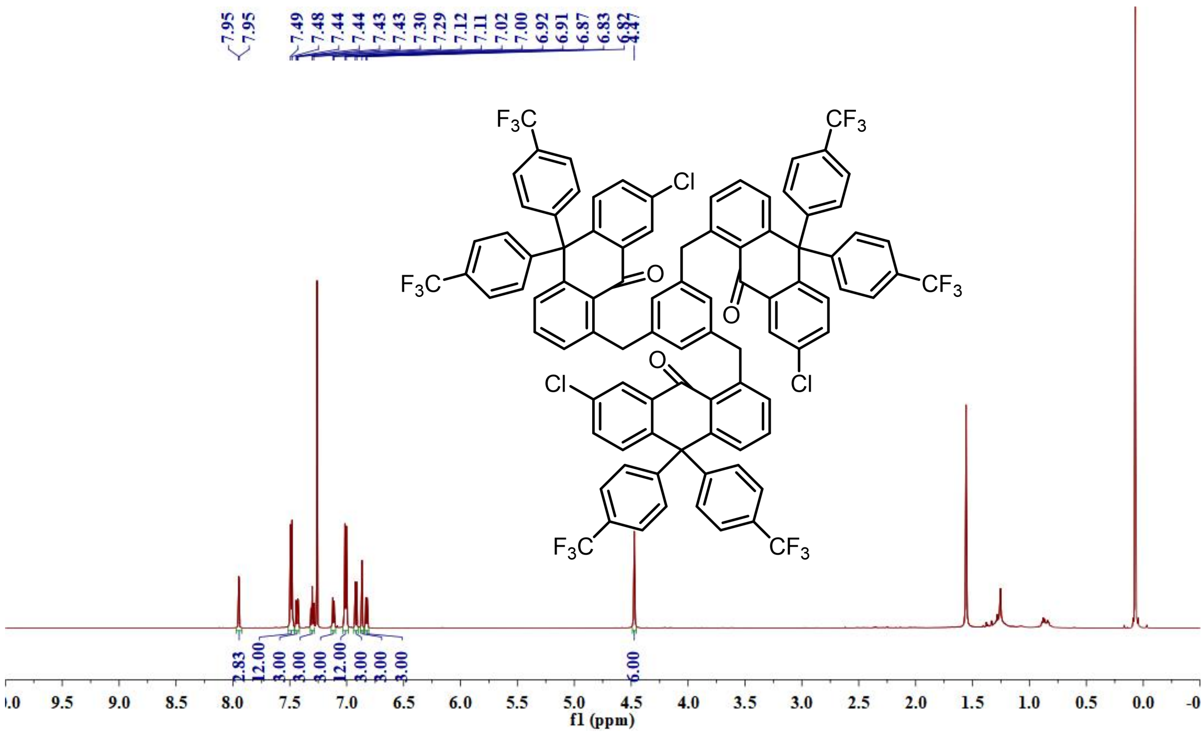

Fig. S106. ^{1}H NMR spectrum of 8,8',8''-(benzene-1,3,5-triyltris(methylene))tris(2-chloro-10,10-bis(4-(trifluoromethyl)phenyl)anthracen-9(10*H*)-one) (4a) (600 MHz, $CDCl_3$, 298 K).

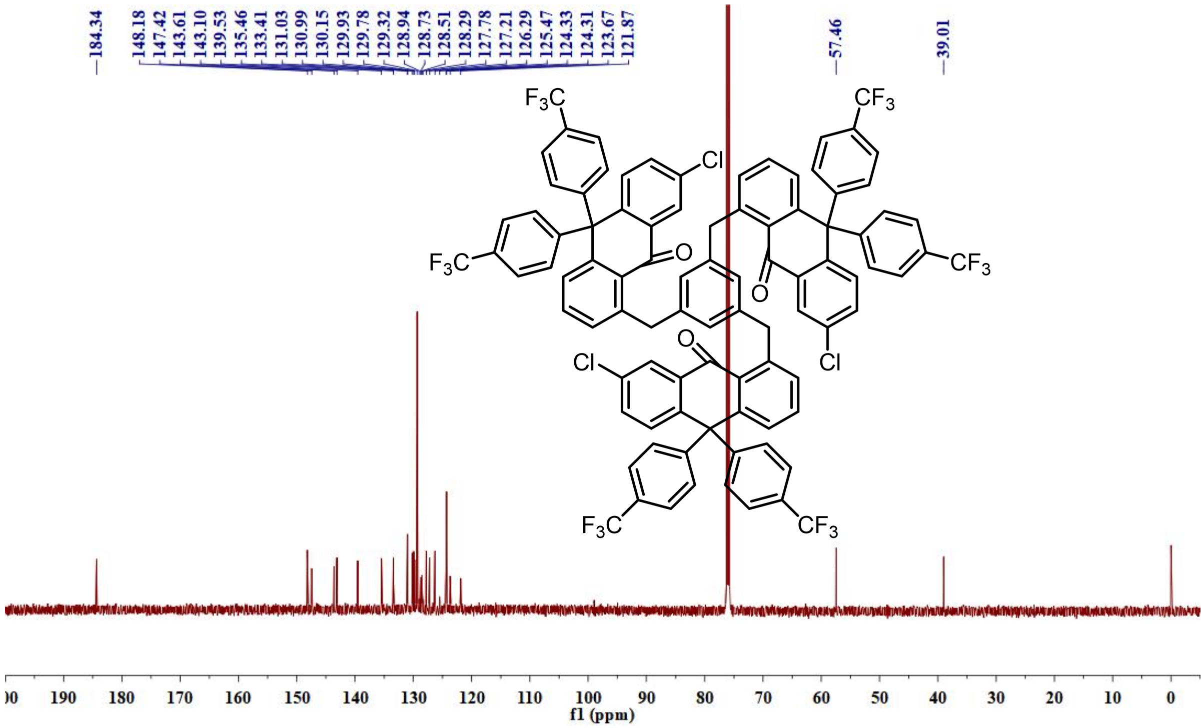

Fig. S107. ^{13}C NMR spectrum of 8,8',8''-(benzene-1,3,5-triyltris(methylene))tris(2-chloro-10,10-bis(4-(trifluoromethyl)phenyl)anthracen-9(10*H*)-one) (4a) (151 MHz, $CDCl_3$, 298 K).

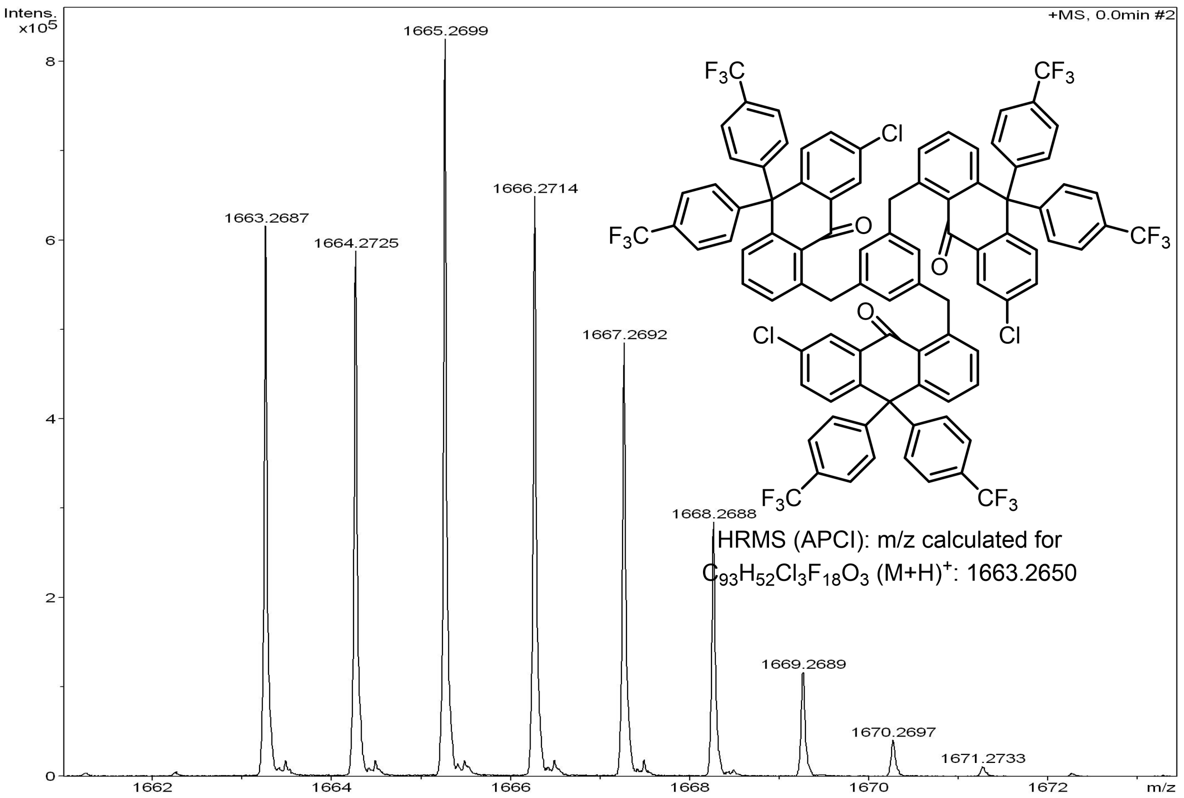

Fig. S108. HRMS (APCI, positive mode, methanol/chloroform) spectrum of 8,8',8''-(benzene-1,3,5-triyltris(methylene))tris(2-chloro-10,10-bis(4-(trifluoromethyl)phenyl)anthracen-9(10*H*)-one) (4a).

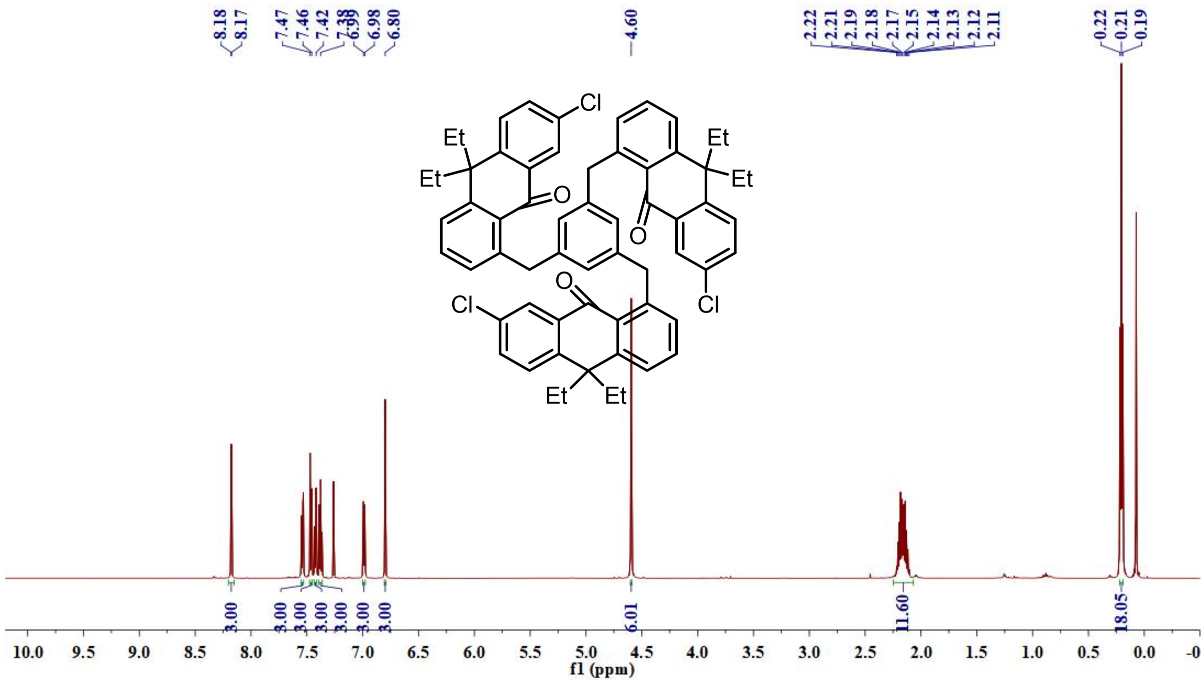

Fig. S109. ^{1}H NMR spectrum of 8,8',8''-(benzene-1,3,5-triyltris(methylene))tris(2-chloro-10,10-diethylanthracen-9(10*H*)-one) (4b) (600 MHz, $CDCl_3$, 298 K).

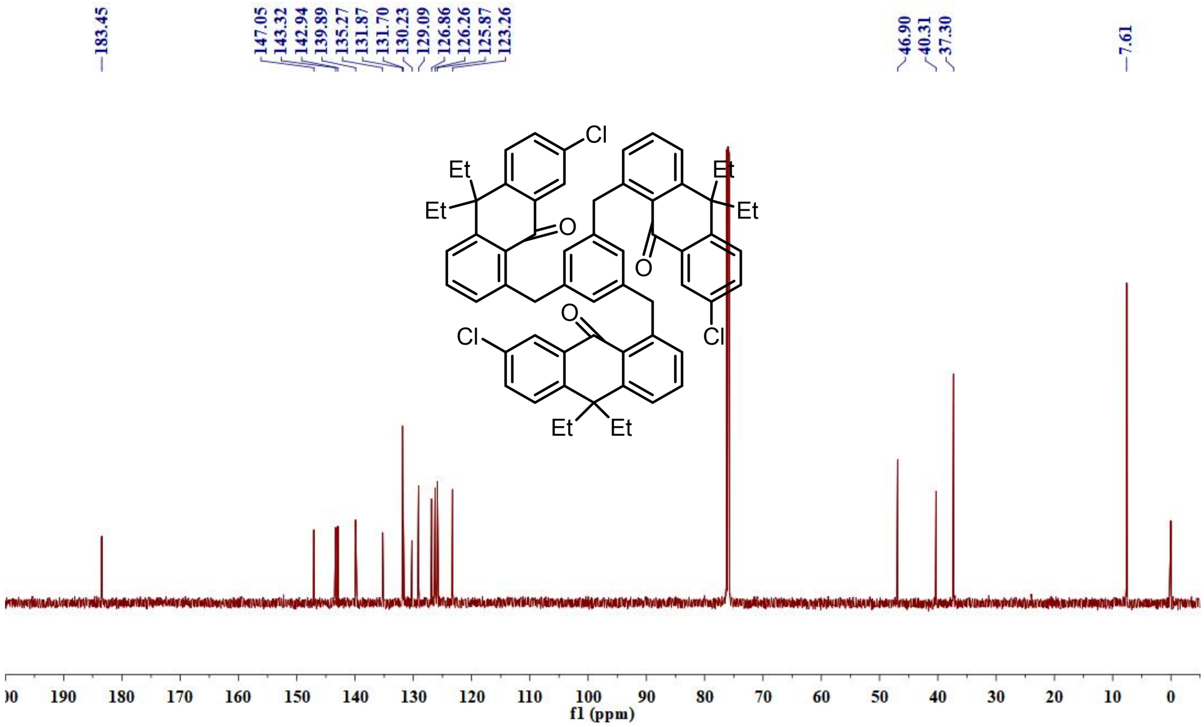

Fig. S110. ^{13}C NMR spectrum of 8,8',8''-(benzene-1,3,5-triyltris(methylene))tris(2-chloro-10,10-diethylanthracen-9(10*H*)-one) (4b) (151 MHz, $CDCl_3$, 298 K).

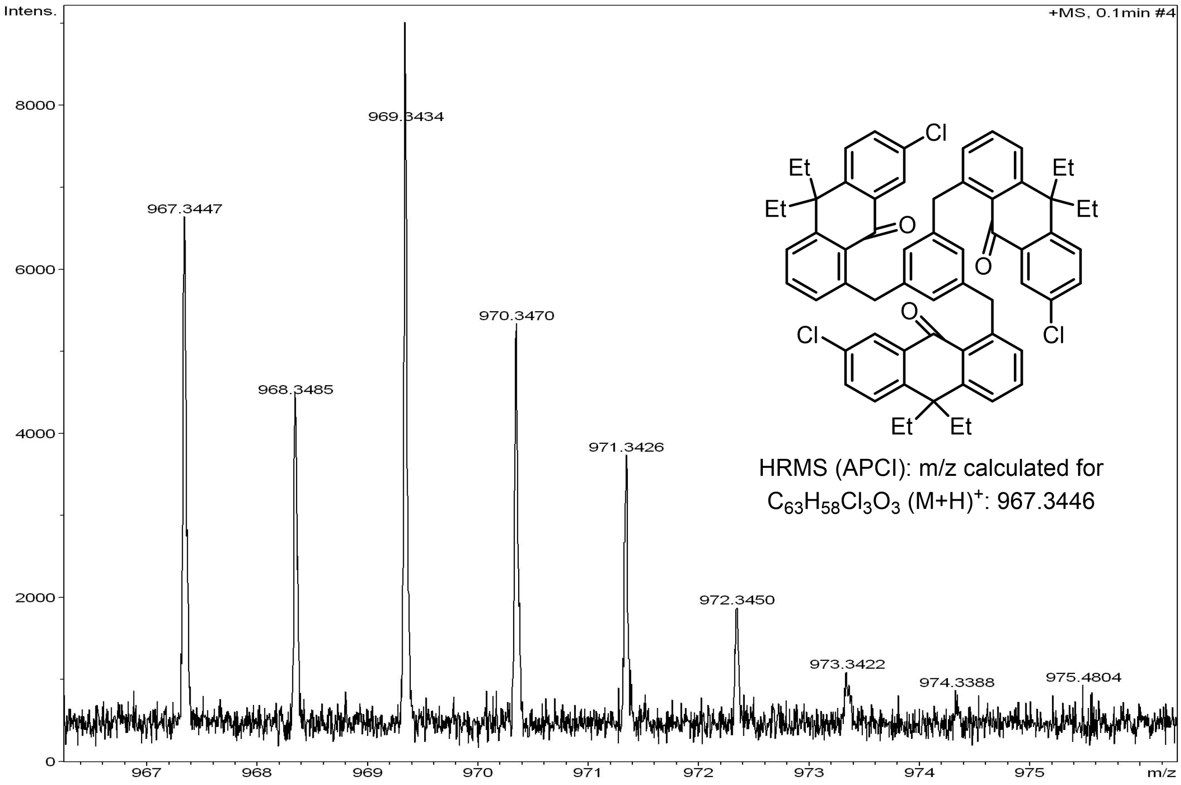

Fig. S111. HRMS (APCI, positive mode, methanol/chloroform) spectrum of 8,8',8''-(benzene-1,3,5-triyltris(methylene))tris(2-chloro-10,10-diethylanthracen-9(10*H*)-one) (4b).

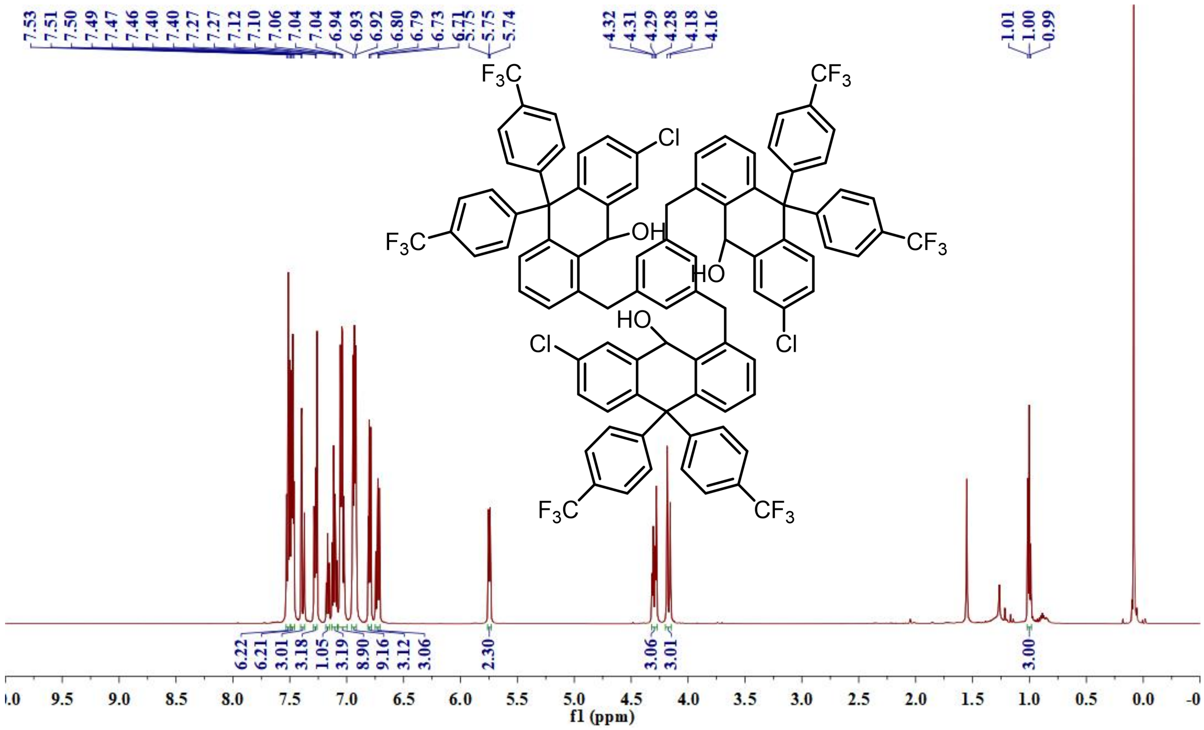

Fig. S112. **^{1}H NMR spectrum of 8,8',8''-(benzene-1,3,5-triyltris(methylene))tris(2-chloro-10,10-bis(4-(trifluoromethyl)phenyl)-9,10-dihydroanthracen-9-ol) (3a) (600 MHz, $CDCl_3$, 298 K).**

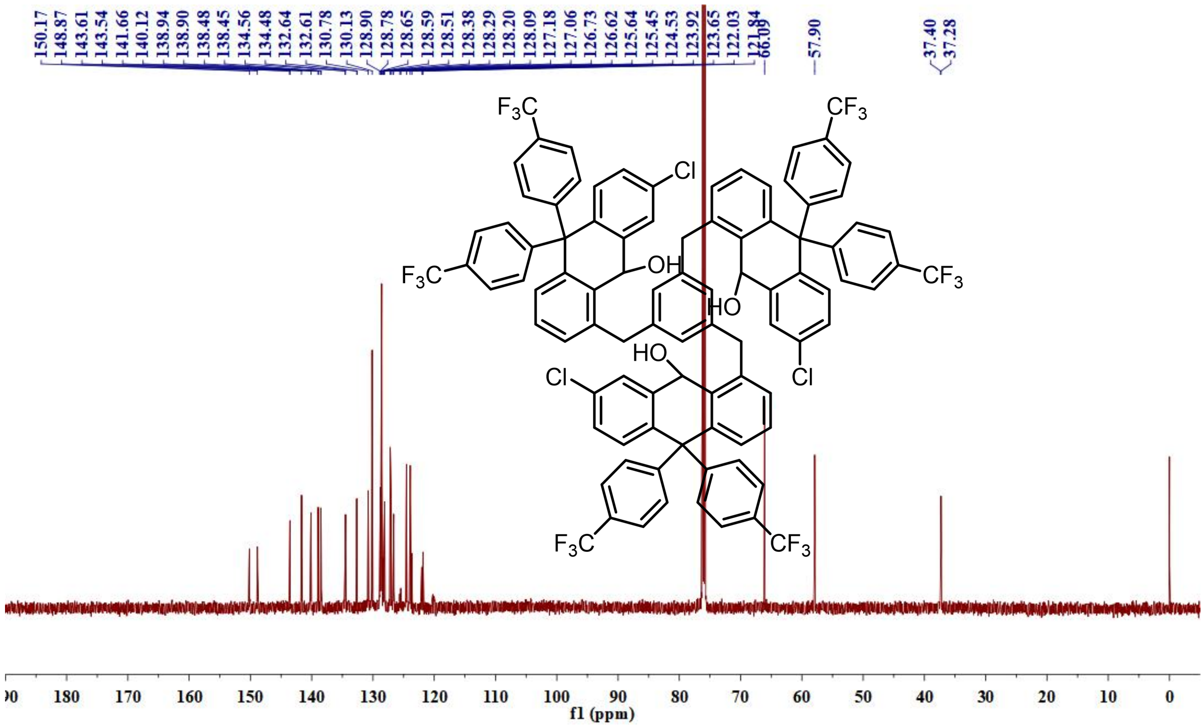

Fig. S113. **^{13}C NMR spectrum of 8,8',8''-(benzene-1,3,5-triyltris(methylene))tris(2-chloro-10,10-bis(4-(trifluoromethyl)phenyl)-9,10-dihydroanthracen-9-ol) (3a) (151 MHz, $CDCl_3$, 298 K).**

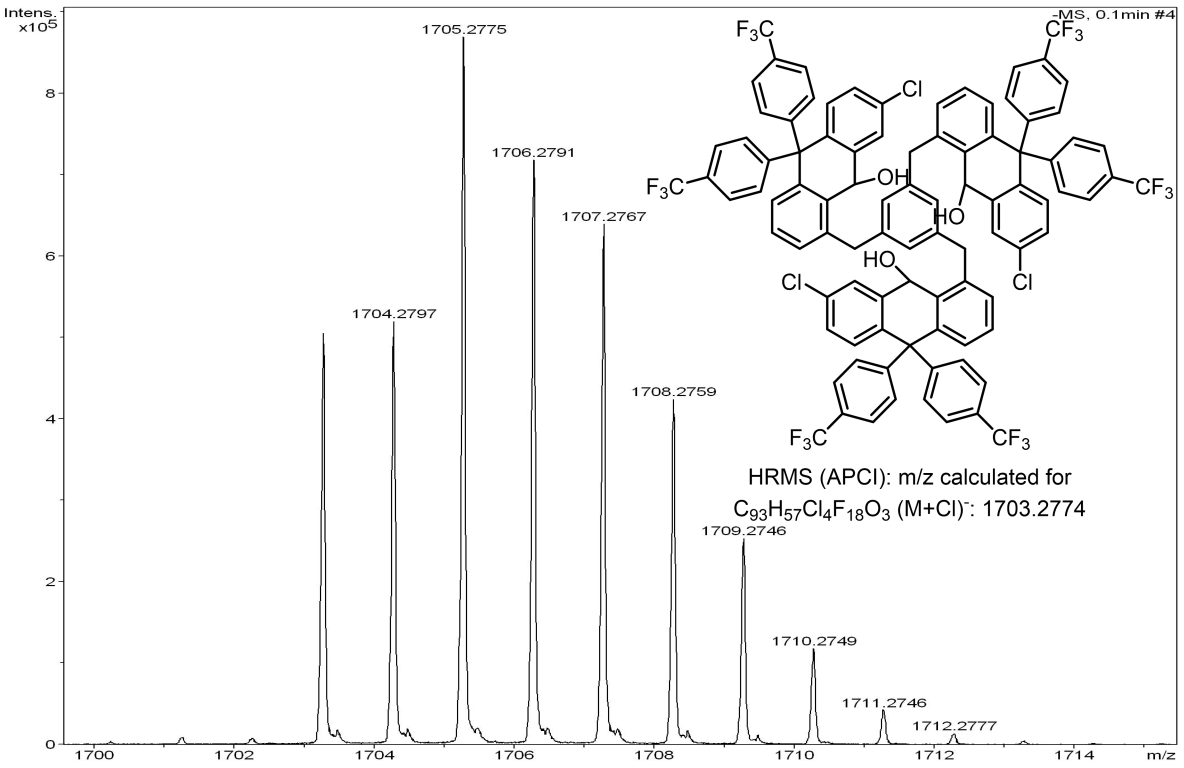

Fig. S114. HRMS (APCI, negative mode, methanol/chloroform) spectrum of 8,8',8''-(benzene-1,3,5-triyltris(methylene))tris(2-chloro-10,10-bis(4-(trifluoromethyl)phenyl)-9,10-dihydroanthracen-9-ol) (3a).

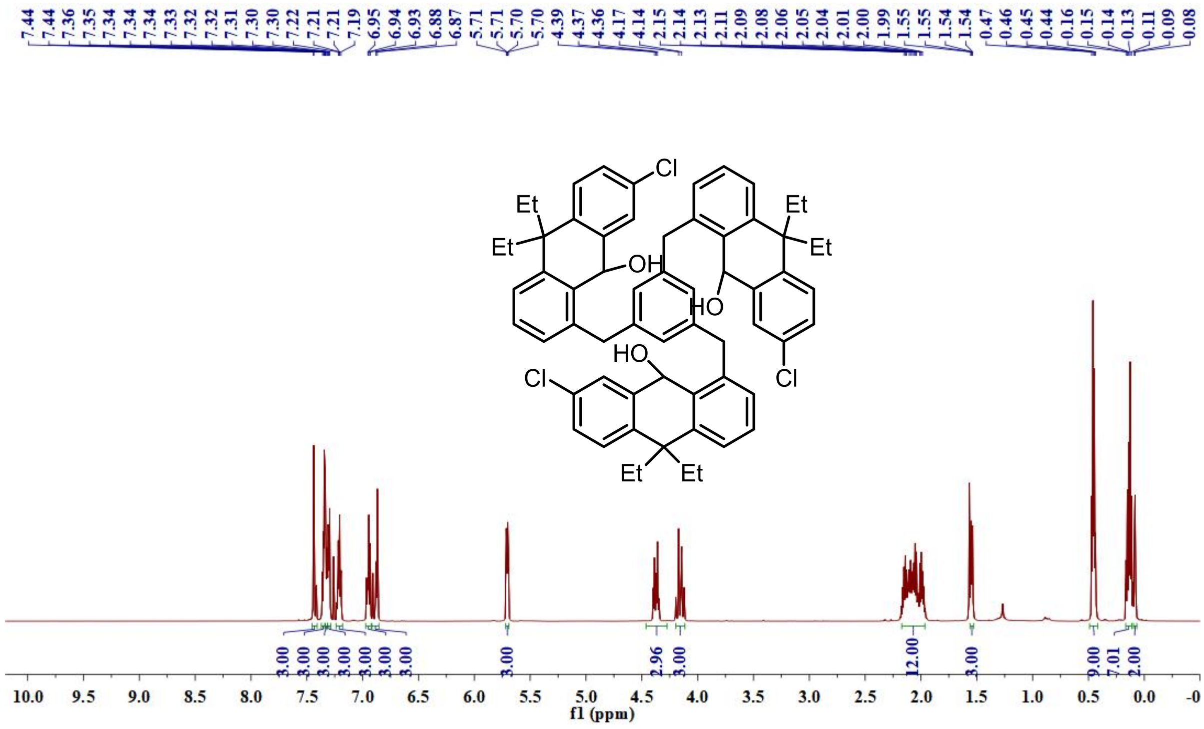

Fig. S115. ^{1}H NMR spectrum of 8,8',8''-(benzene-1,3,5-triyltris(methylene))tris(2-chloro-10,10-diethyl-9,10-dihydroanthracen-9-ol) (3b) (600 MHz, $CDCl_3$, 298 K).

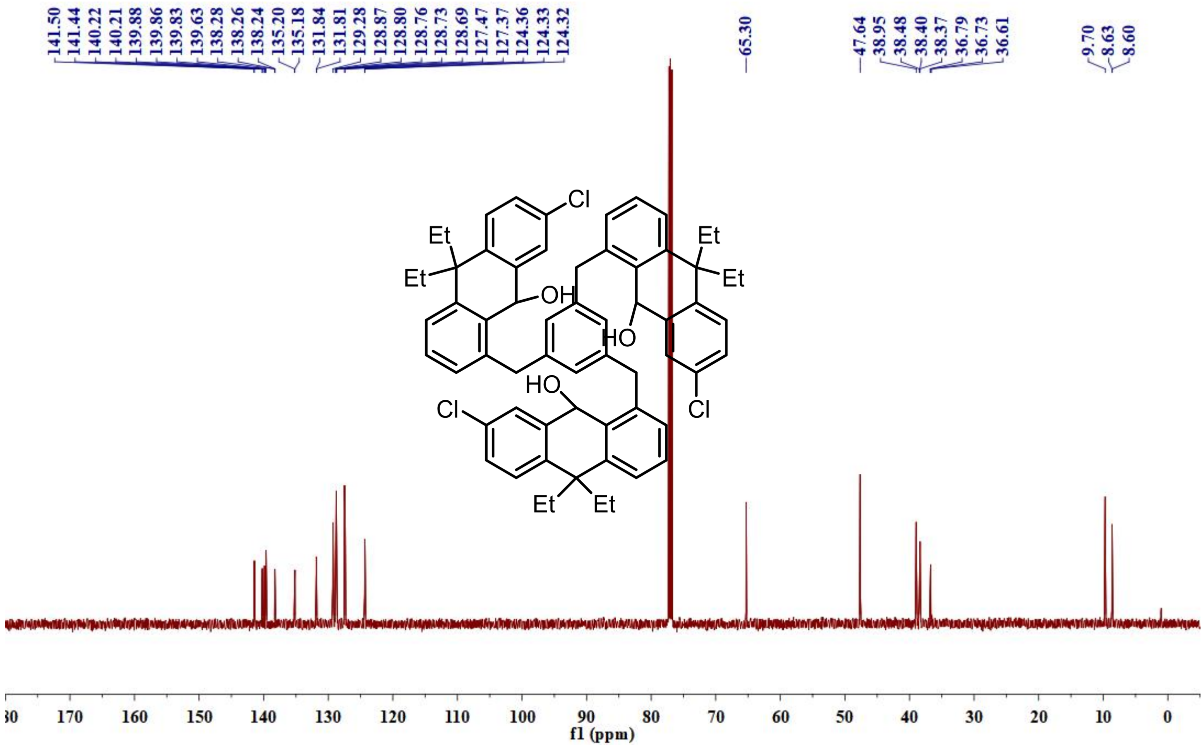

Fig. S116. ^{13}C **NMR spectrum of 8,8',8''-(benzene-1,3,5-triyltris(methylene))tris(2-chloro-10,10-diethyl-9,10-dihydroanthracen-9-ol) (3b) (151 MHz, $CDCl_3$, 298 K).**

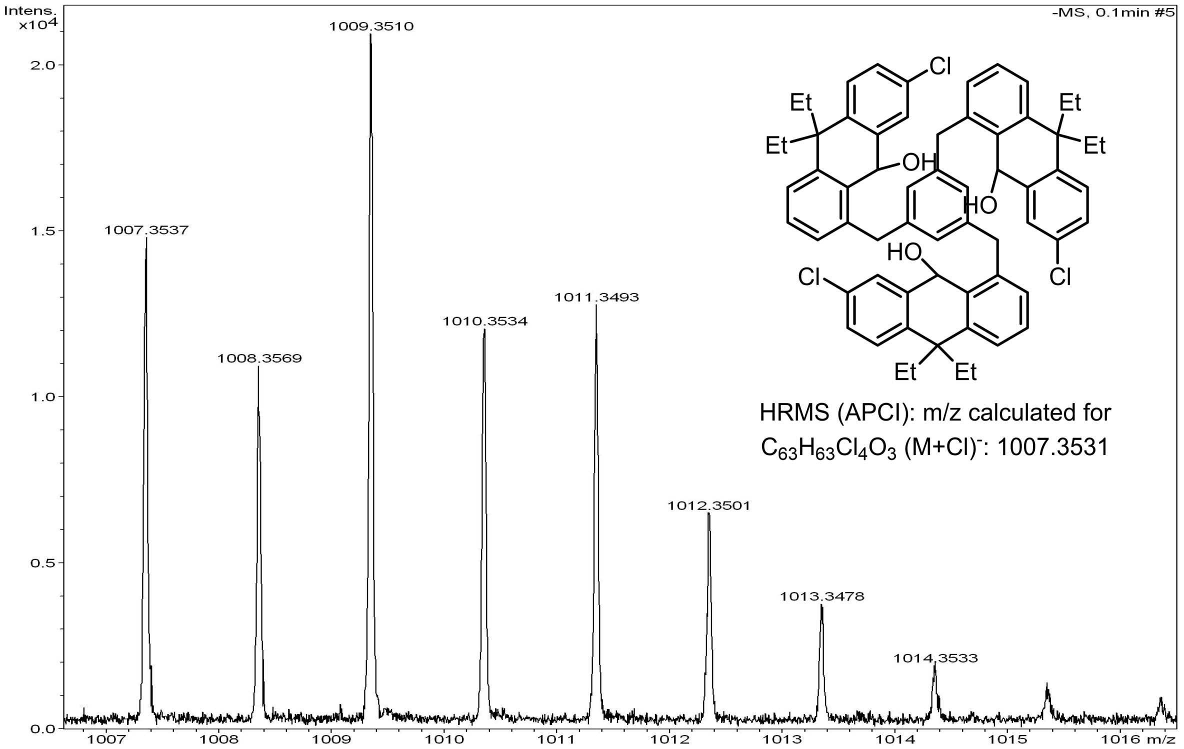

Fig. S117. HRMS (APCI, negative mode, methanol/chloroform) spectrum of 8,8',8''-(benzene-1,3,5-triyltris(methylene))tris(2-chloro-10,10-diethyl-9,10-dihydroanthracen-9-ol) (3b).

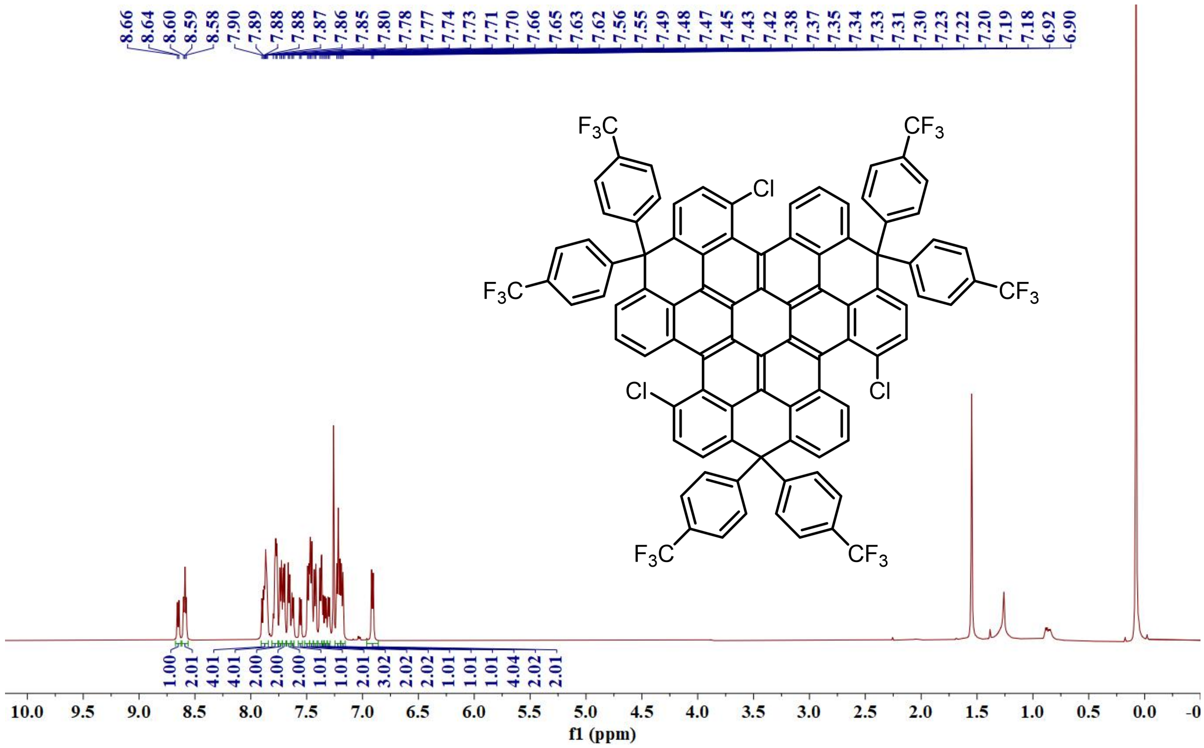

Fig. S118. **^{1}H NMR spectrum of 3,10,17-trichloro-7,7,14,14,21,21-hexakis(4-(trifluoromethyl)phenyl)-14,21-dihydro-7*H*-triphenaleno[1,2,3,4,5-*fghij*:1',2',3',4',5'-*pqrst*:1'',2'',3'',4'',5''-*za*$_1$*b*$_1$*c*$_1$*d*$_1$]trinaphthylene (2a) (600 MHz, $CDCl_3$, 298 K).**

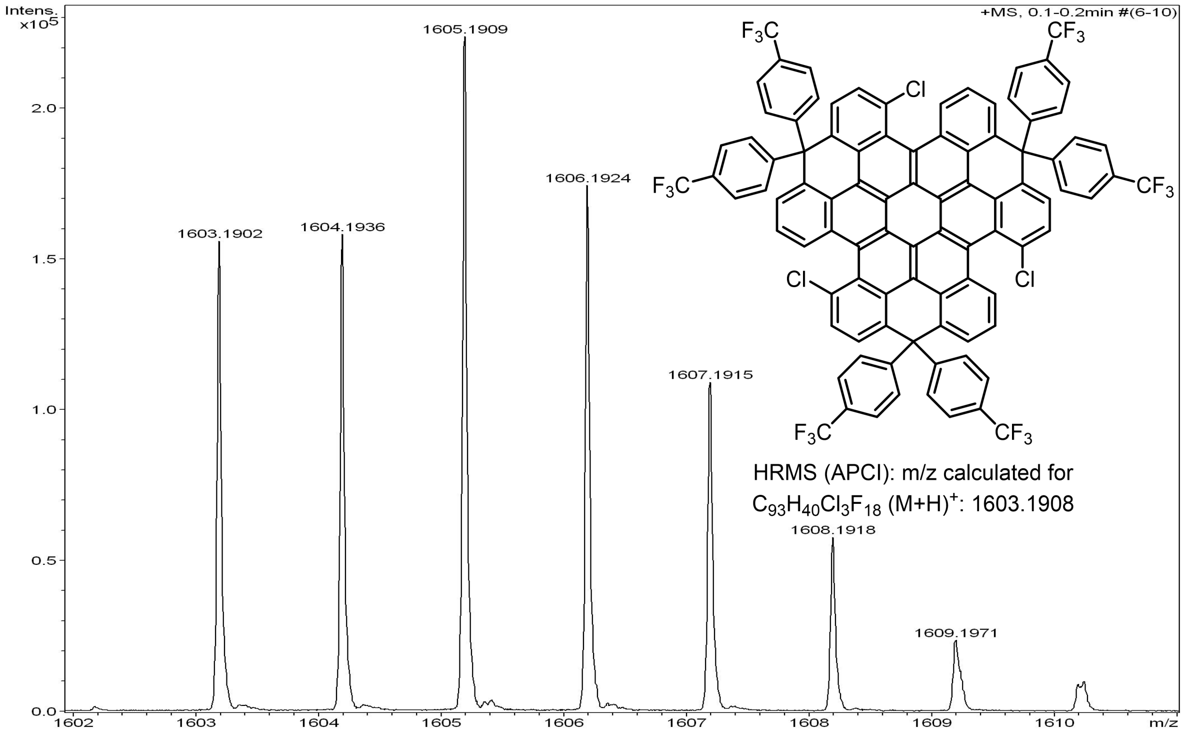

Fig. S119. HRMS (APCI, positive mode, methanol/chloroform) spectrum of 3,10,17-trichloro-7,7,14,14,21,21-hexakis(4-(trifluoromethyl)phenyl)-14,21-dihydro-7*H*-triphenaleno[1,2,3,4,5-*fghij*:1',2',3',4',5'-*pqrst*:1'',2'',3'',4'',5''-*za*$_1$*b*$_1$*c*$_1$*d*$_1$]trinaphthylene (2a).

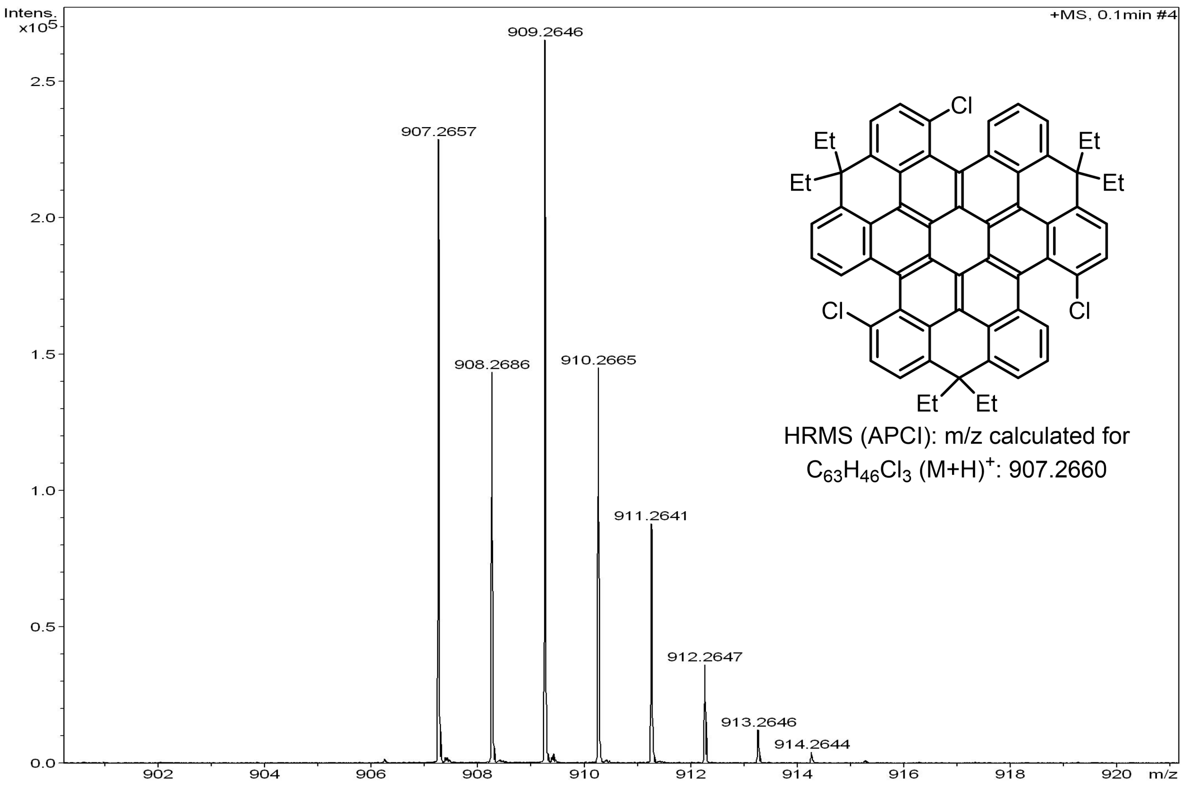

Fig. S120. HRMS (APCI, positive mode, methanol/chloroform) spectrum of 3,10,17-trichloro-7,7,14,14,21,21-hexaethyl-14,21-dihydro-7H-triphenaleno[1,2,3,4,5-fghij:1',2',3',4',5'-pqrst:1'',2'',3'',4'',5''-za1b1c1d1]trinaphthylene (2b).

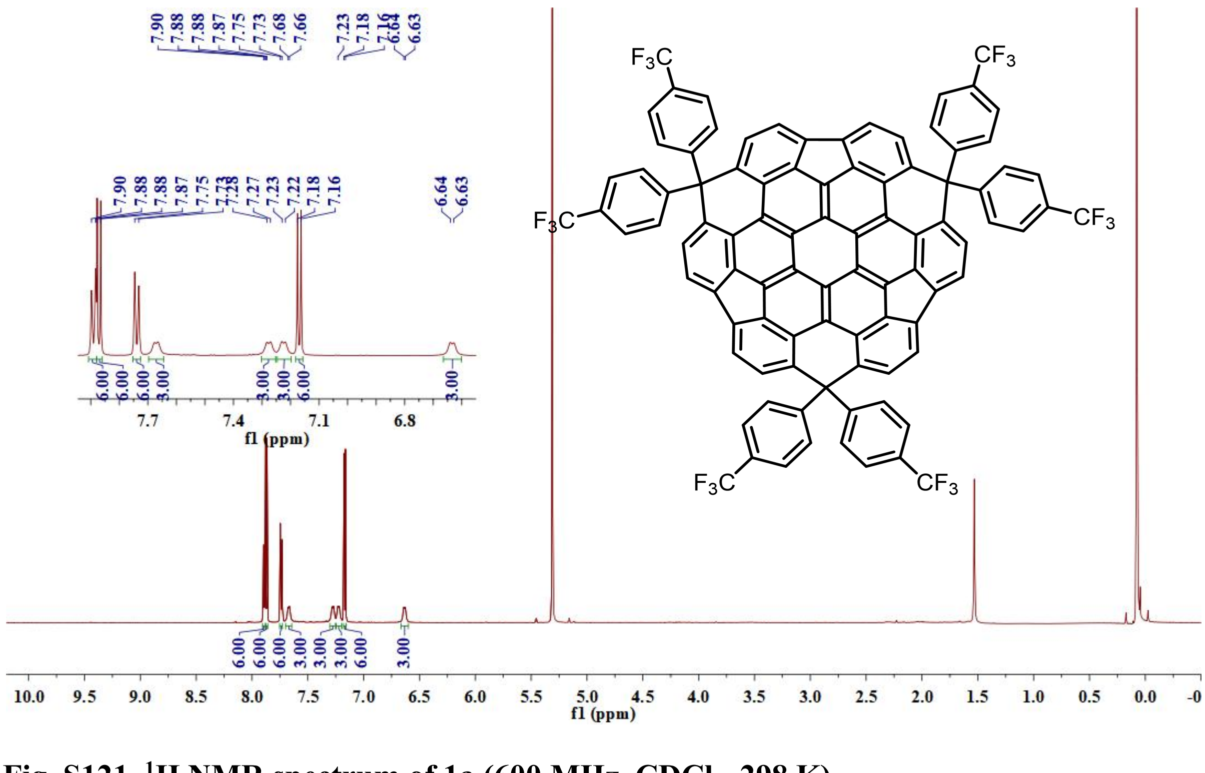

Fig. S121. ^{1}H NMR spectrum of 1a (600 MHz, $CDCl_2$, 298 K).

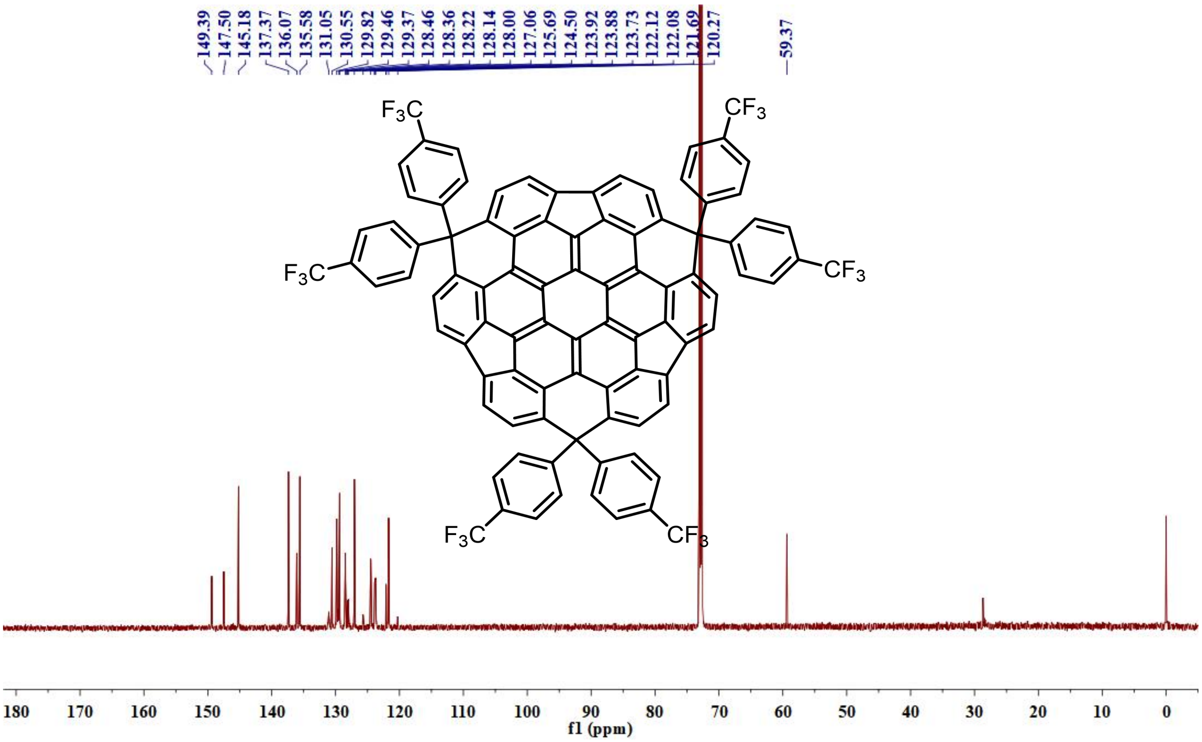

Fig. S122. ^{13}**C NMR spectrum of 1a (151 MHz, CDCl$_3$, 298K).**

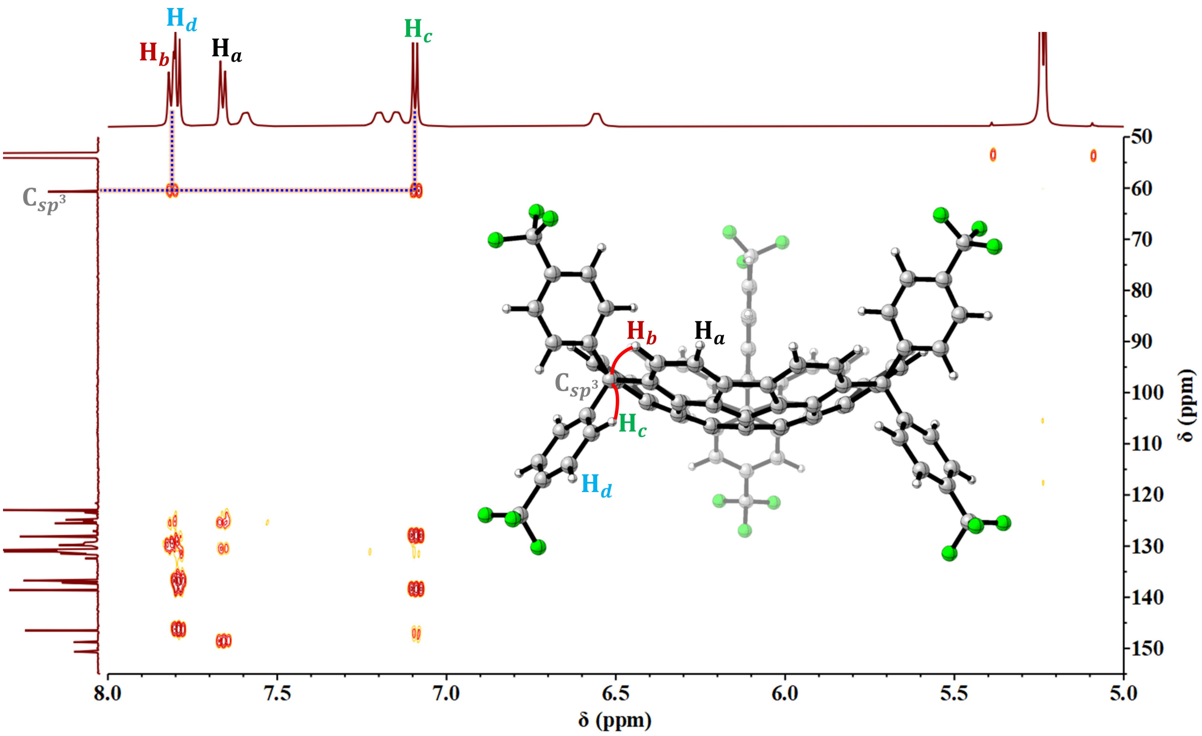

Fig. S123. ^{1}**H-**^{13}**C HMBC NMR spectrum of 1a (600 MHz, CD$_2$Cl$_2$, 298K).**

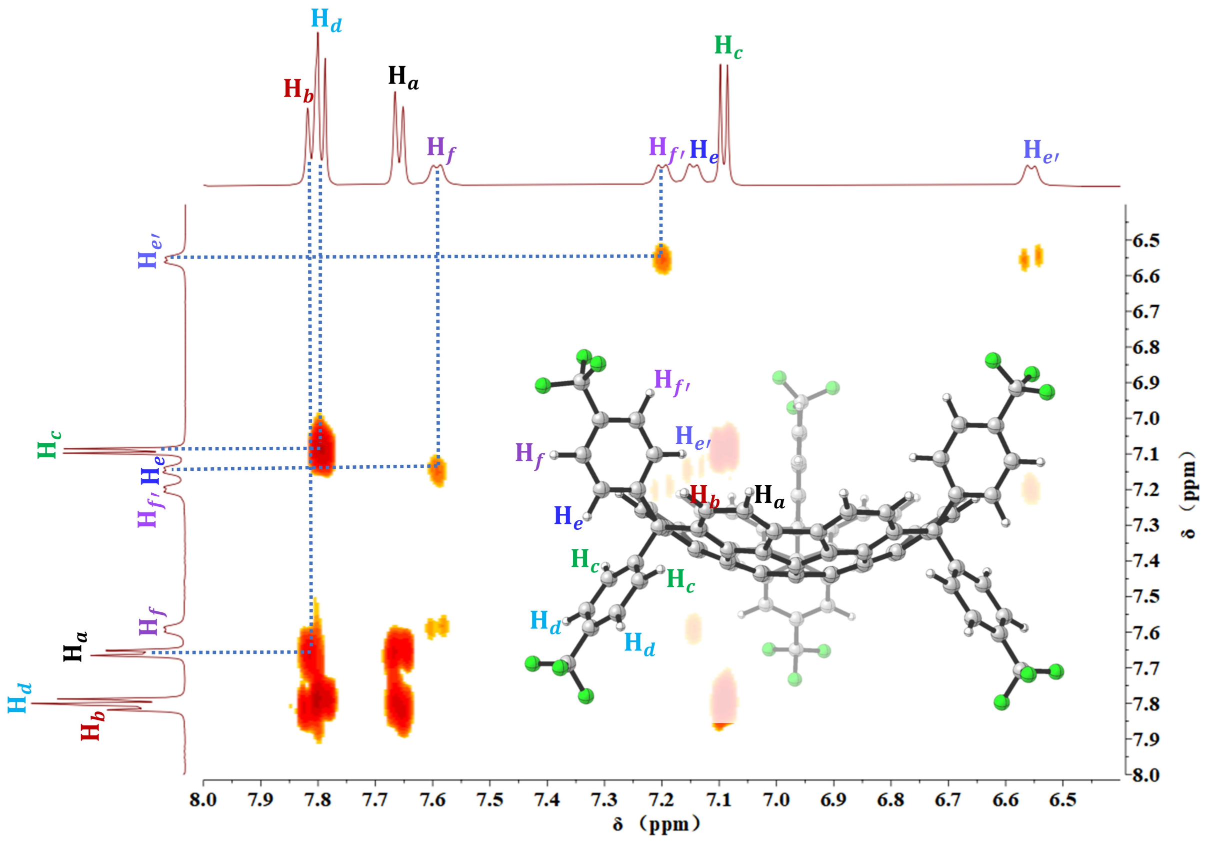

Fig. S124. ^{1}H-^{1}H **COSY NMR spectrum of 1a (600 MHz,** CD_2Cl_2**, 298K).**

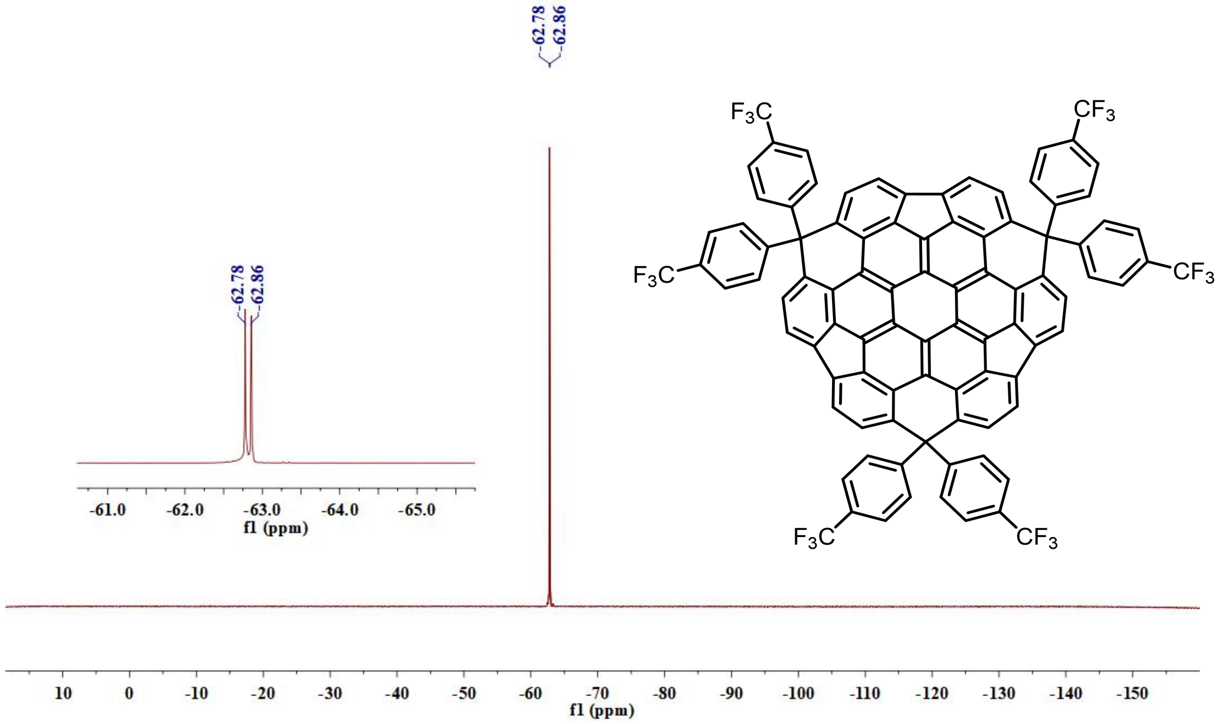

Fig. S125. ^{19}F **NMR spectrum of 1a (375 MHz,** $CDCl_3$**, 298K).**

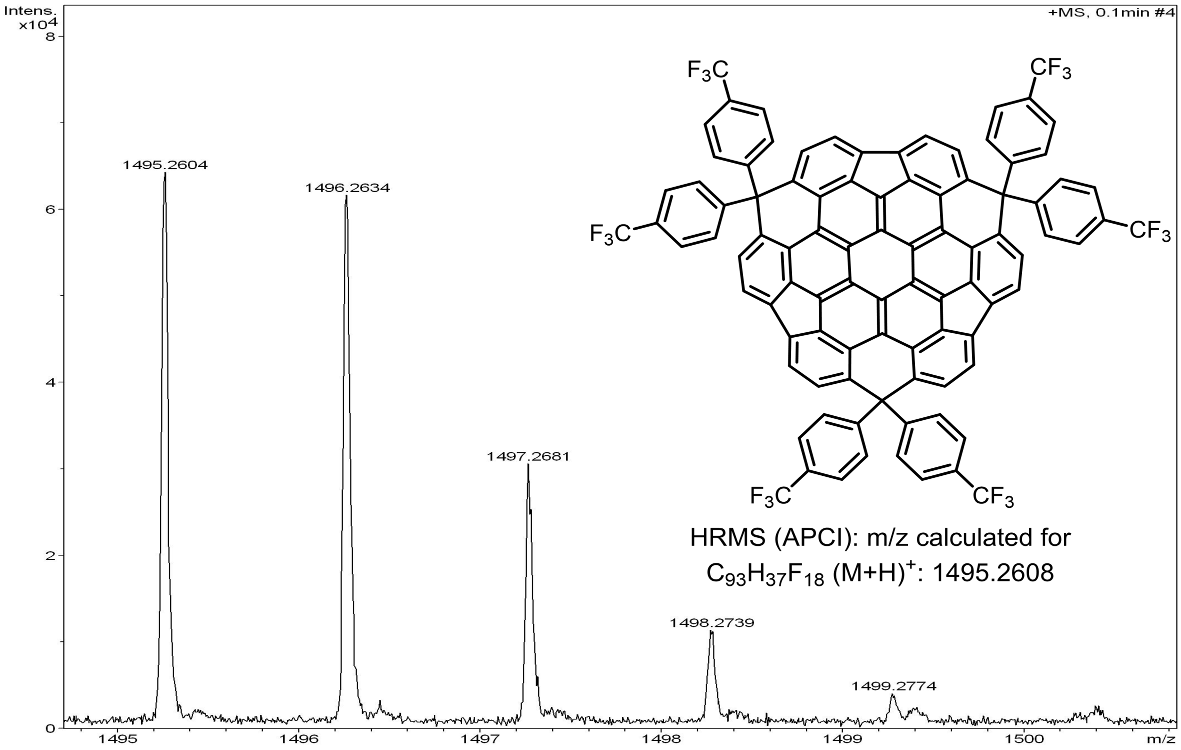

Fig. S126. HRMS (APCI, positive mode, methanol/chloroform) spectrum of 1a.

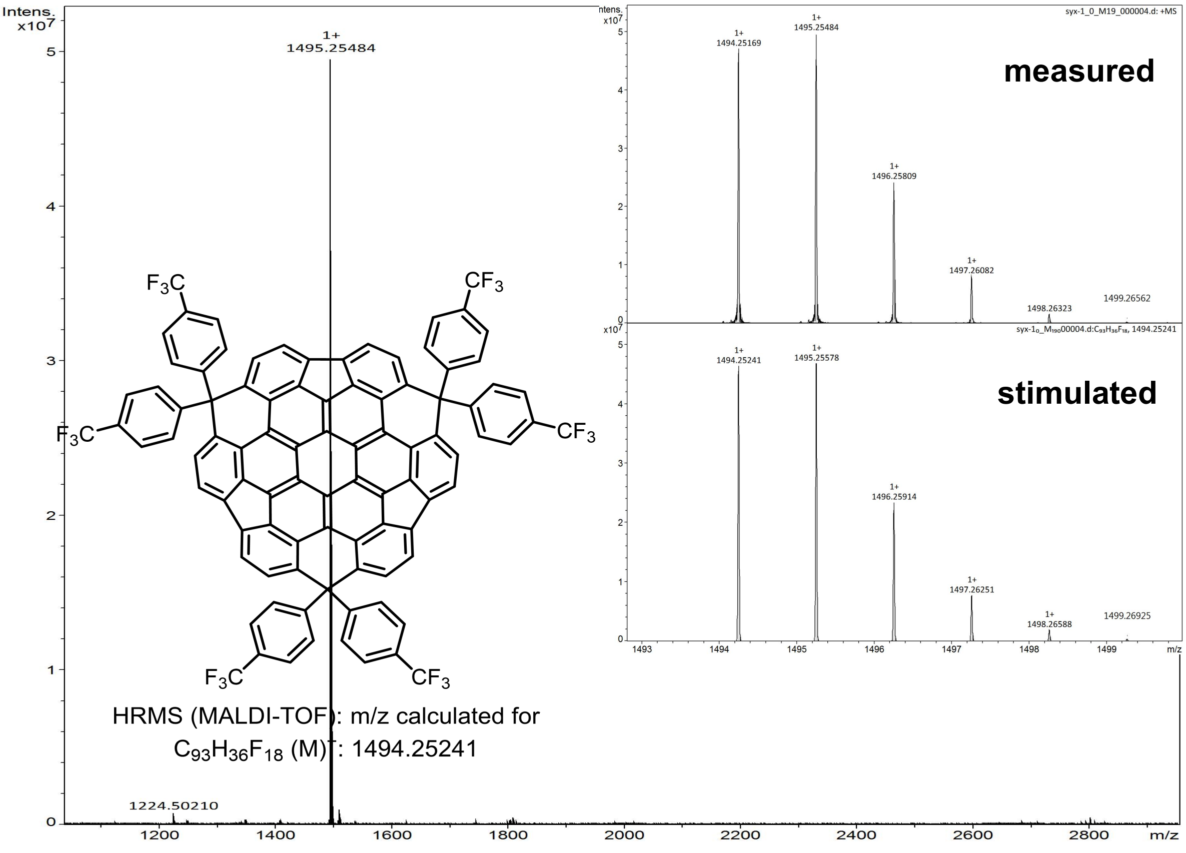

Fig. S127. FT-ICR HRMS (MALDI-TOF, positive mode, chloroform/DCTB) spectrum of 1a.

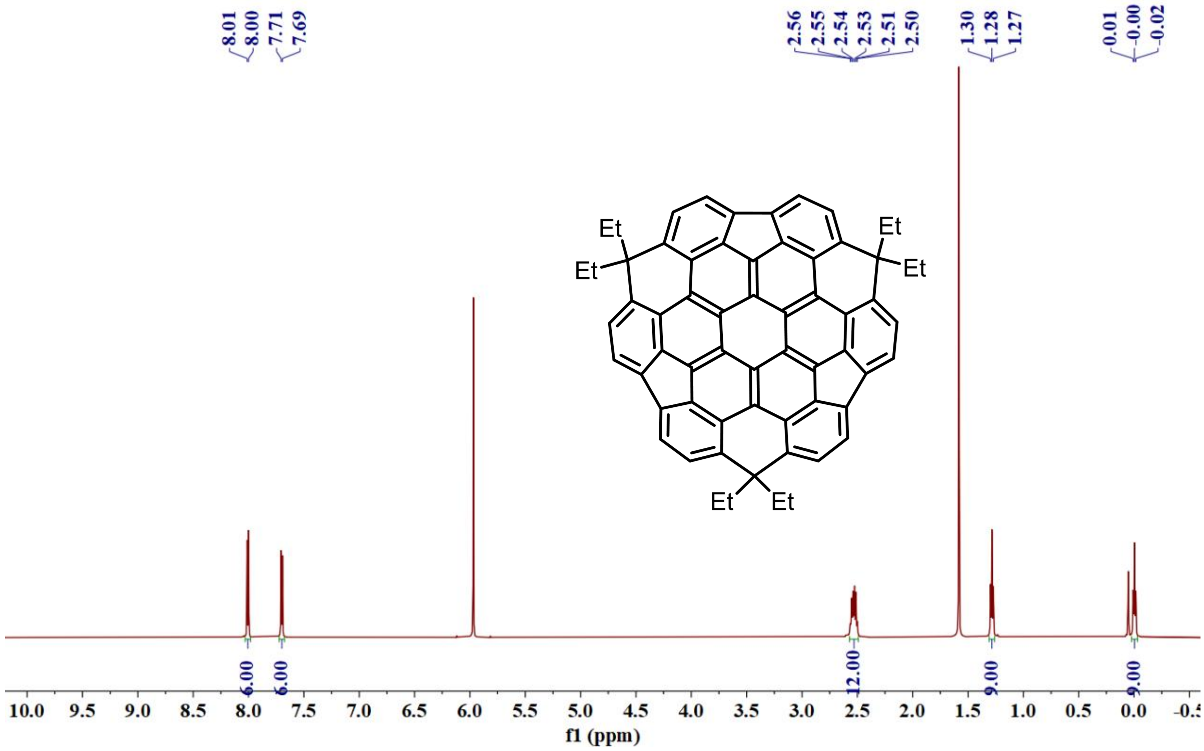

Fig. S128. ^{1}H NMR spectrum of 1b (600 MHz, $C_2D_2Cl_4$, 298 K).

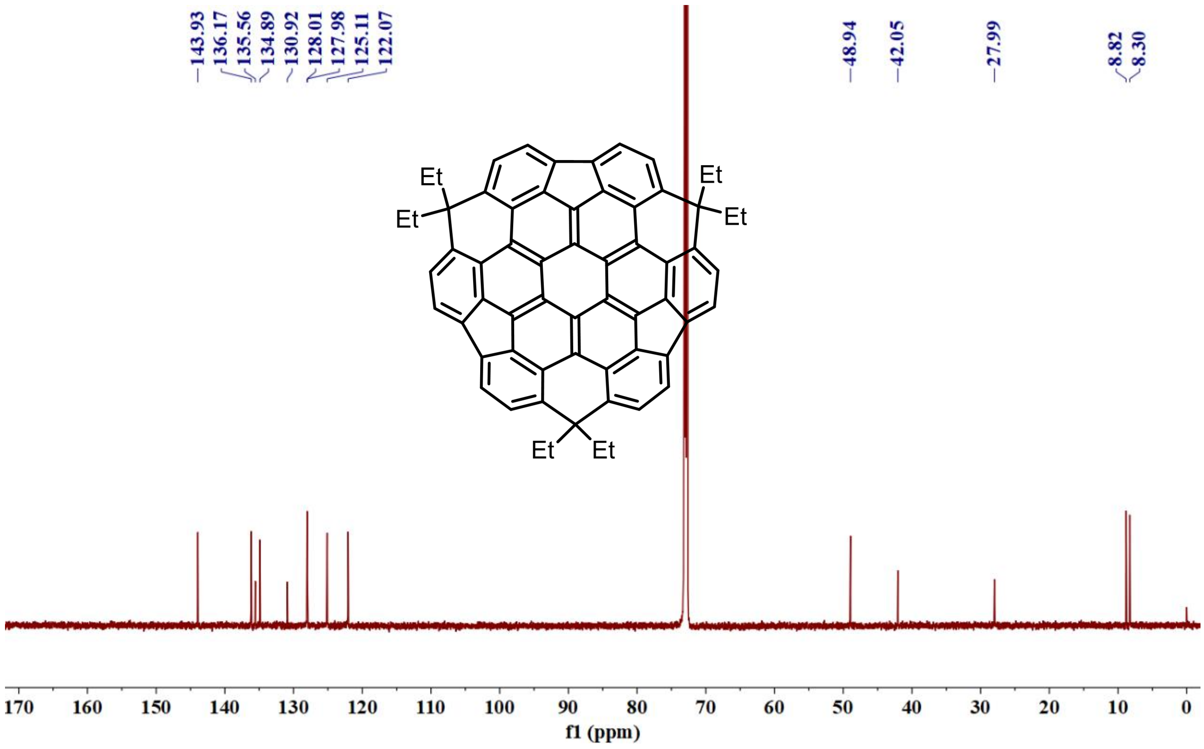

Fig. S129. ^{13}C NMR spectrum of 1b (151 MHz, $C_2D_2Cl_4$, 298 K).

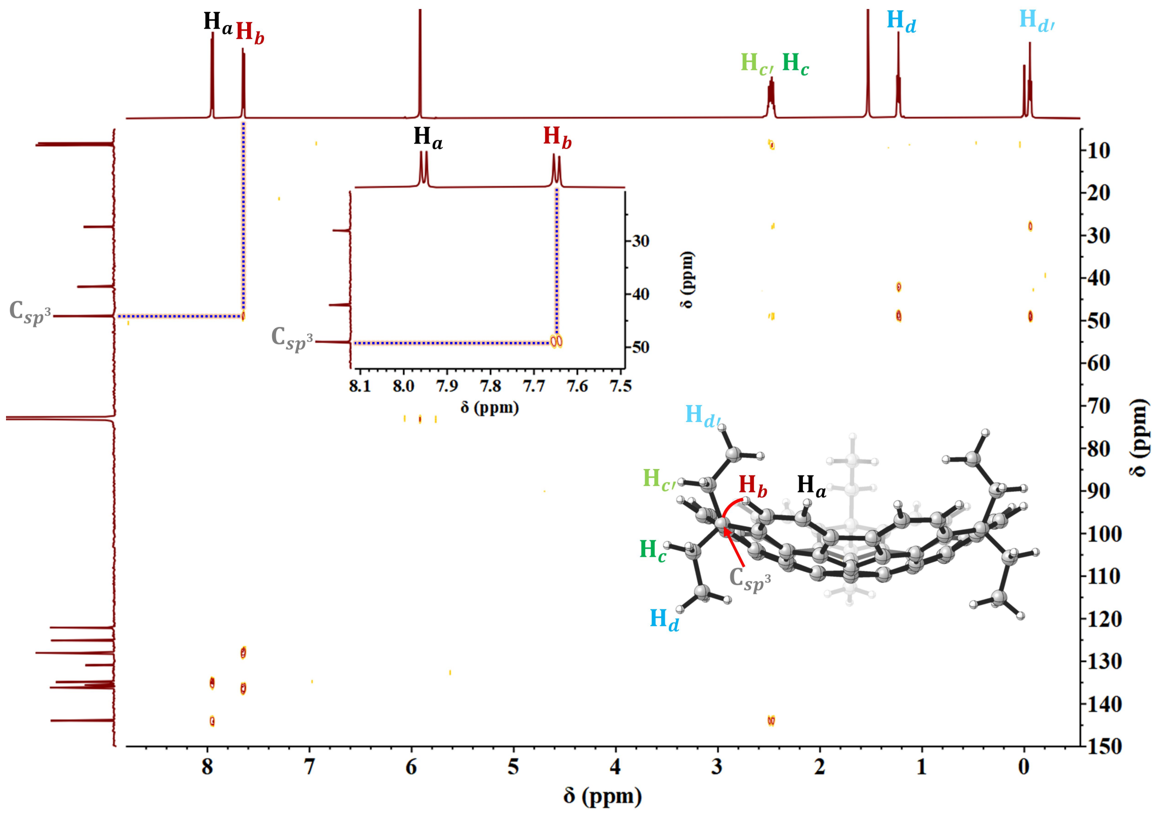

Fig. S130. ^1H-^{13}C HMBC NMR spectrum of 1b (600 MHz, $C_2D_2Cl_4$, 298 K).

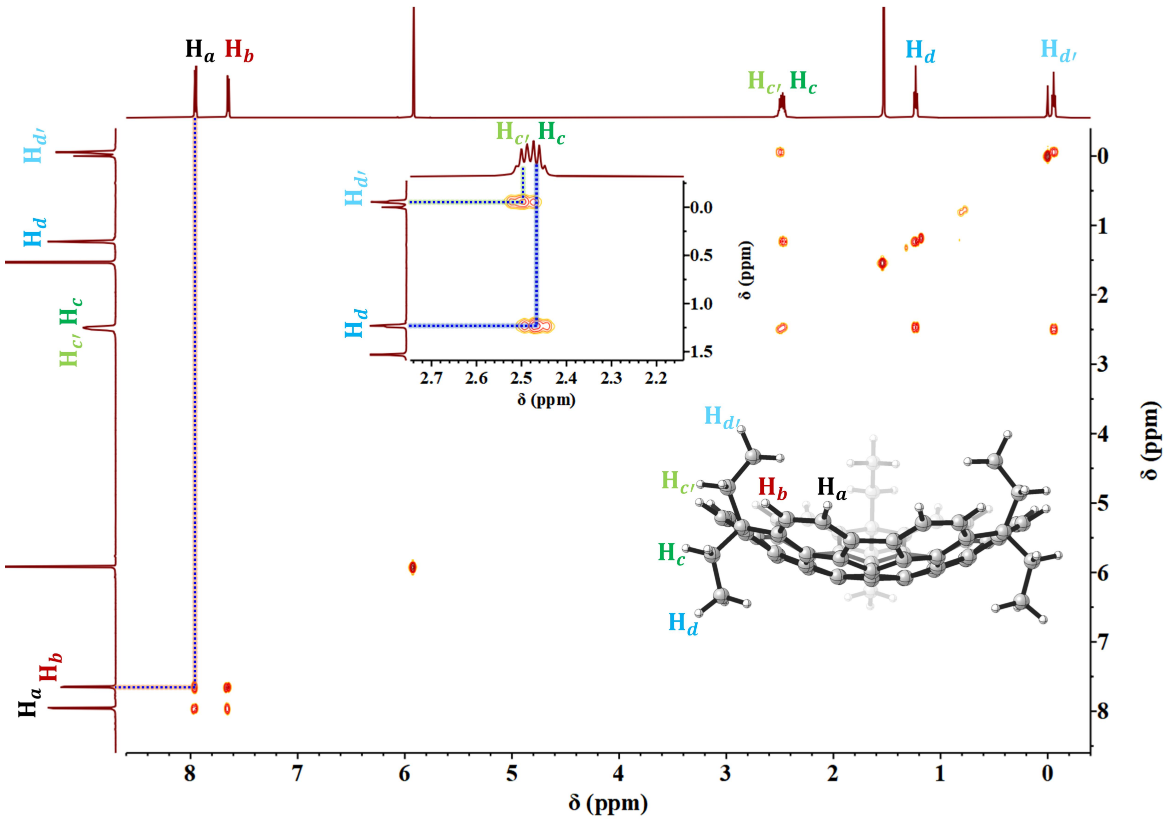

Fig. S131. ^1H-^1H COSY NMR spectrum of 1b (600 MHz, $C_2D_2Cl_4$, 298 K).

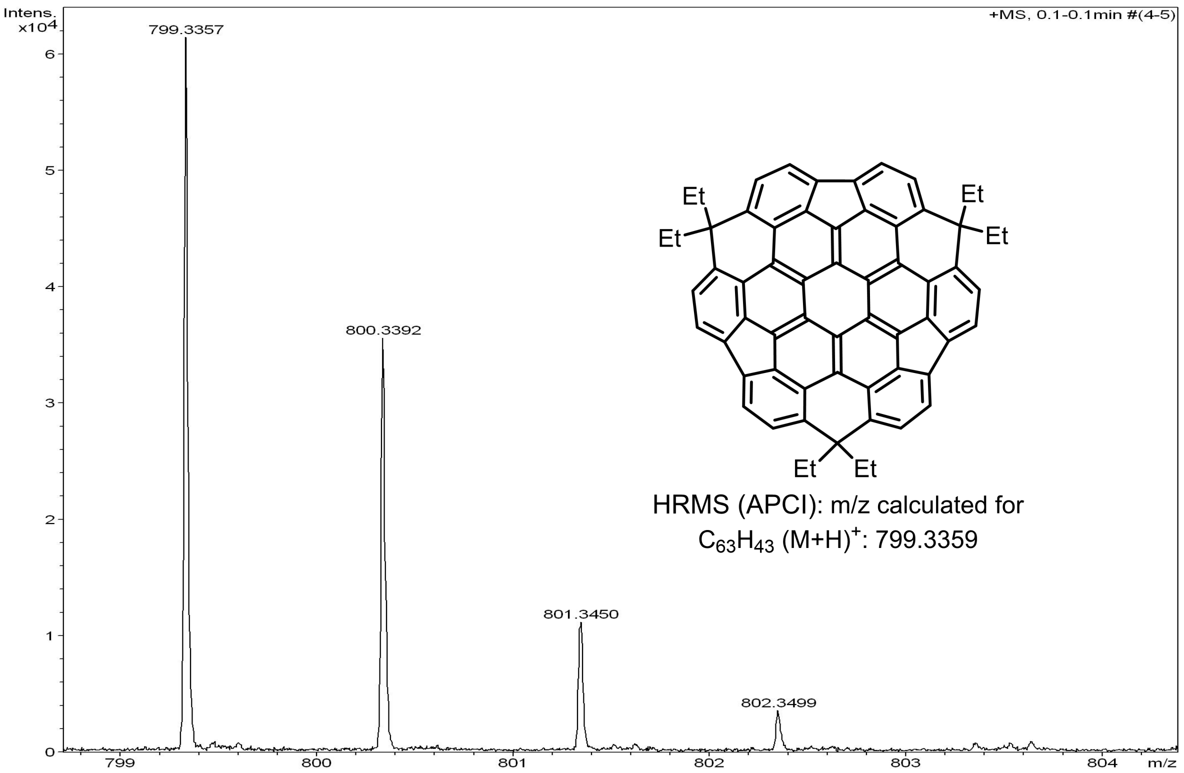

Fig. S132. HRMS (APCI, positive mode, methanol/chloroform) spectrum of 1b.

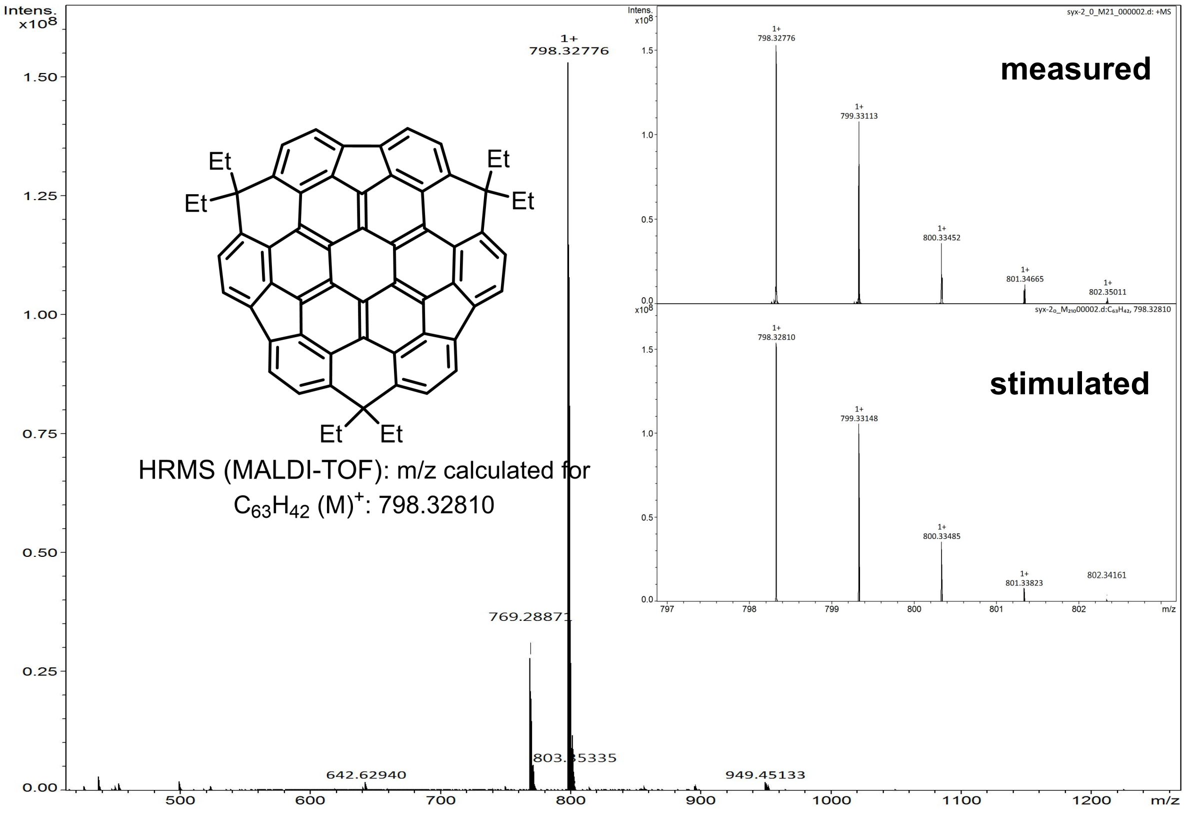

Fig. S133. FT-ICR HRMS (MALDI-TOF, positive mode, chloroform/DCTB) spectrum of 1b.

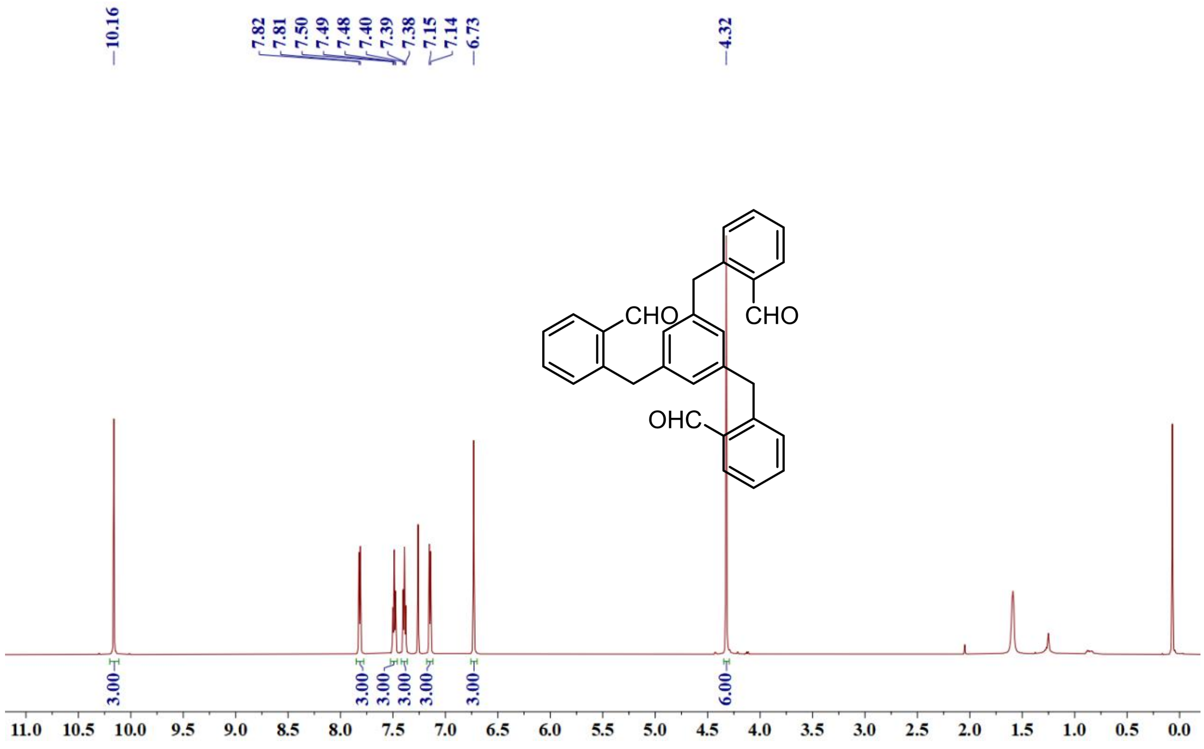

Fig. S134. ^{1}**H NMR spectrum of 2,2',2''-(benzene-1,3,5-triyltris(methylene))tribenzaldehyde (15) (600 MHz, $CDCl_3$, 298 K).**

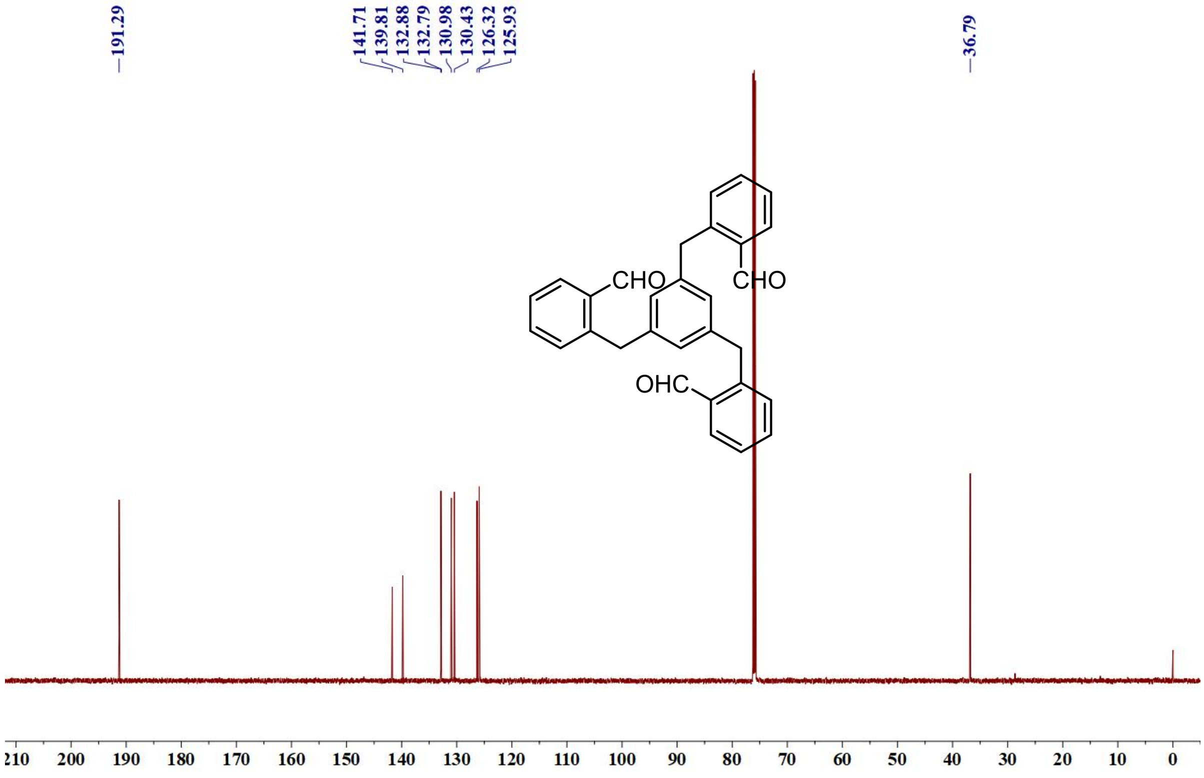

Fig. S135. ^{13}**C NMR spectrum of 2,2',2''-(benzene-1,3,5-triyltris(methylene))tribenzaldehyde (15) (151 MHz, $CDCl_3$, 298 K).**

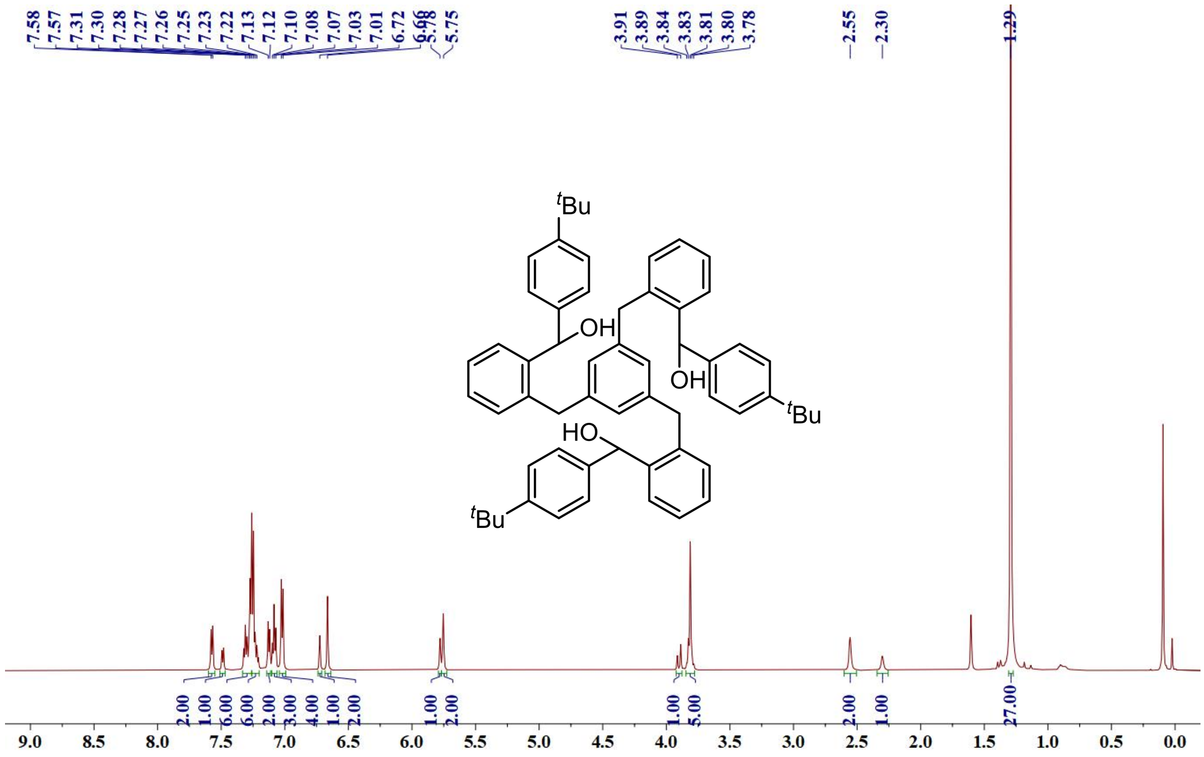

Fig. S136. ^{1}**H NMR spectrum of ((benzene-1,3,5-triyltris(methylene))tris(benzene-2,1-diyl))tris((4-(*tert*-butyl)phenyl)methanol) (16) (600 MHz, $CDCl_3$, 298 K).**

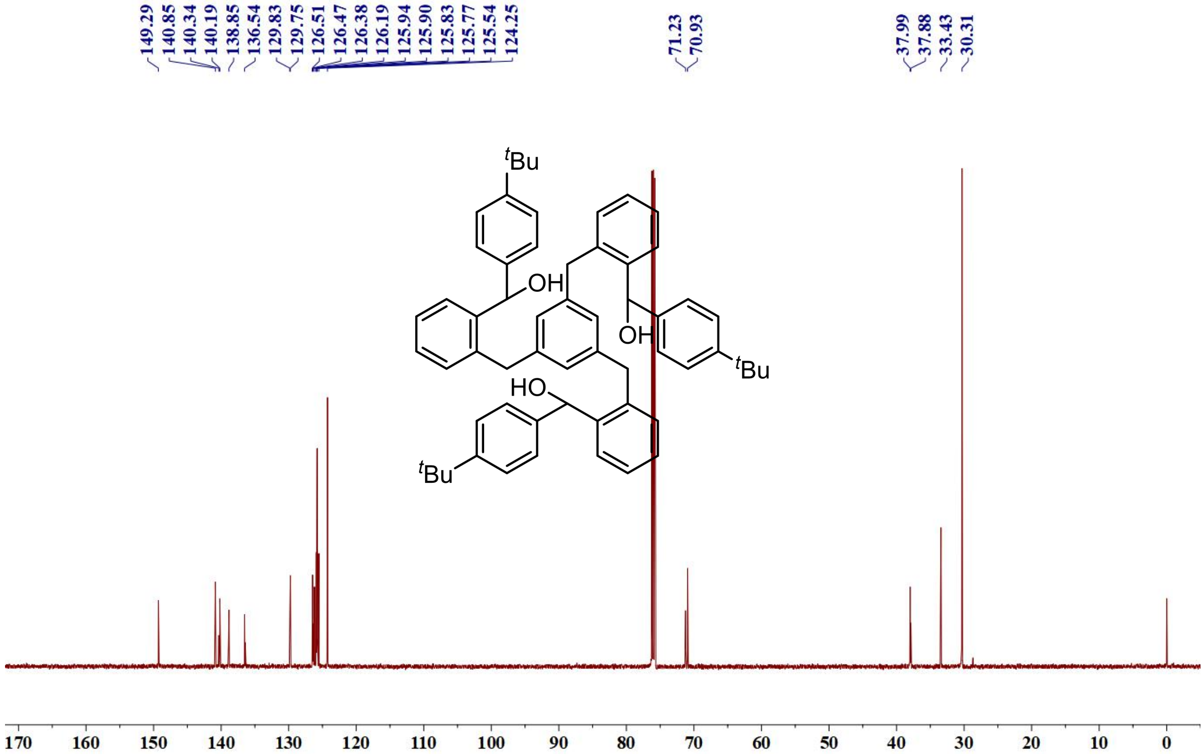

Fig. S137. ^{13}**C NMR spectrum of ((benzene-1,3,5-triyltris(methylene))tris(benzene-2,1-diyl))tris((4-(*tert*-butyl)phenyl)methanol) (16) (151 MHz, $CDCl_3$, 298 K).**

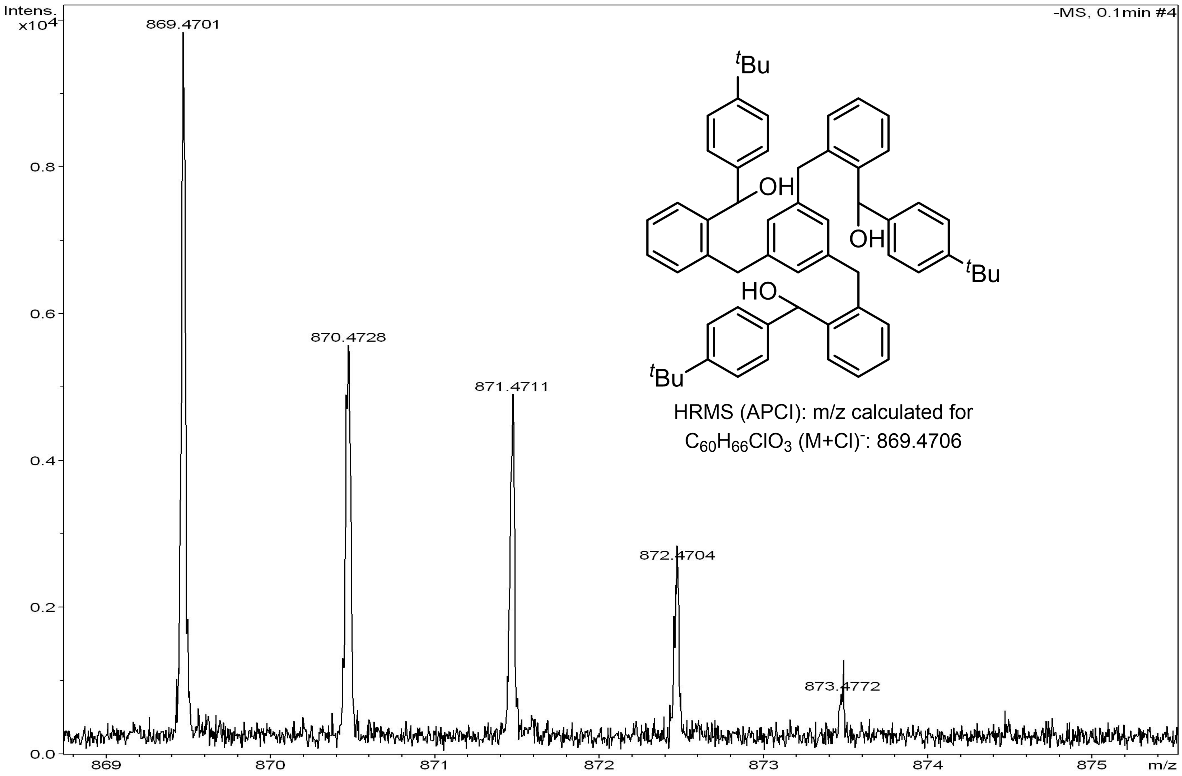

Fig. S138. HRMS (APCI, negative mode, methanol/chloroform) spectrum of ((benzene-1,3,5-triyltris(methylene))tris(benzene-2,1-diyl))tris((4-(*tert*-butyl)phenyl)methanol) (16).

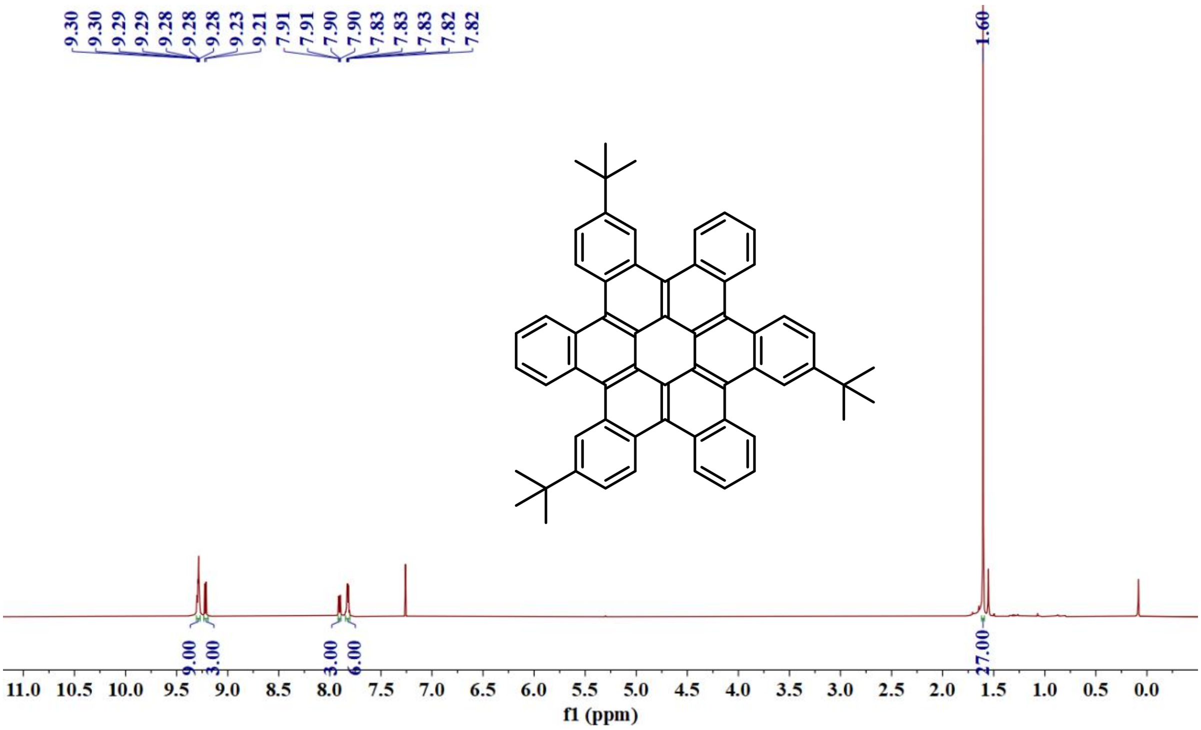

Fig. S139. ^{1}H NMR spectrum of 2,10,18-tri-*tert*-butyltrinaphtho[1,2,3,4-*fgh*:1',2',3',4'-*pqr*:1'',2'',3'',4''-*za*$_1$*b*$_1$]trinaphthylene (tri-*tert*-butylhexabenzocoronene, ^{t}Bu$_3$-HBC) (600 MHz, CDCl$_3$, 298 K).

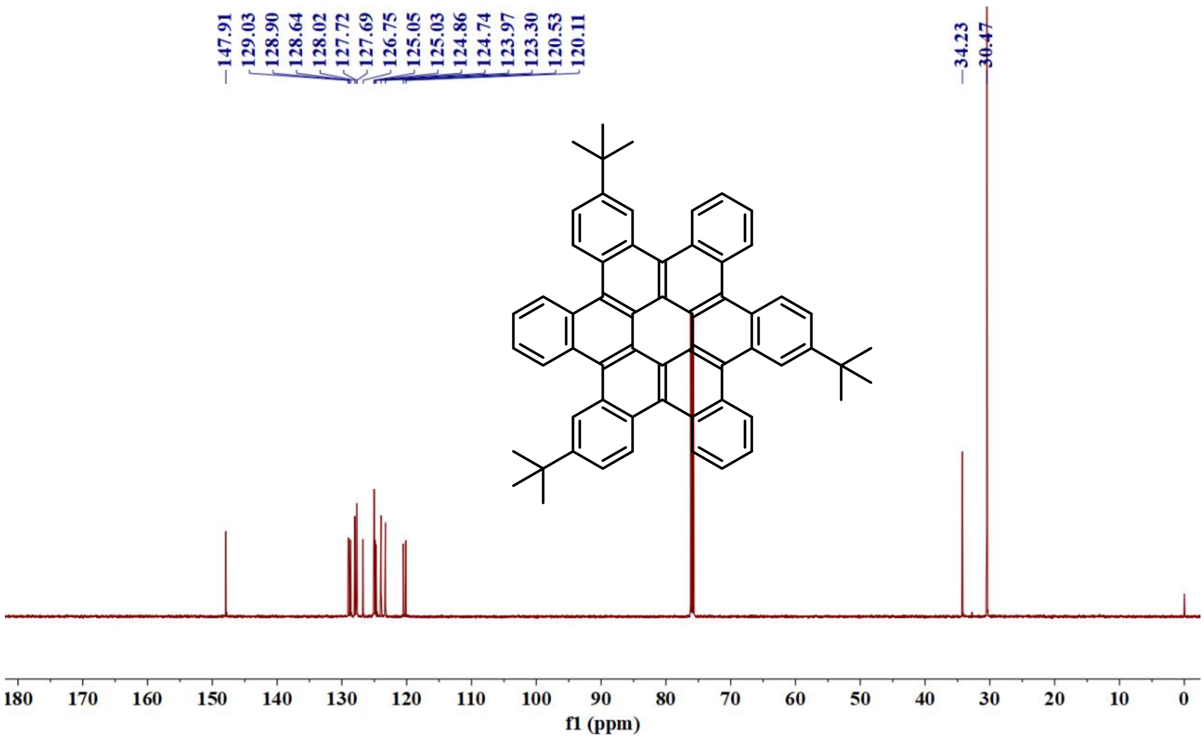

Fig. S140. ^{13}**C NMR spectrum of 2,10,18-tri-*tert*-butyltrinaphtho[1,2,3,4-*fgh*:1',2',3',4'-*pqr*:1'',2'',3'',4''-*za*$_1$*b*$_1$]trinaphthylene (tri-*tert*-butylhexabenzocoronene, ^{t}Bu$_3$-HBC) (151 MHz, CDCl$_3$, 298 K).**

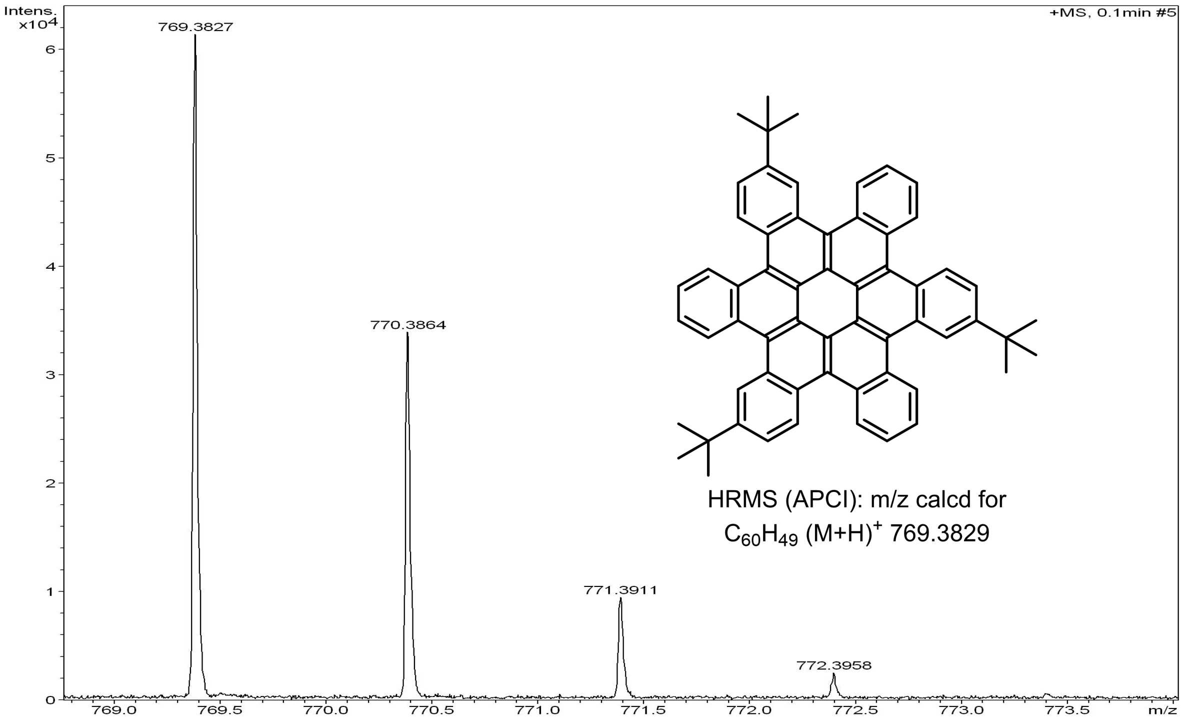

Fig. S141. HRMS (APCI, positive mode, methanol/chloroform) spectrum of 2,10,18-tri-*tert*-butyltrinaphtho[1,2,3,4-*fgh*:1',2',3',4'-*pqr*:1'',2'',3'',4''-*za*$_1$*b*$_1$]trinaphthylene (tri-*tert*-butylhexabenzocoronene, ^{t}Bu$_3$-HBC).

9. Optimized Cartesian Coordinates

Table S23. Optimized cartesian coordinates of 1a (M062X/6-31G(d,p)).

Atom	X	Y	Z
C	-0.738442	-1.263664	1.518477
C	-1.424946	0.002216	1.546483
C	-0.736985	1.267228	1.516266
C	0.702437	1.228787	1.549603
C	1.454043	0.000420	1.521245
C	0.701068	-1.226974	1.551832
C	2.807858	-0.000800	1.097182
C	3.446595	-1.225558	0.673839
C	2.690437	-2.388250	0.723582
C	1.360654	-2.369560	1.187944
C	-1.412011	2.439170	1.088178
C	-0.669311	3.603242	0.663055
C	0.715490	3.529939	0.715653
C	1.363155	2.370131	1.183988
C	-1.414945	-2.435439	1.092359
C	-2.793250	-2.375747	0.663409
C	-3.422042	-1.139449	0.710245
C	-2.742810	0.002731	1.177317
C	0.711304	-3.529137	0.721203
C	-0.673590	-3.600959	0.668942
C	-3.420610	1.144943	0.708209
C	-2.790335	2.380411	0.659228
C	2.692780	2.386436	0.719480
C	3.447123	1.222645	0.670961
C	4.655910	-1.265643	-0.068134
C	4.966727	-2.458285	-0.715067
C	4.112604	-3.600324	-0.730201
C	2.929076	-3.576284	-0.017896
C	2.933734	3.573455	-0.022840
C	4.119065	3.596087	-0.732427
C	4.972026	2.453016	-0.715188
C	4.656670	1.260671	-0.070373
C	-1.237734	4.668969	-0.082138
C	-0.359611	5.534622	-0.727701
C	1.056647	5.365633	-0.740112
C	1.626277	4.329326	-0.025626
C	-4.566498	0.757932	-0.036852
C	-5.175695	1.770821	-0.752247
C	-4.612625	3.081202	-0.736436
C	-3.425983	3.407428	-0.086228
C	-3.430224	-3.403291	-0.080201
C	-4.616489	-3.076753	-0.730932
C	-5.177943	-1.765704	-0.749068
C	-4.567460	-0.752321	-0.035493
C	1.620856	-4.330576	-0.019309
C	1.049922	-5.367317	-0.732090
C	-0.366499	-5.534827	-0.718945
C	-1.243422	-4.667189	-0.074485
C	5.547628	-0.002078	-0.093105
C	-2.776904	4.810629	-0.109046
C	-2.782723	-4.807258	-0.100623
C	6.425110	0.004669	-1.351741
C	6.421759	-0.010914	1.187355
C	-3.209600	5.573054	-1.368362
C	-3.219271	5.567349	1.169813
C	-3.216500	-5.571656	-1.358372
C	-3.225738	-5.560948	1.179786
C	5.787354	-0.025505	-2.601915
C	6.520003	-0.004565	-3.776425
C	7.914326	0.048508	-3.718945
C	8.560883	0.082694	-2.493123
C	7.815888	0.061674	-1.312315
C	6.777780	1.178081	1.829455
C	7.620101	1.168263	2.936909
C	8.120435	-0.036685	3.415714
C	7.781211	-1.230389	2.785858
C	6.937723	-1.213756	1.682065
C	-4.493363	5.367709	1.710778
C	-4.925625	6.097624	2.811992
C	-4.083780	7.041816	3.390215
C	-2.814541	7.256358	2.864273
C	-2.388517	6.523153	1.762221
C	-3.885414	6.789290	-1.329555
C	-4.247667	7.441056	-2.511026
C	-3.934375	6.874074	-3.735691
C	-3.256957	5.653447	-3.792767
C	-2.899500	5.013856	-2.618805
C	-2.394680	-6.513994	1.776152
C	-2.821430	-7.244473	2.879756
C	-4.091681	-7.029951	3.403228
C	-4.933845	-6.088453	2.821053
C	-4.500848	-5.361234	1.718368
C	-3.892416	-6.787757	-1.317076
C	-4.255353	-7.441575	-2.497203
C	-3.942616	-6.876801	-3.723029
C	-3.265011	-5.656396	-3.782609
C	-2.906863	-5.014778	-2.609966
C	8.698878	0.022559	-4.998833
F	8.775231	-1.218495	-5.504599
F	9.953937	0.458173	-4.827105
F	8.126512	0.788563	-5.940050
C	8.983256	-0.059538	4.644446

F	9.672225	1.081790	4.788041
F	9.867741	-1.067161	4.607797
F	8.253719	-0.220516	5.758674
C	-4.524149	7.791349	4.614573
F	-5.854908	7.954323	4.643244
F	-3.963660	9.008019	4.677548
F	-4.184138	7.145248	5.740024
C	-4.287218	7.560689	-5.023179
F	-5.083044	8.619953	-4.830087
F	-4.917759	6.726156	-5.864312
F	-3.190037	7.994213	-5.663638
C	-4.532746	-7.776572	4.629116
F	-3.972708	-8.993299	4.694952
F	-5.863570	-7.939024	4.657743
F	-4.192804	-7.128124	5.753231
C	-4.296281	-7.565550	-5.009145
F	-5.091691	-8.624713	-4.813786
F	-3.199476	-7.999785	-5.649772
F	-4.927684	-6.732507	-5.851116
H	5.877792	-2.509316	-1.304754
H	4.388959	-4.442222	-1.358408
H	4.398230	4.438040	-1.359303
H	5.887053	2.504118	-1.298372
H	-0.769959	6.350647	-1.315808
H	1.648920	6.025482	-1.367512
H	-6.041333	1.589495	-1.382824
H	-5.110785	3.845456	-1.326372
H	-5.115594	-3.841412	-1.319544
H	-6.043364	-1.584432	-1.379960
H	1.641308	-6.028796	-1.358611
H	-0.777900	-6.351411	-1.305532
H	4.702023	-0.061786	-2.641935
H	6.018499	-0.021168	-4.738971
H	9.643612	0.132102	-2.452781
H	8.334787	0.093853	-0.360685
H	6.397773	2.126438	1.466548
H	7.890908	2.099078	3.424179
H	8.178747	-2.169926	3.155619
H	6.680519	-2.151313	1.201187
H	-5.160549	4.635293	1.269802
H	-5.918605	5.936069	3.218411
H	-2.161210	7.998266	3.311579
H	-1.396593	6.702384	1.362505
H	-4.138330	7.244076	-0.378214
H	-4.775705	8.387336	-2.470793
H	-3.017987	5.212247	-4.755714
H	-2.372690	4.064246	-2.658461
H	-1.401964	-6.693204	1.378410
H	-2.167827	-7.984252	3.330193
H	-5.927593	-5.926911	3.225582
H	-5.168241	-4.630873	1.274281
H	-4.144844	-7.240854	-0.364800
H	-4.783475	-8.387724	-2.455023
H	-3.026422	-5.216968	-4.746461
H	-2.379847	-4.065366	-2.651570

No imaginary frequency.

Table S24. Optimized cartesian coordinates of 1a-TS1 (M062X/6-31G(d,p)).

Atom	X	Y	Z
C	-0.602154	-1.260215	1.306624
C	-1.288399	-0.001140	1.440808
C	-0.614790	1.268284	1.337136
C	0.820990	1.239517	1.206206
C	1.577644	0.019091	1.101108
C	0.832844	-1.212922	1.180221
C	2.891703	0.032254	0.558056
C	3.480307	-1.183497	0.045849
C	2.717462	-2.336291	0.099459
C	1.446239	-2.339433	0.702024
C	-1.344478	2.435635	0.994199
C	-0.667414	3.597163	0.465381
C	0.711532	3.523495	0.335058
C	1.425303	2.379253	0.746827
C	-1.318550	-2.427380	0.933674
C	-2.740849	-2.374926	0.685498
C	-3.369190	-1.147792	0.844320
C	-2.642607	-0.006836	1.239492
C	0.750332	-3.482576	0.262791
C	-0.628314	-3.569691	0.380917
C	-3.381662	1.135622	0.874417
C	-2.766675	2.372909	0.746999
C	2.693261	2.397847	0.131997
C	3.473945	1.256830	0.050459
C	4.648346	-1.199788	-0.768381
C	4.829768	-2.318507	-1.571829
C	3.941602	-3.439119	-1.595005
C	2.864008	-3.478535	-0.735186
C	2.807223	3.554753	-0.689455
C	3.848725	3.527504	-1.587140
C	4.745111	2.412659	-1.610100
C	4.625708	1.286481	-0.804658
C	-1.331179	4.654183	-0.211369
C	-0.550018	5.504501	-0.988011
C	0.849947	5.329213	-1.192436
C	1.509364	4.306750	-0.539206
C	-4.613987	0.749539	0.282470
C	-5.319734	1.764059	-0.334432

C	-4.763732	3.076067	-0.386325	F	10.513033	-1.521449	-4.466448
C	-3.498489	3.399194	0.093899	F	11.542582	-1.451503	-2.575880
C	-3.458666	-3.391405	0.001667	F	11.295927	0.355565	-3.743035
C	-4.723288	-3.065427	-0.479477	C	7.679923	0.539831	4.797339
C	-5.294952	-1.762410	-0.390654	F	8.760110	1.335098	4.830414
C	-4.604736	-0.758985	0.260970	F	8.024446	-0.637044	5.338720
C	1.566632	-4.242968	-0.616606	F	6.766083	1.094685	5.608691
C	0.920479	-5.259774	-1.292159	C	-3.863789	7.916268	4.822872
C	-0.481053	-5.449594	-1.104254	F	-4.691478	8.933044	4.532043
C	-1.276820	-4.618584	-0.321984	F	-2.746070	8.456038	5.329285
C	5.561270	0.048036	-0.642457	F	-4.448963	7.201854	5.794043
C	-2.859240	4.800300	-0.025137	C	-5.211865	7.421881	-4.671073
C	-2.803082	-4.783862	-0.151177	F	-5.730184	8.628594	-4.410281
C	6.874882	-0.090437	-1.443483	F	-6.198663	6.661050	-5.170572
C	6.080740	0.143050	0.829605	F	-4.307777	7.570384	-5.651982
C	-3.477199	5.548176	-1.213966	C	-3.933356	-7.884775	4.680803
C	-3.098027	5.573853	1.296277	F	-3.136935	-8.954926	4.808979
C	-3.381668	-5.518177	-1.367922	F	-5.203242	-8.317897	4.698363
C	-3.071294	-5.579833	1.151578	F	-3.765141	-7.132325	5.778611
C	7.501436	0.908596	-2.189874	C	-4.829583	-7.455560	-4.920533
C	8.736610	0.698763	-2.805005	F	-5.658645	-8.474837	-4.660617
C	9.389736	-0.513259	-2.664169	F	-3.802182	-7.948673	-5.630009
C	8.822921	-1.506625	-1.867711	F	-5.476876	-6.598366	-5.724777
C	7.598136	-1.286174	-1.262356	H	5.664152	-2.345609	-2.263333
C	6.631936	1.353358	1.264151	H	4.120293	-4.218839	-2.329639
C	7.164293	1.477079	2.538211	H	3.990867	4.309408	-2.327518
C	7.155396	0.381565	3.399990	H	5.500700	2.459708	-2.376616
C	6.623010	-0.829734	2.980229	H	-1.037139	6.309373	-1.531327
C	6.090213	-0.947709	1.698502	H	1.352443	5.974018	-1.907592
C	-4.154711	5.252385	2.151230	H	-6.262227	1.582626	-0.842918
C	-4.392946	5.996855	3.303087	H	-5.339972	3.842985	-0.895714
C	-3.575686	7.077402	3.611238	H	-5.285364	-3.820766	-1.021662
C	-2.518977	7.413675	2.769717	H	-6.236105	-1.576060	-0.899878
C	-2.284309	6.664306	1.624494	H	1.434284	-5.890022	-2.012203
C	-4.234073	6.708261	-1.074934	H	-0.955116	-6.253066	-1.660592
C	-4.795217	7.331154	-2.192403	H	7.080898	1.894884	-2.279002
C	-4.598314	6.792919	-3.453879	H	9.191959	1.496715	-3.381442
C	-3.843209	5.628107	-3.611176	H	9.351494	-2.440549	-1.705054
C	-3.292519	5.014356	-2.499729	H	7.199927	-2.055282	-0.609864
C	-2.120274	-6.457282	1.676616	H	6.640989	2.208919	0.594536
C	-2.396661	-7.217893	2.809690	H	7.592000	2.419439	2.865109
C	-3.635046	-7.111072	3.429015	H	6.625405	-1.683577	3.649245
C	-4.598140	-6.245734	2.916091	H	5.677009	-1.901365	1.386551
C	-4.314403	-5.486703	1.788923	H	-4.799604	4.410449	1.926046
C	-3.984513	-6.770698	-1.287410	H	-5.209651	5.729200	3.965336
C	-4.471033	-7.401814	-2.434357	H	-1.875194	8.251450	3.016822
C	-4.355611	-6.778736	-3.667041	H	-1.452590	6.930726	0.980999
C	-3.753125	-5.522539	-3.765678	H	-4.396998	7.140400	-0.093827
C	-3.270960	-4.903516	-2.625296	H	-5.381484	8.235774	-2.074361
C	10.691038	-0.777872	-3.363011	H	-3.690423	5.212439	-4.602558

H	-2.706687	4.106756	-2.617312
H	-1.150274	-6.555043	1.202367
H	-1.648900	-7.896251	3.206277
H	-5.568142	-6.169103	3.397037
H	-5.071996	-4.814525	1.400442
H	-4.081652	-7.270038	-0.329764
H	-4.942378	-8.375856	-2.361312
H	-3.669841	-5.036976	-4.733060
H	-2.800243	-3.926818	-2.697868

Only one imaginary frequency (-40.61).

Table S25. Optimized cartesian coordinates of 1a-IM (M062X/6-31G(d,p)).

Atom	X	Y	Z
C	0.597260	1.264582	1.285190
C	1.273804	0.000057	1.424783
C	0.597500	-1.264589	1.285118
C	-0.834835	-1.227431	1.121392
C	-1.585012	-0.000203	1.018236
C	-0.835068	1.227163	1.121476
C	-2.888565	-0.000308	0.450042
C	-3.457933	1.219963	-0.081584
C	-2.682205	2.362933	-0.014638
C	-1.429174	2.358819	0.628105
C	1.331333	-2.430314	0.942450
C	0.665060	-3.578393	0.372295
C	-0.708951	-3.495474	0.205887
C	-1.428721	-2.359165	0.627936
C	1.330867	2.430467	0.942580
C	2.760091	2.372712	0.738261
C	3.378014	1.141736	0.905646
C	2.633640	0.000192	1.263854
C	-0.709629	3.495297	0.206132
C	0.664369	3.578459	0.372518
C	3.378236	-1.141192	0.905602
C	2.760549	-2.372278	0.738157
C	-2.681738	-2.363466	-0.014836
C	-3.457682	-1.220641	-0.081708
C	-4.588628	1.233359	-0.955634
C	-4.691611	2.319802	-1.813867
C	-3.793686	3.433929	-1.805992
C	-2.779443	3.492693	-0.875574
C	-2.778731	-3.493171	-0.875877
C	-3.792931	-3.434502	-1.806344
C	-4.691071	-2.320543	-1.814155
C	-4.588352	-1.234175	-0.955799
C	1.342283	-4.625052	-0.307594
C	0.579406	-5.452649	-1.125538
C	-0.813130	-5.263574	-1.367369
C	-1.486287	-4.253292	-0.709577
C	4.630674	-0.753640	0.358494
C	5.349245	-1.761328	-0.254512
C	4.786914	-3.068014	-0.347680
C	3.505750	-3.391143	0.088165
C	3.505090	3.391744	0.088298
C	4.786317	3.068878	-0.347559
C	5.348904	1.762300	-0.254438
C	4.630528	0.754447	0.358525
C	-1.487131	4.253038	-0.709258
C	-0.814173	5.263479	-1.367008
C	0.578340	5.452785	-1.125207
C	1.341383	4.625289	-0.307322
C	-5.517674	-0.000518	-0.806181
C	2.862566	-4.785529	-0.078372
C	2.861650	4.786020	-0.078172
C	-6.832675	-0.000566	-1.607291
C	-6.042138	-0.000719	0.665373
C	3.509590	-5.515109	-1.263209
C	3.058822	-5.585581	1.234186
C	3.508425	5.515750	-1.263054
C	3.057883	5.586074	1.234391
C	-7.519873	-1.194271	-1.870223
C	-8.764564	-1.200345	-2.488307
C	-9.377680	-0.000629	-2.827556
C	-8.764855	1.199106	-2.487824
C	-7.520167	1.193083	-1.869748
C	-6.328737	-1.202708	1.316261
C	-6.879373	-1.206351	2.592147
C	-7.153072	-0.001147	3.230593
C	-6.879395	1.204291	2.592498
C	-6.328753	1.201093	1.316646
C	4.111249	-5.304107	2.108376
C	4.317851	-6.074269	3.249301
C	3.471982	-7.140910	3.527291
C	2.420996	-7.439453	2.664529
C	2.218403	-6.664976	1.529851
C	4.249664	-6.687132	-1.125176
C	4.835460	-7.293631	-2.237690
C	4.683258	-6.727469	-3.494479
C	3.949652	-5.549887	-3.649673
C	3.372073	-4.952714	-2.541547
C	2.217275	6.665270	1.530227
C	2.419877	7.439756	2.664904
C	3.471059	7.141416	3.527488
C	4.317118	6.074963	3.249330
C	4.110509	5.304800	2.108416
C	4.248264	6.687936	-1.125085
C	4.833744	7.294607	-2.237663

C	4.681463	6.728469	-3.494462
C	3.948088	5.550743	-3.649593
C	3.370811	4.953399	-2.541393
C	10.690318	-0.000643	-3.555195
F	10.515652	-0.000333	-4.886164
F	11.422516	1.081968	-3.257100
F	11.422233	-1.083573	-3.257566
C	-7.695209	-0.001038	4.630320
F	-8.446873	-1.085480	4.870465
F	-8.452804	1.079909	4.867826
F	-6.712257	0.002873	5.544096
C	3.654569	-7.941698	4.784355
F	4.931420	-7.939719	5.193231
F	3.283909	-9.219416	4.612985
F	2.917996	-7.453679	5.793714
C	5.260714	-7.400242	-4.706293
F	6.297403	-8.189538	-4.393807
F	5.687327	-6.504873	-5.609502
F	4.350486	-8.170204	-5.323202
C	3.653703	7.942184	4.784557
F	3.282439	9.219755	4.613417
F	4.930692	7.940718	5.193011
F	2.917682	7.453759	5.794123
C	5.258564	7.401536	-4.706285
F	6.295855	8.190125	-4.393987
F	4.348397	8.172325	-5.322253
F	5.684179	6.506412	-5.610201
H	-5.452433	2.323329	-2.584076
H	-3.910514	4.190512	-2.576475
H	-3.909573	-4.191028	-2.576911
H	-5.451836	-2.324138	-2.584417
H	1.077304	-6.248973	-1.671580
H	-1.297762	-5.888726	-2.111696
H	6.307652	-1.576410	-0.731008
H	5.372879	-3.829328	-0.854407
H	5.372131	3.830324	-0.854262
H	6.307347	1.577587	-0.730944
H	-1.298933	5.888588	-2.111286
H	1.076082	6.249222	-1.671229
H	-7.111350	-2.141849	-1.546134
H	-9.269248	-2.140977	-2.682293
H	-9.269775	2.139690	-2.681431
H	-7.111851	2.140626	-1.545286
H	-6.114506	-2.148580	0.827617
H	-7.100387	-2.144988	3.089390
H	-7.100481	2.142768	3.090047
H	-6.114530	2.147121	0.828299
H	4.781983	-4.477315	1.903293
H	5.140865	-5.846682	3.918594
H	1.766047	-8.277767	2.878558
H	1.395590	-6.907012	0.865615
H	4.381521	-7.138430	-0.148034
H	5.415033	-8.203001	-2.119615
H	3.843216	-5.104170	-4.633451
H	2.805944	-4.032435	-2.657309
H	1.394306	6.907154	0.866129
H	1.764776	8.277918	2.879060
H	5.140288	5.847537	3.918489
H	4.781391	4.478160	1.903200
H	4.380181	7.139216	-0.147943
H	5.413151	8.204093	-2.119641
H	3.841584	5.105046	-4.633369
H	2.804853	4.033008	-2.657112

No imaginary frequency.

Table S26. Optimized cartesian coordinates of 1a-TS2 (M062X/6-31G(d,p)).

Atom	X	Y	Z
C	-0.585368	-1.382600	1.397044
C	-1.305616	-0.134889	1.406204
C	-0.664278	1.136573	1.189771
C	0.772589	1.133731	1.076359
C	1.562143	-0.072642	1.087537
C	0.849616	-1.309803	1.273925
C	2.880249	-0.075411	0.554141
C	3.511233	-1.310091	0.137576
C	2.772655	-2.470913	0.296340
C	1.499571	-2.455786	0.900528
C	-1.419080	2.248017	0.732126
C	-0.766490	3.375422	0.107351
C	0.615447	3.330458	0.003303
C	1.348598	2.246437	0.524836
C	-1.268738	-2.597425	1.132586
C	-2.689598	-2.605444	0.870051
C	-3.350325	-1.385672	0.903964
C	-2.657041	-0.194081	1.194418
C	0.832112	-3.652283	0.567373
C	-0.544339	-3.767503	0.692963
C	-3.420333	0.888792	0.714869
C	-2.835626	2.124417	0.476710
C	2.624037	2.247640	-0.069024
C	3.428352	1.122446	-0.038442
C	4.670713	-1.357567	-0.706370
C	4.839403	-2.534186	-1.426537
C	3.984936	-3.677584	-1.328439
C	2.936757	-3.679032	-0.438198
C	2.735859	3.333481	-0.980691
C	3.820908	3.273829	-1.829802

C	4.749071	2.191040	-1.723475	C	-4.364893	-6.188685	3.554400
C	4.601804	1.124977	-0.845032	C	-4.110588	-5.529472	2.356931
C	-1.447034	4.348369	-0.670896	C	-3.886877	-7.164667	-0.654137
C	-0.674705	5.147900	-1.508140	C	-4.378676	-7.906180	-1.730816
C	0.734038	4.997021	-1.673917	C	-4.233238	-7.426984	-3.023064
C	1.411084	4.056277	-0.922944	C	-3.597105	-6.204708	-3.251900
C	-4.635042	0.415867	0.150236	C	-3.111071	-5.475372	-2.180562
C	-5.356768	1.347514	-0.570356	C	10.678285	0.743121	-3.371035
C	-4.833183	2.662697	-0.742169	F	10.476403	1.258662	-4.594017
C	-3.584214	3.064622	-0.279108	F	11.387035	-0.382968	-3.532064
C	-3.375589	-3.701067	0.282978	F	11.444478	1.616605	-2.702183
C	-4.643733	-3.457249	-0.235523	C	7.754168	-0.087379	4.803018
C	-5.247919	-2.166552	-0.279773	F	7.273953	0.889489	5.584610
C	-4.588722	-1.087070	0.275114	F	9.085962	0.073489	4.742619
C	1.666280	-4.466548	-0.242265	F	7.535092	-1.254570	5.427093
C	1.051509	-5.558799	-0.822099	C	-4.107688	7.924705	4.063707
C	-0.343021	-5.771213	-0.614620	F	-5.409079	7.932412	4.386699
C	-1.162125	-4.894671	0.090135	F	-3.737261	9.193393	3.836025
C	5.558757	-0.076507	-0.627488	F	-3.436852	7.510086	5.148814
C	-2.981305	4.466510	-0.520386	C	-5.255872	6.648103	-5.426426
C	-2.685029	-5.083477	0.272948	F	-6.338634	7.409530	-5.218417
C	6.872563	0.055950	-1.429786	F	-5.600102	5.674693	-6.282997
C	6.071743	-0.049583	0.849813	F	-4.349337	7.414155	-6.053501
C	-3.598121	5.084053	-1.782351	C	-3.677766	-7.759689	5.381163
C	-3.265597	5.350532	0.720660	F	-3.099795	-8.968569	5.437448
C	-3.248359	-5.945488	-0.864612	F	-4.987310	-7.923190	5.619306
C	-2.928306	-5.758711	1.646784	F	-3.181270	-7.040752	6.398990
C	7.550279	1.286295	-1.324767	C	-4.716752	-8.222912	-4.200585
C	8.771909	1.511513	-1.936082	F	-5.560189	-9.198205	-3.838355
C	9.381727	0.488893	-2.659240	F	-3.696531	-8.800368	-4.854588
C	8.773184	-0.753215	-2.723607	F	-5.352337	-7.443984	-5.089349
C	7.540784	-0.966741	-2.105636	H	5.603528	-2.608818	-2.182192
C	6.031187	1.096610	1.642565	H	4.163335	-4.505218	-2.008838
C	6.559947	1.087841	2.931861	H	3.977023	4.006462	-2.616243
C	7.134871	-0.070220	3.435992	H	5.587788	2.200823	-2.410159
C	7.189355	-1.221908	2.652313	H	-1.173289	5.887063	-2.128890
C	6.663778	-1.206103	1.369858	H	1.230422	5.591507	-2.435430
C	-4.349019	5.094063	1.564603	H	-6.286986	1.093139	-1.070134
C	-4.634028	5.934568	2.636640	H	-5.421160	3.361567	-1.330074
C	-3.836509	7.047171	2.875748	H	-5.183300	-4.277396	-0.700562
C	-2.755946	7.321361	2.042304	H	-6.188705	-2.055107	-0.811114
C	-2.475408	6.476907	0.975844	H	1.584430	-6.235719	-1.483514
C	-4.390045	6.229846	-1.761806	H	-0.794322	-6.633838	-1.096508
C	-4.945656	6.731650	-2.939891	H	7.119038	2.081948	-0.727358
C	-4.711123	6.086436	-4.144893	H	9.264731	2.472650	-1.832306
C	-3.924933	4.933519	-4.182363	H	9.263370	-1.571194	-3.240798
C	-3.377792	4.440165	-3.009574	H	7.156404	-1.971167	-2.133175
C	-2.006369	-6.671911	2.167705	H	5.578047	2.008115	1.266765
C	-2.254327	-7.334207	3.364601	H	6.513499	1.981246	3.545124
C	-3.434275	-7.090268	4.059261	H	7.634344	-2.127794	3.051801

H	6.703883	-2.106644	0.763425
H	-4.982422	4.231777	1.389193
H	-5.480392	5.725776	3.282677
H	-2.138616	8.194751	2.225283
H	-1.629972	6.700837	0.334104
H	-4.586394	6.741717	-0.826238
H	-5.565941	7.621209	-2.913284
H	-3.754287	4.425571	-5.126094
H	-2.770945	3.539057	-3.033622
H	-1.082740	-6.872732	1.636043
H	-1.532463	-8.043633	3.755724
H	-5.288406	-6.004619	4.093378
H	-4.845309	-4.829359	1.974786
H	-4.010164	-7.550696	0.351662
H	-4.877748	-8.853232	-1.556881
H	-3.492123	-5.831007	-4.265760
H	-2.617399	-4.522989	-2.354021

Only one imaginary frequency (-41.11).

Table S27. Optimized cartesian coordinates of 1b (M062X/6-31G(d,p)).

Atom	X	Y	Z
C	1.288719	0.682021	-1.370410
C	1.198099	-0.751908	-1.409654
C	-0.053899	-1.456805	-1.370695
C	-1.250414	-0.661369	-1.409649
C	-1.234863	0.775337	-1.370379
C	0.052263	1.413844	-1.409276
C	-2.376796	1.492099	-0.937612
C	-2.269767	2.876577	-0.539243
C	-1.015762	3.471631	-0.636393
C	0.101246	2.738201	-1.080680
C	-0.103726	-2.804199	-0.938173
C	-1.356293	-3.403855	-0.540176
C	-2.498628	-2.615383	-0.637430
C	-2.421902	-1.281204	-1.081429
C	2.480499	1.312460	-0.937676
C	3.626094	0.527459	-0.539811
C	3.514395	-0.856051	-0.637326
C	2.320612	-1.456593	-1.081476
C	1.269402	3.387116	-0.636446
C	2.476038	2.701067	-0.539334
C	2.298589	-2.792799	-0.637506
C	1.101129	-3.494734	-0.540200
C	-3.568041	-0.594124	-0.637204
C	-3.577229	0.793855	-0.539701
C	-3.275492	3.563734	0.187247
C	-2.908088	4.795670	0.732705
C	-1.586144	5.326128	0.706159
C	-0.584950	4.640975	0.040870
C	-4.433996	-1.490533	0.039725
C	-5.486336	-0.886842	0.704973
C	-5.583226	0.534249	0.731845
C	-4.633356	1.400744	0.186774
C	-1.448634	-4.618522	0.186154
C	-2.699339	-4.916443	0.731276
C	-3.819710	-4.036870	0.704625
C	-3.726832	-2.827113	0.039578
C	3.507843	-3.094694	0.039402
C	3.511141	-4.308027	0.704424
C	2.328844	-5.102427	0.731126
C	1.103538	-4.712913	0.186080
C	4.724208	1.054673	0.186603
C	5.607533	0.120357	0.731561
C	5.405937	-1.289702	0.704654
C	4.311816	-1.813995	0.039502
C	0.926168	4.585091	0.040816
C	1.975260	5.194442	0.706035
C	3.254416	4.567805	0.732549
C	3.529809	3.312080	0.187111
C	-4.672359	2.932903	0.367081
C	-0.203869	-5.512747	0.366327
C	4.876250	2.579779	0.366961
C	-5.255702	3.298728	1.751450
C	-5.624176	3.530628	-0.712068
C	-0.229287	-6.200465	1.750882
C	-0.245506	-6.636126	-0.712580
C	5.484920	2.901496	1.751370
C	5.869638	3.105665	-0.712126
C	-4.448484	2.791952	2.943784
C	-5.221693	3.278047	-2.161655
C	-0.193824	-5.247630	2.942912
C	5.449704	2.883417	-2.161733
C	4.642395	2.455738	2.943649
C	-0.228140	-6.161469	-2.162231
H	-3.642455	5.362727	1.296773
H	-1.384346	6.230176	1.274068
H	-6.212859	-1.461655	1.272692
H	-6.413208	0.948990	1.295879
H	-2.823362	-5.836031	1.295181
H	-4.703621	-4.314233	1.272359
H	4.372203	-4.649963	1.272047
H	2.384582	-6.028687	1.295005
H	6.465960	0.472625	1.295495
H	6.088087	-1.916630	1.272266
H	1.840812	6.110927	1.273951
H	4.028674	5.079132	1.296541
H	-6.281608	2.918269	1.805980
H	-5.359518	4.387958	1.806268

H	-6.624568	3.116374	-0.530410
H	-5.685786	4.611621	-0.530288
H	0.612836	-6.899057	1.805695
H	-1.120957	-6.834586	1.805807
H	-1.150811	-7.230018	-0.530641
H	0.613497	-7.295274	-0.530861
H	6.479955	2.446382	1.805974
H	5.668832	3.980096	1.806199
H	6.010751	4.179176	-0.530343
H	6.836768	2.618812	-0.530419
H	-4.922380	3.089105	3.883769
H	-4.372289	1.700809	2.932456
H	-3.432825	3.197954	2.932814
H	-5.948859	3.734595	-2.839029
H	-4.240445	3.707625	-2.384226
H	-5.182094	2.207633	-2.384306
H	-0.214560	-5.806280	3.883087
H	0.713247	-4.636382	2.931513
H	-1.053028	-4.570746	2.931573
H	6.208371	3.285483	-2.839087
H	5.331741	1.818808	-2.384490
H	4.502576	3.383795	-2.384218
H	5.136884	2.717096	3.883661
H	3.659506	2.935665	2.932602
H	4.485808	1.373207	2.932355
H	-0.260057	-7.019558	-2.839508
H	-1.090783	-5.526434	-2.384758
H	0.679078	-5.592055	-2.385057

No imaginary frequency.

Table S28. Optimized cartesian coordinates of 1-H (M062X/6-31G(d,p)).

Atom	X	Y	Z
C	1.264857	0.730266	1.366529
C	0.000000	1.418453	1.400815
C	-1.264857	0.730266	1.366529
C	-1.228396	-0.709213	1.400909
C	0.000000	-1.460551	1.366461
C	1.228396	-0.709213	1.400909
C	0.000000	-2.812121	0.938235
C	1.230145	-3.451707	0.524722
C	2.397137	-2.702322	0.594352
C	2.375174	-1.371287	1.055311
C	-2.435410	1.406071	0.938414
C	-3.604455	0.660553	0.525068
C	-3.538929	-0.724777	0.594525
C	-2.375174	-1.371287	1.055311
C	2.435410	1.406071	0.938414
C	2.374246	2.791170	0.524757
C	1.141736	3.427051	0.594017
C	0.000000	2.742557	1.054987
C	3.538929	-0.724777	0.594525
C	3.604455	0.660553	0.525068
C	-1.141736	3.427051	0.594017
C	-2.374246	2.791170	0.524757
C	-2.397137	-2.702322	0.594352
C	-1.230145	-3.451707	0.524722
C	1.271212	-4.654743	-0.223985
C	2.470420	-4.976306	-0.851049
C	3.618226	-4.132407	-0.845514
C	3.591906	-2.945821	-0.133853
C	-3.591906	-2.945821	-0.133853
C	-3.618226	-4.132407	-0.845514
C	-2.470420	-4.976306	-0.851049
C	-1.271212	-4.654743	-0.223985
C	-4.666989	1.226580	-0.223440
C	-5.545091	0.348863	-0.850534
C	-5.388062	-1.067114	-0.845216
C	-4.347216	-1.637657	-0.133722
C	-0.755290	4.583376	-0.134398
C	-1.769841	5.199399	-0.845954
C	-3.074647	4.627430	-0.851117
C	-3.395733	3.428242	-0.223782
C	3.395733	3.428242	-0.223782
C	3.074647	4.627430	-0.851117
C	1.769841	5.199399	-0.845954
C	0.755290	4.583376	-0.134398
C	4.347216	-1.637657	-0.133722
C	5.388062	-1.067114	-0.845216
C	5.545091	0.348863	-0.850534
C	4.666989	1.226580	-0.223440
C	0.000000	-5.483452	-0.347821
C	-4.749111	2.741829	-0.347063
C	4.749111	2.741829	-0.347063
H	2.515013	-5.883489	-1.448939
H	4.466918	-4.409718	-1.464611
H	-4.466918	-4.409718	-1.464611
H	-2.515013	-5.883489	-1.448939
H	-6.353108	0.763824	-1.448329
H	-6.052566	-1.663375	-1.464386
H	-1.585746	6.072933	-1.465236
H	-3.838053	5.119660	-1.448919
H	3.838053	5.119660	-1.448919
H	1.585746	6.072933	-1.465236
H	6.052566	-1.663375	-1.464386
H	6.353108	0.763824	-1.448329
H	0.000000	-6.237390	0.454022
H	0.000000	-6.041037	-1.290123
H	-5.401574	3.118702	0.455210

H	-5.232468	3.020757	-1.289081
H	5.401574	3.118702	0.455210
H	5.232468	3.020757	-1.289081

No imaginary frequency.

Table S29. Optimized cartesian coordinates of 1-H TS (M062X/6-31G(d,p)).

Atom	X	Y	Z
C	1.233194	0.712007	-0.178724
C	-0.000001	1.369855	-0.179708
C	-1.233194	0.712005	-0.179951
C	-1.186298	-0.684904	-0.179427
C	0.000001	-1.423945	-0.178473
C	1.186299	-0.684902	-0.178015
C	0.000002	-2.807844	-0.178118
C	1.256930	-3.499741	-0.177538
C	2.429671	-2.734171	-0.177183
C	2.343514	-1.353027	-0.177255
C	-2.431637	1.403936	-0.180487
C	-3.659258	0.661346	-0.180480
C	-3.582746	-0.737101	-0.180184
C	-2.343512	-1.353030	-0.179693
C	2.431636	1.403939	-0.178531
C	2.402439	2.838362	-0.179109
C	1.153047	3.471240	-0.179755
C	-0.000002	2.706078	-0.180182
C	3.582749	-0.737096	-0.177365
C	3.659257	0.661353	-0.177884
C	-1.153051	3.471240	-0.180592
C	-2.402443	2.838359	-0.180689
C	-2.429666	-2.734172	-0.179167
C	-1.256926	-3.499742	-0.178555
C	1.347486	-4.896825	-0.177716
C	2.679637	-5.357042	-0.177717
C	3.867487	-4.521345	-0.177590
C	3.788750	-3.120012	-0.177300
C	-3.788744	-3.120014	-0.179358
C	-3.867482	-4.521348	-0.179015
C	-2.679632	-5.357045	-0.178583
C	-1.347480	-4.896826	-0.178342
C	-4.914442	1.281420	-0.180520
C	-5.979137	0.357866	-0.180282
C	-5.849400	-1.088685	-0.179984
C	-4.596415	-1.721192	-0.179906
C	-0.807631	4.841122	-0.180359
C	-1.981815	5.610007	-0.180330
C	-3.299470	4.999185	-0.180501
C	-3.567075	3.615362	-0.180664
C	3.567070	3.615367	-0.178891
C	3.299464	4.999189	-0.179125
C	1.981807	5.610009	-0.179544
C	0.807625	4.841122	-0.179911
C	4.596418	-1.721185	-0.177401
C	5.849403	-1.088675	-0.177775
C	5.979137	0.357875	-0.178041
C	4.914441	1.281428	-0.178122
C	0.000003	-5.710189	-0.177951
C	-4.945214	2.855106	-0.180714
C	4.945210	2.855114	-0.178485
H	2.867291	-6.428443	-0.177928
H	4.818244	-5.047674	-0.177799
H	-4.818240	-5.047675	-0.179091
H	-2.867284	-6.428446	-0.178428
H	-7.000786	0.731174	-0.180257
H	-6.780628	-1.648845	-0.179754
H	-1.962342	6.696573	-0.180100
H	-4.133494	5.697423	-0.180427
H	4.133487	5.697428	-0.178949
H	1.962332	6.696575	-0.179567
H	6.780632	-1.648834	-0.177929
H	7.000785	0.731185	-0.178280
H	-0.000185	-6.374786	0.694505
H	0.000189	-6.374979	-1.050257
H	-5.520928	3.187483	0.691580
H	-5.520707	3.187253	-1.053244
H	5.520678	3.187567	0.693944
H	5.520948	3.187187	-1.050880

Only one imaginary frequency (-84.60).

Table S30. Optimized cartesian coordinates of *c*-HBC (M062X/6-31G(d,p)).

Atom	X	Y	Z
C	1.106073	0.923082	0.000111
C	-0.246377	1.419394	0.000017
C	-1.352435	0.496322	-0.000062
C	-1.106073	-0.923082	-0.000111
C	0.246377	-1.419394	-0.000017
C	1.352435	-0.496322	0.000062
C	0.484832	-2.793037	0.000012
C	1.795383	-3.259339	0.403976
C	2.885646	-2.349507	0.403939
C	2.661279	-0.976657	0.000061
C	-2.661279	0.976657	-0.000061
C	-3.720415	0.074825	0.403781
C	-3.477571	-1.324295	0.403681
C	-2.176459	-1.816403	-0.000207

C	2.176459	1.816403	0.000207
C	1.924972	3.184561	0.404095
C	0.591866	3.673779	0.403967
C	-0.484832	2.793037	-0.000012
C	-1.795383	3.259339	-0.403976
C	-2.885646	2.349507	-0.403939
C	3.720415	-0.074825	-0.403781
C	3.477571	1.324295	-0.403681
C	-1.924972	-3.184561	-0.404095
C	-0.591866	-3.673779	-0.403967
C	1.992160	-4.552724	0.943446
C	3.217046	-4.949792	1.431577
C	4.294440	-4.050742	1.431480
C	4.122986	-2.774581	0.943295
C	-2.946608	-4.001770	-0.943559
C	-2.677904	-5.261085	-1.431664
C	-1.360565	-5.744499	-1.431547
C	-0.341194	-4.957861	-0.943317
C	-4.938938	0.551047	0.943238
C	-5.895216	-0.311259	1.431359
C	-5.655256	-1.693826	1.431233
C	-4.464342	-2.183370	0.943011
C	-1.992160	4.552724	-0.943446
C	-3.217046	4.949792	-1.431577
C	-4.294440	4.050742	-1.431480
C	-4.122986	2.774581	-0.943295
C	2.946608	4.001770	0.943559
C	2.677904	5.261085	1.431664
C	1.360565	5.744499	1.431547
C	0.341194	4.957861	0.943317
C	4.938938	-0.551047	-0.943238
C	5.895216	0.311259	-1.431359
C	5.655256	1.693826	-1.431233
C	4.464342	2.183370	-0.943011
H	1.143541	-5.220362	1.034894
H	3.332605	-5.940449	1.859766
H	5.248457	-4.341749	1.859576
H	4.931726	-2.059119	1.034568
H	-3.949141	-3.600750	-1.035045
H	-3.477985	-5.856569	-1.859878
H	-1.135496	-6.716172	-1.859676
H	0.682801	-5.300482	-1.034554
H	-5.092835	1.619791	1.034711
H	-6.810917	0.083935	1.859627
H	-6.384229	-2.374578	1.859344
H	-4.249122	-3.241486	1.034309
H	-1.143541	5.220362	-1.034894
H	-3.332605	5.940449	-1.859766
H	-5.248457	4.341749	-1.859576
H	-4.931726	2.059119	-1.034568
H	3.949141	3.600750	1.035045
H	3.477985	5.856569	1.859878
H	1.135496	6.716172	1.859676
H	-0.682801	5.300482	1.034554
H	5.092835	-1.619791	-1.034711
H	6.810917	-0.083935	-1.859627
H	6.384229	2.374578	-1.859344
H	4.249122	3.241486	-1.034309

No imaginary frequency.

Table S31. Cartesian coordinates of 1a@C_{60} (B3LYP-D3(BJ)/6-31+G(d,p)).

Atom	X	Y	Z
F	-10.344473	-0.103220	-2.889409
C	-1.461126	-0.178445	3.035471
C	0.552763	1.354176	3.041428
C	-0.559946	-1.301382	3.071781
C	-0.870618	1.135200	3.064491
C	-2.812012	-0.352643	2.598710
C	3.529866	-0.698073	2.248982
C	1.081356	2.611189	2.609548
C	-3.290587	-1.649412	2.176270
C	1.394916	0.186015	3.078157
C	-3.593146	0.781004	2.163557
C	-1.668865	2.188403	2.682085
C	0.873027	-1.156143	3.049100
C	-2.985996	2.034200	2.208301
C	-1.168138	3.419496	2.215580
C	-3.370381	3.181470	1.454542
C	3.065975	-2.011584	2.207732
C	2.709780	0.350924	2.708372
C	-1.071036	-2.522229	2.696140
C	0.201602	3.674247	2.179319
C	-4.496281	-1.847626	1.445575
C	-2.385291	-2.707863	2.224749
C	2.457838	2.721164	2.187707
C	1.701428	-2.242202	2.624682
C	4.624890	-0.169258	1.505523
C	-2.169859	4.097201	1.461430
C	-4.550338	3.045567	0.737405
C	3.239354	1.568852	2.240034
C	-0.276600	-3.589324	2.234393
C	-4.808353	0.664677	1.432067
C	1.112494	-3.488913	2.196161
C	-5.263822	1.806960	0.770759
C	0.639223	4.818089	1.453468
C	-6.310717	-0.829558	2.814004
C	3.844470	-2.962212	1.488847
C	2.598014	6.049010	0.304295

C	-4.652187	-3.079350	0.804494	C	-6.792604	-2.082669	3.222781
F	-8.304265	-0.300638	7.364908	C	3.722813	6.250034	4.851100
C	-1.074118	-4.501600	1.483595	C	-8.779978	-0.574508	-0.642601
C	5.373115	-1.109794	0.812316	C	4.893761	-7.320736	-0.566803
C	-5.548604	-0.700914	1.460674	C	-8.593560	-1.254209	6.451828
C	-0.362814	-5.461599	0.778159	C	4.801772	-6.805312	-1.858422
C	2.414907	5.883398	2.834208	C	3.917705	7.873672	-0.615709
C	4.432198	1.328286	1.497548	C	3.078890	8.002700	6.534192
C	-1.726103	5.210262	0.762041	C	3.033766	-5.810158	3.686173
C	-6.539752	-0.778413	0.283289	C	-7.514382	-2.225144	4.402640
F	-9.917608	-1.152880	6.182000	C	5.189719	-4.823000	3.281800
C	1.824481	-4.488949	1.476354	C	5.313746	-7.593195	-3.031985
F	2.695516	9.298244	6.463953	C	-9.207785	-0.800347	-3.111119
C	-2.467473	-3.919657	1.478790	C	4.824519	-6.133541	5.275066
C	2.158463	5.155596	1.480228	C	3.863804	-5.031712	2.871854
C	4.195413	3.655443	0.812334	C	3.506612	-6.354713	4.880716
C	2.971598	3.831766	1.461229	C	3.952980	8.357803	-3.083043
C	-7.765688	-1.109233	5.205148	C	5.669320	-5.366914	4.468099
C	4.911654	2.418390	0.785610	C	5.323450	-6.664744	6.590214
C	1.066349	-5.460113	0.819218	F	6.640488	-6.969934	6.539370
C	-0.344280	5.570366	0.805639	F	4.659998	-7.780558	6.968217
C	3.945020	-5.273671	0.342392	F	5.169473	-5.758329	7.584083
C	-3.649036	-4.096270	0.773390	F	4.074953	7.608117	-4.202429
C	4.993548	-2.486571	0.851560	F	3.060159	9.337127	-3.363056
F	6.598462	-7.276269	-3.321293	F	5.142798	8.960821	-2.863203
C	2.209813	5.717602	-1.002899	H	-4.926602	3.838922	0.097085
F	-8.406057	-2.456699	7.041430	H	-6.179905	1.744509	0.193030
F	4.590704	-7.351489	-4.149584	H	-5.554726	-3.257210	0.228859
C	-6.054072	-0.949505	-1.022389	H	6.214976	-0.825538	0.186644
F	2.356429	7.433780	7.527907	H	-0.858464	-6.189204	0.141042
C	3.377200	-4.446708	1.511903	H	-2.390002	5.792401	0.128527
C	3.855448	-4.775649	-0.966751	H	4.605057	4.480029	0.238697
F	4.372965	7.980390	6.925292	H	5.792979	2.347675	0.153734
C	-7.915723	-0.586135	0.453838	H	1.581464	-6.226873	0.250343
C	3.462768	7.136023	0.478671	H	-0.042290	6.441666	0.233611
C	1.574469	6.937615	3.224013	H	-3.817433	-4.966331	0.144410
C	-6.906597	-0.941118	-2.119550	H	5.602124	-3.183668	0.284749
C	4.466662	-6.559921	0.523169	H	1.547171	4.879555	-1.159870
C	2.655664	6.447267	-2.098278	H	-4.995157	-1.094348	-1.176668
F	5.278297	-8.924825	-2.802237	H	3.451408	-3.787442	-1.128829
C	-6.576802	0.278949	3.625533	H	-8.325053	-0.436350	1.445900
C	2.875701	7.290942	5.225432	H	3.795386	7.411950	1.472408
F	-9.574351	-2.071000	-3.403895	H	0.734248	7.215895	2.597379
C	3.494200	5.555957	3.662335	H	-6.503994	-1.067511	-3.119297
C	-8.278164	-0.752196	-1.930605	H	4.537848	-6.982860	1.518286
C	-7.296432	0.142745	4.813219	H	2.346623	6.164722	-3.099314
C	4.279581	-5.524591	-2.057499	H	-6.222119	1.261041	3.336698
C	1.799264	7.637119	4.404348	H	4.169530	4.756081	3.383307
F	-8.628317	-0.294766	-4.224184	H	-7.484977	1.010272	5.436893
C	3.515226	7.531767	-1.905681	H	4.191646	-5.118916	-3.060100

H	1.144483	8.454535	4.688218
H	-6.597406	-2.959037	2.614609
H	4.565061	5.985265	5.481663
H	-9.843236	-0.418080	-0.494179
H	5.289940	-8.318572	-0.410633
H	4.593043	8.709233	-0.464451
H	2.008783	-6.001267	3.391786
H	-7.873172	-3.202430	4.708782
H	5.856126	-4.228663	2.666389
H	2.850933	-6.957532	5.500240
H	6.699204	-5.202140	4.767553
C	-1.516435	-2.784989	-1.980933
C	-2.591616	-1.808105	-1.998052
C	-0.324512	-1.387769	-0.331869
C	-1.341800	-3.282499	-3.335837
C	1.104166	-0.988126	-6.817786
C	2.427431	0.936808	-1.149683
C	-3.089088	-1.705661	-3.360009
C	-2.512666	-0.667533	-1.198718
C	-1.966219	-2.248841	-5.488245
C	3.138336	1.685598	-3.805396
C	3.044041	-1.854653	-3.295447
C	2.866961	-1.353009	-1.942747
C	-0.632792	-2.533382	-5.991954
C	0.300520	-3.172618	-5.174723
C	-2.314207	-2.616313	-4.187906
C	2.177202	-2.827657	-3.798532
C	2.012544	2.224317	-1.678164
C	3.536113	0.447079	-3.298356
C	1.057000	-0.937965	-0.319611
C	2.979921	-0.641593	-5.442344
C	1.348987	0.423956	-0.321059
C	-1.056654	0.967610	-0.347936
C	3.175419	0.066658	-1.942322
C	-1.357772	-0.453899	-0.346510
C	-0.403978	-2.575217	-1.166412
C	-0.061004	-3.555444	-3.819838
C	1.830009	-1.843636	-1.149071
C	-0.222637	-1.418955	-6.829895
C	0.268731	1.395611	-0.336177
C	0.928262	-2.857598	-1.671122
C	1.683520	-2.723986	-5.162347
C	2.076266	-1.652992	-5.966074
C	3.455717	-0.740314	-4.134397
C	1.563153	2.760986	-5.183444
C	2.566219	0.647988	-5.970030
C	2.643262	1.787717	-5.168331
C	-1.302732	-0.446152	-6.844907
C	1.098032	-3.340453	-2.968979
C	-2.380421	-0.958675	-6.015215
C	1.406544	0.433774	-6.819521
C	2.362256	2.594043	-2.977207
C	-2.029415	1.633051	-1.198661
C	0.449426	2.553976	-5.998340
C	0.370125	1.367380	-6.833873
C	-3.488775	-0.466635	-3.866264
C	0.677116	2.505717	-1.176822
C	-3.126557	-0.085999	-5.222120
C	1.389143	3.260073	-3.828793
C	-3.408808	0.721315	-3.030782
C	-2.823651	1.336132	-5.223783
C	-1.012352	0.918348	-6.846498
C	-2.930188	0.622244	-1.724100
C	0.106928	3.531625	-3.345672
C	-0.256003	3.146933	-1.991725
C	-2.999757	1.835634	-3.870104
C	-1.788137	1.827766	-6.019777
C	-1.639731	2.704345	-2.002089
C	-1.053407	3.318701	-4.196203
C	-0.884690	2.838419	-5.495345
C	-2.132345	2.806606	-3.366133

No imaginary frequency.

Table S32. Cartesian coordinates of 1b@C_{60} (B3LYP-D3(BJ)/6-31+G(d,p)).

Atom	X	Y	Z
C	-3.778164	1.384202	0.260736
C	-3.743946	0.478262	1.377051
C	-3.746249	0.941094	-1.106830
C	-3.416107	2.686585	0.502556
C	-3.772808	-0.927726	1.077092
C	-3.312431	0.932076	2.659933
C	-3.774938	-0.478780	-1.333263
C	-3.316821	1.826906	-2.140732
C	-2.969029	3.571369	-0.495825
C	-2.963377	3.151573	1.751158
C	-3.740845	-1.441497	-0.265704
C	-3.405844	-1.787315	2.083225
C	-2.907855	-0.009422	3.678448
C	-2.907368	2.304622	2.858146
C	-3.409566	-0.919178	-2.581633
C	-2.917672	3.181205	-1.834142
C	-2.908171	1.313830	-3.427998
C	-2.258924	4.654100	0.096693
C	-2.255033	4.375858	1.586040
C	-3.306101	-2.778175	-0.515308
C	-2.956364	-1.363682	3.347390
C	-2.949868	-3.099839	1.860364
C	-2.223833	0.365541	4.868867

C	-2.221857	2.762815	4.018523	H	-3.466913	1.541969	7.011196
C	-2.957015	-2.224484	-2.847986	H	-3.465093	3.202340	6.422290
C	-2.958525	-0.068571	-3.607950	H	-0.982827	-3.585635	-5.231858
C	-2.236071	4.027466	-2.753239	H	-0.983404	-0.499926	-6.317994
C	-2.224169	2.092213	-4.404006	H	-1.189748	4.068966	-6.094909
C	-1.602792	5.475915	-0.811312	H	-1.200013	5.391937	-4.973172
C	-1.592079	4.813398	2.725899	H	-3.480781	5.289572	-4.844941
C	-2.903515	-3.188296	-1.840913	H	-3.471937	3.949664	-5.988711
C	-2.895818	-3.634973	0.573230	H	-1.078221	-5.406640	-3.633641
C	-2.241006	-2.415992	3.986651	H	-1.062165	-6.345701	1.443863
C	-2.236817	-3.566771	3.001191	H	0.578975	2.636349	4.859995
C	-1.623881	-0.677150	5.586038	H	0.581519	0.984075	5.463990
C	-2.208981	1.859826	5.277339	H	1.098822	2.305960	6.516082
C	-1.625088	4.026230	3.919481	H	-5.680285	2.167893	6.135143
C	-2.244607	-2.249808	-4.080702	H	-5.010195	0.874983	5.134646
C	-2.245735	-0.820971	-4.584520	H	-5.008666	2.541382	4.544200
C	-1.642112	5.172779	-2.208285	H	0.572347	2.894868	-4.701559
C	-2.217526	3.633974	-4.251477	H	0.569740	4.251620	-3.581918
C	-1.623122	1.377001	-5.447751	H	1.088968	4.491999	-5.253620
C	-2.216570	-4.404674	-2.113196	H	-5.689871	4.210361	-4.951974
C	-2.207009	-4.866878	0.388044	H	-5.020972	3.991161	-3.331402
C	-1.581852	-2.038402	5.149940	H	-5.013710	2.647651	-4.480509
C	-1.570952	-4.770812	2.808497	H	-1.164677	-7.317625	-0.478541
C	-1.046695	2.203980	6.248261	H	-1.172450	-7.005265	-2.184663
C	-3.524246	2.154356	6.102526	H	-3.453275	-6.851888	-2.161937
C	-1.582400	-3.444020	-4.336901	H	-3.446177	-7.171441	-0.429482
C	-1.584391	-0.050122	-5.532476	H	0.595456	-5.526930	-0.150185
C	-1.056461	4.306827	-5.033200	H	0.591460	-5.222645	-1.882999
C	-3.533830	4.196241	-4.921286	H	1.114083	-6.794028	-1.266996
C	-1.619097	-4.502492	-3.376075	H	-5.665269	-6.412252	-1.176386
C	-2.196034	-5.505554	-1.023429	H	-5.001270	-4.897753	-1.798434
C	-1.605592	-5.411054	1.530121	H	-4.994567	-5.218514	-0.059856
C	0.382368	2.020634	5.738953	C	0.958604	0.919478	-2.855894
C	-4.878693	1.918211	5.432528	C	2.260306	1.076412	-3.333482
C	0.372932	3.964016	-4.615529	C	0.438480	1.829626	-1.848902
C	-4.887347	3.728897	-4.383900	C	0.441667	-0.408320	-2.573217
C	-1.031798	-6.515710	-1.213988	C	3.096405	2.149373	-2.820324
C	-3.509447	-6.370933	-1.177284	C	3.102090	-0.089668	-3.543779
C	0.396449	-5.979910	-1.122566	C	1.240020	2.859266	-1.356656
C	-4.865532	-5.676458	-1.043731	C	-0.397365	1.063540	-0.942343
H	-1.005839	6.324285	-0.488183	C	-0.395755	-0.319503	-1.388872
H	-0.994092	5.720567	2.727701	C	1.247860	-1.528222	-2.776917
H	-1.085053	-0.446259	6.498584	C	2.596657	3.022121	-1.852185
H	-1.084220	4.420872	4.772735	C	4.455343	1.645503	-2.711223
H	-1.105888	5.850140	-2.864039	C	2.606223	-1.365369	-3.269794
H	-1.083108	1.920608	-6.215329	C	4.458984	0.261732	-3.157792
H	-0.980587	-2.740522	5.720901	C	1.237498	3.170486	0.062515
H	-0.969367	-5.224071	3.591563	C	-0.399217	1.358526	0.419137
H	-1.183537	3.241322	6.575068	C	-0.391713	-1.351095	-0.452611
H	-1.185510	1.607522	7.157450	C	1.252588	-2.601617	-1.797800

C	3.435444	3.429776	-0.736958
C	5.260288	2.035407	-1.639924
C	3.448432	-2.342112	-2.597821
C	5.267145	-0.676538	-2.514303
C	2.595617	3.520372	0.446848
C	0.435833	2.431406	0.932374
C	-0.395016	0.280295	1.393632
C	0.448841	-2.516048	-0.661332
C	-0.393454	-1.046259	0.968399
C	2.610897	-3.107432	-1.688779
C	4.740330	2.945814	-0.632838
C	6.101728	1.057802	-0.969991
C	4.751383	-2.005083	-2.228481
C	6.104748	-0.270265	-1.398013
C	3.095676	3.121446	1.687489
C	0.957083	2.014030	2.222439
C	0.442103	0.685144	2.508325
C	0.968120	-2.931471	0.631279
C	0.446332	-2.024464	1.638986
C	3.110029	-3.507657	-0.448040
C	5.260415	2.530625	0.659487
C	6.101413	1.363556	0.451167
C	5.270684	-2.420168	-0.935773
C	6.107317	-1.348117	-0.422659
C	2.258551	2.353402	2.594089
C	4.454419	2.615795	1.795532
C	1.248105	-0.253355	3.152218
C	2.271545	-3.418635	0.736283
C	1.249338	-1.637939	2.711969
C	4.467112	-3.156259	-0.063919
C	6.103949	0.328801	1.387321
C	6.106972	-1.054778	0.941469
C	3.099440	1.375440	3.264953
C	4.456779	1.537395	2.770868
C	2.604554	0.098972	3.537760
C	3.109988	-3.014498	1.852668
C	2.609324	-2.141220	2.819694
C	4.467045	-2.850780	1.357676
C	5.264976	0.417306	2.570903
C	5.270688	-1.821564	1.849975
C	3.446753	-1.067849	3.331123
C	4.750009	-0.911749	2.856892

No imaginary frequency.

REFERENCES

1. S. Higashibayashi, H. Sakurai, Asymmetric synthesis of a chiral buckybowl, trimethylsumanene. *J. Am. Chem. Soc.* **130**, 8592–8593 (2008).

2. T. Amaya, H. Sakane, T. Muneishi, T. Hirao, Bowl-to-bowl inversion of sumanene derivatives. *Chem. Commun.* **6**, 765–767 (2008).

3. Q. Tan, S. Higashibayashi, S. Karanjit, H. Sakurai, Enantioselective synthesis of a chiral nitrogen-doped buckybowl. *Nat. Commun.* **3**, 891 (2012).

4. J. Liu, S. Osella, J. Ma, R. Berger, D. Beljonne, D. Schollmeyer, X. Feng, K. Müllen, Fused dibenzo[*a*,*m*]rubicene: A new bowl-shaped subunit of C_{70} containing two pentagons. *J. Am. Chem. Soc.* **138**, 8364–8367 (2016).

5. Y. Zou, W. Zeng, T. Y. Gopalakrishna, Y. Han, Q. Jiang, J. Wu, Dicyclopenta[4,3,2,1-*ghi*:4′,3′,2′,1′-*pqr*]perylene: A bowl-shaped fragment of fullerene C_{70} with global antiaromaticity. *J. Am. Chem. Soc.* **141**, 7266–7270 (2019).

6. Y. Tanaka, N. Fukui, H. Shinokubo, As-Indaceno[3,2,1,8,7,6-*ghijklm*]terrylene as a near-infrared absorbing C_{70}-fragment. *Nat. Commun.* **11**, 3873 (2020).

7. T. Guo, A. Li, J. Xu, K. K. Baldridge, J. Siegel, Enantiopure C_5 pentaindenocorannulenes: Chiral graphenoid materials. *Angew. Chem. Int. Ed.* **60**, 25809–25814 (2021).

8. K. Kawasumi, Q. Zhang, Y. Segawa, L. T. Scott, K. Itami, A grossly warped nanographene and the consequences of multiple odd-membered-ring defects. *Nat. Chem.* **5**, 739–744 (2013).

9. J. M. Fernandez-Garcia, P. J. Evans, S. M. Rivero, I. Fernandez, D. Garcia-Fresnadillo, J. Perles, J. Casado, N. Martín, π-Extended corannulene-based nanographenes: Selective formation of negative curvature. *J. Am. Chem. Soc.* **140**, 17188–17196 (2018).

10. K. Kato, Y. Segawa, L. T. Scott, K. Itami, A quintuple [6]helicene with a corannulene core as a C_5-symmetric propeller-shaped π-system. *Angew. Chem. Int. Ed.* **57**, 1337–1341 (2018).

11. D. Meng, G. Liu, C. Xiao, Y. Shi, L. Zheng, L. Jiang, K. K. Baldridge, Y. Li, J. S. Siegel, Z. Wang, Corannurylene pentapetalae. *J. Am. Chem. Soc.* **141**, 5402–5408 (2019).

12. K. Kise, S. Ooi, H. Saito, H. Yorimitsu, A. Osuka, T. Tanaka, Five-fold symmetric pentaindolo- and pentakis(benzoindolo)corannulenes: Unique structural dynamics derived from the combination of helical and bowl inversions. *Angew. Chem. Int. Ed.* **61**, e202112589 (2022).

13. X.-P. Zhang, S.-W. Ying, Y.-L. Zhang, W.-X. Zhang, W. Shi, B.-W. Chen, H.-R. Tian, G. Xu, S.-S. Wang, Q. Zhang, S.-Y. Xie, L.-S. Zheng, Decafluorinated and perfluorinated warped nanographenes: Synthesis, structural analysis, and properties. *J. Am. Chem. Soc.* **146**, 30913–30921 (2024).

14. M. A. Petrukhina, Coordination of buckybowls: The first concave-bound metal complex. *Angew. Chem. Int. Ed.* **47**, 1550–1552 (2008).

15. D. Bandera, K. K. Baldridge, A. Linden, R. Dorta, J. S. Siegel, Stereoselective coordination of C_5-symmetric corannulene derivatives with an enantiomerically pure [Rh^{I}(nbd*)] metal complex. *Angew. Chem. Int. Ed.* **50**, 865–867 (2011).

16. A. V. Zabula, S. N. Spisak, A. S. Filatov, A. Y. Rogachev, R. Clerac, M. A. Petrukhina, Supramolecular trap for a transient corannulene trianion. *Chem. Sci.* **7**, 1954–1961 (2016).

17. Z. Zhou, Z. Wei, Y. Tokimaru, S. Ito, K. Nozaki, M. A. Petrukhina, Stepwise reduction of azapentabenzocorannulene. *Angew. Chem. Int. Ed.* **58**, 12107–12111 (2019).

18. A. Y. Rogachev, S. Liu, Q. Xu, J. Li, Z. Zhou, S. N. Spisak, Z. Wei, M. A. Petrukhina, Placing metal in the bowl: Does rim alkylation matter? *Organometallics* **38**, 552–566 (2019).

19. H. Yokoi, Y. Hiraoka, S. Hiroto, D. Sakamaki, S. Seki, H. Shinokubo, Nitrogen-embedded buckybowl and its assembly with C_{60}. *Nat. Commun.* **6**, 8215 (2015).

20. Y.-M. Liu, D. Xia, B.-W. Li, Q.-Y. Zhang, T. Sakurai, Y.-Z. Tan, S. Seki, S.-Y. Xie, L.-S. Zheng, Functional sulfur-doped buckybowls and their concave-convex supramolecular assembly with fullerenes. *Angew. Chem. Int. Ed.* **55**, 13047–13051 (2016).

21. S. Lampart, L. M. Roch, A. K. Dutta, Y. Wang, R. Warshamanage, A. D. Finke, A. Linden, K. K. Baldridge, J. S. Siegel, Pentaindenocorannulene: Properties, assemblies, and C_{60} complex. *Angew. Chem. Int. Ed.* **55**, 14648–14652 (2016).

22. Y. Shoji, T. Kajitani, F. Ishiwari, Q. Ding, H. Sato, H. Anetai, T. Akutagawa, H. Sakurai, T. Fukushi, Hexathioalkyl sumanenes: An electron-donating buckybowl as a building block for supramolecular materials. *Chem. Sci.* **8**, 8405–8410 (2017).

23. Y.-Y. Xu, H.-R. Tian, S.-H. Li, Z.-C. Chen, Y.-R. Yao, S.-S. Wang, X. Zhang, Z.-Z. Zhu, S.-L. Deng, Q. Zhang, S. Yang, S.-Y. Xie, R.-B. Huang, L.-S. Zheng, Flexible decapyrrylcorannulene hosts. *Nat. Commun.* **10**, 485 (2019).

24. Y. Sun, X. Wang, B. Yang, M. Chen, Z. Guo, Y. Wang, J. Li, M. Xu, Y. Zhang, H. Sun, J. Dang, J. Fan, J. Li, J. Wei, Trichalcogenasupersumanenes and its concave-convex supramolecular assembly with fullerenes. *Nat. Commun.* **14**, 3446 (2023).

25. H. Liu, J. Guo, T. Che, C. Ding, B. Xu, Q. Tan, A nitrogen-containing graphenic buckybowl for photocatalysis and assembly with fullerenes. *Angew. Chem. Int. Ed.* **64**, e202506862 (2025).

26. T. Arnaya, S. Seki, T. Moriuchi, K. Nakamoto, T. Nakata, H. Sakane, A. Saeki, S. Tagawa, T. Hirao, Anisotropic electron transport properties in sumanene crystal. *J. Am. Chem. Soc.* **131**, 408–409 (2009).

27. J. Li, A. Terec, Y. Wang, H. Joshi, Y. Lu, H. Sun, M. C. Stuparu, π-Conjugated discrete oligomers containing planar and nonplanar aromatic motifs. *J. Am. Chem. Soc.* **139**, 3089–3094 (2017).

28. G. Gao, M. Chen, J. Roberts, M. Feng, C. Xiao, G. Zhang, S. Parkin, C. Risko, L. Zhang, Rational functionalization of a C_{70} buckybowl to enable a C_{70}: Buckybowl cocrystal for organic semiconductor applications. *J. Am. Chem. Soc.* **142**, 2460–2470 (2020).

29. X. Fu, Y. Zhen, Z. Ni, Y. Li, H. Dong, J. S. Siegel, W. Hu, One-pot domino carbonylation protocol for aromatic diimides toward n-type organic semiconductors. *Angew. Chem. Int. Ed.* **59**, 14024–14028 (2020).

30. S. Furukawa, J. Wu, M. Koyama, K. Hayashi, N. Hoshino, T. Takeda, Y. Suzuki, J. Kawamata, M. Saito, T. Akutagawa, Ferroelectric columnar assemblies from the bowl-to-bowl inversion of aromatic cores. *Nat. Commun.* **12**, 768 (2021).

31. Z.-L. Qiu, Y. Cheng, Q. Zeng, Q. Wu, X.-J. Zhao, R.-J. Xie, L. Feng, K. Liu, Y.-Z. Tan, Synthesis and interlayer assembly of a graphenic bowl with peripheral selenium annulation. *J. Am. Chem. Soc.* **145**, 3289–3293 (2023).

32. B. Yang, Y. Sun, J. Hu, S. Meng, M. Chen, X. Wang, M. Ren, J. Guo, Y. Wang, J. Li, M. Jia, J. Fan, H. Sun, J. Li, H. Xu, J. Wei, Triazasupersumanenes: Bowl-shaped nanographenes with tunable properties and unexpected charge transport performance. *Chem* **11**, 102628 (2025).

33. B. D. Steinberg, E. A. Jackson, A. S. Filatov, A. Wakamiya, M. A. Petrukhina, L. T. Scott, Aromatic π-systems more curved than C_{60}. The complete family of all indenocorannulenes synthesized by iterative microwave-assisted intramolecular arylations. *J. Am. Chem. Soc.* **131**, 10537–10545 (2009).

34. T. Amaya, T. Nakata, T. Hirao, Synthesis of highly strained π-bowls from sumanene. *J. Am. Chem. Soc.* **131**, 10810–10811 (2009).

35. T.-C. Wu, H.-J. Hsin, M.-Y. Kuo, C.-H. Li, Y.-T. Wu, Synthesis and structural analysis of a highly curved buckybowl containing corannulene and sumanene fragments. *J. Am. Chem. Soc.* **133**, 16319–16321 (2011).

36. T. Amaya, T. Ito, T. Hirao, Construction of a hemifullerene skeleton: A regioselective intramolecular oxidative cyclization. *Angew. Chem. Int. Ed.* **54**, 5483–5487 (2015).

37. C. F. Dickinson, J. K. Yang, G. P. A. Yap, M. A. Tius, Modular synthesis of a semibuckminsterfullerene. *Org. Lett.* **24**, 5095–5098 (2022).

38. G.-Y. Wu, C.-L. Huang, H.-W. Kang, W.-T. Ou, Y.-S. Ho, M.-J. Cheng, Y.-T. Wu, *exo*-6b^2-Methyl-substituted pentabenzocorannulene: Synthesis, structural analysis, and properties. *Angew. Chem. Int. Ed.* **63**, e202408321 (2024).

39. X. Li, Y. Zhu, J. Shao, B. Wang, S. Zhang, Y. Shao, X. Jin, X. Yao, R. Fang, X. Shao, Non-pyrolytic, large-scale synthesis of trichalcogenasumanene: A two-step approach. *Angew. Chem. Int. Ed.* **53**, 535–538 (2014).

40. S. Ito, Y. Tokimaru, K. Nozaki, Benzene-fused azacorannulene bearing an internal nitrogen atom. *Angew. Chem. Int. Ed.* **54**, 7256–7260 (2015).

41. S. Furukawa, Y. Suda, J. Kobayashi, T. Kawashima, T. Tada, S. Fujii, M. Kiguchi, M. Saito, Triphosphasumanene trisulfide: High out-of-plane anisotropy and janus-type π-surfaces. *J. Am. Chem. Soc.* **139**, 5787–5792 (2017).

42. Q. Tan, D. Zhou, T. Zhang, B. Liu, B. Xu, Iodine-doped sumanene and its application for the synthesis of chalcogenasumanenes and silasumanenes. *Chem. Commun.* **53**, 10279–10282 (2017).

43. S. Nakatsuka, N. Yasuda, T. Hatakeyama, Four-step synthesis of B_2N_2-embedded corannulene. *J. Am. Chem. Soc.* **140**, 13562–13565 (2018).

44. Y. Tokimaru, S. Ito, K. Nozaki, A hybrid of corannulene and azacorannulene: Synthesis of a highly curved nitrogen-containing buckybowl. *Angew. Chem. Int. Ed.* **57**, 9818–9822 (2018).

45. Q.-Q. Li, Y. Hamamoto, G. Kwek, B. Xing, Y. Li, S. Ito, Diazapentabenzocorannulenium: A hydrophilic/biophilic cationic buckybowl. *Angew. Chem. Int. Ed.* **61**, e202112638 (2022).

46. X. Zhang, M. R. Mackinnon, G. J. Bodwell, S. Ito, Synthesis of a π-extended azacorannulenophane enabled by strain-induced 1,3-dipolar cycloaddition. *Angew. Chem. Int. Ed.* **61**, e202116585 (2022).

47. W. E. Barth, R. G. Lawton, Dibenzo[*ghi*,*mno*]fluoranthene. *J. Am. Chem. Soc.* **88**, 380–381 (1966).

48. H. Sakurai, T. Daiko, T. Hirao, A synthesis of sumanene, a fullerene fragment. *Science* **301**, 1878 (2003).

49. L. T. Scott, E. A. Jackson, Q. Zhang, B. D. Steinberg, M. Bancu, B. Li, A short, rigid, structurally pure carbon nanotube by stepwise chemical synthesis. *J. Am. Chem. Soc.* **134**, 107–110 (2012).

50. K. Shoyama, F. Würthner, Synthesis of a carbon nanocone by cascade annulation. *J. Am. Chem. Soc.* **141**, 13008–13012 (2019).

51. Z.-Z. Zhu, Z.-C. Chen, Y.-R. Yao, C.-H. Cui, S.-H. Li, X.-J. Zhao, Q. Zhang, H.-R. Tian, P.-Y. Xu, F.-F. Xie, X.-M. Xie, Y.-Z. Tan, S.-L. Deng, J. M. Quimby, L. T. Scott, S.-Y. Xie, R.-B. Huang, L.-S. Zheng, Rational synthesis of an atomically precise carboncone under mild conditions. *Sci. Adv.* **5**, eaaw0982 (2019).

52. E. H. Fort, P. M. Donovan, L. T. Scott, Diels-alder reactivity of polycyclic aromatic hydrocarbon bay regions: Implications for metal-free growth of single-chirality carbon nanotubes. *J. Am. Chem. Soc.* **131**, 16606–16607 (2009).

53. U. H. F. Bunz, S. Menning, N. Martín, *para*-Connected cyclophenylenes and hemispherical polyarenes: Building blocks for single-walled carbon nanotubes? *Angew. Chem. Int. Ed.* **51**, 7094–7101 (2012).

54. K. T. Rim, M. Siaj, S. Xiao, M. Myers, V. D. Carpentier, L. Liu, C. Su, M. L. Steigerwald, M. S. Hybertsen, P. H. McBreen, G. W. Flynn, C. Nuckolls, Forming aromatic hemispheres on transition-metal surfaces. *Angew. Chem. Int. Ed.* **46**, 7891–7895 (2007).

55. A. C. Whalley, K. N. Plunkett, A. A. Gorodetsky, C. L. Schenck, C.-Y. Chiu, M. L. Steigerwald, C. Nuckolls, Bending contorted hexabenzocoronene into a bowl. *Chem. Sci.* **2**, 132–135 (2011).

56. B. Chan, Y. Kawashima, M. Katouda, T. Nakajima, K. Hirao, From C_{60} to infinity: Large-scale quantum chemistry calculations of the heats of formation of higher fullerenes. *J. Am. Chem. Soc.* **138**, 1420–1429 (2016).

57. R. Haddon, Comment on the relationship of the pyramidalization angle at a conjugated carbon atom to the σ bond angles. *J. Phys. Chem. A* **105**, 4164–4165 (2001).

58. A. Sygula, A. Abdourazak, P. Rabideau, Cyclopentacorannulene: π-Facial stereoselective deuterogenation and determination of the bowl-to-bowl inversion barrier for a constrained buckybowl. *J. Am. Chem. Soc.* **118**, 339–343 (1996).

59. T. Amaya, H. Sakane, T. Nakata, T. Hirao, A theoretical study of the bowl-to-bowl inversion of sumanene derivatives. *Pure Appl. Chem.* **82**, 969–978 (2010).

60. T. M. Krygowski, Crystallographic studies of inter- and intramolecular interactions reflected in aromatic character of .pi.-electron systems. *J. Chem. Inf. Comput. Sci.* **33**, 70–78 (1993).

61. H. Fallah-Bagher-Shaidaei, C. Wannere, C. Corminboeuf, R. Puchta, P. Schleyer, Which NICS aromaticity index for planar π rings is best? *Org. Lett.* **8**, 863–866 (2006).

62. D. Geuenich, K. Hess, F. Köhler, R. Herges, Anisotropy of the induced current density (ACID), a general method to quantify and visualize electronic delocalization. *Chem. Rev.* **105**, 3758–3772 (2005).

63. H. Fliegl, S. Taubert, O. Lehtonen, D. Sundholm, The gauge including magnetically induced current method. *Phys. Chem. Chem. Phys.* **13**, 20500–20518 (2011).

64. T. Seiders, K. Baldridge, G. Grube, J. Siegel, Structure/energy correlation of bowl depth and inversion barrier in corannulene derivatives: Combined experimental and quantum mechanical analysis. *J. Am. Chem. Soc.* **123**, 517–525 (2001).

65. R. Rieger, K. Müllen, Forever young: Polycyclic aromatic hydrocarbons as model cases for structural and optical studies. *J. Phys. Org. Chem.* **23**, 315–325 (2010).

66. T. Lu, F. Chen, Multiwfn: A multifunctional wavefunction analyzer. *J. Comput. Chem.* **33**, 580–592 (2012).

67. T. Lu, Q. Chen, Independent gradient model based on hirshfeld partition: A new method for visual study of interactions in chemical systems. *J. Comput. Chem.* **43**, 539–555 (2022).

68. T. Lu, Q. Chen, Simple, efficient, and universal energy decomposition analysis method based on dispersion-corrected density functional theory. *J. Phys. Chem. A* **127**, 7023–7035 (2023).

69. D. B. Hibbert, P. Thordarson, The death of the job plot, transparency, open science and online tools, uncertainty estimation methods and other developments in supramolecular chemistry data analysis. *Chem. Commun.* **52**, 12792–12805 (2016).

70. P. Thordarson, Determining association constants from titration experiments in supramolecular chemistry. *Chem. Soc. Rev.* **40**, 1305–1323 (2011).

71. Bindfit, (2016); http://supramolecular.org.

72. K. Campbell, A. Zappas, U. Bunz, Y. S. Thio, D. G. Bucknall, Fluorescence quenching of a poly(*para*-phenylene ethynylenes) by C_{60} fullerenes. *J. Photochem. Photobiol. A Chem.* **249**, 41–46 (2012).

73. B. Li, Z. Chao, C. Li, Z. Gu, Cu-catalyzed enantioselective ring opening of cyclic diaryliodoniums toward the synthesis of chiral diarylmethanes. *J. Am. Chem. Soc.* **140**, 9400–9403 (2018).

74. A. M. M. Heuer, S. C. C. Coste, G. Singh, B. Q. Q. Mercado, J. M. M. Mayer, A guide to tris(4-substituted)-triphenylmethyl radicals. *J. Org. Chem.* **88**, 9893–9901 (2023).

75. S. Rashidnadimi, T. H. Hung, K.-T. Wong, A. J. Bard, Electrochemistry and electrogenerated chemiluminescence of 3,6-di(spirobifluorene)-N-phenylcarbazole. *J. Am. Chem. Soc.* **130**, 634–639 (2008).

76. M. J. Frisch, G. W. Trucks, H. B. Schlegel, G. E. Scuseria, M. A. Robb, J. R. Cheeseman, G. Scalmani, V. Barone, G. A. Petersson, H. Nakatsuji, X. Li, M. Caricato, A. V. Marenich, J. Bloino, B. G. Janesko, R. Gomperts, B. Mennucci, H. P. Hratchian, J. V. Ortiz, A. F. Izmaylov, J. L. Sonnenberg, D. Williams-Young, F. Ding, F. Lipparini, F. Egidi, J. Goings, B. Peng, A. Petrone, T. Henderson, D. Ranasinghe, V. G. Zakrzewski, J. Gao, N. Rega, G. Zheng, W. Liang, M. Hada, M. Ehara, K. Toyota, R. Fukuda, J. Hasegawa, M. Ishida, T. Nakajima, Y. Honda, O. Kitao, H. Nakai, T. Vreven, K. Throssell, J. A. Montgomery, Jr., J. E.

Peralta, F. Ogliaro, M. J. Bearpark, J. J. Heyd, E. N. Brothers, K. N. Kudin, V. N. Staroverov, T. A. Keith, R. Kobayashi, J. Normand, K. Raghavachari, A. P. Rendell, J. C. Burant, S. S. Iyengar, J. Tomasi, M. Cossi, J. M. Millam, M. Klene, C. Adamo, R. Cammi, J. W. Ochterski, R. L. Martin, K. Morokuma, O. Farkas, J. B. Foresman, D. J. Fox, Gaussian 16, revision A.03 (Gaussian Inc., 2016).

77. G. M. Sheldrick, SHELXT–Integrated space-group and crystal-structure determination. *Acta. Crystallogr. A Found. Adv*. **71**, 3–8 (2015).

78. G. M. Sheldrick, Crystal structure re inement with SHELXL. *Acta Crystallogr. C Struct. Chem.* **71**, 3–8 (2015).

79. O. V. Dolomanov, L. J. Bourhis, R. J. Gildea, J. A. K. Howard, H. Puschmann, OLEX2: A complete structure solution, re inement and analysis program. *J. Appl. Cryst*. **42**, 339–341 (2009).

80. M. Zheng, F. Bai, F. Li, Y. Li, D. Zhu, The interaction between conjugated polymer andfullerenes. *J. Appl. Polym. Sci*. **70**, 599–603 (1998).
